# Heterobifunctional Ligase Recruiters Enable pan-Degradation of Inhibitor of Apoptosis Proteins

**DOI:** 10.1021/acs.jmedchem.2c01817

**Published:** 2023-03-30

**Authors:** Yuen Lam Dora Ng, Aleša Bricelj, Jacqueline A. Jansen, Arunima Murgai, Kirsten Peter, Katherine A. Donovan, Michael Gütschow, Jan Krönke, Christian Steinebach, Izidor Sosič

**Affiliations:** † Department of Hematology, Oncology and Cancer Immunology, Charité—Universitätsmedizin Berlin, Corporate Member of Freie Universität Berlin and Humboldt-Universität zu Berlin, D-12203 Berlin, Germany; ‡ Faculty of Pharmacy, University of Ljubljana, Aškerčeva Cesta 7, SI-1000 Ljubljana, Slovenia; § German Cancer Consortium (DKTK) Partner Site Berlin and German Cancer Research Center (DKFZ), D-69120 Heidelberg, Germany; ∥ Department of Cancer Biology, Dana-Farber Cancer Institute, Boston, Massachusetts 02215, United States; ⊥ Department of Biological Chemistry and Molecular Pharmacology, Harvard Medical School, Boston, Massachusetts 02215, United States; # Phamaceutical Institute, Department of Pharmaceutical & Medicinal Chemistry, University of Bonn, An der Immenburg 4, D-53121 Bonn, Germany

## Abstract

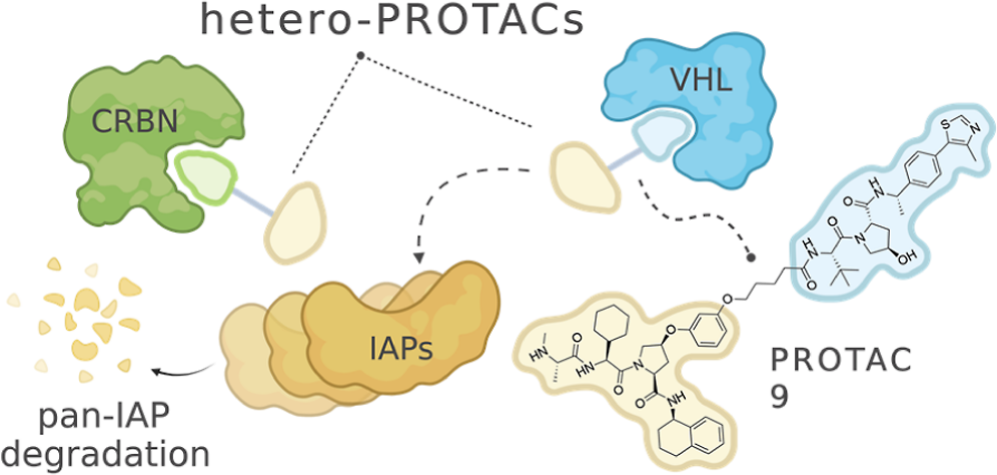

Proteolysis targeting chimeras (PROTACs) represent a new pharmacological modality to inactivate disease-causing proteins. PROTACs operate via recruiting E3 ubiquitin ligases, which enable the transfer of ubiquitin tags onto their target proteins, leading to proteasomal degradation. However, several E3 ligases are validated pharmacological targets themselves, of which inhibitor of apoptosis (IAP) proteins are considered druggable in cancer. Here, we report three series of heterobifunctional PROTACs, which consist of an IAP antagonist linked to either von Hippel-Lindau- or cereblon-recruiting ligands. Hijacking E3 ligases against each other led to potent, rapid, and preferential depletion of cellular IAPs. In addition, these compounds caused complete X-chromosome-linked IAP knockdown, which was rarely observed for monovalent and homobivalent IAP antagonists. In cellular assays, hit degrader **9** outperformed antagonists and showed potent inhibition of cancer cell viability. The hetero-PROTACs disclosed herein are valuable tools to facilitate studies of the biological roles of IAPs and will stimulate further efforts toward E3-targeting therapies.

## Introduction

In the last decade, significant advancements have been made in the field of proteolysis targeting chimeras (PROTACs). PROTACs are now recognized as one of the most promising modalities with the potential to promote the development of targeted therapy drugs.^1−6^ Classical PROTACs are heterobifunctional compounds comprising a ligand that binds to a target protein of interest, a ligand that binds to and recruits an E3 ubiquitin ligase, and a linker tether. Such molecules can facilitate the formation of a ternary target–PROTAC–E3 ligase complex, followed by ubiquitination of the target protein and its subsequent degradation by the proteasome.^7^ PROTACs possess several advantages over conventional inhibitors as they exert their action catalytically, resulting in a potent intracellular degradation of the target proteins. Moreover, PROTACs can discriminate between similar proteins within the same family or even protein isoforms, thus allowing exclusive target-selective degradation.^8,9^

E3 ubiquitin ligases orchestrate protein turnover via facilitating substrate proximity and ubiquitin transfer. They encompass a diverse group of more than 600 enzymes, with most E3 ligases belonging to the really interesting new gene (RING) family. Many have crucial roles in various biological processes^10^ but are also implicated in multiple diseases. Therefore, targeting E3 ligases is considered an attractive approach for small-molecule drugs.^11−15^ Cellular RING E3 ligases are large multi-subunit complexes but usually do not possess a defined ligand-binding site rendering the inhibitor design difficult. Nevertheless, traditional approaches yielded potent compounds targeting murine double minute 2 (MDM2), von Hippel-Lindau (VHL), and inhibitor of apoptosis (IAP) proteins.^11^ The consequence of binding to these E3 ligases is disrupting protein–protein interactions between ligases and their respective substrates. E3 ligases can also be degraded via proximity-induced ubiquitination. Namely, several homodimeric E3 degraders have been developed by linking two identical E3 ligase ligands.^16−19^

**Figure 1:**
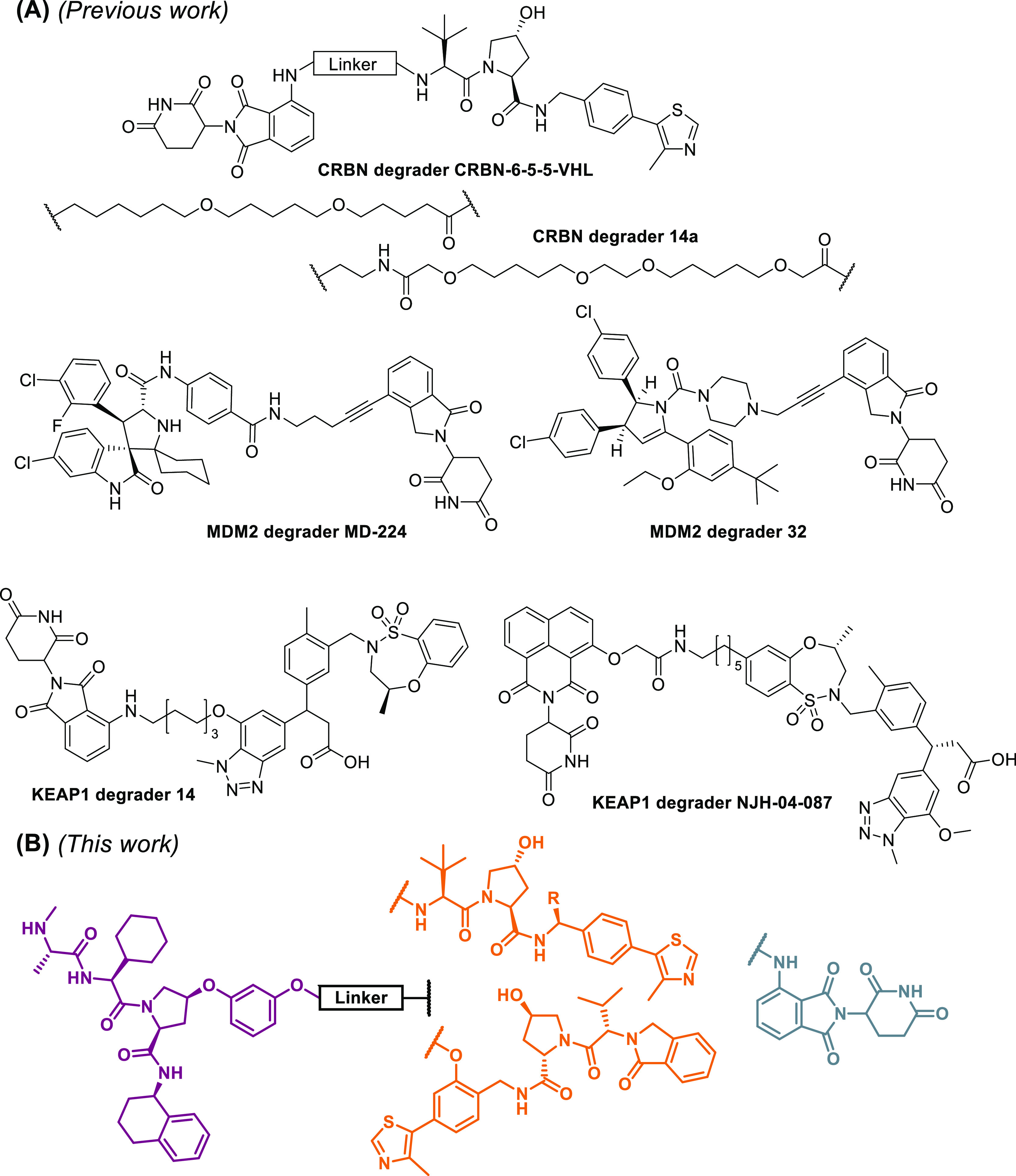
(A) Examples of heterobifunctional E3 ligase degraders. **CRBN-6-5-5-VHL** and **14a** were shown to be potent CRBN degraders which utilize VHL as the recruited ligase,^20,21^ whereas **MD-224** and **PROTAC 32** cause depletion of MDM2 via CRBN-mediated ubiquitination.^18,23^**PROTAC 14** and **NJH-04-087** exhibited degradation of KEAP1 over CRBN.^24,25^ (B) Schematic representation of the designed compounds in this work.

Directing different E3 ligases against each other by heterodimeric PROTACs also proved to be a productive strategy for their depletion. We recently reported preferential degradation of cereblon (CRBN) over VHL with molecules assembled from pomalidomide-based CRBN binders and a VHL ligand (**CRBN-6-5-5-VHL**, Figure 1A).^20^ The prevalence of VHL over CRBN was also observed in a separate study by Ciulli and colleagues (**14a**, Figure 1A).^21^ On the contrary, linking MDM2 inhibitors to lenalidomide resulted in MDM2 degradation (**MD-224** and **PROTAC 32**, Figure 1A).^22,23^ Of note, CRBN levels were not monitored for the latter two examples, thereby not entirely confirming the unilateral degradation of MDM2. Recently, the compendium of heterobifunctional ligase degraders was extended by KEAP1-CRBN recruiters (**PROTAC 14** and **NJH-04-087**, Figure 1A) that preferentially degrade KEAP1.^24,25^

Cellular IAP1 (cIAP1/BIRC2), cellular IAP2 (cIAP2/BIRC3), and X-chromosome-linked IAP (XIAP/BIRC4) have been studied in great detail because of their critical role in controlling the apoptotic machinery.^26^ Their overexpression has been linked to tumor progression, resistance to anti-cancer therapies, and poor prognosis.^27−29^ This clinical significance has translated to the development of numerous mimetics of the IAP-binding motif (i.e., the *N*-terminal Ala-Val-Pro-Ile moiety)^30−32^ of the second mitochondria-derived activator of caspases (SMAC), which functions as an endogenous IAP antagonist.^33,34^ Several SMAC-mimicking IAP monovalent and bivalent antagonists have entered into clinical trials for the treatment of various cancers (Figure S1).^35,36^ However, they demonstrated low efficacy as single agents, and current clinical evaluations are limited to combination studies with other cytotoxic drugs, radiation, and immunotherapy.^37^

IAP antagonists have profound effects on cIAPs levels. Both monovalent and bivalent SMAC mimetics bind to the baculoviral IAP repeat (BIR) type 3 domain of IAPs. This event promotes a rapid and ubiquitin- and proteasome-dependent loss of cIAP1 and cIAP2.^38,39^ It was suggested that BIR3-binding IAP antagonists destabilize a closed/autoinhibited form of cIAP (by blocking the crucial BIR3-RING domain interactions), resulting in dimerization, E3 ubiquitin ligase activation, autoubiquitination, and proteasomal degradation.^40,41^ Although most IAP antagonists do also possess an affinity for the BIR3 domain of XIAP, this rarely results in XIAP autodegradation.^38^ IAP antagonists such as bestatin and more recently the improved LCL161 also represent valuable E3-recruiter moieties for the assembly of heterobifunctional protein degraders.^42^

Encouraged by our successful outcomes with heterodimerizing PROTACs and the fact that IAPs are validated anti-cancer targets, we decided to include IAPs in the hetero-PROTAC approach for E3 modulation (Figure 1B). We systematically designed three series of bifunctional molecules. Two series were assembled by linking the selected IAP ligand^43^ to two VHL ligands with differently oriented exit vectors. In the third series, the CRBN ligand pomalidomide was incorporated into hetero-PROTACs (for E3 ligand structures, see Table S1). Our efforts to induce IAP degradation through hijacking another E3 ligase via hetero-PROTACs led to selective depletion of IAPs, including XIAP, and, in one case, also to selective XIAP degradation. Notably, the pan-IAP deficit could translate into very potent inhibitors of cancer cell viability, thus further substantiating the rationale of our strategy.

## Results and Discussion

### Design and Synthesis of Bifunctional IAP PROTACs

**Scheme 1:**
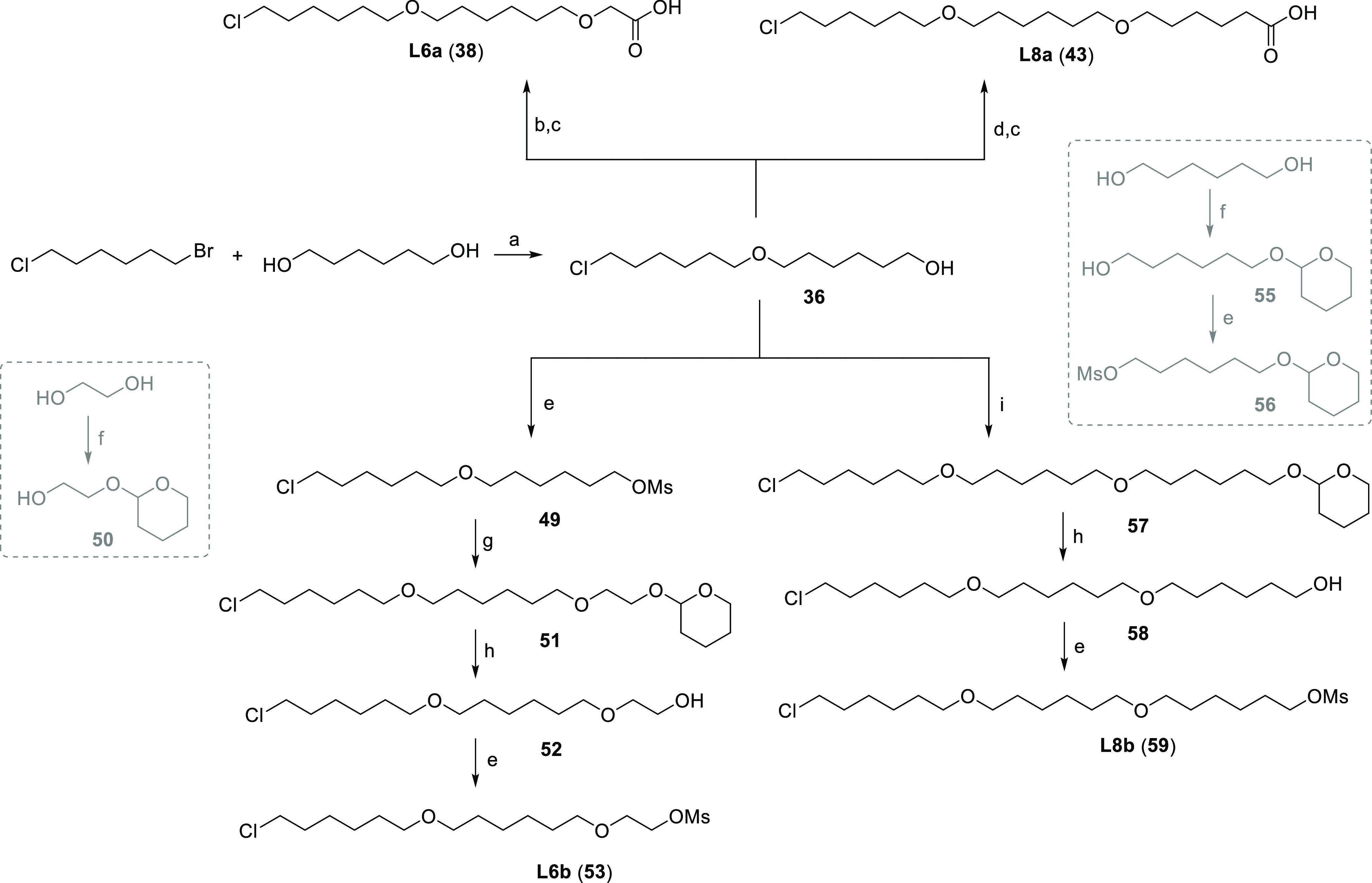
Syntheses of Linkers **L6a**, **L8a** and **L6b**, **L8b** Reagents and conditions: (a) 50% NaOH (aq), DMSO, rt, 24 h; (b) *tert*-butyl bromoacetate, NaH, DMF/THF, 0 °C to rt, 18 h; (c) TFA, CH_2_Cl_2_, 40 °C, 2 h; (d) *tert*-butyl 6-bromohexanoate (**41**), toluene, 50% NaOH (aq), TBAHS, rt, 18 h; (e) MsCl, DIPEA, CH_2_Cl_2_, rt, 2 h; (f) 3,4-dihydro-2*H*-pyran, CuSO_4_ × 5 H_2_O, MeCN, rt, 4 h; (g) **50**, TBAHS, 50% NaOH (aq), toluene, rt, 18 h; (h) *p*TsOH × H_2_O, MeOH, rt, 20 h; (i) **56**, TBAHS, 50% NaOH (aq), toluene, rt, 18 h.

**Scheme 2:**
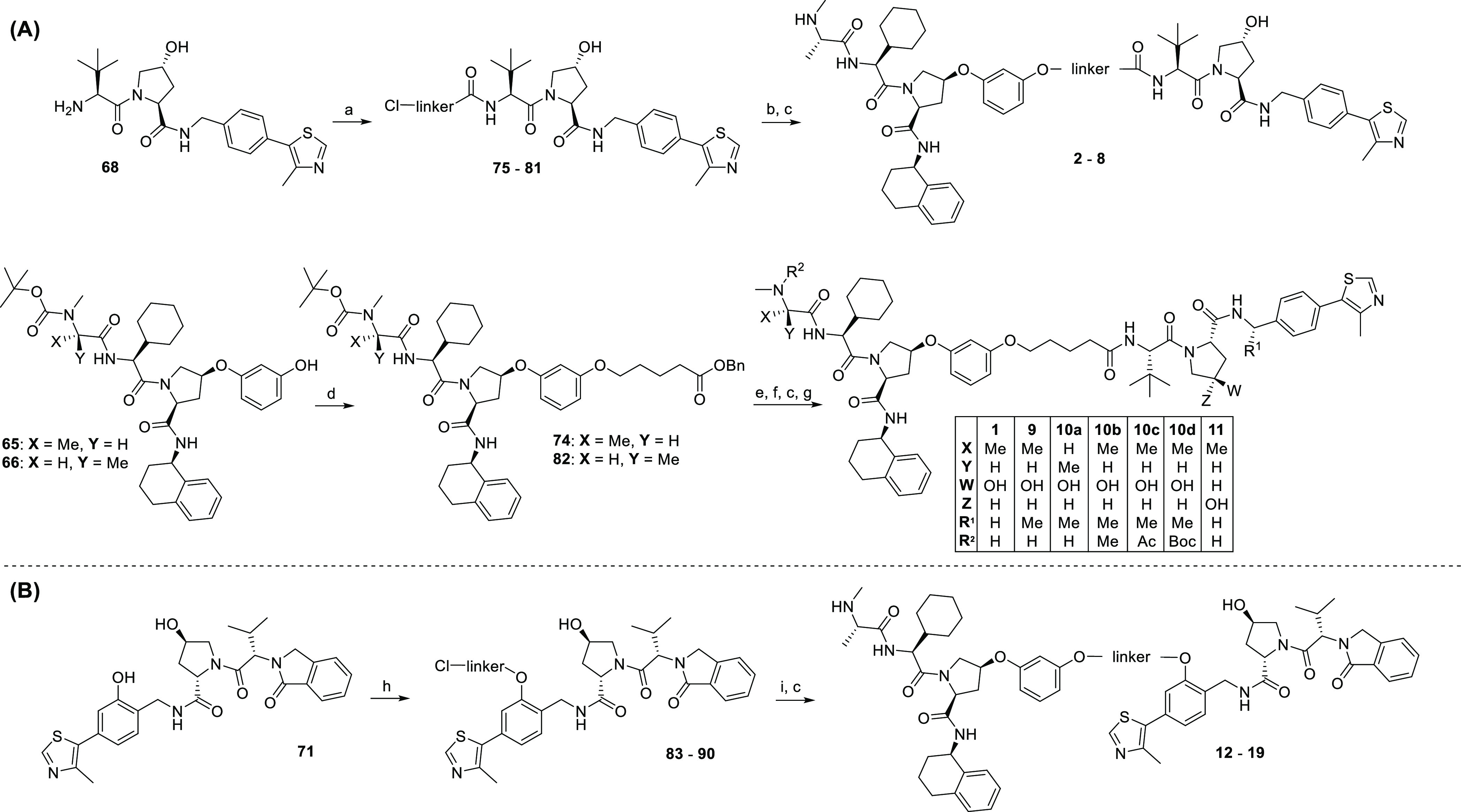
(A) Syntheses of the IAP-VHL Series 1 Hetero-PROTACs **2**–**8**, **1**, and **9** and Negative Controls **10a–d** and **11** Reagents and conditions: (a) linker **L2a–L8a** (Table S2), HATU, DIPEA, DMF, rt, 16 h; (b) step 1: NaI, acetone, 60 °C, 48 h; step 2: **65**, Cs_2_CO_3_, DMF, 60 °C, 16 h; (c) 1 M HCl in EtOAc, rt, 4 h; (d) step 1: linker **L1a** (Table S2), NaI, acetone, 60 °C, 48 h; step 2: Cs_2_CO_3_, DMF, 60 °C, 16 h; (e) Pd/C, H_2_, EtOAc, rt, 18 h; (f) **68** or **69**, HATU, DIPEA, DMF, rt, 16 h; (g) **9**, formaldehyde, Pd/C, H_2_, DMF, rt, 16 h (for compound **10b**) or **9**, Ac_2_O, DIPEA, CH_2_Cl_2_, 0 °C to rt, 16 h (for compound **10c**); (B) synthesis of the IAP-VHL series 2 hetero-PROTACs **12–19**. Reagents and conditions: (h) linker **L1b–L8b** (Table S3), Cs_2_CO_3_, DMF, rt, 16 h, then 60 °C, 3 h; (i) step 1: NaI, acetone, 60 °C, 48 h and step 2: **65**, Cs_2_CO_3_, DMF, 60 °C, 16 h.

It is widely accepted that linkers play a significant role in PROTAC activity, as even subtle differences in length or composition influence the degradation activity and selectivity.^44,45^ Three libraries of heterobifunctional PROTACs were systematically designed with eight linkers of varying length and chemical composition in each library (Tables S2–S6). The synthesis of the first series of IAP-VHL hetero-PROTACs (Series 1) was accomplished by a straightforward approach employing chloro to carboxylic acid (Cl-to-CO_2_H) linkers **L1a–L8a** (Table S2). Most of the linkers were acquired by a BAIB/TEMPO-mediated oxidation of the appropriate primary alcohol precursors. In contrast, **L6a**, **L7a**, and **L8a** were prepared by elongating C6–O–C6 or C6–O–C5 alcohols with *tert*-butyl bromoacetate, *tert*-butyl 5-bromopentanoate, or *tert*-butyl 6-bromohexanoate (Schemes 1 and S1), respectively, followed by deprotection of the *tert*-butyl ester. Linkers **L2a–L8a** were first coupled to the VHL ligand VH032 (VHL1 ligand, **68**),^46^ and the obtained conjugates **75–81** were applied to alkylate the Boc-protected IAP ligand **65** (Scheme 2A). Because intramolecular cyclization occurred when coupling the C5 linker **L1a** to the VHL1 ligand, **L1a** was first attached to the Boc-protected IAP ligand **65** or to the negative control IAP ligand **66**. This was followed by deprotection of the terminal carboxylic acid before coupling with either VH032 (**68**) or a methylated VHL ligand (Me-VHL1, **69**) which is known to enhance the VHL-binding affinity.^47^ The desired hetero-PROTACs **1**–**9** and controls **10a** and **11** were obtained following Boc deprotection of the corresponding IAP ligands (Scheme 2A). Additional control compounds were obtained by derivatization of PROTAC **9** into dimethyl- (**10b**) or acetyl- (**10c**) bearing analogues.

For the second IAP-VHL series (Series 2), a different exit vector at the VHL side was employed, presumably leading to differently oriented ternary complexes.^48,49^ For this subseries, methane-sulfonate to chloro (OMs-to-Cl) linkers **L1b–L8b** (Table S3) were utilized as crucial building blocks. Most of these (**L1b–L5b**) were prepared by subjecting the selected alcohols to mesylation. At the same time, **L6b**, **L7b**, and **L8b** were obtained through elongation of C6–O–C6 or C6–O–C5 mesylates with the corresponding tetrahydropyranyl (THP)-protected diols. Following cleavage of the THP-protecting group, mesylates were prepared (Schemes 1 and S5–S7). The obtained linkers were attached to the phenolic VHL ligand (VHL2 ligand, **71**) through *O*-alkylation. These ligand–linker conjugates **83–90** were then connected to the IAP ligand **65**, followed by removing the Boc-protecting group under acidic conditions to yield hetero-PROTACs **12–19** (Scheme 2B).

**Scheme 3:**
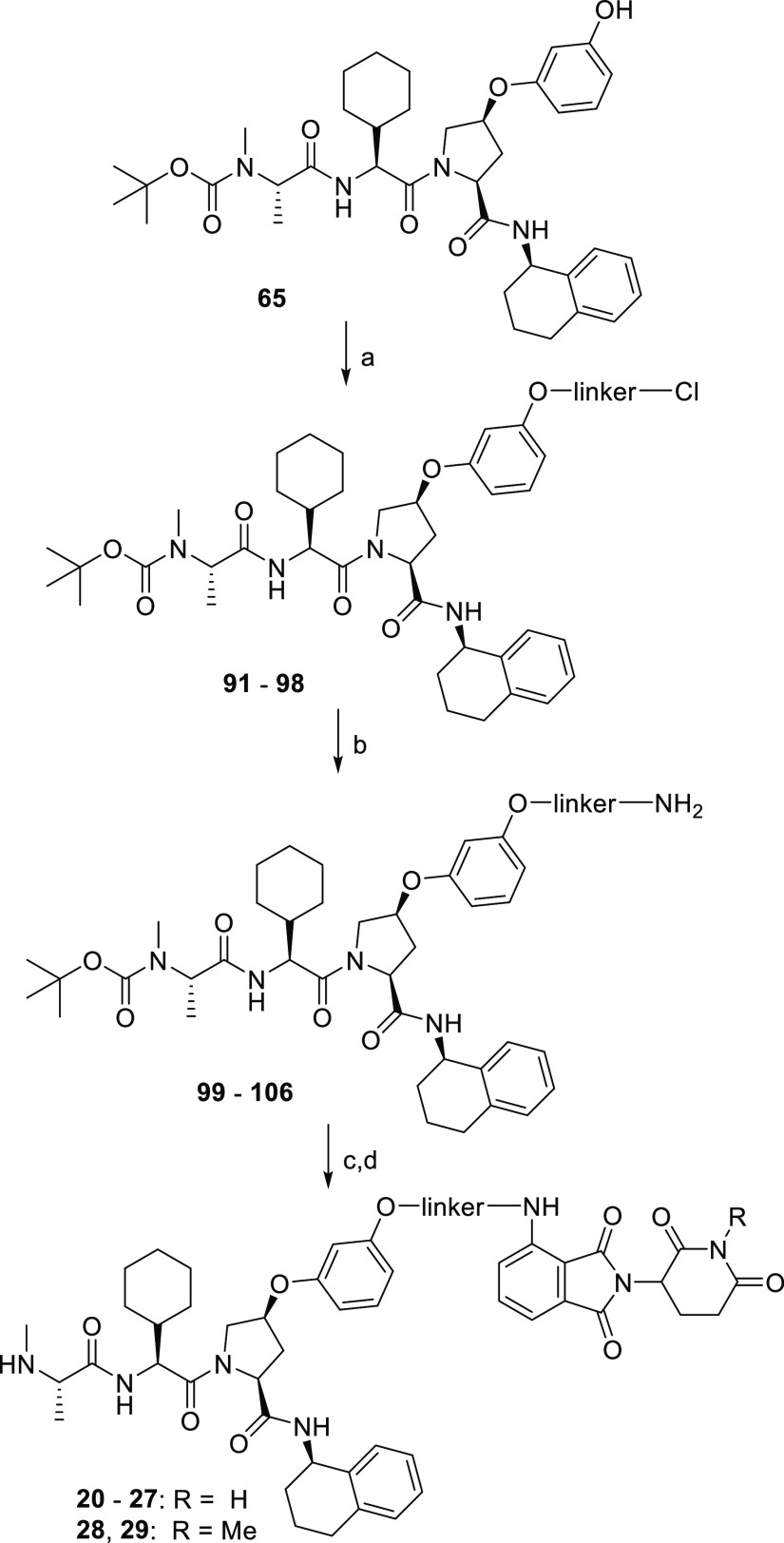
Synthesis of the IAP-CRBN Series Hetero-PROTACs **20–29** Reagents and conditions: (a) linkers **L1b–L8b** (Table S3), K_2_CO_3_, DMF, 70 °C, 20 h; (b) step 1: NaN_3_, DMF, 80 °C, 4 h; step 2: Pd/C, H_2_, MeOH, rt, 3 h; (c) **72** or **73**, DIPEA, DMSO, 90 °C, 20 h; (d) 1 M HCl in EtOAc, rt, 4 h.

In the third series of hetero-PROTACs, OMs-to-Cl linkers were used to alkylate the IAP ligand **65**, and the resulting chloro-linker-IAP ligand conjugates **91–98** were converted to amino-functionalized building blocks via azidolysis and hydrogenolysis. Finally, these amine building blocks **99–106** were reacted with 4-fluorothalidomide (**72**) in a nucleophilic aromatic substitution. Removal of the Boc protecting group yielded the envisioned IAP-CRBN heterobifunctional PROTACs **20–27**. For the assembly of negative control compounds **28** and **29**, an *N*-methylated thalidomide derivative **73** was used in place of 4-fluorothalidomide (**72**) (Scheme 3).

**Figure 2:**
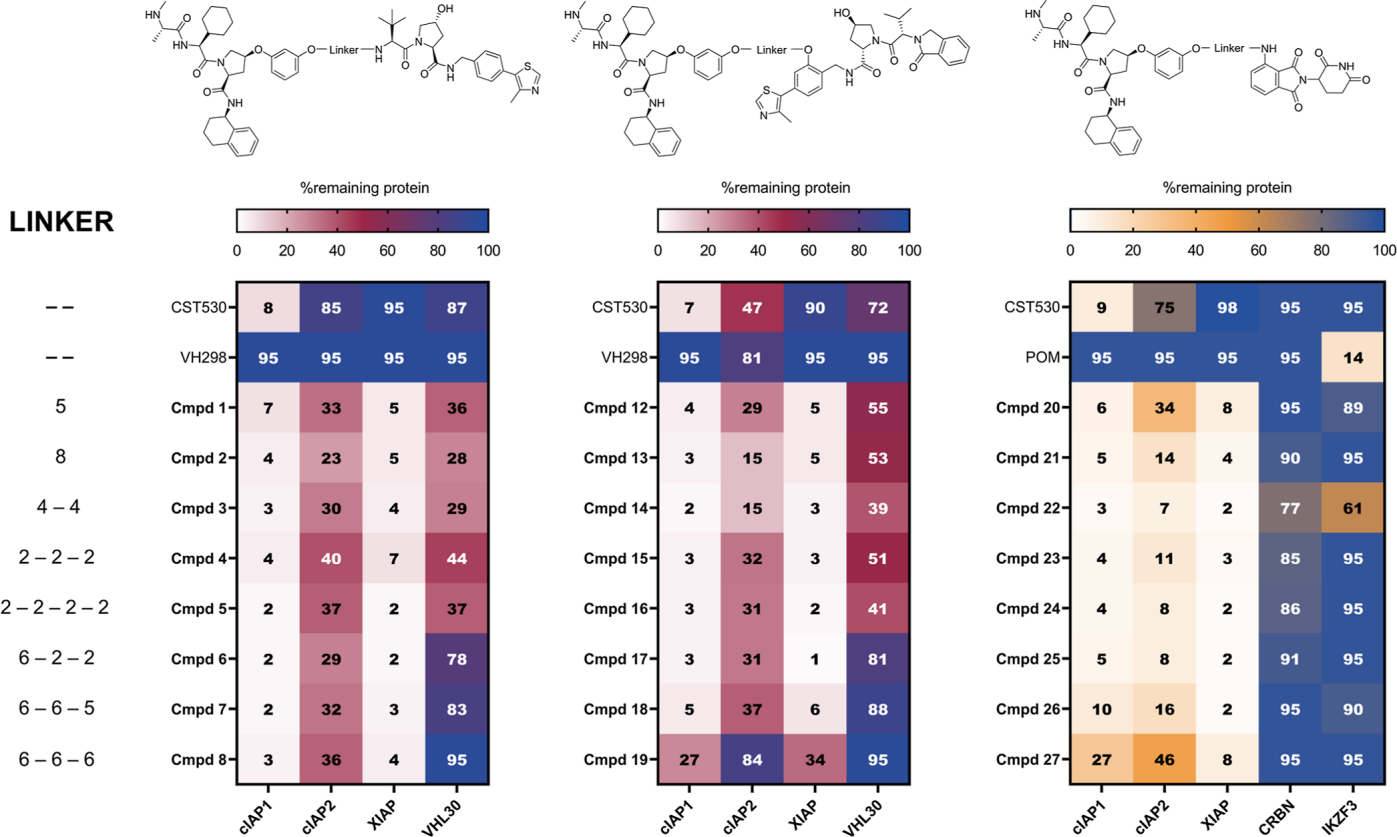
Degradation profiles of Series 1 (left), Series 2 (middle), and Series 3 (right) of hetero-PROTACs on cIAP1, cIAP2, XIAP, VHL30, CRBN, and IKZF3 expression levels. Percentage degradation is indicated as the remaining protein levels after MM.1S cells were subjected to 16 h treatment with each compound at 0.1 μM. Values are normalized to respective loading controls and to DMSO-treated conditions. All data represent an average of at least three independent experiments. CST530: IAP ligand and VH298: VHL ligand.

The physicochemical properties of all hetero-PROTACs (Tables S4–S6) and known IAP inhibitors (Table S7) are provided in the Supporting Information. Despite encompassing a wide range in terms of lipophilicity (*e*log *D*_7.4_ from 3.4 to 5.8), a similar activity window was observed for IAP-VHL Series 1 PROTACs. In Series 2, the most lipophilic hetero-PROTAC **19** showed the lowest IAP degradation levels at 0.1 μM, whereas for IAP-CRBN series, high PROTAC lipophilicity led to XIAP-selective depletion (Figure 2 and Table S6).

### Hetero-PROTACs Induce Potent pan-IAP Degradation

**Figure 3:**
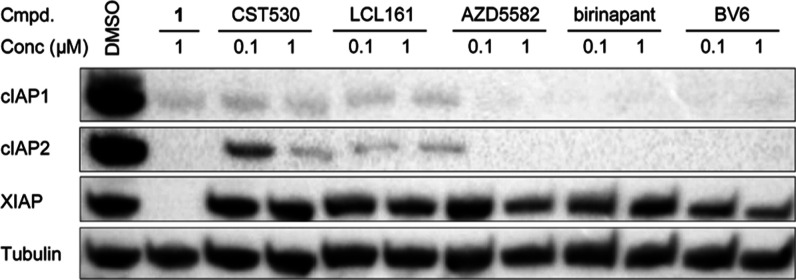
Monovalent (CST530 and LCL161) and bivalent IAP antagonists (AZD5582, birinapant, and BV6) induce potent cIAP autodegradation, whereas the IAP-VHL hetero-PROTAC **1** is a pan-IAP degrader. MM.1S cells were treated at 0.1 or 1 μM for 16 h.

At the outset of our studies, we were aware of the different outcomes possible, as the E3 ligase crosstalk can result in a favored degradation of one ligase or depletion of both E3s in the case of simultaneous cross- and/or autoubiquitination. In the case of preferred ubiquitination of IAP(s), our compounds might be degraders of either cIAP1, cIAP2, XIAP, or of two or even three IAPs. To evaluate the capability of our panel of hetero-PROTACs for E3 ligase degradation, VHL19, VHL30, CRBN, cIAP1, cIAP2, and XIAP levels were quantified by western blot analyses. For each series, MM.1S cells were treated with 0.1 or 1 μM of each compound for 16 h. Original blots are provided in the Supporting Information (Figures S2–S4), and an overview is given in Figure 2. The IAP antagonists LCL161, AZD5582, birinapant, and BV6 were assessed as comparators (Figure 3). These monovalent and bivalent IAP-targeting compounds led to substantial degradation of cIAP1 and cIAP2 at concentrations as low as 0.1 μM. The bivalent IAP antagonists performed better than the monovalent SMAC mimetics, but all of these compounds failed to degrade XIAP (Figure 3).

The effect of representatives from Series 1 on IAP depletion was significantly enhanced in comparison to the incorporated IAP ligand CST530 itself (Figures 2, 3, and S2), which caused only potent cIAP1 autodegradation but moderate cIAP2 depletion in MM.1S cells. As expected, treatment with the VHL inhibitor VH298 did not affect the levels of IAPs. Hetero-PROTACs, on the other hand, induced complete cIAP1 and XIAP degradation even at 0.1 μM. In addition, a substantial reduction of cIAP2 levels was observed for these hetero-PROTACs (Figures 2 and S2–S4). We were particularly pleased with the ability of PROTACs to degrade XIAP, which was rarely down-regulated by monovalent or bivalent IAP ligands in all previous studies. Of note, after a 16 h treatment with 0.1 μM concentration, a reduction of VHL30 levels was observed for all Series 1 PROTACs (Figures 2 and S2). However, this effect was less pronounced for compounds containing long and hydrophobic linkers (i.e., PROTACs **6**–**8**). The most profound degradation of VHL30 (71% protein degradation, as quantified by western blot) was observed for hetero-PROTAC **3**, meaning that both ligases were degraded simultaneously at 0.1 μM concentration. The effect on VHL30 levels was very similar at 1 μM (Figure S5). Bidirectional degradation was in stark contrast to the properties of our CRBN-VHL hetero-PROTAC CRBN-6-5-5-VHL, which selectively degraded CRBN.^20,50^ Similarly, the VHL-targeting homo-PROTAC CM11 only caused a reduction of the long VHL isoform but spared the 19 kDa protein.^17^ Comparative analysis between **1**, **2**, and CM11 confirmed similarities between PROTACs whereby IAP-VHL hetero-PROTAC **1** also notably and dose dependently reduced VHL19 levels (42% remaining VHL19 at 1 μM, Figure S6). We estimate that the effects of IAP-VHL degraders would not be masked by the hypoxia-inducible factor (HIF)-dependent hypoxic response because recent results showed that homo-PROTAC-mediated VHL30 degradation or siRNA-mediated knockdown of VHL leads to almost undetectable stabilization of HIF-1α.^17,51^

**Figure 4:**
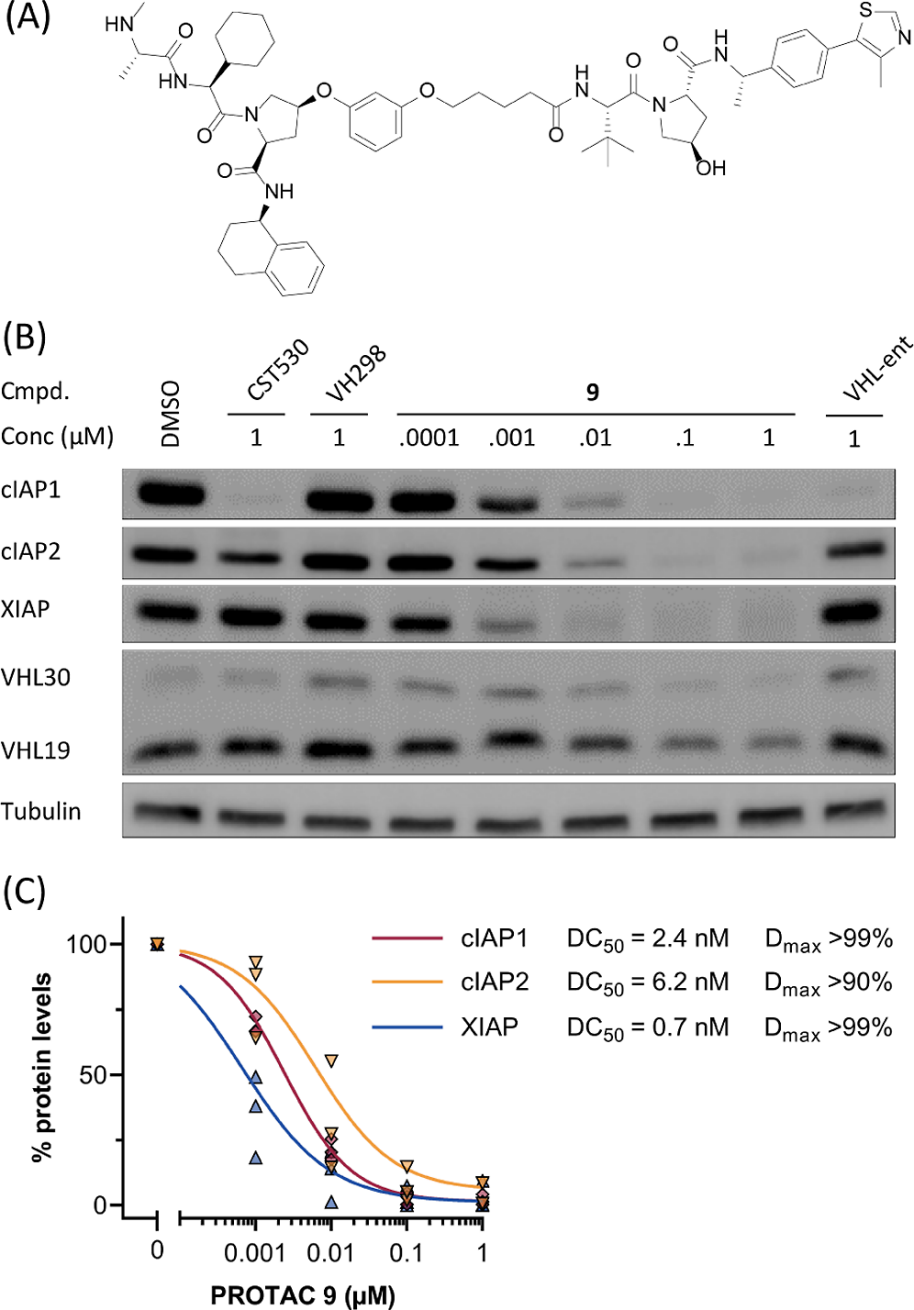
(A) Structure of pan-IAP degrader PROTAC **9**. (B) IAP-VHL hetero-PROTAC **9** (CST626) induces cIAP1, cIAP2, XIAP, and VHL30 degradation in a dose-dependent manner. Hetero-PROTAC **11** (impaired VHL binding) and the monovalent IAP inhibitor CST530 induce only cIAP1 degradation. VHL inhibitor VH298 has no effects on the investigated proteins. MM.1S cells were treated with PROTACs or controls at indicated concentrations for 16 h. (C) Quantification of (B) and calculation of the DC_50_ values from repeats (*n* = 4).

After the initial PROTAC screening, **1** was selected for further optimization due to its comparatively small size and thus the higher chance to overcome PK/PD penalties.^52^ We modified the compound by incorporating the Me-VHL ligand **69** with improved binding affinity for VHL into the hetero-PROTAC. The resulting compound **9** (Figure 4A) showed enhanced pan-IAP degradation in MM.1S cells at even lower concentrations (Figure 4B). Interestingly, also stronger VHL19 degradation was observed at 1 μM (24% remaining VHL19). Densitometric quantifications of western blotting bands after treatment with hetero-PROTAC **9** in MM.1S cells revealed DC_50,16h_ values of 2.4 nM (cIAP1), 6.2 nM (cIAP2), and 0.7 nM (XIAP). Maximum cIAP1, cIAP2, and XIAP degradation (Dmax) of 99, 90, and 99%, respectively, at 0.1 μM concentration of **9** was achieved (Figure 4C). A head-to-head comparison of **9** with birinapant demonstrated that the latter caused more pronounced cIAP1 degradation, whereas **9** outperformed birinapant in depleting cIAP2 and XIAP (Figure S7A). On the other hand, AZD5582 showed stronger cIAPs degradation than IAP-VHL hetero-PROTAC **9** but did not influence XIAP levels even at 1 μM (Figure S7B).

Profiling the activities of the second series of hetero-PROTACs, where we utilized a different linker exit vector, revealed a degradation profile similar to that of the first series. Namely, hetero-PROTACs **12–14** with C5, C8, and C4–O–C4 linkers, respectively, induced the most potent pan-IAP degradation (Figures 2 and S3). Unidirectional ubiquitination between the two E3 ligases was again observed only for hetero-PROTACs with long linkers (compounds **17–19**). In line with this, also no effect on VHL19 levels was seen (Figures 2 and S3). In terms of achieving degradation of a pair of IAPs, **19** seemed interesting as it caused dual cIAP1/XIAP degradation at 0.1 μM concentration in MM.1S. However, profiling of the concentration dependence of **19** showed pan-IAP degradation at 1 μM and no degradation at 10 nM (Figure S8).

To investigate the relative ability of E3 ligases to induce degradation of each other upon treatment with the IAP-CRBN hetero-PROTACs, MM.1S cells were used. For **21–26**, consistent, unidirectional, and distinct degradation of all three IAPs was observed already at 0.1 μM concentration (Figure 2). Of these, compounds **22**, **25**, and **26** showed pronounced pan-IAP depletion, and, concurrently, they induced substantial IKZF3 degradation at 1 μM as an effect of modulating the substrate scope upon pomalidomide binding (Figure S4B). At 0.1 μM, only **22** caused depletion of IKZF3, with approximately 40% of IKZF3 degraded after 16 h treatment. This dual mode could be useful in settings where these secondary effects are desirable. The most intriguing finding within the IAP-CRBN hetero-PROTAC series was observed for compound **27**, equipped with the longest linker. An isoform-selective XIAP degradation was indicated after 16 h-treatment at 0.1 μM (Figures 2 and S4A). A significant and selective decrease of XIAP levels compared to cIAP1 and cIAP2 was confirmed on a proteome level, where MM.1S cells were treated with hetero-PROTAC **27** for 3 h (see Figure 6B). This result unveils that IAP selectivity within the IAP-CRBN hetero-PROTAC series can be tuned by linker modifications.

**Figure 5:**
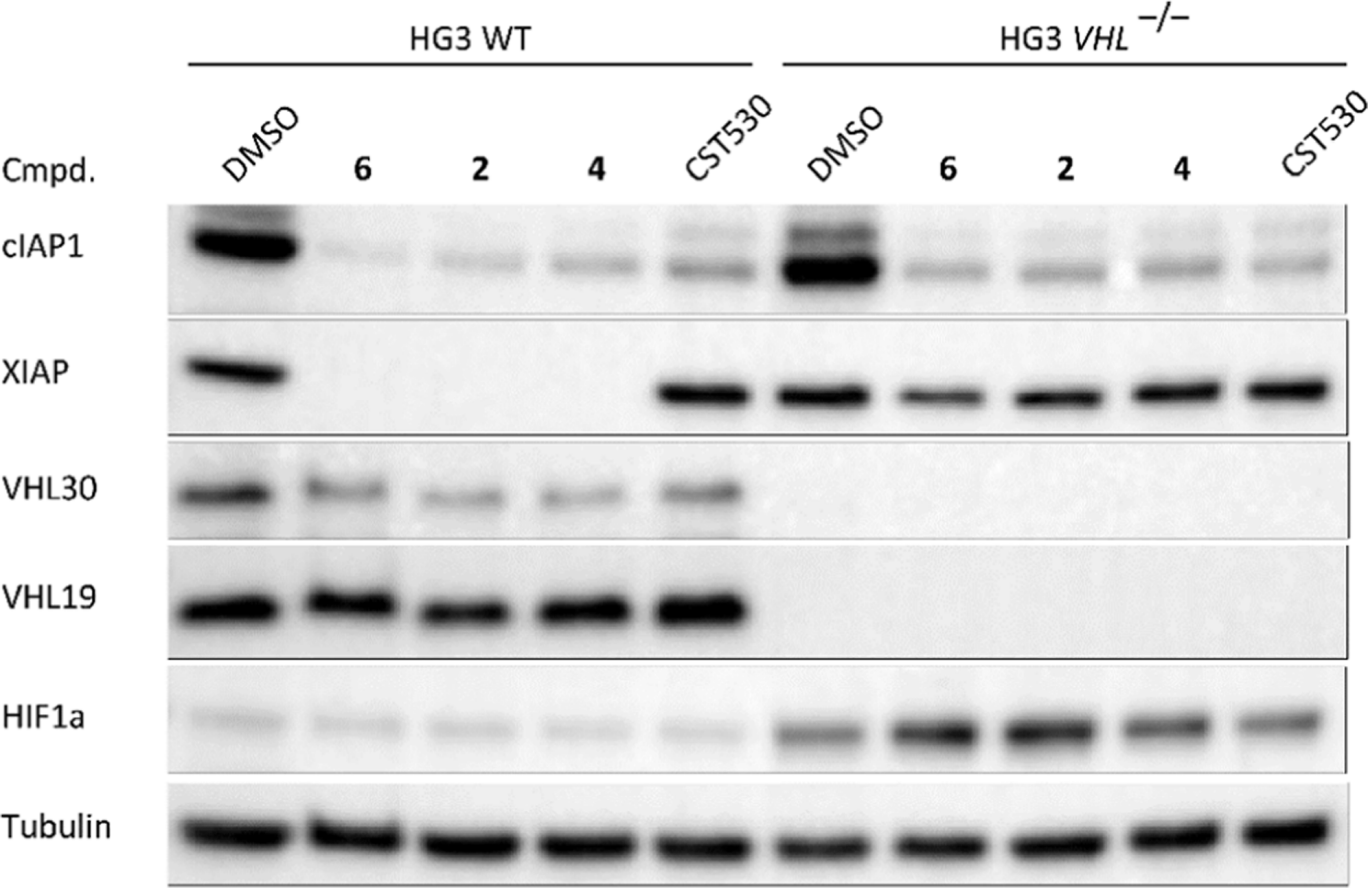
PROTACs **6**, **2**, and **4** retain degradation of cIAP1 and XIAP in chronic lymphocytic leukemia wild-type cells (HG3) and HG3 VHL-knockout cells. Cells were treated with compounds at 1 μM for 16 h.

**Figure 6:**
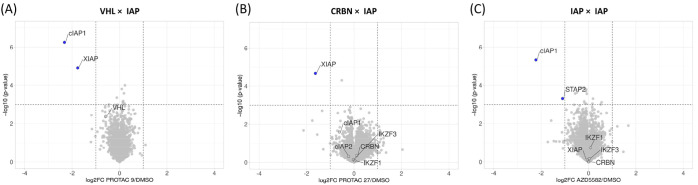
diaPASEF quantitative proteomics for (A) hetero-PROTAC **9** (CST626), (B) hetero-PROTAC **27** (SAB142), and (C) homobivalent compound AZD5582. MM.1S cells were treated with either DMSO or the mentioned compounds at 0.1 μM for 3 h in four and two biological replicates, respectively. Downstream statistical analysis was performed using Bioconductor’s limma package. The quantified proteins were plotted as log 2-fold change (compound/DMSO) versus −log 10 of *p*-value using RStudio. Note: dataset for **27** was obtained in an independent/separate proteomics run.

### Mechanistic Considerations

To understand the mechanism of E3 recruitment and ubiquitin transfer, we tested a set of control compounds with inactivated IAP- or VHL-binding motifs (Table S4). As little was known about appropriately rendering IAP ligands inoperative, we synthesized a series of putative IAP-non-binding controls **10a–d** (Scheme 2A and Figure S9). In **10a**, the stereochemistry of the critical *N*-methyl alanine portion was inverted or substituted with a second methyl group (**10b**). However, literature data indicated remaining affinity for the XIAP-BIR3 domain.^53^ Indeed, **10a** and **10b** were still able to induce pan-IAP or cIAP1 and cIAP2 degradation, respectively (Figure S9). Further increasing the size of the *N*-terminal substituent and lowering the basicity in **10c** (R = acetyl) and **10d** (R = Boc) led to inactivated PROTACs. Both methylation and acetylation were performed through a convenient late-stage modification reactions of the final PROTAC **9**, highlighting the general utility of these transformations to produce inactivated IAP-recruiting PROTACs. By analogy with series **10**, hetero-PROTAC **11** (VHL-*ent*) possessing a VHL non-binding diastereomer only induced cIAP1 degradation (Figure 4B), which is a common attribute of IAP antagonists.

Next, cellular activities of hetero-PROTACs **2**, **4**, and **6** were evaluated in chronic lymphocytic leukemia cells (HG3), for which a VHL-knockout cell line was created (Figure 5). In both VHL^+/+^ and VHL^–/–^ cells, cIAP1 autodegradation was observed after treatment with our hetero-PROTACs, demonstrating that ligand binding is the conditio per quam. In contrast, recruitment of a VHL is required for XIAP degradation as this occurred only in HG3 wild-type cells. Thus, VHL knockout confirmed the involvement of E3 ubiquitin ligase CRL2^VHL^ in the induced degradation of XIAP (and, at least in part, cIAP1) by these IAP-VHL heterobifunctional PROTACs. This provides additional evidence that the degradation of IAPs relies on the formation of a hetero–ternary complex consisting of both ligases and the degraders.

When evaluating the time dependence of hetero-PROTAC **9**, we observed complete degradation of cIAP1, cIAP2, and XIAP already after 3 h at 0.1 μM compound concentration. Interestingly, the effect on VHL30 depletion was most pronounced after 6 h of treatment in MM.1S cells (Figure S10A).

Next, we examined the persistence of IAP degradation in MM.1S cells after a single exposure to 1 μM of **9** and subsequent removal of the compound. Results indicated a nearly full and stable pan-degradation of IAPs up to 72 h. VHL19 and VHL30 levels restored more rapidly following drug washout (Figure S11A). Nevertheless, intracellularly cycling quantities of PROTAC that remain inside the cells after washout may be sufficient to generate these characteristics. In contrast, when the system was further challenged with the competing VH298 after the washout, XIAP levels increased more rapidly (Figure S11B), consistent with an IAP antagonist mode and an unleashed resynthesis of XIAP. In a series of experiments where individual IAPs were knocked out, we observed no differences in VHL30 degradation by hetero-PROTAC **9**; e.g., in cIAP1 knockout cells, VHL30 degradation could also be mediated by cIAP2 (Figure S12). A set of experiments were performed to demonstrate the involvement of the ubiquitin–proteasome system in degradation. Treatment of cells with a proteasome inhibitor MG132 completely abrogated degradation of IAPs. The reliance on CRL2^VHL^ was assessed with a neddylation inhibitor MLN4924, which blocks the activity of CRLs (Figure S13A). Similarly, a selective ubiquitin-activating enzyme inhibitor MLN7243 also prevented the PROTAC-induced degradation of IAPs (Figure S13A).

Concentration- and time-dependent degradation of IAPs in MM.1S cells was evaluated for **25** too (Figures S14 and S10B). Complete cIAP1 and XIAP depletion occurred already at 10 nM, whereas 0.1 μM concentration was needed for the complete depletion of all IAPs. The corresponding CRBN-non-binding control **28** failed to degrade XIAP at 1 μM concentration but caused a significant deficit of cIAP2 (32% remaining protein, Figure S14). Pan-depletion of IAPs by **25** was also very rapid as we observed complete degradation already after 3 h at 0.1 μM (Figure S10B). In addition, the proteasome-mediated mechanism of IAP degradation by **25** was confirmed using the same experiments as for hetero-PROTAC **9** (Figure S13B).

To analyze the proteome-wide degradation selectivity of hetero-PROTACs **9**, **25**, and **27**, a diaPASEF-based mass spectrometry approach was employed.^54^ MM.1S cells were treated with 100 nM PROTACs for 3 h. Of the total 7170 unique proteins identified, **9** (Figure 6A) and **25** (Figure S15) degraded cIAP1 and XIAP to levels below the detection level, whereas cIAP2 could not be evaluated in this experiment as it was undetected in DMSO–vehicle treatments. Accordingly, global proteomic plots show the mathematically imputed levels of IAP proteins in treatment groups if the corresponding IAP was detected in the control treatment (see also Experimental Section). AZD5582 also depleted cIAP1 below the detection level but did not cause XIAP degradation (Figure 6C), which is in accordance with the fact that IAP antagonists have no effect on XIAP.

In a separate experiment, global proteome analysis in MM.1S cells after treatment with **27** (Figure 6B) showed selective XIAP degradation with no impact on cIAP1 and cIAP2, rendering this compound significantly more selective for XIAP over cIAP1 and cIAP2 that were only degraded at higher concentrations and after prolonged treatment times. Moreover, we did not observe changes in CRBN, IKZF1, IKZF3, and VHL levels in the proteomic data, thus further substantiating the unilateral effect of our hetero-PROTACs.

### pan-IAP Degradation Reduces Cell Viability

**Figure 7:**
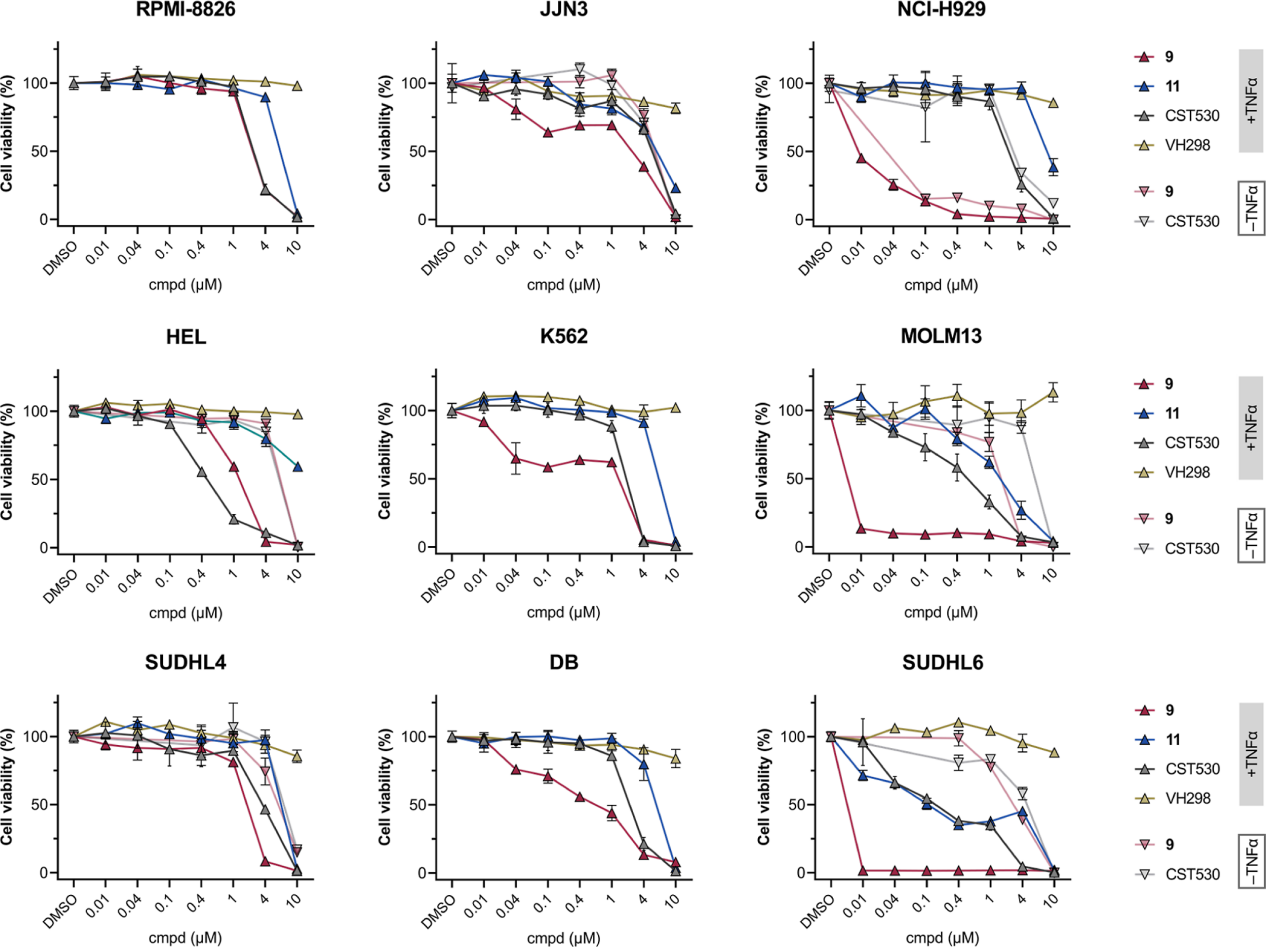
Cell viability screenings in nine different hematological cancer cell lines with pan-IAP degrader **9**, its VHL non-binding control **11**, as well as the structurally related IAP antagonist CST530 and the VHL inhibitor VH298 as respective controls. In certain cases, viability inhibition was assessed in the presence and absence of TNF-α. Multiple myeloma, acute myeloid leukemia, and lymphoma cell lines were treated with the respective compounds at indicated concentrations for 96 h. Viability is normalized to their respective DMSO controls. Data represent means ± s.d. of at least three independent biological replicates.

To assess the pharmacological consequences of IAP depletion, the pan-IAP degraders **9** and **25**, along with appropriate inactivated PROTACs, were evaluated for their cell growth inhibition in nine hematological cell lines (Figures 7 and S17), i.e., three myeloma (RPMI-8226, JJN3, and NCI-H929), three leukemia (HEL, K562, and MOLM13), and three lymphoma cells (SUDHL4, DB, and SUDHL6). We included the monovalent ligase ligands VH298 (VHL), pomalidomide (CRBN), and CST530 (IAP), as well as the homobivalent SMAC mimetic AZD5582 as reference standards. As TNF-α and related signaling cascades represent crucial factors for the single-agent activity of IAP-targeting compounds,^29,38,39,55^ the viability inhibition of sensitive cell lines was evaluated in the presence and absence of this inflammatory stimulus. Co-administration of TNF-α potentiated the inhibitory effects of both SMAC mimetics and PROTACs for all cell lines tested, consistent with previous studies.^56−59^ pan-IAP degraders **9** and **25** were more potent than the positive control IAP monovalent and bivalent antagonists in several cell lines (Figures 7 and S17). PROTACs **9** and **25** demonstrated superior activity over AZD5582 in NCI-H929, reaching IC_50_ values of 8.5 and 27 nM, respectively (Tables 1 and S8). In addition, PROTAC **9** demonstrated a competitive IC_50_ profile in MOLM13 cells at 2.1 nM and SUDHL6 cells at 1.6 nM. While the activity of PROTAC **25** in these two cell lines did not supersede PROTAC **9**, it was able to induce potent cell viability reduction in other cell lines such as JJN3 and SUDHL4 (Table S8), surpassing that of IAP antagonists. These effects were independent of IAP baseline levels (Figure S16), which is in agreement with the previous studies of IAP antagonists.

**Table 1:** Cell Viability Profiles (IC_50_ Values) of the VHL-Recruiting PROTAC **9**, the VHL Non-Binding Control **11**, as Well as the Monovalent IAP Antagonist CST530 and the VHL Inhibitor VH298 for Comparison^a^

		IC_50_ (μM)			
cell line	disease^b^	**9**	**11**	CST530	VH298
**RPMI-8826**	MM	2.54	>5	2.79	>10
**JJN3**	MM	1.14	>5	4.73	>10
**NCI-H929**	MM	0.0085	>5	2.34	>10
**HEL**	AML	1.17	>10	0.45	>10
**K562**	AML	0.42	>5	1.71	>10
**MOLM13**	AML	0.0021	>1	0.42	>10
**SUDHL4**	DLBCL	1.69	>5	3.44	>10
**DB**	DLBCL	0.46	>5	2.21	>10
**SUDHL6**	DLBCL	0.0016	0.17	0.17	>10

a Values correspond to TNFα-challenged conditions.

b MM, multiple myeloma; AML, acute myeloid leukemia; DLBCL, diffuse large B-cell lymphoma.

## Conclusions

In this study, we designed heterobifunctional compounds assembled from an IAP antagonist linked to either a VHL- or a CRBN-recruiting ligand. The entire set of PROTACs consisted of 32 tailored members, which were subjected to in-depth biological studies. Through appropriate control experiments (chemical controls and impairment of the ubiquitin–proteasome system), we provided significant evidence for the engagement with the proposed E3 ligases and PROTAC-induced ubiquitin transfer. The accompanied heterodimerization approach led to novel E3 modulators with IAP degradation profiles that could not be reached with monomeric or homobivalent SMAC mimetics. Among the set of IAP degraders were compounds that induced depletion of the 19 and 30 kDa VHL isoforms. The described pan-IAP degraders will serve as selective tools to explore the biology of IAPs and thus open up new avenues for apoptosis research in various cellular contexts. In addition, selected compounds from our series warrant further appraisal as anti-cancer agents on account of their ability of depleting validated cancer-related IAPs. Preliminary cell-based evaluations of our lead hetero-PROTAC **9** demonstrated that induced degradation of IAPs supersedes the biological effects of monovalent and bivalent IAP antagonists in certain cases. Therefore, further development of IAP-targeting heterobifunctional compounds may lead to degraders with significant therapeutic benefits in the battle against cancer. Exploiting PROTAC methodology to induce the degradation of therapeutically relevant ligases raises hope to unlock this difficult-to-tackle class of drug targets. We also anticipate that the contest between two different E3s may be extendable to any other ligandable ligase.

## Experimental Section

### Chemistry General Remarks

Preparative column chromatography was performed using Merck silica gel 60 (0.063–0.200 mm) or using an automated flash chromatography system puriFlash XS 520Plus. Melting points were determined on a Büchi 510 oil bath apparatus or on a Reichelt hot-stage apparatus and were uncorrected. ^1^H NMR and ^13^C NMR spectra were recorded on a Bruker Avance 400 MHz NMR spectrometer, a Bruker Avance 500 MHz NMR spectrometer, or a Bruker Avance III 600 MHz NMR spectrometer, respectively. NMR spectra were processed and analyzed in MestReNova. Chemical shifts are given in parts per million (ppm) and are referenced to the deuterated solvent used. Coupling constants *J* are given in Hz, and the splitting patterns are given as s (singlet), d (doublet), t (triplet), q (quartet), or m (multiplet). In the case of overlapping extraneous solvent peaks, multiplet analyses in ^1^H NMR spectra were performed using qGSD (quantitative Global Spectral Deconvolution). Resonance assignments were made on the basis of one- and two-dimensional NMR techniques which include ^1^H, ^13^C, DEPT, HSQC, and HMBC experiments. *Important note*: the presence of amide rotamers significantly complicated the appearance and validation of the ^1^H and ^13^C NMR spectra associated with synthetic intermediates and final PROTACs. The presence of rotamers was demonstrated by recording a representative ^1^H NMR at 80 °C (see the Supporting Information). Thus, reported resonances and integrals may have limited accuracy. High-resolution mass measurements were recorded on a Thermo Scientific Q Exactive Plus mass spectrometer (Thermo Fisher Scientific). The purity and identity of compounds were determined on an Infinity Lab LC/MSD system (Agilent) with the ESI source coupled with an HPLC 1260 Infinity II (Agilent) using an EC50/2 Nucleodur C18 Gravity 3 μm column (Macherey-Nagel). The column temperature was 40 °C. HPLC conditions started with 90% H_2_O containing 2 mM NH_4_Ac. The gradient ramped up to 100% MeCN in 10 min, followed by further flushing with 100% MeCN for 5 min. The flow rate was 0.5 mL/min. The samples were dissolved in H_2_O, MeOH, or MeCN (approx. 1 mg/mL), and 2 μL of the sample solution was injected. Positive total ion scans were observed from 100 to 1000 *m*/*z* (or more if necessary), and UV absorption was detected from 190 to 600 nm using a diode array detector (DAD). The purity was determined at 220–600 nm. Analytical reversed-phase HPLC for PROTACs **11** and **20–27** was performed on a Thermo Scientific Dionex UltiMate 3000 UHPLC modular system (Thermo Fisher Scientific), equipped with a photodiode array detector set to 254 nm. A Waters Acquity UPLC HSS C18 SB column (1.8 μm, 2.1 mm × 50 mm) was used and thermostated at 40 °C. The mobile phase consisted of 0.1% TFA in H_2_O (A) and MeCN (B), employing the following gradient: 95% A to 5% A in 10 min, then 95% B for 4 min, with a flow rate of 0.3 mL/min, and an injection volume of 5 μL. All compounds that were evaluated in biological assays are >95% pure by HPLC analysis.

Note: To provide readers a clearer picture of all synthesized compounds and to enable easier tracking of experimental procedures, a table with structures of all intermediates is given at the end of the Supporting Information.

### General Procedures

#### General Procedure I: Mesylation

To a solution of the corresponding alcohol (7 mmol) in dry CH_2_Cl_2_ (15 mL), DIPEA (1.36 g, 1.79 mL, 10.5 mmol) was added under an argon atmosphere and the mixture was cooled to 0 °C. Subsequently, methanesulfonyl chloride (1.20 g, 0.81 mL, 10.5 mmol) was added dropwise at 0 °C, followed by stirring of the mixture at rt for 2 h. After the reaction was complete (monitored by TLC), MeOH (20 mL) was added to the mixture carefully. The volatiles were then evaporated, and the crude product was purified by column chromatography.

#### General Procedure II: Alkylation of the IAP Ligand Using Cl-Bearing Linkers or Conjugates

The corresponding linker or the VHL ligand–linker conjugate (0.30 mmol) was dissolved in dry acetone (15 mL), and NaI (0.45 g, 3.0 mmol) was added. The mixture was stirred at 60 °C for 48 h. After evaporation of the solvent, the residue was suspended in EtOAc (50 mL) and subsequently washed with 10% Na_2_SO_3_ solution, H_2_O, and brine (each 25 mL). The organic layer was dried over Na_2_SO_4_, filtered, and evaporated. This intermediate was dissolved in dry DMF (5 mL), and Cs_2_CO_3_ and the corresponding IAP ligand **65** or **66** (1.0 equiv based on the yield from the Finkelstein reaction) were added. The mixture was stirred at 60 °C for 18 h. After cooling, it was quenched with half-saturated brine (100 mL) and extracted with CH_2_Cl_2_ (3 × 50 mL). The combined organic layers were washed with 5% LiCl solution and brine (each 50 mL), dried over Na_2_SO_4_, filtered, and evaporated. The crude product was purified by column or flash chromatography.

#### General Procedure III: Coupling of the VHL1 Ligand and Cl-to-COOH Linkers

The corresponding Cl-to-COOH linker **L1a–L8a** (0.50 mmol) was dissolved in dry DMF (5 mL), and DIPEA (0.35 mL, 2 mmol) was added, followed by the addition of HATU (0.21 g, 0.55 mmol). After stirring for 5 min, the corresponding VHL ligand **68–70** (deprotected amine, 0.62 mmol) dissolved in dry DMF (5 mL) and DIPEA (0.35 mL, 2 mmol) were added to the mixture. The combined mixture was stirred at room temperature for 16 h, after which half-saturated brine (50 mL) was added, and the product was extracted with EtOAc (3 × 50 mL). The combined organic phases were washed with saturated NH_4_Cl solution, 5% LiCl solution, and brine (each 50 mL), dried over Na_2_SO_4_, filtered, and concentrated in vacuo.

#### General Procedure IV: Alkylation of the VHL2 Ligand Using OMs-to-Cl Linkers

The corresponding OMs-to-Cl linker **L1b–L8b** (1.2 mmol) was dissolved in dry DMF (10 mL), followed by the addition of Cs_2_CO_3_ (0.49 g, 1.5 mmol). Then, the phenolic VHL ligand **71** (0.55 g, 1.0 mmol) dissolved in dry DMF (5 mL) was added. The combined mixture was stirred at room temperature for 16 h and 3 h at 60 °C. After cooling, half-saturated brine (50 mL) was added, and the product was extracted with EtOAc (3 × 50 mL). The combined organic phases were washed with saturated NH_4_Cl solution, 5% LiCl solution, and brine (each 50 mL); dried over Na_2_SO_4_, filtered, and concentrated in vacuo. The crude product was purified by column or flash chromatography.

#### General Procedure V: Alkylation of the IAP Ligand Using OMs-to-Cl Linkers

To a solution of IAP ligand **65** (0.17 g, 0.25 mmol) and K_2_CO_3_ (52 mg, 0.38 mmol) in dry DMF (2 mL), a solution of the corresponding mesylate-bearing linker **L1b–L8b** (0.30 mmol) in dry DMF (2 mL) was added under an argon atmosphere. The mixture was stirred at 70 °C for 20 h. The volatiles were then evaporated, and the crude product was purified by column chromatography.

#### General Procedure VI: Synthesis of Alkyl Azides and Subsequent Reduction to Amines

To a solution of the corresponding IAP ligand–linker–chloro conjugate **91–98** (0.19 mmol) in dry DMF (5 mL), NaN_3_ (25 mg, 0.38 mmol) was added under an argon atmosphere. After stirring the mixture at 80 °C for 4 h, the volatiles were removed, and H_2_O (40 mL) was added. The product was extracted with EtOAc (60 mL). The organic layer was washed with brine (50 mL), dried over Na_2_SO_4_, filtered, concentrated, and further dried under high vacuum. This azide intermediate was dissolved in dry MeOH (5 mL) and treated with 10% Pd/C (22 mg, 20% w/w). The reaction mixture was stirred under H_2_ (1 atm, balloon) for 2 h. The mixture was filtered through Celite and washed with MeOH, and the filtrate was concentrated. The products were used in the next step without further purification.

#### General Procedure VII: Removal of Boc Protecting Groups

The Boc-protected PROTAC precursor was treated with 1 M HCl in EtOAc (5 mL), and the mixture was stirred at rt for 4 h. After removal of the volatiles, the oily residue was treated with Et_2_O (5 mL), and the mixture was stirred at rt for 1 h. If a colorless precipitate appeared, it was collected by suction filtration and washed with Et_2_O (2 × 2 mL). Because of sufficient purity, PROTACs **2**, **4**, **6**, **9**, **10**, and **12–17** were used as hydrochloride salts. For the remaining final PROTACs, additional purification by column chromatography was necessary, and those compounds were transformed into free bases.

#### General Procedure VIII: Nucleophilic Aromatic Substitution

The corresponding IAP ligand–linker–amine conjugate **99–106** (0.11 mmol) was dissolved in dry DMSO (2 mL), and DIPEA (44 mg, 56 μL, 0.33 mmol) and the corresponding CRBN ligand **72** or **73** (32 mg, 0.11 mmol) were added. The mixture was stirred at 90 °C for 20 h. After cooling, H_2_O (30 mL) and saturated NaHCO_3_ solution (10 mL) were added, and the mixture was extracted with CH_2_Cl_2_ (5 × 50 mL). The combined organic layers were washed with brine (200 mL), dried over Na_2_SO_4_, filtered, and concentrated. The oily residue was purified by column chromatography.

### Syntheses of Linkers

#### Syntheses of **L1a–L8a** Linkers (**L1a**: Cl-to-CO_2_Bn and **L2a–L8a**: Cl-to-CO_2_H)

##### **L1a**: Benzyl 5-Chloropentanoate (**30**)

5-Chlorovaleric acid (1.85 g, 13.55 mmol), benzyl bromide (2.32 g, 1.61 mL, 13.55 mmol), and Na_2_CO_3_ (1.72 g, 16.26 mmol) were mixed in MeCN (20 mL) and heated to 80 °C for 18 h. After cooling, the suspension was filtered and then partitioned between H_2_O (100 mL) and EtOAc (2 × 100 mL). The combined organic layers were washed with H_2_O, NaHCO_3_ solution, and brine (each 50 mL); dried over Na_2_SO_4_; filtered; and concentrated in vacuo. The crude product was purified by column chromatography (petroleum ether/EtOAc 19:1) to give a colorless oil. Yield (1.87 g, 61%); *R*_f_ = 0.43 (petroleum ether/EtOAc 19:1); ^1^H NMR (600 MHz, DMSO-*d*_6_): δ 1.61–1.76 (m, 4H), 2.39 (t, *J* = 7.3 Hz, 2H), 3.62 (t, *J* = 6.4 Hz, 2H), 5.08 (s, 2H), 7.29–7.39 (m, 5H); ^13^C NMR (151 MHz, DMSO-*d*_6_): δ 22.00, 31.46, 32.76, 40.24, 45.08, 65.54, 128.09, 128.14, 128.58, 136.39, 172.67; LC–MS (ESI) *m*/*z*: [M + H]^+^ calcd for C_12_H_16_ClO_2_, 227.08; found, 227.1.

##### **L2a**: 8-Chlorooctanoic Acid (**31**)

This compound was synthesized as we described previously.^60^

##### 4-(4-Chlorobutoxy)butan-1-ol (**32**)

1,4-Butanediol (33.80 g, 375 mmol) was mixed in DMSO (50 mL) and aqueous NaOH (50%, 19.3 mL, 375 mmol). After stirring for 10 min, 1-bromo-4-chlorobutane (12.86 g, 75 mmol) was added while cooling with a water bath. The resulting suspension was vigorously stirred at rt for 24 h. After the addition of a saturated NH_4_Cl solution (150 mL), the mixture was extracted with CH_2_Cl_2_ (3 × 150 mL). The combined organic layers were washed with H_2_O (150 mL) and brine (150 mL), dried over Na_2_SO_4_, filtered, and concentrated. The crude product was purified by column chromatography (gradient of petroleum ether/EtOAc 2:1 to 3:2) to give a colorless oil. Yield (4.88 g, 36%); *R*_f_ = 0.31 (petroleum ether/EtOAc 1:1); ^1^H NMR (500 MHz, DMSO-*d*_6_): δ 1.38–1.54 (m, 4H), 1.54–1.63 (m, 2H), 1.70–1.79 (m, 2H), 3.30–3.43 (m, 6H), 3.63 (t, *J* = 6.7 Hz, 2H), 4.32 (t, *J* = 5.2 Hz, 1H); ^13^C NMR (126 MHz, DMSO-*d*_6_): δ 26.04, 26.76, 29.34, 29.42, 45.44, 60.71, 69.23, 70.08; LC–MS (ESI) *m*/*z*: [M + H]^+^ calcd for C_8_H_18_ClO_2_, 181.10; found, 180.9.

##### **L3a**: 4-(4-Chlorobutoxy)butanoic Acid (**33**)

Alcohol **32** (1.08 g, 6 mmol) was dissolved in MeCN (15 mL) and H_2_O (15 mL). TEMPO (0.20 g, 1.32 mmol) was then added, followed by the portionwise addition of (diacetoxyiodo)benzene (4.25 g, 13.2 mmol). The orange mixture was stirred at rt for 16 h. It was neutralized by the addition of saturated NaHCO_3_ solution (100 mL), and the aqueous layer was washed with EtOAc (2 × 100 mL). The aqueous phase was then acidified by the careful addition of 2 N HCl solution until pH = 1. The mixture was then extracted with EtOAc (2 × 100 mL), and the combined organic layers were dried over Na_2_SO_4_, filtered, and concentrated. The crude product was purified by column chromatography (gradient of CH_2_Cl_2_ to CH_2_Cl_2_/MeOH 9:1) to give a brownish oil. Yield (0.89 g, 76%); *R*_f_ = 0.45 (CH_2_Cl_2_/MeOH 9:1); ^1^H NMR (500 MHz, DMSO-*d*_6_): δ 1.54–1.63 (m, 2H), 1.66–1.79 (m, 4H), 2.23 (t, *J* = 7.3 Hz, 2H), 3.35 (dt, *J* = 6.3, 8.0 Hz, 4H), 3.63 (t, *J* = 6.6 Hz, 2H), 11.97 (s, 1H); ^13^C NMR (126 MHz, DMSO-*d*_6_): δ 24.88, 26.72, 29.28, 30.56, 45.45, 69.17, 69.26, 69.39, 174.38; LC–MS (ESI) *m*/*z*: [M + H]^+^ calcd for C_8_H_16_ClO_3_, 195.08; found, 195.1.

##### **L4a**: 2-(2-(2-Chloroethoxy)ethoxy)acetic Acid (**34**)

This compound was synthesized as we described previously.^60^

##### **L5a**: 2-(2-(2-(2-Chloroethoxy)ethoxy)ethoxy)acetic Acid (**35**)

This compound was synthesized as we described previously.^60^

##### 6-((6-Chlorohexyl)oxy)hexan-1-ol (**36**)

This compound was synthesized as we described previously.^20^

##### *tert*-Butyl 2-((6-((6-Chlorohexyl)oxy)hexyl)oxy)acetate (**37**)

Linker **36** (1.89 g, 8 mmol), *tert*-butyl bromoacetate (3.08 g, 3.5 mL, 24 mmol), and TBAHS (2.71 g, 8 mmol) were mixed in toluene (6 mL), followed by the addition of 50% aqueous NaOH solution (4 mL) at 0 °C. The mixture was vigorously stirred at rt for 18 h. The mixture was diluted with H_2_O (100 mL) and extracted with EtOAc (3 × 50 mL). The combined organic layers were washed with brine (50 mL), dried over Na_2_SO_4_, filtered, and concentrated. The crude product was purified by column chromatography (petroleum ether/EtOAc 10:1) to give a colorless oil. Yield (1.42 g, 51%); *R*_f_ = 0.48 (petroleum ether/EtOAc 10:1); ^1^H NMR (600 MHz, DMSO-*d*_6_): δ 1.24–1.39 (m, 8H), 1.41 (s, 9H), 1.44–1.51 (m, 6H), 1.66–1.73 (m, 2H), 3.29–3.34 (m, 4H), 3.40 (t, *J* = 6.5 Hz, 2H), 3.60 (t, *J* = 6.6 Hz, 2H), 3.90 (s, 2H); ^13^C NMR (151 MHz, DMSO-*d*_6_): δ 25.15, 25.58, 25.68, 26.24, 27.89, 29.22, 29.27, 29.34, 32.17, 45.47, 68.17, 69.93, 70.03, 70.65, 80.66, 169.63; LC–MS (ESI) *m*/*z*: [M + H]^+^ calcd for C_18_H_36_ClO_4_, 351.23; found, 351.2.

##### **L6a**: 2-((6-((6-Chlorohexyl)oxy)hexyl)oxy)acetic Acid (**38**)

This compound was produced from **37** after acidic cleavage of the *tert*-butyl ester group in CH_2_Cl_2_/TFA (1:1) at 40 °C for 2 h and was used in the next step without further purification after evaporation of the volatiles.

##### *tert*-Butyl 5-((5-((6-Chlorohexyl)oxy)pentyl)oxy)pentanoate (**39**)

This compound was synthesized as we described previously.^20^

##### **L7a**: 5-((5-((6-Chlorohexyl)oxy)pentyl)oxy)pentanoic Acid (**40**)

This compound was produced from **39** after acidic cleavage of the *tert*-butyl ester group in CH_2_Cl_2_/TFA (1:1) at 40 °C for 2 h and was used in the next step without further purification after evaporation of the volatiles.

##### *tert*-Butyl 6-Bromohexanoate (**41**)

This compound was synthesized as we described previously.^20^

##### *tert*-Butyl 6-((6-((6Chlorohexyl)oxy)hexyl)oxy)hexanoate (**42**)

This compound was synthesized as we described previously.^20^

##### **L8a**: 6-((6-((6-Chlorohexyl)oxy)hexyl)oxy)hexanoic Acid (**43**)

This compound was produced from **42** after acidic cleavage of the *tert*-butyl ester group in CH_2_Cl_2_/TFA (1:1) at 40 °C for 2 h and was used in the next step without further purification after evaporation of the volatiles.

#### Syntheses of **L1b–L8b** Linkers (OMs-to-Cl)

##### **L1b**: 5-Chloropentyl Methanesulfonate (**44**)

This compound was prepared using general procedure **I** and 5-chloro-1-pentanol (2.0 g, 16.3 mmol). The crude product was purified by column chromatography (EtOAc) to give a colorless oil. Yield (2.80 g, 85%); *R*_f_ = 0.60 (EtOAc); ^1^H NMR (400 MHz, CDCl_3_): δ 1.52–1.63 (m, 2H), 1.74–1.87 (m, 4H), 3.01 (s, 3H), 3.55 (t, *J* = 6.5 Hz, 2H), 4.24 (t, *J* = 6.4 Hz, 2H); ^13^C NMR (101 MHz, CDCl_3_): δ 22.99, 28.56, 31.97, 37.52, 44.69, 69.72; HRMS (ESI) *m*/*z*: [M + Na]^+^ calcd for C_6_H_13_O_3_ClSNa, 223.0166; found, 223.0164.

##### **L2b**: 8-Chlorooctyl Methanesulfonate (**45**)

This compound was synthesized as we described previously.^60^

##### **L3b**: 4-(4-Chlorobutoxy)butyl Methanesulfonate (**46**)

This compound was prepared using general procedure **I** and **32** (0.41 g, 2.29 mmol). The crude product was purified by column chromatography (EtOAc/*n*-hexanes 1:1) to give a colorless oil. Yield (0.33 g, 56%); *R*_f_ = 0.22 (EtOAc/*n*-hexanes 1:2); ^1^H NMR (400 MHz, CDCl_3_): δ 1.65–1.74 (m, 4H), 1.79–1.89 (m, 4H), 3.00 (s, 3H), 3.43 (td, *J* = 6.2, 2.1 Hz, 4H), 3.56 (t, *J* = 6.6 Hz, 2H), 4.25 (t, *J* = 6.5 Hz, 2H); ^13^C NMR (101 MHz, CDCl_3_): δ 25.83, 26.37, 27.16, 29.63, 37.50, 45.09, 70.00, 70.07, 70.14; HRMS (ESI) *m*/*z*: [M + H]^+^ calcd for C_9_H_20_O_4_ClS, 259.0765; found, 259.0764.

##### **L4b**: 2-(2-(2-Chloroethoxy)ethoxy)ethyl Methanesulfonate (**47**)

This compound was synthesized as we described previously.^60^

##### **L5b**: 2-(2-(2-(2-Chloroethoxy)ethoxy)ethoxy)ethyl Methanesulfonate (**48**)

This compound was synthesized as we described previously.^60^

##### 6-((6-Chlorohexyl)oxy)hexyl Methanesulfonate (**49**)

This compound was prepared using general procedure **I** and **36** (1.05 g, 3.38 mmol). The crude product was purified by column chromatography (EtOAc/*n*-hexanes 1:2) to give a colorless oil. Yield (0.75 g, 57%); *R*_f_ = 0.35 (EtOAc/*n*-hexanes 1:2); ^1^H NMR (400 MHz, CDCl_3_): δ 1.32–1.50 (m, 8H), 1.53–1.62 (m, 4H), 1.71–1.82 (m, 4H), 3.00 (s, 3H), 3.39 (t, *J* = 6.6 Hz, 4H), 3.53 (t, *J* = 6.7 Hz, 2H), 4.22 (t, *J* = 6.6 Hz, 2H); ^13^C NMR (101 MHz, CDCl_3_): δ 25.44, 25.65, 25.82, 26.84, 29.22, 29.67, 29.70, 32.68, 37.50, 45.21, 70.18, 70.76, 70.86; HRMS (ESI) *m*/*z*: [M + H]^+^ calcd for C_17_H_36_O_5_ClS, 387.1967; found, 387.1960.

##### 2-((Tetrahydro-2*H*-pyran-2-yl)oxy)ethan-1-ol (**50**)

To a solution of ethylene glycol (3.00 g, 48.34 mmol) in dry MeCN (25 mL), 3,4-dihydro-2*H*-pyran (4.47 g, 4.82 mL, 53.17 mmol) was added under an argon atmosphere. Subsequently, CuSO_4_ × 5 H_2_O (2.41 g, 9.67 mmol) was added, followed by stirring of the mixture at rt for 3 h. After the reaction was complete, the mixture was filtered, and the filtrate was concentrated. The crude product was purified by column chromatography (EtOAc) to give a colorless oil. Yield (1.21 g, 18%); *R*_f_ = 0.30 (EtOAc); ^1^H NMR (400 MHz, CDCl_3_): δ 1.49–1.60 (m, 4H), 1.73–1.89 (m, 2H), 2.80–2.86 (m, 1H), 3.51–3.58 (m, 1H), 3.66–3.81 (m, 4H), 3.88–3.97 (m, 1H), 4.55–4.59 (m, 1H); ^13^C NMR (101 MHz, CDCl_3_): δ 20.01, 25.27, 30.81, 62.24, 63.26, 70.72, 100.15; HRMS (ESI) *m*/*z*: [M + Na]^+^ calcd for C_7_H_14_O_3_Na, 169.0835; found, 169.0837.

##### 2-(2-((6-((6-Chlorohexyl)oxy)hexyl)oxy)ethoxy)tetrahydro-2*H*-pyran (**51**)

To a solution of **50** (0.72 g, 4.93 mmol) and **49** (1.55 g, 4.93 mmol) in toluene (20 mL), TBAHS (1.67 g, 4.93 mmol) and 50% NaOH (aq) (2.5 mL) were added. The reaction mixture was stirred at rt for 18 h. H_2_O (70 mL) was then added, and the mixture was extracted with EtOAc (3 × 100 mL). The combined organic layers were further washed with H_2_O and brine (each 200 mL). The organic layers were dried over Na_2_SO_4_, filtered, and concentrated in vacuo. The crude product was purified by column chromatography (EtOAc/*n*-hexanes 1:2) to give a colorless oil. Yield (1.08 g, 50%); *R*_f_ = 0.45 (EtOAc/*n*-hexanes 1:2); ^1^H NMR (400 MHz, CDCl_3_): δ 1.30–1.47 (m, 8H), 1.47–1.65 (m, 10H), 1.67–1.87 (m, 4H), 3.37 (td, *J* = 6.6, 2.1 Hz, 4H), 3.41–3.55 (m, 5H), 3.55–3.61 (m, 3H), 3.80–3.90 (m, 2H), 4.62 (t, *J* = 4.3 Hz, 1H); ^13^C NMR (101 MHz, CDCl_3_): δ 19.59, 25.55, 25.64, 26.10, 26.18, 26.83, 29.70, 29.73, 29.84, 30.67, 32.68, 45.18, 62.31, 66.73, 70.08, 70.78, 71.00, 71.44, 99.03; HRMS (ESI) *m*/*z*: [M + H]^+^ calcd for C_19_H_38_O_4_Cl, 365.2453; found, 365.2445.

##### 2-((6-((6-Chlorohexyl)oxy)hexyl)oxy)ethan-1-ol (**52**)

To a solution of **51** (1.05 g, 2.88 mmol) in MeOH (10 mL), *p*TsOH × H_2_O (0.27 g, 1.44 mmol) was added. The reaction mixture was stirred at rt for 20 h. Saturated NaHCO_3_ solution (50 mL) was added, and it was extracted with CH_2_Cl_2_ (3 × 100 mL). The combined organic layers were washed with brine (200 mL). The organic layers were dried over Na_2_SO_4_, filtered, and concentrated in vacuo. The crude product was purified by column chromatography (EtOAc/*n*-hexanes 1:2) to give a colorless oil. Yield (0.70 g, 87%); *R*_f_ = 0.25 (EtOAc/*n*-hexanes 1:2); ^1^H NMR (400 MHz, CDCl_3_): δ 1.31–1.41 (m, 6H), 1.40–1.48 (m, 2H), 1.50–1.64 (m, 6H), 1.71–1.81 (m, 2H), 2.22 (t, *J* = 5.9 Hz, 1H), 3.38 (td, *J* = 6.6, 1.1 Hz, 4H), 3.42–3.48 (m, 2H), 3.48–3.54 (m, 4H), 3.65–3.74 (m, 2H); ^13^C NMR (101 MHz, CDCl_3_): δ 25.61, 26.09, 26.14, 26.81, 29.67, 29.77, 32.65, 45.17, 61.91, 70.77, 70.93, 71.40, 71.85; HRMS (ESI) *m*/*z*: [M + H]^+^ calcd for C_14_H_30_O_3_Cl, 281.1878; found, 281.1873.

##### **L6b:** 2-((6-((6-Chlorohexyl)oxy)hexyl)oxy)ethyl Methanesulfonate (**53**)

This compound was prepared using general procedure **I** and **52** (0.68 g, 2.41 mmol). The crude product was purified by column chromatography (EtOAc/*n*-hexanes 1:2) to give a colorless oil. Yield (0.80 g, 93%); *R*_f_ = 0.18 (EtOAc/*n*-hexanes 1:2); ^1^H NMR (400 MHz, CDCl_3_): δ 1.29–1.48 (m, 8H), 1.50–1.62 (m, 6H), 1.72–1.81 (m, 2H), 3.04 (s, 3H), 3.38 (td, *J* = 6.6, 2.2 Hz, 4H), 3.44–3.49 (m, 2H), 3.52 (t, *J* = 6.7 Hz, 2H), 3.65–3.69 (m, 2H), 4.32–4.37 (m, 2H); ^13^C NMR (101 MHz, CDCl_3_): δ 25.62, 26.02, 26.12, 26.81, 29.60, 29.68, 29.80, 32.66, 37.75, 45.20, 68.54, 69.43, 70.80, 70.91, 71.59; HRMS (ESI) *m*/*z*: [M + H]^+^ calcd for C_15_H_32_O_5_ClS, 359.1654; found, 359.1647.

##### **L7b**: 5-((5-((6-Chlorohexyl)oxy)pentyl)oxy)pentyl Methanesulfonate (**54**)

This compound was synthesized as we described previously.^60^

##### 6-((Tetrahydro-2*H*-pyran-2-yl)oxy)hexan-1-ol (**55**)

To a solution of 1,6-hexanediol (6.00 g, 50.77 mmol) in dry MeCN (35 mL), 3,4-dihydro-2*H*-pyran (4.70 g, 5.08 mL, 55.85 mmol) was added under an argon atmosphere. Subsequently, CuSO_4_ × 5 H_2_O (2.53 g, 10.15 mmol) was added, followed by stirring of the mixture at rt for 3 h. After the reaction was complete, the mixture was filtered, and the filtrate was concentrated. The crude product was purified by column chromatography (EtOAc/*n*-hexanes 1:1) to give a colorless oil. Yield (3.72 g, 36%); *R*_f_ = 0.18 (EtOAc/*n*-hexanes 1:1); ^1^H NMR (400 MHz, CDCl_3_): δ 1.34–1.42 (m, 5H), 1.47–1.64 (m, 8H), 1.67–1.75 (m, 1H), 1.77–1.88 (m, 1H), 3.38 (dt, *J* = 9.5, 6.5 Hz, 1H), 3.45–3.52 (m, 1H), 3.63 (t, *J* = 6.6 Hz, 2H), 3.73 (dt, *J* = 9.6, 6.8 Hz, 1H), 3.82–3.89 (m, 1H), 4.54–4.58 (m, 1H); ^13^C NMR (101 MHz, CDCl_3_): δ 19.86, 25.61, 25.68, 26.16, 29.81, 30.91, 32.83, 62.55, 63.04, 67.65, 99.04; HRMS (ESI) *m*/*z*: [M + H]^+^ calcd for C_11_H_23_O_3_, 203.1642; found, 203.1638.

##### 6-((Tetrahydro-2*H*-pyran-2-yl)oxy)hexyl Methanesulfonate (**56**)

This compound was prepared using general procedure **I** and **55** (3.00 g, 14.83 mmol). The crude product was purified by column chromatography (EtOAc/*n*-hexanes 1:2) to give a colorless oil. Yield (3.81 g, 92%); *R*_f_ = 0.18 (EtOAc/*n*-hexanes 1:2); ^1^H NMR (400 MHz, CDCl_3_): δ 1.38–1.47 (m, 4H), 1.51–1.64 (m, 6H), 1.67–1.84 (m, 4H), 3.00 (s, 3H), 3.38 (dt, *J* = 9.7, 6.4 Hz, 1H), 3.46–3.53 (m, 1H), 3.74 (dt, *J* = 9.6, 6.7 Hz, 1H), 3.82–3.91 (m, 1H), 4.22 (t, *J* = 6.6 Hz, 2H), 4.54–4.58 (m, 1H); ^13^C NMR (101 MHz, CDCl_3_): δ 19.85, 25.42, 25.59, 25.87, 29.19, 29.65, 30.89, 37.48, 62.58, 67.47, 70.18, 99.08; HRMS (ESI) *m*/*z*: [M + H]^+^ calcd for C_12_H_25_O_5_S, 281.1417; found, 281.1413.

##### 2-((6-((6-((6-Chlorohexyl)oxy)hexyl)oxy)hexyl)oxy)tetrahydro-2*H*-pyran (**57**)

To a solution of **36** (2.11 g, 8.92 mmol) and **56** (2.50 g, 8.92 mmol) in toluene (40 mL), TBAHS (3.03 g, 8.92 mmol) and 50% NaOH (aq) (4.5 mL) were added. The reaction mixture was stirred at rt for 18 h. H_2_O (70 mL) was then added, and the mixture was extracted with EtOAc (3 × 100 mL). The combined organic layers were further washed with H_2_O and brine (each 200 mL). The organic layers were dried over Na_2_SO_4_, filtered, and concentrated in vacuo. The crude product was purified by column chromatography (EtOAc/*n*-hexanes 1:2) to give a light yellow oil. Yield (1.80 g, 48%); *R*_f_ = 0.50 (EtOAc/*n*-hexanes 1:2); ^1^H NMR (400 MHz, CDCl_3_): δ 1.31–1.38 (m, 8H), 1.46–1.64 (m, 16H), 1.66–1.83 (m, 4H), 3.35–3.40 (m, 8H), 3.44–3.55 (m, 4H), 3.72 (dt, *J* = 9.6, 6.8 Hz, 1H), 3.85 (ddd, *J* = 11.1, 7.5, 3.2 Hz, 1H), 4.53–4.58 (m, 1H); ^13^C NMR (101 MHz, CDCl_3_): δ 19.82, 25.61, 25.66, 26.20, 26.26, 26.85, 29.72, 29.84, 30.89, 32.69, 45.20, 62.48, 67.70, 70.79, 70.98, 71.02, 98.97; MS (ESI) *m*/*z*: [M + MeOH + H]^+^ calcd for C_24_H_50_O_5_Cl, 454.10; found, 454.1.

##### 6-((6-((6-Chlorohexyl)oxy)hexyl)oxy)hexan-1-ol (**58**)

To a solution of **57** (1.77 g, 4.20 mmol) in MeOH (20 mL), *p*TsOH × H_2_O (0.40 g, 2.10 mmol) was added. The reaction mixture was stirred at rt for 20 h. Saturated NaHCO_3_ solution (50 mL) was added, and it was extracted with CH_2_Cl_2_ (3 × 100 mL). The combined organic layers were washed with brine (200 mL). The organic layers were dried over Na_2_SO_4_, filtered, and concentrated in vacuo. The crude product was purified by column chromatography (EtOAc/*n*-hexanes 1:2) to give a light yellow oil. Yield (1.00 g, 71%); *R*_f_ = 0.20 (EtOAc/*n*-hexanes 1:2); ^1^H NMR (400 MHz, CDCl_3_): δ 1.29–1.47 (m, 12H), 1.50–1.62 (m, 10H), 1.72–1.81 (m, 2H), 3.38 (td, *J* = 6.6, 1.7 Hz, 8H), 3.52 (t, *J* = 6.7 Hz, 2H), 3.62 (t, *J* = 6.6 Hz, 2H); ^13^C NMR (101 MHz, CDCl_3_): δ 25.64, 25.71, 26.12, 26.18, 26.83, 29.69, 29.81, 32.68, 32.82, 45.20, 63.00, 70.79, 70.90, 70.98, 71.02; HRMS (ESI) *m*/*z*: [M + H]^+^ calcd for C_18_H_38_O_3_Cl, 337.2504; found, 337.2497.

##### **L8b**: 6-((6-((6-Chlorohexyl)oxy)hexyl)oxy)hexyl Methanesulfonate (**59**)

This compound was prepared using general procedure **I** and **58** (1.00 g, 2.97 mmol). The crude product was purified by column chromatography (EtOAc/*n*-hexanes 1:2) to give a colorless oil. Yield (0.79 g, 64%); *R*_f_ = 0.40 (EtOAc/*n*-hexanes 1:2); ^1^H NMR (400 MHz, CDCl_3_): δ 1.30–1.47 (m, 12H), 1.51–1.61 (m, 8H), 1.70–1.82 (m, 4H), 2.99 (s, 3H), 3.34–3.41 (m, 8H), 3.53 (t, *J* = 6.7 Hz, 2H), 4.22 (t, *J* = 6.5 Hz, 2H); ^13^C NMR (101 MHz, CDCl_3_): δ 25.44, 25.66, 25.82, 26.20, 26.85, 29.21, 29.68, 29.72, 29.84, 32.69, 37.48, 45.22, 70.19, 70.72, 70.80, 71.02, 71.04; HRMS (ESI) *m*/*z*: [M + H]^+^ calcd for C_19_H_40_O_5_ClS, 415.2280; found, 415.2270.

#### Synthesis of E3 Ligands

##### *tert*-Butyl *N*-[(1*S*)-2-[[(1*S*)-1-Cyclohexyl-2-[(2*S*,4*R*)-4-hydroxy-2-[[(1*R*)-tetralin-1-yl]carbamoyl]pyrrolidin-1-yl]-2-oxo-ethyl]amino]-1-methyl-2-oxo-ethyl]-*N*-methyl-carbamate (**60**)

This compound was synthesized as described previously.^43^ A colorless solid was obtained. Yield (72%); *R*_f_ = 0.18 (petroleum ether/EtOAc 1:2); mp 88–90 °C; ^1^H NMR (600 MHz, DMSO-*d*_6_): δ 0.83–1.29 (m, 8H, CH_2_), 1.39 (s, 9H, CH_3_), 1.52–1.93 (m, 11H), 1.96–2.04 (m, 1H, CH, CH_2_, 2-H, 3-H, 3′-H), 2.65–2.75 (m, 2H, 4-H), 2.73 (s, 3H, CH_3_), 3.49–3.64 (m, 1H), 3.64–3.79 (m, 1H, 5′-H), 4.28–4.45 (m, 3H, CH, 2′-H, 4′-H), 4.45–4.69 (m, 1H, CH), 4.88–4.96 (m, 1H, 1-H), 5.07 (d, *J* = 3.6 Hz, 1H, OH), 7.03–7.20 (m, 3H), 7.29 (d, *J* = 7.6 Hz, 1H, Ar–H), 7.37–7.83 (m, 1H), 8.21 (d, *J* = 8.8 Hz, 1H, CONH); ^13^C NMR (151 MHz, DMSO-*d*_6_): δ 15.13 (CH_3_), 20.58 (C-3), 25.75, 25.93, 26.04, 27.82 (CH_2_), 28.18 (C(CH_3_)_3_), 28.95 (C-4), 29.14 (CH_2_), 30.08 (C-2, CH_3_), 38.01 (CH, C-3′), 46.69 (C-1), 53.38, 54.75 (CH), 55.71 (C-5′), 58.97 (C-2′), 68.98 (C-4′), 79.24 (C(CH_3_)_3_), 125.79, 126.71, 128.41, 128.65 (C-5 to C-8), 137.07, 137.86 (C-4a, C-8a), 155.21, 169.81, 170.51, 171.14 (CO); LC–MS (ESI) 99% purity, *m*/*z*: [M + H]^+^ calcd for C_32_H_49_N_4_O_6_, 585.36; found, 585.2.

##### *tert*-Butyl *N*-[(1*R*)-2-[[(1*S*)-1-Cyclohexyl-2-[(2*S*,4*R*)-4-hydroxy-2-[[(1*R*)-tetralin-1-yl]carbamoyl]pyrrolidin-1-yl]-2-oxo-ethyl]amino]-1-methyl-2-oxo-ethyl]-*N*-methyl-carbamate (**61**)

This compound was synthesized analogously to **60** but using Boc-*N*-Me-d-Ala-OH. A colorless solid was obtained. Yield (61%); *R*_f_ = 0.43 (EtOAc); mp 188–190 °C; ^1^H NMR (600 MHz, DMSO-*d*_6_): δ 0.85–1.21 (m, 5H), 1.24 (d, *J* = 7.2 Hz, 3H), 1.39 (s, 9H), 1.51–2.04 (m, 12H), 2.65–2.73 (m, 2H), 2.73 (s, 3H), 3.59 (dd, *J* = 2.7, 10.4 Hz, 1H), 3.62–3.79 (m, 1H), 4.33–4.43 (m, 3H), 4.57–4.83 (m, 1H) 4.88–4.98 (m, 1H), 5.04 (d, *J* = 3.7 Hz, 1H), 7.03–7.10 (m, 2H), 7.10–7.16 (m, 1H), 7.30 (d, *J* = 7.6 Hz, 1H), 7.73 (d, *J* = 8.9 Hz, 1H), 8.23 (d, *J* = 8.8 Hz, 1H); ^13^C NMR (151 MHz, DMSO-*d*_6_): δ 15.64, 20.58, 25.72, 25.90, 26.08, 27.72, 28.19, 28.94, 29.09, 30.05, 30.10, 37.97, 46.67, 53.55, 54.64, 55.54, 58.95, 68.87, 79.13, 125.75, 126.69, 128.43, 128.62, 137.04, 137.89, 155.12, 169.93, 171.11, 171.53; LC–MS (ESI) 99% purity, *m*/*z*: [M + H]^+^ calcd for C_32_H_49_N_4_O_6_, 585.36; found, 585.5; HRMS (ESI) *m*/*z*: [M + H]^+^ calcd for C_32_H_49_N_4_O_6_, 585.3647; found, 585.3626.

##### 3-Benzyloxyphenol (**62**)

Benzyl bromide (3.42 g, 2.4 mL, 20 mmol), resorcinol (4.40 g, 40 mmol), and K_2_CO_3_ (2.76 g, 20 mmol) in DMF (20 mL) were stirred at 80 °C for 18 h. The brown oil was filtered, rinsed with EtOAc (100 mL), and washed with half-saturated brine (2 × 100 mL) and 5% LiCl solution (100 mL). The organic layer was dried over Na_2_SO_4_, filtered, and concentrated. The material was purified by column chromatography (petroleum ether/EtOAc 19:1) to give a brown oil. Yield (1.92 g, 48%); *R*_f_ = 0.37 (petroleum ether/EtOAc 8:1); ^1^H NMR (500 MHz, DMSO-*d*_6_): δ 5.02 (s, 2H), 6.32–6.37 (m, 1H), 6.38 (t, *J* = 2.3 Hz, 1H), 6.42 (dd, *J* = 2.4, 7.9 Hz, 1H), 7.04 (t, *J* = 8.2 Hz, 1H), 7.27–7.34 (m, 1H), 7.34–7.41 (m, 2H), 7.39–7.47 (m, 2H), 9.35 (br s, 1H); ^13^C NMR (126 MHz, DMSO-*d*_6_): δ 69.16, 102.27, 105.64, 108.19, 127.70, 127.86, 128.53, 129.98, 137.41, 158.70, 159.72; LC–MS (ESI) 99% purity, *m*/*z*: [M + H]^+^ calcd for C_13_H_13_O_2_, 201.09; found, 210.0.

##### *tert*-Butyl *N*-[(1*S*)-2-[[(1*S*)-2-[(2*S*,4*S*)-4-(3-Benzyloxyphenoxy)-2-[[(1*R*)-tetralin-1-yl]carbamoyl]pyrrolidin-1-yl]-1-cyclohexyl-2-oxo-ethyl]amino]-1-methyl-2-oxo-ethyl]-*N*-methyl-carbamate (**63**)

Compound **60** (2.92 g, 5.0 mmol) was dissolved in dry toluene (100 mL), and it was sonicated for 10 min to remove any dissolved gases. Subsequently, the solution was transferred into a round-bottom flask equipped with a large stirring bar, and the colorless solution was purged with argon for 5 min. Then, the other substrates were added in the following order: **62** (1.05 g, 5.25 mmol), PS-TPP (1 mmol/g loading, 5.75 g), and finally DEAD (40% in toluene, 2.40 mL, 5.25 mmol). After purging for another 5 min, the vessel was closed, and the yellow suspension was stirred at rt for 18 h. The resin was filtered off, and it was washed with EtOAc (2 × 100 mL). The combined organic layers were washed with H_2_O and brine (each 250 mL), dried over Na_2_SO_4_, filtered, and concentrated in vacuo. The crude material was purified by column chromatography as follows: the column was packed and started with petroleum ether/EtOAc 2:1. Then, the polarity was gradually increased to 1:2, and it was eluted until the complete elimination of the side product. Subsequently, the polarity was further increased to petroleum ether/EtOAc 1:4 and kept at this level. A colorless solid was obtained. Yield (1.76 g, 46%); *R*_f_ = 0.50 (petroleum ether/EtOAc 1:4); mp 66–68 °C; ^1^H NMR (600 MHz, DMSO-*d*_6_): δ 1.07 (s, 3H), 1.01–1.20 (m, 6H), 1.20–1.31 (m, 1H), 1.31–1.45 (m, 12H), 1.53–1.82 (m, 4H), 2.04–2.11 (m, 1H), 2.44–2.56 (m, 1H), 2.67 (q, *J* = 7.8 Hz, 2H), 2.70 (s, 3H), 3.59 (dd, *J* = 4.3, 11.0 Hz, 1H), 4.24 (dd, *J* = 5.9, 10.8 Hz, 1H), 4.30 (t, *J* = 7.9 Hz, 1H), 4.44 (dd, *J* = 5.2, 9.0 Hz, 1H), 4.53 (s, 1H), 4.87–4.94 (m, 1H), 5.02 (q, *J* = 4.0 Hz, 1H), 5.05 (s, 2H), 6.43–6.51 (m, 1H), 6.54 (t, *J* = 2.4 Hz, 1H), 6.61 (dd, *J* = 2.4, 8.2 Hz, 1H), 7.01–7.15 (m, 4H), 7.17 (t, *J* = 8.2 Hz, 1H), 7.23 (d, *J* = 7.4 Hz, 1H), 7.28–7.34 (m, 1H), 7.34–7.45 (m, 5H), 7.83 (d, *J* = 8.6 Hz, 1H); ^13^C NMR (151 MHz, DMSO-*d*_6_): δ 14.82, 16.97, 17.63, 20.14, 21.24, 24.31, 25.66, 25.83, 25.98, 28.22, 28.47, 28.95, 29.13, 29.88, 30.23, 33.72, 34.61, 40.97, 46.85, 52.18, 55.33, 58.77, 69.46, 75.16, 80.73, 102.85, 107.88, 108.30, 125.94, 126.93, 127.86, 128.04, 128.55, 128.64, 128.87, 130.31, 137.22, 137.44, 158.31, 159.78, 169.55, 170.11, 170.51; LC–MS (ESI) 97% purity, *m*/*z*: [M + H]^+^ calcd for C_45_H_58_N_4_O_7_, 767.43; found, 767.4.

##### *tert*-Butyl *N*-[(1*R*)-2-[[(1*S*)-2-[(2*S*,4*S*)-4-(3-Benzyloxyphenoxy)-2-[[(1*R*)-tetralin-1-yl]carbamoyl]pyrrolidin-1-yl]-1-cyclohexyl-2-oxo-ethyl]amino]-1-methyl-2-oxo-ethyl]-*N*-methyl-carbamate (**64**)

This compound was synthesized analogously to **63** but using precursor **61** (1.75 g, 3.0 mmol) and on a smaller scale (3.0 mmol). A colorless solid was obtained. Yield (1.06 g, 46%); *R*_f_ = 0.50 (petroleum ether/EtOAc 1:1); mp 84–86 °C; ^1^H NMR (600 MHz, DMSO-*d*_6_): δ 0.84–0.95 (m, 2H), 1.01–1.12 (m, 4H), 1.24 (d, *J* = 7.2 Hz, 3H), 1.31–1.37 (m, 10H), 1.52–1.65 (m, 6H), 1.67–1.81 (m, 5H), 2.10 (br s, 1H), 2.63–2.72 (m, 2H), 2.73 (s, 2H), 3.61 (br s, 1H), 4.24–4.40 (m, 2H), 4.44 (dd, *J* = 5.0, 9.0 Hz, 1H), 4.88–4.94 (m, 1H), 4.96–5.05 (m, 1H), 5.06 (s, 2H), 6.45–6.51 (m, 1H), 6.52–6.56 (m, 1H), 6.61 (dd, *J* = 2.4, 8.0 Hz, 1H), 7.01–7.20 (m, 4H), 7.23–7.27 (m, 1H), 7.29–7.34 (m, 1H), 7.33–7.45 (m, 4H), 7.86 (s, 1H), 7.92 (s, 1H); ^13^C NMR (151 MHz, DMSO-*d*_6_): δ 14.56, 16.06, 20.31, 20.54, 21.22, 25.93, 26.06, 26.31, 28.48, 29.20, 29.36, 30.09, 30.51, 35.00, 47.11, 52.55, 53.22, 54.61, 55.29, 58.93, 60.21, 69.72, 75.44, 79.30, 103.10, 108.14, 108.51, 126.18, 127.18, 128.12, 128.28, 128.88, 129.11, 130.55, 137.49, 137.70, 155.50, 158.56, 160.05, 170.24, 170.90, 172.42; LC–MS (ESI) 98% purity, *m*/*z*: [M + H]^+^ calcd for C_45_H_58_N_4_O_7_, 767.43; found, 767.5; HRMS (ESI) *m*/*z*: [M + H]^+^ calcd for C_45_H_59_N_4_O_7_, 767.4378; found, 767.4350.

##### *tert*-Butyl *N*-[(1*S*)-2-[[(1*S*)-1-Cyclohexyl-2-[(2*S*,4*S*)-4-(3-hydroxyphenoxy)-2-[[(1*R*)-tetralin-1-yl]carbamoyl]pyrrolidin-1-yl]-2-oxo-ethyl]amino]-1-methyl-2-oxo-ethyl]-*N*-methyl-carbamate (**65**)

Compound **63** (1.69 g, 2.2 mmol) was dissolved in dry MeOH (20 mL), and 10% Pd/C (0.17 g, 10% w/w) was added. The vessel was closed, evacuated, and refilled with nitrogen gas (3×), followed by hydrogen gas. The black mixture was stirred for 18 h at rt. The charcoal was removed by filtration, and the title compound was obtained as a colorless solid after evaporation. Yield (1.31 g, 81%); *R*_f_ = 0.43 (EtOAc); mp 92–96 °C; ^1^H NMR (600 MHz, DMSO-*d*_6_): δ 0.71–1.24 (m, 9H), 1.38 (s, 9H), 1.52–1.84 (m, 10H), 2.01–2.14 (m, 1H), 2.43–2.53 (m, 1H), 2.64–2.76 (m, 5H), 3.60 (dd, *J* = 4.4, 10.8 Hz, 1H), 4.18–4.27 (m, 1H), 4.32 (t, *J* = 7.8 Hz, 1H), 4.44 (dd, *J* = 5.1, 9.0 Hz, 1H), 4.85–5.00 (m, 2H), 6.23–6.40 (m, 3H), 7.00–7.16 (m, 4H), 7.24 (d, *J* = 7.5 Hz, 1H), 7.84 (d, *J* = 8.5 Hz, 1H), 8.31 (d, *J* = 8.4 Hz, 1H), 9.40 (s, 1H); ^13^C NMR (151 MHz, DMSO-*d*_6_): δ 15.11, 20.04, 25.61, 25.78, 25.92, 28.17, 28.90, 29.07, 29.65, 29.81, 30.16, 34.64, 39.52, 46.78, 52.25, 53.27, 55.23, 58.65, 74.96, 79.17, 103.14, 106.14, 108.55, 125.89, 126.86, 128.51, 128.79, 130.06, 137.17, 137.40, 155.27, 158.29, 158.76, 169.40, 169.97, 170.38; LC–MS (ESI) 99% purity, *m*/*z*: [M + H]^+^ calcd for C_38_H_53_N_4_O_7_, 677.39; found, 677.6.

##### *tert*-Butyl *N*-[(1*R*)-2-[[(1*S*)-1-Cyclohexyl-2-[(2*S*,4*S*)-4-(3-hydroxyphenoxy)-2-[[(1*R*)-tetralin-1-yl]carbamoyl]pyrrolidin-1-yl]-2-oxo-ethyl]amino]-1-methyl-2-oxo-ethyl]-*N*-methyl-carbamate (**66**)

This compound was synthesized analogously to compound **65** but using precursor **64** (1.02 g, 1.33 mmol) in dry MeOH (13 mL) and 10% Pd/C (0.10 g, 10% w/w). A colorless solid was obtained. Yield (0.87 g, 96%); *R*_f_ = 0.57 (EtOAc); mp 92–96 °C; ^1^H NMR (600 MHz, DMSO-*d*_6_): δ 0.82–1.28 (m, 9H), 1.35 (s, 9H), 1.53–1.82 (m, 10H), 2.01–2.21 (m, 1H), 2.27–2.49 (m, 1H), 2.62–2.71 (m, 2H), 2.74 (s, 3H), 3.62 (br s, 1H), 4.31 (p, *J* = 13.5, 15.6 Hz, 2H), 4.44 (dd, *J* = 4.8, 9.0 Hz, 1H), 4.88–4.96 (m, 2H), 6.19–6.45 (m, 3H), 6.93–7.19 (m, 4H), 7.25 (d, *J* = 7.5 Hz, 1H), 7.86 (br s, 1H), 7.91 (br s, 1H), 9.39 (s, 1H); ^13^C NMR (151 MHz, DMSO-*d*_6_): δ 15.73, 19.94, 25.59, 25.73, 25.98, 28.15, 28.88, 29.04, 29.65, 29.75, 30.19, 34.77, 46.79, 52.27, 52.84, 54.93, 58.57, 74.96, 79.23, 103.14, 106.09, 108.55, 125.88, 126.87, 128.59, 128.79, 130.06, 137.17, 137.36, 155.19, 158.24, 158.75, 169.41, 170.47, 170.99; LC–MS (ESI) 97% purity, *m*/*z*: [M + H]^+^ calcd for C_38_H_53_N_4_O_7_, 677.39; found, 677.7; HRMS (ESI) *m*/*z*: [M + H]^+^ calcd for C_38_H_53_N_4_O_7_, 677.3909; found, 677.3896.

##### *tert*-Butyl *N*-[(1*S*)-2-[[(1*S*)-1-Cyclohexyl-2-oxo-2-[(2*S*,4*S*)-4-phenoxy-2-[[(1*R*)-tetralin-1-yl]carbamoyl]pyrrolidin-1-yl]ethyl]amino]-1-methyl-2-oxo-ethyl]-*N*-methyl-carbamate (**67**)

This compound was synthesized analogously to **63** but using phenol (99 mg, 1.05 mmol) instead of **62** and on a smaller scale (1.0 mmol). A colorless solid was obtained. Yield (185 mg, 28%); *R*_f_ = 0.22 (petroleum ether/EtOAc 1:4); mp 70–74 °C; ^1^H NMR (500 MHz, DMSO-*d*_6_): δ 0.97–1.26 (m, 8H), 1.38 (s, 9H), 1.50–1.84 (m, 11H), 2.06–2.14 (m, 1H), 2.49–2.58 (m, 1H), 2.64–2.74 (m, 5H), 3.62 (dd, *J* = 4.4, 10.7 Hz, 1H), 4.26 (dd, *J* = 6.0, 10.7 Hz, 1H), 4.32 (t, *J* = 7.8 Hz, 1H), 4.45 (dd, *J* = 5.1, 9.1 Hz, 1H), 4.87–4.95 (m, 1H), 4.99–5.07 (m, 1H), 6.83–6.98 (m, 3H), 7.01–7.17 (m, 3H), 7.21–7.32 (m, 3H), 7.63 (br s, 1H), 7.82 (d, *J* = 8.5 Hz, 1H); ^13^C NMR (126 MHz, DMSO-*d*_6_): δ 14.75, 20.01, 25.59, 25.76, 25.91, 28.16, 28.88, 29.09, 29.79, 30.16, 34.54, 36.86, 46.76, 52.18, 53.01, 55.24, 58.71, 75.04, 79.18, 115.70, 121.22, 125.86, 126.86, 128.51, 128.78, 129.70, 137.15, 137.38, 154.94, 157.07, 169.99, 170.43; LC–MS (ESI) 99% purity, *m*/*z*: [M + H]^+^ calcd for C_38_H_53_N_4_O_6_, 661.39; found, 661.5; HRMS (ESI) *m*/*z*: [M + H]^+^ calcd for C_38_H_53_N_4_O_6_, 661.3960; found, 661.3916.

##### (2*S*,4*S*)-1-((*S*)-2-Cyclohexyl-2-((*S*)-2 (Methylamino)propanamido)acetyl)-4-phenoxy-*N*-((*R*)-1,2,3,4-tetrahydronaphthalen-1-yl)pyrrolidine-2-carboxamide (**CST530**)^53^

Compound **67** (70 mg, 106 μmol) was stirred with 1 M HCl in EtOAc (3 mL) for 3 h at rt. Subsequently, it was diluted with EtOAc (25 mL) and washed with saturated NaHCO_3_ solution and brine (each 10 mL), dried over Na_2_SO_4_, filtered, and concentrated in vacuo. The product material was purified by column chromatography (CH_2_Cl_2_/MeOH + 7 N NH_3_ 29:1) to give the title compound as a colorless solid. Yield (49 mg, 83%); *R*_f_ = 0.37 (CH_2_Cl_2_/MeOH + NH_3_ 19:1); mp 80–84 °C; ^1^H NMR (500 MHz, DMSO-*d*_6_): δ 0.88–1.18 (m, 9H), 1.52–1.85 (m, 10H), 2.16 (s, 3H), 2.04–2.13 (m, 1H), 2.50–2.59 (m, 1H), 2.62–2.77 (m, 2H), 2.87–3.00 (m, 1H), 3.64 (dd, *J* = 4.5, 10.8 Hz, 1H), 4.23–4.34 (m, 1H), 4.39 (dd, *J* = 7.1, 8.6 Hz, 1H), 4.45 (dd, *J* = 5.2, 9.0 Hz, 1H), 4.87–4.95 (m, 1H), 5.04 (p, *J* = 5.4 Hz, 1H), 6.88–6.92 (m, 2H), 6.95 (t, *J* = 7.5 Hz, 1H), 7.02–7.19 (m, 3H), 7.21–7.33 (m, 3H), 7.84 (d, *J* = 8.6 Hz, 1H), 7.89 (d, *J* = 8.7 Hz, 1H); ^13^C NMR (126 MHz, DMSO-*d*_6_): δ 19.19, 19.97, 25.55, 25.75, 25.92, 27.91, 28.86, 29.18, 29.77, 34.39, 34.54, 46.73, 52.15, 54.45, 58.63, 59.28, 74.96, 115.69, 121.20, 125.86, 126.84, 128.54, 128.76, 129.69, 137.14, 137.35, 157.06, 169.98, 170.57, 174.56; LC–MS (ESI) 97% purity, *m*/*z*: [M + H]^+^ calcd for C_33_H_45_N_4_O_4_, 561.34; found, 561.3.

##### (2*S*,4*R*)-1-((*S*)-2-Amino-3,3-dimethylbutanoyl)-4-hydroxy-*N*-(4-(4-methylthiazol-5-yl)benzyl)pyrrolidine-2-carboxamide (**68**)

This compound was synthesized as we described previously.^20^

##### (2*S*,4*R*)-1-((*S*)-2-Amino-3,3-dimethylbutanoyl)-4-hydroxy-*N*-((*S*)-1-(4-(4-methylthiazol-5-yl)phenyl)ethyl)pyrrolidine-2-carboxamide (**69**)

This compound was synthesized as described previously.^61,62^

##### (2*S*,4*S*)-1-((*S*)-2-Amino-3,3-dimethylbutanoyl)-4-hydroxy-*N*-(4-(4-methylthiazol-5-yl)benzyl)pyrrolidine-2-carboxamide (**70**)

This compound was synthesized as described previously.^63^

##### (2*S*,4*R*)-4-Hydroxy-*N*-(2-hydroxy-4-(4-methylthiazol-5-yl)benzyl)-1-((*S*)-3-methyl-2-(1-oxoisoindolin-2-yl)butanoyl)pyrrolidine-2-carboxamide (**71**)

This compound was synthesized as we described previously.^60^

##### 2-(2,6-Dioxopiperidin-3-yl)-4-fluoroisoindoline-1,3-dione (**72**)

This compound was synthesized as we described previously.^16^

##### 4-Fluoro-2-(1-methyl-2,6-dioxo-3-piperidyl)isoindoline-1,3-dione (**73**)

This compound was synthesized as we described previously.^20^

#### Synthesis of E3–Linker Conjugates

##### Benzyl 5-(3-(((3*S*,5*S*)-1-((*S*)-2-((*S*)-2-((*tert*-Butoxycarbonyl)(methyl)amino)propanamido)-2-cyclohexylacetyl)-5-(((*R*)-1,2,3,4-tetrahydronaphthalen-1-yl)carbamoyl)pyrrolidin-3-yl)oxy)phenoxy)pentanoate (**74**)

This compound was prepared using general procedure **II**, linker **L1a** (68 mg, 0.30 mmol), and IAP ligand **65**. The crude product was purified by column chromatography (CH_2_Cl_2_/MeOH 39:1) to give a colorless oil. Yield (156 mg, 60%); *R*_f_ = 0.40 (CH_2_Cl_2_/MeOH 39:1); ^1^H NMR (600 MHz, DMSO-*d*_6_): δ 0.77–1.12 (m, 6H), 1.18 (s, 3H), 1.38 (s, 9H), 1.51–1.83 (m, 14H), 2.05–2.12 (m, 1H), 2.38–2.56 (m, 3H), 2.63–2.75 (m, 5H), 3.55–3.63 (m, 1H), 3.85–3.96 (m, 2H), 4.23 (dd, *J* = 6.1, 11.0 Hz, 1H), 4.31 (t, *J* = 7.9 Hz, 1H), 4.44 (dd, *J* = 5.1, 9.1 Hz, 1H), 4.88–5.05 (m, 2H), 5.07 (d, *J* = 1.2 Hz, 2H), 6.40–6.54 (m, 3H), 7.00–7.18 (m, 4H), 7.21–7.26 (m, 1H), 7.28–7.39 (m, 5H), 7.81 (d, *J* = 8.5 Hz, 1H), 8.31 (d, *J* = 8.3 Hz, 1H); ^13^C NMR (151 MHz, DMSO-*d*_6_): δ 15.28, 20.10, 21.38, 25.62, 25.78, 25.94, 28.16, 28.18, 28.90, 29.10, 29.84, 30.18, 33.27, 34.57, 46.80, 52.16, 53.47, 55.26, 58.72, 65.53, 67.20, 75.11, 79.19, 102.50, 107.50, 107.85, 125.88, 126.86, 128.08, 128.14, 128.49, 128.58, 128.81, 130.19, 136.43, 137.16, 137.41, 154.69, 158.26, 160.02, 170.04, 170.46, 172.81; LC–MS (ESI) 96% purity, *m*/*z*: [M + H]^+^ calcd for C_50_H_67_N_4_O_9_, 867.49; found, 867.6; HRMS (ESI) *m*/*z*: [M + H]^+^ calcd for C_50_H_67_N_4_O_9_, 867.4863; found, 867.4861.

##### (2*S*,4*R*)-1-((*S*)-2-(8-Chlorooctanamido)-3,3-dimethylbutanoyl)-4-hydroxy-*N*-(4-(4-methylthiazol-5-yl)benzyl)pyrrolidine-2-carboxamide (**75**)

This compound was synthesized as we described previously.^60^

##### (2*S*,4*R*)-1-((*S*)-2-(4-(4-Chlorobutoxy)butanamido)-3,3-dimethylbutanoyl)-4-hydroxy-*N*-(4-(4-methylthiazol-5-yl)benzyl)pyrrolidine-2-carboxamide (**76**)

This compound was prepared using general procedure **III**, linker **L3a** (97 mg, 0.50 mmol), and VHL ligand **68** (265 mg, 0.62 mmol). The crude product was purified by column chromatography (CH_2_Cl_2_/MeOH 29:1) to give a colorless semi-solid. Yield (191 mg, 63%); *R*_f_ = 0.35 (CH_2_Cl_2_/MeOH 19:1); ^1^H NMR (600 MHz, DMSO-*d*_6_): δ 0.92 (s, 9H), 1.21–1.28 (m, 1H), 1.54–1.62 (m, 2H), 1.61–1.80 (m, 4H), 1.86–1.93 (m, 1H), 1.99–2.06 (m, 1H), 2.13–2.21 (m, 1H), 2.22–2.31 (m, 1H), 2.43 (s, 3H), 3.27–3.40 (m, 3H), 3.56–3.70 (m, 4H), 4.21 (dd, *J* = 5.5, 15.8 Hz, 1H), 4.31–4.37 (m, 1H), 4.39–4.46 (m, 2H), 4.53 (d, *J* = 9.3 Hz, 1H), 5.10 (d, *J* = 3.6 Hz, 1H), 7.35–7.45 (m, 4H), 7.84 (d, *J* = 9.3 Hz, 1H), 8.53 (t, *J* = 6.1 Hz, 1H), 8.97 (s, 1H); ^13^C NMR (151 MHz, DMSO-*d*_6_): δ 16.09, 25.73, 26.53, 26.75, 29.29, 31.78, 35.38, 38.10, 40.24, 41.82, 45.49, 45.51, 56.49, 56.53, 58.86, 69.03, 69.28, 69.61, 127.60, 128.80, 129.81, 131.33, 139.67, 147.89, 151.59, 169.84, 171.95, 172.10; LC–MS (ESI) 95% purity, *m*/*z*: [M + H]^+^ calcd for C_30_H_44_ClN_4_O_5_S, 607.27; found, 607.3; HRMS (ESI) *m*/*z*: [M + H]^+^ calcd for C_30_H_44_ClN_4_O_5_S, 607.2716; found, 607.2707.

##### (2*S*,4*R*)-1-((*S*)-2-(2-(2-(2-Chloroethoxy)ethoxy)acetamido)-3,3-dimethylbutanoyl)-4-hydroxy-*N*-(4-(4-methylthiazol-5-yl)benzyl)pyrrolidine-2-carboxamide (**77**)

This compound was synthesized as we described previously.^60^

##### (2*S*,4*R*)-1-((*S*)-2-(*tert*-Butyl)-14-chloro-4-oxo-6,9,12-trioxa-3-azatetradecanoyl)-4-hydroxy-*N*-(4-(4-methylthiazol-5-yl)benzyl)pyrrolidine-2-carboxamide (**78**)

This compound was synthesized as we described previously.^60^

##### (2*S*,4*R*)-1-((*S*)-2-(2-((6-((6-Chlorohexyl)oxy)hexyl)oxy)acetamido)-3,3-dimethylbutanoyl)-4-hydroxy-*N*-(4-(4-methylthiazol-5-yl)benzyl)pyrrolidine-2-carboxamide (**79**)

This compound was prepared using general procedure **III**, linker **L6a** (147 mg, 0.50 mmol), and VHL ligand **68** (265 mg, 0.62 mmol). The crude product was purified by column chromatography (CH_2_Cl_2_/MeOH 29:1) to give a colorless oil. Yield (233 mg, 66%); *R*_f_ = 0.27 (CH_2_Cl_2_/MeOH 19:1); ^1^H NMR (600 MHz, DMSO-*d*_6_): δ 0.92 (s, 9H), 1.30–1.62 (m, 14H), 1.70–1.78 (m, 2H), 2.05–2.13 (m, 1H), 2.44–2.52 (m, 1H), 2.49 (s, 3H), 3.33–3.38 (m, 4H), 3.41–3.52 (m, 4H), 3.61 (dd, *J* = 3.8, 11.2 Hz, 1H), 3.82–3.94 (m, 2H), 4.01–4.06 (m, 1H), 4.32 (dd, *J* = 5.4, 15.1 Hz, 1H), 4.46 (d, *J* = 8.7 Hz, 1H), 4.48–4.55 (m, 2H), 4.69 (t, *J* = 7.9 Hz, 1H), 7.17 (d, *J* = 8.7 Hz, 1H), 7.33 (s, 4H), 7.40 (t, *J* = 6.0 Hz, 1H), 8.76 (s, 1H). The signal for OH is missing. ^13^C NMR (151 MHz, DMSO-*d*_6_): δ 15.63, 25.48, 25.90, 25.98, 26.34, 26.67, 29.40, 29.65, 32.51, 34.96, 35.90, 43.17, 45.04, 55.24, 56.62, 56.92, 58.50, 69.81, 70.07, 70.70, 70.77, 71.85, 128.14, 129.45, 130.35, 132.11, 138.40, 147.58, 150.75, 170.51, 170.76, 171.27; LC–MS (ESI) 98% purity, *m*/*z*: [M + H]^+^ calcd for C_36_H_56_ClN_4_O_6_S, 707.36; found, 707.6; HRMS (ESI) *m*/*z*: [M + H]^+^ calcd for C_36_H_56_ClN_4_O_6_S, 707.3604; found, 707.3592.

##### (2*S*,4*R*)-1-((*S*)-2-(5-((5-((6-Chlorohexyl)oxy)pentyl)oxy)pentanamido)-3,3-dimethylbutanoyl)-4-hydroxy-*N*-(4-(4-methylthiazol-5-yl)benzyl)pyrrolidine-2-carboxamide (**80**)

This compound was synthesized as we described previously.^60^

##### (2*S*,4*R*)-1-((*S*)-2-(6-((6-((6-Chlorohexyl)oxy)hexyl)oxy)hexanamido)-3,3-dimethylbutanoyl)-4-hydroxy-*N*-(4-(4-methylthiazol-5-yl)benzyl)pyrrolidine-2-carboxamide (**81**)

This compound was prepared using general procedure **III**, linker **L8a** (175 mg, 0.50 mmol), and VHL ligand **68** (265 mg, 0.62 mmol). The crude product was purified by column chromatography (CH_2_Cl_2_/MeOH 29:1) to give a colorless oil. Yield (225 mg, 59%); *R*_f_ = 0.31 (CH_2_Cl_2_/MeOH 19:1); ^1^H NMR (600 MHz, DMSO-*d*_6_): δ 0.92 (s, 9H), 1.20–1.32 (m, 8H), 1.33–1.40 (m, 2H), 1.40–1.55 (m, 10H), 1.65–1.72 (m, 2H), 1.86–1.93 (m, 1H), 1.98–2.05 (m, 1H), 2.06–2.13 (m, 1H), 2.21–2.29 (m, 1H), 2.43 (s, 3H), 3.24–3.33 (m, 8H), 3.60 (t, *J* = 6.6 Hz, 2H), 3.63 (d, *J* = 10.4 Hz, 1H), 3.63–3.69 (m, 1H), 4.20 (dd, *J* = 5.5, 15.8 Hz, 1H), 4.32–4.36 (m, 1H), 4.39–4.45 (m, 2H), 4.53 (d, *J* = 9.3 Hz, 1H), 5.10 (d, *J* = 3.6 Hz, 1H), 7.37 (d, *J* = 8.3 Hz, 2H), 7.41 (d, *J* = 8.1 Hz, 2H), 7.80 (d, *J* = 9.3 Hz, 1H), 8.52 (t, *J* = 6.1 Hz, 1H), 8.96 (s, 1H); ^13^C NMR (151 MHz, DMSO-*d*_6_): δ 16.10, 25.17, 25.48, 25.58, 25.73, 25.76, 26.26, 26.55, 29.17, 29.25, 29.38, 29.41, 32.19, 35.03, 35.37, 38.11, 41.83, 45.51, 56.47, 56.49, 58.87, 69.04, 69.95, 70.06, 127.60, 128.81, 129.81, 131.34, 139.67, 147.89, 151.59, 169.89, 172.12, 172.23; LC–MS (ESI) 98% purity, *m*/*z*: [M + H]^+^ calcd for C_40_H_63_ClN_4_O_6_S, 763.42; found, 763.9; HRMS (ESI) *m*/*z*: [M + H]^+^ calcd for C_40_H_63_ClN_4_O_6_S, 763.4230; found, 763.4215.

##### Benzyl 5-(3-(((3*S*,5*S*)-1-((*S*)-2-((*R*)-2-((*tert*-Butoxycarbonyl)(methyl)amino)propanamido)-2-cyclohexylacetyl)-5-(((*R*)-1,2,3,4-tetrahydronaphthalen-1-yl)carbamoyl)pyrrolidin-3-yl)oxy)phenoxy)pentanoate (**82**)

This compound was prepared using general procedure **II**, linker **L1a** (68 mg, 0.30 mmol), and IAP ligand **66**. The crude product was purified by flash chromatography (gradient from 0 to 5% MeOH in CH_2_Cl_2_) to give a colorless oil. Yield (156 mg, 60%); *R*_f_ = 0.40 (CH_2_Cl_2_/MeOH 39:1); ^1^H NMR (600 MHz, DMSO-*d*_6_): δ 0.82–1.17 (m, 6H), 1.24 (s, 3H), 1.35 (s, 9H), 1.49–1.85 (m, 14H), 2.00–2.19 (m, 1H), 2.33–2.47 (m, 3H), 2.61–2.81 (m, 5H), 3.54–3.69 (m, 1H), 3.85–3.93 (m, 2H), 4.23–4.39 (m, 2H), 4.43 (dd, *J* = 4.9, 9.0 Hz, 1H), 4.87–5.03 (m, 2H), 5.08 (s, 2H), 6.39–6.58 (m, 3H), 7.00–7.18 (m, 4H), 7.25 (d, *J* = 7.5 Hz, 1H), 7.29–7.38 (m, 5H), 7.83 (s, 1H), 7.92 (s, 1H); ^13^C NMR (151 MHz, DMSO-*d*_6_): δ 15.75, 20.00, 21.34, 25.60, 25.73, 25.98, 28.15, 28.87, 29.02, 29.77, 30.19, 33.26, 34.67, 46.79, 52.41, 53.80, 54.95, 58.63, 65.51, 67.18, 75.13, 78.97, 102.46, 107.52, 107.81, 125.85, 126.86, 128.06, 128.12, 128.57, 128.79, 130.17, 136.42, 137.15, 137.98, 155.18, 158.21, 160.00, 169.88, 170.43, 172.17, 172.78; LC–MS (ESI) 99% purity, *m*/*z*: [M + H]^+^ calcd for C_50_H_67_N_4_O_9_, 867.49; found, 867.8; HRMS (ESI) *m*/*z*: [M + H]^+^ calcd for C_50_H_67_N_4_O_9_, 867.4863; found, 867.4886.

##### (2*S*,4*R*)-*N*-(2-((5-Chloropentyl)oxy)-4-(4-methylthiazol-5-yl)benzyl)-4-hydroxy-1-((*S*)-3-methyl-2-(1-oxoisoindolin-2-yl)butanoyl)pyrrolidine-2-carboxamide (**83**)

This compound was prepared using general procedure **IV** and linker **L1b** (240 mg, 1.20 mmol). The crude product was purified by column chromatography (CH_2_Cl_2_/MeOH 29:1) to give a colorless solid. Yield (346 mg, 53%); *R*_f_ = 0.30 (CH_2_Cl_2_/MeOH 19:1); mp 84–86 °C; ^1^H NMR (600 MHz, DMSO-*d*_6_): δ 0.73 (d, *J* = 6.7 Hz, 3H), 0.95 (d, *J* = 6.6 Hz, 3H), 1.52–1.62 (m, 2H), 1.71–1.84 (m, 4H), 1.88–1.95 (m, 1H), 2.00–2.09 (m, 1H), 2.27–2.36 (m, 1H), 2.46 (s, 3H), 3.64–3.71 (m, 3H), 3.74–3.81 (m, 1H), 4.05 (t, *J* = 6.2 Hz, 2H), 4.17–4.36 (m, 3H), 4.37–4.58 (m, 3H), 4.70 (d, *J* = 10.8 Hz, 1H), 5.08 (d, *J* = 4.1 Hz, 1H), 6.96–7.03 (m, 2H), 7.32 (d, *J* = 7.7 Hz, 1H), 7.45–7.53 (m, 1H), 7.56–7.64 (m, 2H), 7.70 (d, *J* = 7.6 Hz, 1H), 8.36 (t, *J* = 5.9 Hz, 1H), 8.98 (s, 1H); ^13^C NMR (151 MHz, DMSO-*d*_6_): δ 16.21, 18.80, 19.06, 23.23, 28.11, 28.57, 31.94, 37.21, 38.28, 45.54, 46.99, 55.59, 57.96, 58.87, 67.71, 68.79, 111.91, 120.98, 123.20, 123.81, 127.15, 127.86, 128.09, 131.17, 131.49, 131.56, 131.77, 142.39, 148.09, 151.65, 156.10, 167.66, 168.28, 171.72; LC–MS (ESI) 98% purity, *m*/*z*: [M + H]^+^ calcd for C_34_H_42_ClN_4_O_5_S, 653.26; found, 653.3; HRMS (ESI) *m*/*z*: [M + H]^+^ calcd for C_34_H_42_ClN_4_O_5_S, 653.2559; found, 653.2548.

##### (2*S*,4*R*)-*N*-(2-((8-Chlorooctyl)oxy)-4-(4-methylthiazol-5-yl)benzyl)-4-hydroxy-1-((*S*)-3-methyl-2-(1-oxoisoindolin-2-yl)butanoyl)pyrrolidine-2-carboxamide (**84**)

This compound was synthesized as we described previously.^60^

##### (2*S*,4*R*)-*N*-(2-(4-(4-Chlorobutoxy)butoxy)-4-(4-methylthiazol-5-yl)benzyl)-4-hydroxy-1-((*S*)-3-methyl-2-(1-oxoisoindolin-2-yl)butanoyl)pyrrolidine-2-carboxamide (**85**)

This compound was prepared using general procedure **IV** and linker **L3b** (310 mg, 1.20 mmol). The crude product was purified by column chromatography (CH_2_Cl_2_/MeOH 29:1) to give a colorless solid. Yield (302 mg, 60%); *R*_f_ = 0.37 (CH_2_Cl_2_/MeOH 29:1); mp 78–80 °C; ^1^H NMR (600 MHz, DMSO-*d*_6_): δ 0.73 (d, *J* = 6.8 Hz, 3H), 0.95 (d, *J* = 6.5 Hz, 3H), 1.56–1.64 (m, 2H), 1.64–1.83 (m, 6H), 1.88–1.95 (m, 1H), 1.99–2.07 (m, 1H), 2.26–2.37 (m, 1H), 2.46 (s, 3H), 3.34–3.47 (m, 4H), 3.62 (t, *J* = 6.7 Hz, 2H), 3.65–3.71 (m, 1H), 3.77 (dd, *J* = 4.4, 10.6 Hz, 1H), 4.06 (t, *J* = 6.3 Hz, 2H), 4.18–4.35 (m, 3H), 4.37–4.59 (m, 3H), 4.70 (d, *J* = 10.9 Hz, 1H), 5.08 (d, *J* = 4.1 Hz, 1H), 6.96–7.01 (m, 2H), 7.32 (d, *J* = 7.7 Hz, 1H), 7.49 (ddd, *J* = 2.2, 6.2, 8.1 Hz, 1H), 7.56–7.64 (m, 2H), 7.70 (dd, *J* = 1.0, 7.6 Hz, 1H), 8.36 (t, *J* = 6.0 Hz, 1H), 8.98 (s, 1H); ^13^C NMR (151 MHz, DMSO-*d*_6_): δ 16.20, 18.81, 19.06, 25.86, 26.07, 26.80, 28.57, 29.35, 37.22, 38.27, 45.52, 46.99, 55.60, 57.96, 58.88, 67.74, 68.80, 69.32, 69.85, 111.88, 120.95, 123.20, 123.81, 127.15, 127.88, 128.09, 131.16, 131.49, 131.56, 131.77, 142.39, 148.07, 151.65, 156.09, 167.66, 168.28, 171.71; LC–MS (ESI) 98% purity, *m*/*z*: [M + H]^+^ calcd for C_37_H_48_ClN_4_O_6_S, 711.30; found, 711.5; HRMS (ESI) *m*/*z*: [M + H]^+^ calcd for C_37_H_48_ClN_4_O_6_S, 711.2978; found, 711.2965.

##### (2*S*,4*R*)-*N*-(2-(2-(2-(2-Chloroethoxy)ethoxy)ethoxy)-4-(4-methylthiazol-5-yl)benzyl)-4-hydroxy-1-((*S*)-3-methyl-2-(1-oxoisoindolin-2-yl)butanoyl)pyrrolidine-2-carboxamide (**86**)

This compound was synthesized as we described previously.^60^

##### (2*S*,4*R*)-*N*-(2-(2-(2-(2-(2-Chloroethoxy)ethoxy)ethoxy)ethoxy)-4-(4-methylthiazol-5-yl)benzyl)-4-hydroxy-1-((*S*)-3-methyl-2-(1-oxoisoindolin-2-yl)butanoyl)pyrrolidine-2-carboxamide (**87**)

This compound was synthesized as we described previously.^60^

##### (2*S*,4*R*)-*N*-(2-(2-((6-((6-Chlorohexyl)oxy)hexyl)oxy)ethoxy)-4-(4-methylthiazol-5-yl)benzyl)-4-hydroxy-1-((*S*)-3-methyl-2-(1-oxoisoindolin-2-yl)butanoyl)pyrrolidine-2-carboxamide (**88**)

This compound was prepared using general procedure **IV** and linker **L6b** (430 mg, 1.20 mmol). The crude product was purified by flash chromatography (gradient from 0 to 5% MeOH in CH_2_Cl_2_), followed by preparative HPLC (gradient from 70 to 100% MeOH) to separate the inversely attached linker–ligand side product. A colorless resin of the chloroalkane product was obtained. Yield (316 mg, 39%); *R*_f_ = 0.29 (CH_2_Cl_2_/MeOH 19:1); ^1^H NMR (600 MHz, DMSO-*d*_6_): δ 0.73 (d, *J* = 6.7 Hz, 3H), 0.95 (d, *J* = 6.6 Hz, 3H), 1.24–1.39 (m, 8H), 1.40–1.55 (m, 6H), 1.64–1.72 (m, 2H), 1.88–1.95 (m, 1H), 1.99–2.07 (m, 1H), 2.27–2.37 (m, 1H), 2.46 (s, 3H), 3.26–3.32 (m, 4H), 3.47 (t, *J* = 6.5 Hz, 2H), 3.59 (t, *J* = 6.6 Hz, 2H), 3.65–3.80 (m, 4H), 4.13–4.36 (m, 5H), 4.37–4.60 (m, 3H), 4.71 (d, *J* = 10.8 Hz, 1H), 5.06 (s, 1H), 7.00 (dd, *J* = 1.6, 7.8 Hz, 1H), 7.05 (d, *J* = 1.7 Hz, 1H), 7.33 (d, *J* = 7.8 Hz, 1H), 7.45–7.53 (m, 1H), 7.57–7.64 (m, 2H), 7.70 (d, *J* = 7.6 Hz, 1H), 8.32 (t, *J* = 6.0 Hz, 1H), 8.97 (s, 1H); ^13^C NMR (151 MHz, DMSO-*d*_6_): δ 16.16, 18.76, 19.03, 25.15, 25.64, 25.72, 26.25, 28.54, 29.23, 29.36, 32.17, 37.21, 38.22, 45.49, 46.96, 55.54, 57.94, 58.86, 68.12, 68.77, 68.82, 69.93, 70.05, 70.61, 112.42, 121.21, 123.16, 123.75, 127.37, 127.85, 128.05, 131.13, 131.41, 131.54, 131.72, 142.34, 148.06, 151.58, 156.08, 167.63, 168.25, 171.68; LC–MS (ESI) 99% purity, *m*/*z*: [M + H]^+^ calcd for C_43_H_60_ClN_4_O_7_S, 811.39; found, 811.8; HRMS (ESI) *m*/*z*: [M + H]^+^ calcd for C_43_H_60_ClN_4_O_7_S, 811.3866; found, 811.3860.

##### (2*S*,4*R*)-*N*-(2-((5-((5-((6-Chlorohexyl)oxy)pentyl)oxy)pentyl)oxy)-4-(4-methylthiazol-5-yl)benzyl)-4-hydroxy-1-((*S*)-3-methyl-2-(1-oxoisoindolin-2-yl)butanoyl)pyrrolidine-2-carboxamide (**89**)

This compound was prepared using general procedure **IV** and linker **L7b** (464 mg, 1.20 mmol). The crude product was purified by flash chromatography (gradient from 0 to 5% MeOH in CH_2_Cl_2_) to give a colorless resin. Yield (411 mg, 49%); *R*_f_ = 0.35 (CH_2_Cl_2_/MeOH 19:1); ^1^H NMR (600 MHz, DMSO-*d*_6_): δ 0.73 (d, *J* = 6.7 Hz, 3H), 0.95 (d, *J* = 6.5 Hz, 3H), 1.24–1.40 (m, 6H), 1.41–1.52 (m, 8H), 1.52–1.64 (m, 2H), 1.65–1.72 (m, 2H), 1.73–1.83 (m, 2H), 1.86–1.97 (m, 1H), 1.99–2.07 (m, 1H), 2.27–2.39 (m, 1H), 2.46 (s, 3H), 3.31–3.39 (m, 8H), 3.59 (t, *J* = 6.6 Hz, 2H), 3.62–3.73 (m, 1H), 3.73–3.82 (m, 1H), 4.04 (t, *J* = 6.3 Hz, 2H), 4.18–4.59 (m, 6H), 4.71 (d, *J* = 10.8 Hz, 1H), 5.06 (s, 1H), 6.96–7.02 (m, 2H), 7.32 (d, *J* = 7.7 Hz, 1H), 7.45–7.55 (m, 1H), 7.57–7.64 (m, 2H), 7.70 (d, *J* = 7.6 Hz, 1H), 8.33 (t, *J* = 5.9 Hz, 1H), 8.97 (s, 1H); ^13^C NMR (151 MHz, DMSO-*d*_6_): δ 16.15, 18.76, 19.02, 22.55, 22.68, 25.13, 26.24, 28.53, 28.64, 29.10, 29.19, 29.22, 32.17, 37.17, 38.21, 45.48, 46.96, 55.53, 57.94, 58.84, 67.86, 68.77, 69.92, 70.01, 70.04, 70.08, 111.86, 120.90, 123.16, 123.75, 127.14, 127.84, 128.04, 131.13, 131.46, 131.53, 131.72, 142.35, 148.03, 151.56, 156.10, 167.63, 168.26, 171.66; LC–MS (ESI) 94% purity, *m*/*z*: [M + H]^+^ calcd for C_45_H_64_ClN_4_O_7_S, 839.42; found, 839.8; HRMS (ESI) *m*/*z*: [M + H]^+^ calcd for C_45_H_64_ClN_4_O_7_S, 839.4179; found, 839.4160.

##### (2*S*,4*R*)-*N*-(2-((6-((6-((6-Chlorohexyl)oxy)hexyl)oxy)hexyl)oxy)-4-(4-methylthiazol-5-yl)benzyl)-4-hydroxy-1-((*S*)-3-methyl-2-(1-oxoisoindolin-2-yl)butanoyl)pyrrolidine-2-carboxamide (**90**)

This compound was prepared using general procedure **IV** and linker **L8b** (498 mg, 1.20 mmol). The crude product was purified by column chromatography (CH_2_Cl_2_/MeOH 29:1) to give a colorless resin. Yield (269 mg, 31%); *R*_f_ = 0.27 (CH_2_Cl_2_/MeOH 19:1); ^1^H NMR (600 MHz, DMSO-*d*_6_): δ 0.73 (d, *J* = 6.7 Hz, 3H), 0.95 (d, *J* = 6.5 Hz, 3H), 1.24–1.41 (m, 10H), 1.42–1.55 (m, 10H), 1.64–1.71 (m, 2H), 1.71–1.79 (m, 2H), 1.88–1.95 (m, 1H), 1.99–2.06 (m, 1H), 2.27–2.35 (m, 1H), 2.46 (s, 3H), 3.27–3.36 (m, 8H), 3.59 (t, *J* = 6.6 Hz, 2H), 3.67 (d, *J* = 10.6 Hz, 1H), 3.77 (dd, *J* = 4.5, 10.6 Hz, 1H), 4.03 (t, *J* = 6.3 Hz, 2H), 4.16–4.60 (m, 6H), 4.70 (d, *J* = 10.8 Hz, 1H), 5.06 (d, *J* = 4.1 Hz, 1H), 6.96–7.01 (m, 2H), 7.32 (d, *J* = 7.6 Hz, 1H), 7.46–7.52 (m, 1H), 7.57–7.64 (m, 2H), 7.70 (d, *J* = 7.6 Hz, 1H), 8.33 (t, *J* = 6.0 Hz, 1H), 8.97 (s, 1H); ^13^C NMR (151 MHz, DMSO-*d*_6_): δ 16.17, 18.78, 19.05, 25.17, 25.58, 25.66, 25.74, 26.26, 28.55, 28.83, 29.25, 29.38, 32.20, 37.19, 38.23, 45.51, 46.99, 55.56, 57.97, 58.87, 67.86, 68.79, 69.95, 70.03, 70.06, 111.87, 120.93, 123.19, 123.77, 127.17, 127.88, 128.07, 131.15, 131.49, 131.55, 131.75, 142.37, 148.05, 151.58, 156.13, 167.66, 168.29, 171.68; LC–MS (ESI) 96% purity, *m*/*z*: [M + H]^+^ calcd for C_47_H_68_ClN_4_O_7_S, 867.45; found, 867.6; HRMS (ESI) *m*/*z*: [M + H]^+^ calcd for C_47_H_68_ClN_4_O_7_S, 867.4492; found, 867.4482.

##### *tert*-Butyl ((*S*)-1-(((*S*)-2-((2*S*,4*S*)-4-(3-((5-Chloropentyl)oxy)phenoxy)-2-(((*R*)-1,2,3,4-tetrahydronaphthalen-1-yl)carbamoyl)pyrrolidin-1-yl)-1-cyclohexyl-2-oxoethyl)amino)-1-oxopropan-2-yl)(methyl)carbamate (**91**)

This compound was prepared using general procedure **V** and linker **L1b** (72 mg, 0.36 mmol). The crude product was purified by column chromatography (CH_2_Cl_2_/MeOH 50:1) to give a pale yellow oil. Yield (0.18 g, 75%); *R*_f_ = 0.20 (CH_2_Cl_2_/MeOH 50:1); ^1^H NMR (400 MHz, CDCl_3_): δ 0.75–1.00 (m, 5H), 1.30 (d, *J* = 7.1 Hz, 3H), 1.37–1.42 (m, 1H), 1.47 (s, 9H), 1.49–1.57 (m, 5H), 1.71–1.88 (m, 8H), 1.98–2.10 (m, 1H), 2.26–2.37 (m, 1H), 2.68–2.81 (m, 5H), 2.92 (s, 1H), 3.52–3.60 (m, 2H), 3.71–3.84 (m, 2H), 3.89 (t, *J* = 6.3 Hz, 2H), 4.18 (dd, *J* = 11.3, 4.8 Hz, 1H), 4.42 (t, *J* = 7.9 Hz, 1H), 4.55–4.70 (m, 1H), 4.76 (d, *J* = 9.3 Hz, 1H), 4.94 (d, *J* = 4.8 Hz, 1H), 5.13 (q, *J* = 5.0 Hz, 1H), 6.33–6.43 (m, 2H), 6.53 (dd, *J* = 8.2, 2.2 Hz, 1H), 6.59 (d, *J* = 8.3 Hz, 2H), 7.02–7.19 (m, 4H), 7.27–7.32 (m, 1H); ^13^C NMR (101 MHz, CDCl_3_): δ; 19.89, 23.41, 25.34, 25.45, 25.71, 28.23, 28.42, 28.72, 28.85, 29.10, 29.57, 29.81, 31.27, 33.25, 34.90, 40.31, 42.34, 42.71, 47.42, 48.70, 53.49, 54.65, 59.91, 61.54, 67.42, 72.10, 76.07, 102.59, 107.29, 108.18, 125.99, 126.89, 128.54, 128.89, 129.90, 136.50, 137.25, 157.77, 160.09, 170.94, 172.37; HRMS (ESI) *m*/*z*: [M + H]^+^ calcd for C_43_H_62_O_7_N_4_Cl, 781.4302; found, 781.4284.

##### *tert*-Butyl ((*S*)-1-(((*S*)-2-((2*S*,4*S*)-4-(3-((8-Chlorooctyl)oxy)phenoxy)-2-(((*R*)-1,2,3,4-tetrahydronaphthalen-1-yl)carbamoyl)pyrrolidin-1-yl)-1-cyclohexyl-2-oxoethyl)amino)-1-oxopropan-2-yl)(methyl)carbamate (**92**)

This compound was prepared using general procedure **V** and linker **L2b** (87 mg, 0.36 mmol). The crude product was purified by column chromatography (CH_2_Cl_2_/MeOH 50:1) to give a colorless oil. Yield (0.20 g, 82%); *R*_f_ = 0.22 (CH_2_Cl_2_/MeOH 50:1); ^1^H NMR (400 MHz, CDCl_3_): δ 0.75–0.97 (m, 5H), 1.30 (d, *J* = 7.1 Hz, 3H), 1.32–1.44 (m, 8H), 1.47 (s, 9H), 1.50–1.58 (m, 4H), 1.69–1.86 (m, 8H), 1.98–2.09 (m, 1H), 2.27–2.37 (m, 1H), 2.70–2.80 (m, 5H), 2.90–2.94 (m, 1H), 3.53 (t, *J* = 6.7 Hz, 2H), 3.75–3.84 (m, 2H), 3.87 (t, *J* = 6.5 Hz, 2H), 4.17 (dd, *J* = 11.5, 4.8 Hz, 1H), 4.42 (d, *J* = 7.9 Hz, 1H), 4.59–4.69 (m, 1H), 4.76 (dd, *J* = 9.7, 2.1 Hz, 1H), 4.93 (d, *J* = 5.1 Hz, 1H), 5.13 (q, *J* = 7.6 Hz, 1H), 6.31–6.41 (m, 2H), 6.51–6.55 (m, 1H), 6.58 (d, *J* = 8.3 Hz, 1H), 7.01–7.17 (m, 5H), 7.27–7.32 (m, 1H); ^13^C NMR (101 MHz, CDCl_3_): δ 22.73, 25.45, 25.57, 25.82, 25.87, 26.77, 28.49, 28.78, 29.14, 29.22, 29.70, 29.93, 31.36, 33.42, 35.07, 40.46, 40.47, 42.56, 47.49, 47.54, 48.80, 53.56, 54.70, 60.04, 60.19, 67.83, 74.19, 102.68, 102.83, 107.73, 107.85, 107.97, 126.14, 127.01, 128.70, 128.99, 129.97, 136.57, 137.34, 160.38, 171.00, 172.54, 175.16; HRMS (ESI) *m*/*z*: [M + H]^+^ calcd for C_46_H_68_O_7_N_4_Cl, 823.4771; found, 823.4753.

##### *tert*-Butyl ((*S*)-1-(((*S*)-2-((2*S*,4*S*)-4-(3-(4-(4-Chlorobutoxy)butoxy)phenoxy)-2-(((*R*)-1,2,3,4-tetrahydronaphthalen-1-yl)carbamoyl)pyrrolidin-1-yl)-1-cyclohexyl-2-oxoethyl)amino)-1-oxopropan-2-yl)(methyl)carbamate (**93**)

This compound was prepared using general procedure **V** and linker **L3b** (165 mg, 0.64 mmol). The crude product was purified by column chromatography (CH_2_Cl_2_/MeOH 50:1) to give a colorless oil. Yield (0.30 g, 69%); *R*_f_ = 0.35 (CH_2_Cl_2_/MeOH 50:1); ^1^H NMR (400 MHz, CDCl_3_): δ 0.75–0.97 (m, 5H), 1.30 (d, *J* = 7.1 Hz, 3H), 1.37–1.44 (m, 1H), 1.47 (s, 9H), 1.49–1.59 (m, 4H), 1.69–1.89 (m, 10H), 1.98–2.08 (m, 1H), 2.28–2.38 (m, 1H), 2.71–2.79 (m, 5H), 2.86–2.93 (m, 1H), 3.42–3.52 (m, 6H), 3.57 (t, *J* = 6.6 Hz, 2H), 3.73–3.87 (m, 2H), 3.90 (d, *J* = 6.2 Hz, 2H), 4.18 (dd, *J* = 11.5, 4.8 Hz, 1H), 4.42 (t, *J* = 7.9 Hz, 1H), 4.57–4.70 (m, 0H), 4.76 (dd, *J* = 9.9, 2.2 Hz, 1H), 4.94 (dd, *J* = 82.8, 5.0 Hz, 1H), 5.09–5.16 (m, 1H), 6.32–6.42 (m, 2H), 6.51–6.55 (m, 1H), 6.58 (d, *J* = 8.4 Hz, 2H), 7.02–7.18 (m, 4H), 7.27–7.32 (m, 1H); ^13^C NMR (101 MHz, CDCl_3_): δ 22.73, 25.46, 25.56, 25.83, 26.08, 26.22, 26.28, 27.01, 28.21, 28.49, 29.22, 29.68, 29.93, 31.37, 33.42, 35.00, 40.48, 42.41, 47.52, 48.81, 53.55, 54.73, 60.01, 67.60, 73.52, 102.74, 102.87, 107.52, 108.15, 126.12, 127.00, 128.73, 128.99, 129.98, 136.60, 137.36, 157.90, 160.29, 171.08, 172.51, 175.11; HRMS (ESI) *m*/*z*: [M + H]^+^ calcd for C_46_H_68_O_8_N_4_Cl, 839.4720; found, 839.4703.

##### *tert*-Butyl ((*S*)-1-(((*S*)-2-((2*S*,4*S*)-4-(3-(2-(2-(2-Chloroethoxy)ethoxy)ethoxy)phenoxy)-2-(((*R*)-1,2,3,4-tetrahydronaphthalen-1-yl)carbamoyl)pyrrolidin-1-yl)-1-cyclohexyl-2-oxoethyl)amino)-1-oxopropan-2-yl)(methyl)carbamate (**94**)

This compound was prepared using general procedure **V** and linker **L4b** (74 mg, 0.30 mmol). The crude product was purified by column chromatography (CH_2_Cl_2_/MeOH 50:1) to give a pale yellow oil. Yield (0.14 g, 66%); *R*_f_ = 0.18 (CH_2_Cl_2_/MeOH 20:1); ^1^H NMR (400 MHz, CDCl_3_): δ 0.78–0.95 (m, 5H), 1.30 (d, *J* = 7.1 Hz, 3H), 1.37–1.43 (m, 1H), 1.47 (s, 9H), 1.56 (s, 4H), 1.75–1.85 (m, 4H), 1.99–2.08 (m, 1H), 2.27–2.36 (m, 1H), 2.71–2.80 (m, 5H), 2.90 (d, *J* = 14.1 Hz, 1H), 3.63 (td, *J* = 5.8, 0.6 Hz, 2H), 3.68–3.82 (m, 7H), 3.82–3.87 (m, 2H), 4.07 (d, *J* = 4.6 Hz, 2H), 4.19 (dd, *J* = 11.4, 4.8 Hz, 1H), 4.41 (t, *J* = 7.9 Hz, 1H), 4.57–4.69 (m, 1H), 4.76 (d, *J* = 9.6 Hz, 1H), 4.93 (d, *J* = 4.2 Hz, 1H), 5.12 (q, *J* = 7.7 Hz, 1H), 6.37–6.42 (m, 2H), 6.56 (d, *J* = 7.2 Hz, 1H), 6.56–6.62 (m, 2H), 7.02–7.18 (m, 4H), 7.27–7.31 (m, 1H); ^13^C NMR (101 MHz, CDCl_3_): δ 20.08, 25.53, 25.64, 25.89, 28.42, 28.52, 29.34, 29.82, 30.01, 33.48, 40.64, 42.84, 47.70, 53.68, 55.33, 60.18, 67.50, 69.83, 70.78, 70.88, 71.47, 76.25, 103.07, 107.72, 108.48, 126.30, 127.22, 128.73, 129.18, 130.18, 136.64, 137.47, 157.89, 160.13, 169.46, 171.68, 172.36; HRMS (ESI) *m*/*z*: [M + H]^+^ calcd for C_44_H_64_O_9_N_4_Cl, 827.4356; found, 827.4340.

##### *tert*-Butyl ((*S*)-1-(((*S*)-2-((2*S*,4*S*)-4-(3-(2-(2-(2-(2-Chloroethoxy)ethoxy)ethoxy)ethoxy)phenoxy)-2-(((*R*)-1,2,3,4-tetrahydronaphthalen-1-yl)carbamoyl)pyrrolidin-1-yl)-1-cyclohexyl-2-oxoethyl)amino)-1-oxopropan-2-yl)(methyl)carbamate (**95**)

This compound was synthesized as we described previously.^60^

##### *tert*-Butyl ((*S*)-1-(((*S*)-2-((2*S*,4*S*)-4-(3-(2-((6-((6-Chlorohexyl)oxy)hexyl)oxy)ethoxy)phenoxy)-2-(((*R*)-1,2,3,4-tetrahydronaphthalen-1-yl)carbamoyl)pyrrolidin-1-yl)-1-cyclohexyl-2-oxoethyl)amino)-1-oxopropan-2-yl)(methyl)carbamate (**96**)

This compound was prepared using general procedure **V** and linker **L6b** (118 mg, 0.33 mmol). The crude product was purified by column chromatography (CH_2_Cl_2_/MeOH 50:1) to give a colorless oil. Yield (0.15 g, 58%); *R*_f_ = 0.30 (CH_2_Cl_2_/MeOH 50:1); ^1^H NMR (400 MHz, CDCl_3_): δ 0.79–0.99 (m, 5H), 1.30 (d, *J* = 7.1 Hz, 3H), 1.33–1.43 (m, 8H), 1.47 (s, 9H), 1.50–1.58 (m, 10H), 1.72–1.86 (m, 6H), 1.99–2.08 (m, 1H), 2.26–2.37 (m, 1H), 2.70–2.81 (m, 5H), 2.90 (d, *J* = 14.1 Hz, 1H), 3.39 (td, *J* = 6.6, 1.5 Hz, 4H), 3.46–3.57 (m, 5H), 3.75 (d, *J* = 3.5 Hz, 2H), 3.80 (d, *J* = 11.5 Hz, 1H), 4.04 (t, *J* = 4.6 Hz, 2H), 4.18 (dd, *J* = 11.4, 4.9 Hz, 1H), 4.42 (t, *J* = 7.9 Hz, 1H), 4.56–4.69 (m, 1H), 4.76 (dd, *J* = 9.8, 2.2 Hz, 1H), 4.93 (t, *J* = 4.8 Hz, 1H), 5.13 (d, *J* = 6.1 Hz, 1H), 6.36–6.42 (m, 2H), 6.55 (dd, *J* = 2.3, 0.8 Hz, 1H), 6.56–6.62 (m, 2H), 7.03–7.18 (m, 4H), 7.27–7.31 (m, 1H); ^13^C NMR (101 MHz, CDCl_3_): δ 22.74, 25.45, 25.56, 25.81, 25.90, 26.01, 26.73, 28.42, 28.48, 29.16, 29.22, 29.55, 29.58, 29.63, 29.70, 29.93, 31.36, 33.38, 35.08, 40.44, 42.53, 47.54, 48.79, 53.56, 54.72, 60.04, 60.20, 67.38, 69.04, 70.59, 70.80, 74.01, 102.88, 107.64, 108.32, 126.15, 127.04, 128.63, 129.00, 129.99, 136.57, 137.34, 157.81, 160.08, 170.99, 172.54, 175.19; HRMS (ESI) *m*/*z*: [M + H]^+^ calcd for C_52_H_80_O_9_N_4_Cl, 939.5608; found, 939.5650.

##### *tert*-Butyl ((*S*)-1-(((*S*)-2-((2*S*,4*S*)-4-(3-((5-((5-((6-Chlorohexyl)oxy)pentyl)oxy)pentyl)oxy)phenoxy)-2-(((*R*)-1,2,3,4-tetrahydronaphthalen-1-yl)carbamoyl)pyrrolidin-1-yl)-1-cyclohexyl-2-oxoethyl)amino)-1-oxopropan-2-yl)(methyl)carbamate (**97**)

This compound was prepared using general procedure **V** and linker **L7b** (254 mg, 0.66 mmol). The crude product was purified by column chromatography (CH_2_Cl_2_/MeOH 50:1) to give a pale yellow oil. Yield (0.36 g, 67%); *R*_f_ = 0.28 (CH_2_Cl_2_/MeOH 50:1); ^1^H NMR (400 MHz, CDCl_3_): δ 0.77–0.98 (m, 5H), 1.29 (d, *J* = 7.1 Hz, 3H), 1.33–1.44 (m, 5H), 1.46 (s, 9H), 1.50–1.65 (m, 15H), 1.72–1.86 (m, 8H), 1.98–2.10 (m, 1H), 2.27–2.37 (m, 1H), 2.75 (d, *J* = 4.6 Hz, 5H), 2.89–2.93 (m, 1H), 3.36–3.44 (m, 8H), 3.52 (t, *J* = 6.7 Hz, 2H), 3.74–3.85 (m, 2H), 3.88 (t, *J* = 6.5 Hz, 2H), 4.17 (dd, *J* = 11.4, 4.7 Hz, 1H), 4.41 (t, *J* = 7.9 Hz, 1H), 4.58–4.69 (m, 1H), 4.75 (dd, *J* = 9.8, 2.2 Hz, 1H), 4.93 (t, *J* = 4.8 Hz, 1H), 5.12 (q, *J* = 6.1 Hz, 1H), 6.34–6.40 (m, 2H), 6.50–6.54 (m, 1H), 6.55–6.71 (m, 2H), 7.02–7.17 (m, 4H), 7.27–7.31 (m, 1H); ^13^C NMR (101 MHz, CDCl_3_): δ 22.73, 25.44, 25.55, 25.81, 25.90, 26.74, 28,24, 28.47, 29.04, 29.15, 29.22, 29.45, 29.51, 29.58, 29.70, 29.93, 31.36, 33.42, 35.09, 40.45, 42.53, 47.53, 48.79, 53.56, 54.70, 60.05, 60.20, 67.78, 74.83, 76.17, 102.86, 107.62, 107.82, 107.87, 126.14, 127.02, 128.62, 128.99, 129.96, 136.55, 137.33, 160.35, 167.57, 170.99, 172.53, 175.19; HRMS (ESI) *m*/*z*: [M + H]^+^ calcd for C_54_H_84_O_9_N_4_Cl, 967.5921; found, 967.5918.

##### *tert*-Butyl ((*S*)-1-(((*S*)-2-((2*S*,4*S*)-4-(3-((6-((6-((6-Chlorohexyl)oxy)hexyl)oxy)hexyl)oxy)phenoxy)-2-(((*R*)-1,2,3,4-tetrahydronaphthalen-1-yl)carbamoyl)pyrrolidin-1-yl)-1-cyclohexyl-2-oxoethyl)amino)-1-oxopropan-2-yl)(methyl)carbamate (**98**)

This compound was prepared using general procedure **V** and linker **L8b** (136 mg, 0.33 mmol). The crude product was purified by column chromatography (CH_2_Cl_2_/MeOH 50:1) to give a colorless oil. Yield (0.24 g, 88%); *R*_f_ = 0.25 (CH_2_Cl_2_/MeOH 50:1); ^1^H NMR (400 MHz, CDCl_3_): δ 0.78–1.01 (m, 5H), 1.30 (d, *J* = 7.1 Hz, 3H), 1.34–1.44 (m, 12H), 1.47 (s, 9H), 1.51–1.59 (m, 10H), 1.73–1.84 (m, 8H), 1.99–2.09 (m, 1H), 2.28–2.38 (m, 1H), 2.72–2.79 (m, 5H), 2.89 (d, *J* = 14.1 Hz, 1H), 3.37–3.43 (m, 8H), 3.49 (d, *J* = 5.2 Hz, 2H), 3.53 (t, *J* = 6.7 Hz, 2H), 3.77–3.85 (m, 2H), 3.87 (t, *J* = 6.5 Hz, 2H), 4.17 (dd, *J* = 11.5, 4.8 Hz, 1H), 4.42 (t, *J* = 8.0 Hz, 1H), 4.57–4.69 (m, 1H), 4.76 (d, *J* = 9.6 Hz, 1H), 4.94 (d, *J* = 4.3 Hz, 1H), 5.14 (d, *J* = 4.8 Hz, 1H), 6.31–6.41 (m, 2H), 6.51–6.55 (m, 1H), 6.58 (d, *J* = 8.4 Hz, 1H), 6.68 (s, 1H), 7.03–7.18 (m, 4H), 7.28–7.31 (m, 1H); ^13^C NMR (101 MHz, CDCl_3_): δ 22.74, 25.45, 25.56, 25.81, 25.91, 25.95, 26.02, 26.76, 28.12, 28.48, 29.17, 29.23, 29.60, 29.65, 29.71, 29.93, 31.36, 33.46, 35.04, 40.46, 42.54, 47.54, 48.80, 53.56, 54.73, 60.06, 60.17, 67.85, 70.61, 70.74, 74.62, 102.88, 107.66, 107.81, 126.15, 127.03, 128.63, 129.00, 129.97, 136.56, 137.34, 157.84, 160.39, 170.96, 172.53, 175.12; HRMS (ESI) *m*/*z*: [M + H]^+^ calcd for C_56_H_88_O_9_N_4_Cl, 995.6234; found, 995.6230.

##### *tert*-Butyl ((*S*)-1-(((*S*)-2-((2*S*,4*S*)-4-(3-((5-Aminopentyl)oxy)phenoxy)-2-(((*R*)-1,2,3,4-tetrahydronaphthalen-1-yl)carbamoyl)pyrrolidin-1-yl)-1-cyclohexyl-2-oxoethyl)amino)-1-oxopropan-2-yl)(methyl)carbamate (**99**)

This compound was prepared using general procedure **VI** and **91** (167 mg, 0.21 mmol). The crude product was used in the next step without further purification. *R*_f_ = 0.35 (CH_2_Cl_2_/MeOH/NH_4_OH 9:1:0.1); HRMS (ESI) *m*/*z*: [M + H]^+^ calcd for C_43_H_64_O_7_N_5_, 762.4800; found, 762.4781.

##### *tert*-Butyl ((*S*)-1-(((*S*)-2-((2*S*,4*S*)-4-(3-((8-Aminooctyl)oxy)phenoxy)-2-(((*R*)-1,2,3,4-tetrahydronaphthalen-1-yl)carbamoyl)pyrrolidin-1-yl)-1-cyclohexyl-2-oxoethyl)amino)-1-oxopropan-2-yl)(methyl)carbamate (**100**)

This compound was prepared using general procedure **VI** and **92** (200 mg, 0.24 mmol). The crude product was used in the next step without further purification. *R*_f_ = 0.12 (CH_2_Cl_2_/MeOH/NH_4_OH 9:1:0.1); HRMS (ESI) *m*/*z*: [M + H]^+^ calcd for C_46_H_70_O_7_N_5_, 804.5270; found, 804.5247.

##### *tert*-Butyl ((*S*)-1-(((*S*)-2-((2*S*,4*S*)-4-(3-(4-(4-Aminobutoxy)butoxy)phenoxy)-2-(((*R*)-1,2,3,4-tetrahydronaphthalen-1-yl)carbamoyl)pyrrolidin-1-yl)-1-cyclohexyl-2-oxoethyl)amino)-1-oxopropan-2-yl)(methyl)carbamate (**101**)

This compound was prepared using general procedure **VI** and **93** (160 mg, 0.19 mmol). The crude product was used in the next step without further purification. *R*_f_ = 0.25 (CH_2_Cl_2_/MeOH/NH_4_OH 9:1:0.1); HRMS (ESI) *m*/*z*: [M + H]^+^ calcd for C_46_H_70_O_8_N_5_, 820.5219; found, 820.5204.

##### *tert*-Butyl ((*S*)-1-(((*S*)-2-((2*S*,4*S*)-4-(3-(2-(2-(2-Aminoethoxy)ethoxy)ethoxy)phenoxy)-2-(((*R*)-1,2,3,4-tetrahydronaphthalen-1-yl)carbamoyl)pyrrolidin-1-yl)-1-cyclohexyl-2-oxoethyl)amino)-1-oxopropan-2-yl)(methyl)carbamate (**102**)

This compound was prepared using general procedure **VI** and **94** (137 mg, 0.17 mmol). The crude product was used in the next step without further purification. *R*_f_ = 0.18 (CH_2_Cl_2_/MeOH/NH_4_OH 9:1:0.1); HRMS (ESI) *m*/*z*: [M + H]^+^ calcd for C_44_H_66_O_9_N_5_, 808.4855; found, 808.4837.

##### *tert*-Butyl ((*S*)-1-(((*S*)-2-((2*S*,4*S*)-4-(3-(2-(2-(2-(2-Aminoethoxy)ethoxy)ethoxy)ethoxy)phenoxy)-2-(((*R*)-1,2,3,4-tetrahydronaphthalen-1-yl)carbamoyl)pyrrolidin-1-yl)-1-cyclohexyl-2-oxoethyl)amino)-1-oxopropan-2-yl)(methyl)carbamate (**103**)

This compound was prepared using general procedure **VI** and **95** (180 mg, 0.21 mmol). The crude product was used in the next step without further purification. *R*_f_ = 0.35 (CH_2_Cl_2_/MeOH/NH_4_OH 9:1:0.1); HRMS (ESI) *m*/*z*: [M + H]^+^ calcd for C_46_H_70_O_10_N_5_, 852.5117; found, 852.5097.

##### *tert*-Butyl ((*S*)-1-(((*S*)-2-((2*S*,4*S*)-4-(3-(2-((6-((6-Aminohexyl)oxy)hexyl)oxy)ethoxy)phenoxy)-2-(((*R*)-1,2,3,4-tetrahydronaphthalen-1-yl)carbamoyl)pyrrolidin-1-yl)-1-cyclohexyl-2-oxoethyl)amino)-1-oxopropan-2-yl)(methyl)carbamate (**104**)

This compound was prepared using general procedure **VI** and **96** (150 mg, 0.16 mmol). The crude product was used in the next step without further purification. *R*_f_ = 0.18 (CH_2_Cl_2_/MeOH/NH_4_OH 9:1:0.1); HRMS (ESI) *m*/*z*: [M + H]^+^ calcd for C_52_H_82_O_9_N_5_, 920.6107; found, 920.6074.

##### *tert*-Butyl ((*S*)-1-(((*S*)-2-((2*S*,4*S*)-4-(3-((5-((5-((6-Aminohexyl)oxy)pentyl)oxy)pentyl)oxy)phenoxy)-2-(((*R*)-1,2,3,4-tetrahydronaphthalen-1-yl)carbamoyl)pyrrolidin-1-yl)-1-cyclohexyl-2-oxoethyl)amino)-1-oxopropan-2-yl)(methyl)carbamate (**105**)

This compound was prepared using general procedure **VI** and **97** (190 mg, 0.28 mmol). The crude product was used in the next step without further purification. *R*_f_ = 0.22 (CH_2_Cl_2_/MeOH/NH_4_OH 9:1:0.1); HRMS (ESI) *m*/*z*: [M + H]^+^ calcd for C_54_H_86_O_9_N_5_, 948.6420; found, 948.6409.

##### *tert*-Butyl ((*S*)-1-(((*S*)-2-((2*S*,4*S*)-4-(3-((6-((6-((6-Aminohexyl)oxy)hexyl)oxy)hexyl)oxy)phenoxy)-2-(((*R*)-1,2,3,4-tetrahydronaphthalen-1-yl)carbamoyl)pyrrolidin-1-yl)-1-cyclohexyl-2-oxoethyl)amino)-1-oxopropan-2-yl)(methyl)carbamate (**106**)

This compound was prepared using general procedure **VI** and **98** (230 mg, 0.23 mmol). The crude product was used in the next step without further purification. *R*_f_ = 0.40 (CH_2_Cl_2_/MeOH/NH_4_OH 9:1:0.1); HRMS (ESI) *m*/*z*: [M + H]^+^ calcd for C_56_H_90_O_9_N_5_, 976.6733; found, 976.6696.

#### Synthesis of Boc-Protected and Final PROTACs

##### *tert*-Butyl ((*S*)-1-(((*S*)-1-Cyclohexyl-2-((2*S*,4*S*)-4-(3-((5-(((*S*)-1-((2*S*,4*R*)-4-hydroxy-2-((4-(4-methylthiazol-5-yl)benzyl)carbamoyl)pyrrolidin-1-yl)-3,3-dimethyl-1-oxobutan-2-yl)amino)-5-oxopentyl)oxy)phenoxy)-2-(((*R*)-1,2,3,4-tetrahydronaphthalen-1-yl)carbamoyl)pyrrolidin-1-yl)-2-oxoethyl)amino)-1-oxopropan-2-yl)(methyl)carbamate (**107**)

The IAP ligand–linker conjugate **74** (147 mg, 0.17 mmol) was dissolved in dry EtOAc (10 mL) and treated with 10% Pd/C (10% w/w). The reaction mixture was stirred under H_2_ (1 atm, balloon) for 18 h. The mixture was filtered through Celite, and the filtrate was concentrated. The oily residue was dissolved in dry DMF (5 mL), and DIPEA (59 μL, 0.34 mmol) and HATU (71 mg, 0.187 mmol) were added. After stirring at rt for 5 min, the VHL ligand **68** (90 mg, 0.20 mmol) in dry DMF (2.5 mL) and DIPEA (59 μL, 0.34 mmol) was added. The combined mixture was stirred at rt for 16 h. Subsequently, it was quenched with half-saturated brine (50 mL) and extracted with CH_2_Cl_2_ (3 × 50 mL). The combined organic layers were washed with saturated NH_4_Cl solution, 5% LiCl solution, and brine (each 50 mL); dried over Na_2_SO_4_; filtered; and evaporated. The crude product was purified by column chromatography (CH_2_Cl_2_/MeOH 29:1) to give a colorless resin. Yield (64 mg, 32%); *R*_f_ = 0.22 (CH_2_Cl_2_/MeOH 9:1). Due to the presence of the *N*-Boc protecting group resulting in an additional set of rotamers, NMR data is only provided for the deprotected final PROTAC. HRMS (ESI) *m*/*z*: [M + H]^+^ calcd for C_65_H_89_N_8_O_11_S, 1189.6366; found, 1189.6333.

##### *tert*-Butyl ((*S*)-1-(((*S*)-1-Cyclohexyl-2-((2*S*,4*S*)-4-(3-((8-(((*S*)-1-((2*S*,4*R*)-4-hydroxy-2-((4-(4-methylthiazol-5-yl)benzyl)carbamoyl)pyrrolidin-1-yl)-3,3-dimethyl-1-oxobutan-2-yl)amino)-8-oxooctyl)oxy)phenoxy)-2-(((*R*)-1,2,3,4-tetrahydronaphthalen-1-yl)carbamoyl)pyrrolidin-1-yl)-2-oxoethyl)amino)-1-oxopropan-2-yl)(methyl)carbamate (**108**)

This compound was prepared using general procedure **II**, VHL1 ligand–linker conjugate **75** (177 mg, 0.30 mmol), and IAP ligand **65**. The crude product was purified by column chromatography (CH_2_Cl_2_/MeOH 29:1) to give a colorless solid. Yield (206 mg, 56%); *R*_f_ = 0.26 (CH_2_Cl_2_/MeOH 19:1); mp 112–116 °C. Due to the presence of the *N*-Boc protecting group resulting in an additional set of rotamers, NMR data is only provided for the deprotected final PROTAC. HRMS (ESI) *m*/*z*: [M + H]^+^ calcd for C_68_H_95_N_8_O_11_S, 1231.6836; found, 1231.6679.

##### *tert*-Butyl ((*S*)-1-(((*S*)-1-Cyclohexyl-2-((2*S*,4*S*)-4-(3-(4-(4-(((*S*)-1-((2*S*,4*R*)-4-hydroxy-2-((4-(4-methylthiazol-5-yl)benzyl)carbamoyl)pyrrolidin-1-yl)-3,3-dimethyl-1-oxobutan-2-yl)amino)-4-oxobutoxy)butoxy)phenoxy)-2-(((*R*)-1,2,3,4-tetrahydronaphthalen-1-yl)carbamoyl)pyrrolidin-1-yl)-2-oxoethyl)amino)-1-oxopropan-2-yl)(methyl)carbamate (**109**)

This compound was prepared using general procedure **II**, VHL1 ligand–linker conjugate **76** (182 mg, 0.30 mmol), and IAP ligand **65**. The crude product was purified by column chromatography (CH_2_Cl_2_/MeOH 19:1) to give a colorless semi-solid. Yield (202 mg, 54%); *R*_f_ = 0.25 (CH_2_Cl_2_/MeOH 19:1). Due to the presence of the *N*-Boc protecting group resulting in an additional set of rotamers, NMR data is only provided for the deprotected final PROTAC. HRMS (ESI) *m*/*z*: [M + H]^+^ calcd for C_68_H_95_N_8_O_12_S, 1247.6785; found, 1247.6775.

##### *tert*-Butyl ((*S*)-1-(((*S*)-1-Cyclohexyl-2-((2*S*,4*S*)-4-(3-(2-(2-(2-(((*S*)-1-((2*S*,4*R*)-4-hydroxy-2-((4-(4-methylthiazol-5-yl)benzyl)carbamoyl)pyrrolidin-1-yl)-3,3-dimethyl-1-oxobutan-2-yl)amino)-2-oxoethoxy)ethoxy)ethoxy)phenoxy)-2-(((*R*)-1,2,3,4-tetrahydronaphthalen-1-yl)carbamoyl)pyrrolidin-1-yl)-2-oxoethyl)amino)-1-oxopropan-2-yl)(methyl)carbamate (**110**)

This compound was prepared using general procedure **II**, VHL1 ligand–linker conjugate **77** (179 mg, 0.30 mmol), and IAP ligand **65**. The crude product was purified by column chromatography (CH_2_Cl_2_/MeOH 19:1) to give a colorless solid. Yield (263 mg, 71%); *R*_f_ = 0.25 (CH_2_Cl_2_/MeOH 19:1); mp 96–100 °C. Due to the presence of the *N*-Boc protecting group resulting in an additional set of rotamers, NMR data is only provided for the deprotected final PROTAC. HRMS (ESI) *m*/*z*: [M + H]^+^ calcd for C_66_H_91_N_8_O_13_S, 1235.6378; found, 1235.6421.

##### *tert*-Butyl ((*S*)-1-(((*S*)-1-Cyclohexyl-2-((2*S*,4*S*)-4-(3-(((*S*)-13-((2*S*,4*R*)-4-hydroxy-2-((4-(4-methylthiazol-5-yl)benzyl)carbamoyl)pyrrolidine-1-carbonyl)-14,14-dimethyl-11-oxo-3,6,9-trioxa-12-azapentadecyl)oxy)phenoxy)-2-(((*R*)-1,2,3,4-tetrahydronaphthalen-1-yl)carbamoyl)pyrrolidin-1-yl)-2-oxoethyl)amino)-1-oxopropan-2-yl)(methyl)carbamate (**111**)

This compound was prepared using general procedure **II**, VHL1 ligand–linker conjugate **78** (192 mg, 0.30 mmol), and IAP ligand **65**. The crude product was purified by column chromatography (CH_2_Cl_2_/MeOH 19:1) to give a colorless semi-solid. Yield (134 mg, 35%); *R*_f_ = 0.21 (CH_2_Cl_2_/MeOH 19:1). Due to the presence of the *N*-Boc protecting group resulting in an additional set of rotamers, NMR data is only provided for the deprotected final PROTAC. HRMS (ESI) *m*/*z*: [M + H]^+^ calcd for C_68_H_95_N_8_O_14_S, 1279.6683; found, 1279.6676.

##### *tert*-Butyl ((*S*)-1-(((*S*)-1-Cyclohexyl-2-((2*S*,4*S*)-4-(3-((6-((6-(2-(((*S*)-1-((2*S*,4*R*)-4-hydroxy-2-((4-(4-methylthiazol-5-yl)benzyl)carbamoyl)pyrrolidin-1-yl)-3,3-dimethyl-1-oxobutan-2-yl)amino)-2-oxoethoxy)hexyl)oxy)hexyl)oxy)phenoxy)-2-(((*R*)-1,2,3,4-tetrahydronaphthalen-1-yl)carbamoyl)pyrrolidin-1-yl)-2-oxoethyl)amino)-1-oxopropan-2-yl)(methyl)carbamate (**112**)

This compound was prepared using general procedure **II**, VHL1 ligand–linker conjugate **79** (212 mg, 0.30 mmol), and IAP ligand **65**. The crude product was purified by column chromatography (CH_2_Cl_2_/MeOH 19:1) to give a colorless solid. Yield (105 mg, 26%); *R*_f_ = 0.15 (CH_2_Cl_2_/MeOH 19:1); mp 70–72 °C. Due to the presence of the *N*-Boc protecting group resulting in an additional set of rotamers, NMR data is only provided for the deprotected final PROTAC. HRMS (ESI) *m*/*z*: [M + H]^+^ calcd for C_74_H_107_N_8_O_13_S, 1347.7673; found, 1347.7704.

##### *tert*-Butyl ((*S*)-1-(((*S*)-1-Cyclohexyl-2-((2*S*,4*S*)-4-(3-((6-((5-((5-(((*S*)-1-((2*S*,4*R*)-4-hydroxy-2-((4-(4-methylthiazol-5-yl)benzyl)carbamoyl)pyrrolidin-1-yl)-3,3-dimethyl-1-oxobutan-2-yl)amino)-5-oxopentyl)oxy)pentyl)oxy)hexyl)oxy)phenoxy)-2-(((*R*)-1,2,3,4-tetrahydronaphthalen-1-yl)carbamoyl)pyrrolidin-1-yl)-2-oxoethyl)amino)-1-oxopropan-2-yl)(methyl)carbamate (**113**)

This compound was prepared using general procedure **II**, VHL1 ligand–linker conjugate **80** (221 mg, 0.30 mmol), and IAP ligand **65**. The crude product was purified by flash chromatography (CH_2_Cl_2_/MeOH 19:1) to give a colorless semi-solid. Yield (235 mg, 57%); *R*_f_ = 0.22 (CH_2_Cl_2_/MeOH 19:1). Due to the presence of the *N*-Boc protecting group resulting in an additional set of rotamers, NMR data is only provided for the deprotected final PROTAC. HRMS (ESI) *m*/*z*: [M + H]^+^ calcd for C_76_H_111_N_8_O_13_S, 1375.7986; found, 1375.7981.

##### *tert*-Butyl ((*S*)-1-(((*S*)-1-Cyclohexyl-2-((2*S*,4*S*)-4-(3-((6-((5-((5-(((*S*)-1-((2*S*,4*R*)-4-hydroxy-2-((4-(4-methylthiazol-5-yl)benzyl)carbamoyl)pyrrolidin-1-yl)-3,3-dimethyl-1-oxobutan-2-yl)amino)-5-oxopentyl)oxy)pentyl)oxy)hexyl)oxy)phenoxy)-2-(((*R*)-1,2,3,4-tetrahydronaphthalen-1-yl)carbamoyl)pyrrolidin-1-yl)-2-oxoethyl)amino)-1-oxopropan-2-yl)(methyl)carbamate (**114**)

This compound was prepared using general procedure **II**, VHL1 ligand–linker conjugate **81** (229 mg, 0.30 mmol), and IAP ligand **65**. The crude product was purified by flash chromatography (CH_2_Cl_2_/MeOH 19:1) to give a colorless solid. Yield (219 mg, 52%); *R*_f_ = 0.23 (CH_2_Cl_2_/MeOH 19:1); mp 74–76 °C. Due to the presence of the *N*-Boc protecting group resulting in an additional set of rotamers, NMR data is only provided for the deprotected final PROTAC. HRMS (ESI) *m*/*z*: [M + H]^+^ calcd for C_78_H_115_N_8_O_13_S, 1403.8299; found, 1403.8300.

##### *tert*-Butyl ((*S*)-1-(((*S*)-1-Cyclohexyl-2-((2*S*,4*S*)-4-(3-((5-(((*S*)-1-((2*S*,4*R*)-4-hydroxy-2-(((*S*)-1-(4-(4-methylthiazol-5-yl)phenyl)ethyl)carbamoyl)pyrrolidin-1-yl)-3,3-dimethyl-1-oxobutan-2-yl)amino)-5-oxopentyl)oxy)phenoxy)-2-(((*R*)-1,2,3,4-tetrahydronaphthalen-1-yl)carbamoyl)pyrrolidin-1-yl)-2-oxoethyl)amino)-1-oxopropan-2-yl)(methyl)carbamate (**115** = **10d**)

This compound was synthesized analogously to **107** but using VHL ligand **69** (92 mg, 0.21 mmol). A colorless solid was obtained after flash chromatography (gradient from 0 to 5% MeOH in CH_2_Cl_2_). Yield (143 mg, 70%); *R*_f_ = 0.23 (CH_2_Cl_2_/MeOH 19:1); mp 134–138 °C; ^1^H NMR (600 MHz, DMSO-*d*_6_): δ 0.93 (s, 9H), 1.03–1.10 (m, 1H), 1.18 (s, 4H), 1.24 (s, 1H), 1.34–1.41 (m, 16H), 1.56–1.71 (m, 6H), 1.72–1.83 (m, 2H), 1.96–2.03 (m, 1H), 2.05–2.11 (m, 1H), 2.14–2.22 (m, 1H), 2.28–2.35 (m, 1H), 2.44 (s, 3H), 2.50–2.56 (m, 1H), 2.64–2.75 (m, 7H), 3.56–3.64 (m, 3H), 3.85–3.93 (m, 3H), 4.21–4.30 (m, 3H), 4.31 (t, *J* = 7.8 Hz, 1H), 4.39–4.47 (m, 2H), 4.52 (d, *J* = 9.3 Hz, 1H), 4.87–4.95 (m, 3H), 5.00–5.06 (m, 1H), 5.07 (d, *J* = 3.5 Hz, 1H), 6.44 (t, *J* = 2.4 Hz, 1H), 6.44–6.55 (m, 2H), 7.00–7.19 (m, 5H), 7.24 (d, *J* = 7.5 Hz, 1H), 7.35–7.44 (m, 5H), 7.78–7.85 (m, 2H), 8.33 (d, *J* = 7.8 Hz, 1H), 8.97 (s, 1H); LC–MS (ESI) 99% purity, *m*/*z*: [M + H]^+^ calcd for C_66_H_91_N_8_O_11_S, 1203.65; found, 1204.0; HRMS (ESI) *m*/*z*: [M + H]^+^ calcd for C_66_H_91_N_8_O_11_S, 1203.6523; found, 1203.6508.

##### *tert*-Butyl ((*R*)-1-(((*S*)-1-Cyclohexyl-2-((2*S*,4*S*)-4-(3-((5-(((*S*)-1-((2*S*,4*R*)-4-hydroxy-2-(((*S*)-1-(4-(4-methylthiazol-5-yl)phenyl)ethyl)carbamoyl)pyrrolidin-1-yl)-3,3-dimethyl-1-oxobutan-2-yl)amino)-5-oxopentyl)oxy)phenoxy)-2-(((*R*)-1,2,3,4-tetrahydronaphthalen-1-yl)carbamoyl)pyrrolidin-1-yl)-2-oxoethyl)amino)-1-oxopropan-2-yl)(methyl)carbamate (**116**)

This compound was synthesized analogously to **107** but using IAP ligand–linker conjugate **82** (147 mg, 0.17 mmol). A colorless solid was obtained after flash chromatography (gradient from 0 to 8% MeOH in CH_2_Cl_2_). Yield (133 mg, 66%); *R*_f_ = 0.22 (CH_2_Cl_2_/MeOH 19:1); mp 132–136 °C. Due to the presence of the *N*-Boc protecting group resulting in an additional set of rotamers, NMR data is only provided for the deprotected final PROTAC. HRMS (ESI) *m*/*z*: [M + H]^+^ calcd for C_65_H_89_N_8_O_11_S, 1189.6366; found, 1189.6315.

##### *tert*-Butyl ((*S*)-1-(((*S*)-1-Cyclohexyl-2-((2*S*,4*S*)-4-(3-((5-(((*S*)-1-((2*S*,4*S*)-4-hydroxy-2-((4-(4-methylthiazol-5-yl)benzyl)carbamoyl)pyrrolidin-1-yl)-3,3-dimethyl-1-oxobutan-2-yl)amino)-5-oxopentyl)oxy)phenoxy)-2-(((*R*)-1,2,3,4-tetrahydronaphthalen-1-yl)carbamoyl)pyrrolidin-1-yl)-2-oxoethyl)amino)-1-oxopropan-2-yl)(methyl)carbamate (**117**)

Compound **74** (225 mg, 0.26 mmol) was dissolved in dry MeOH (8 mL) and treated with 10% Pd/C (45 mg, 20% w/w). The reaction mixture was stirred under H_2_ (1 atm, balloon) for 2 h. The mixture was filtered through Celite and washed with MeOH, and the filtrate was concentrated. The product was dissolved in dry DMF (5 mL) along with HATU (109 mg, 0.286 mmol) and DIPEA (101 mg, 133 μL) 0.78 mmol. A solution of **70** (121 mg, 0.28 mmol) in dry DMF (5 mL) was added, followed by stirring of the mixture at rt for 18 h. The volatiles were then evaporated, and the crude product was purified by column chromatography (CH_2_Cl_2_/MeOH 20:1) to give an off-white resin. Yield (275 mg, 89%); *R*_f_ = 0.25 (CH_2_Cl_2_/MeOH 9:1). Due to the presence of the *N*-Boc protecting group resulting in an additional set of rotamers, NMR data is only provided for the deprotected final PROTAC. HRMS (ESI) *m*/*z*: [M + H]^+^ calcd for C_65_H_89_O_11_N_8_S, 1189.6366; found, 1189.6350.

##### (2*S*,4*S*)-1-((*S*)-2-Cyclohexyl-2-((*S*)-2-(methylamino)propanamido)acetyl)-4-(3-((5-(((*S*)-1-((2*S*,4*R*)-4-hydroxy-2-((4-(4-methylthiazol-5-yl)benzyl)carbamoyl)pyrrolidin-1-yl)-3,3-dimethyl-1-oxobutan-2-yl)amino)-5-oxopentyl)oxy)phenoxy)-*N*-((*R*)-1,2,3,4-tetrahydronaphthalen-1-yl)pyrrolidine-2-carboxamide (PROTAC **1**)

This compound was prepared using general procedure **VII** and PROTAC precursor **107** (60 mg, 50 μmol). After filtration of the solid material, the crude product was purified by column chromatography (CH_2_Cl_2_/MeOH + 7 N NH_3_ 15:1) to give a colorless solid. Yield (24 mg, 43%); *R*_f_ = 0.14 (CH_2_Cl_2_/MeOH + 7 N NH_3_ 19:1); mp 76–80 °C; ^1^H NMR (600 MHz, DMSO-*d*_6_): δ 0.92 (s, 9H), 0.94–1.14 (m, 7H), 1.52–1.83 (m, 14H), 1.86–1.93 (m, 1H), 1.96–2.14 (m, 2H), 2.15 (s, 3H), 2.16–2.21 (m, 2H), 2.28–2.40 (m, 1H), 2.42 (s, 3H), 2.50–2.57 (m, 1H), 2.62–2.74 (m, 2H), 2.89–2.98 (m, 1H), 3.46–3.69 (m, 4H), 3.84–3.93 (m, 2H), 4.13–4.60 (m, 8H), 4.87–4.93 (m, 1H), 4.98–5.06 (m, 1H), 5.13 (d, *J* = 3.6 Hz, 1H), 6.38–6.54 (m, 3H), 7.01–7.18 (m, 4H), 7.23 (d, *J* = 7.5 Hz, 1H), 7.32–7.44 (m, 4H), 7.80 (d, *J* = 8.6 Hz, 1H), 7.86 (d, *J* = 9.4 Hz, 1H), 7.92 (d, *J* = 8.6 Hz, 1H), 8.53 (t, *J* = 6.1 Hz, 1H), 8.95 (s, 1H); ^13^C NMR (151 MHz, DMSO-*d*_6_): δ 16.15, 19.27, 20.14, 22.35, 25.66, 25.85, 26.02, 26.61, 28.03, 28.50, 28.97, 29.31, 29.91, 34.46, 34.65, 34.77, 35.46, 38.15, 40.23, 41.92, 46.89, 52.26, 54.63, 56.59, 56.63, 58.79, 58.96, 59.31, 67.42, 69.12, 75.17, 102.62, 107.61, 107.87, 125.99, 127.00, 127.68, 128.58, 128.89, 128.92, 129.88, 130.30, 131.43, 137.26, 137.41, 139.72, 147.96, 151.70, 158.32, 160.16, 169.97, 170.17, 170.70, 172.23, 172.26, 174.73; LC–MS (ESI) 95% purity, *m*/*z*: [M + H]^+^ calcd for C_60_H_81_N_8_O_9_S, 1089.58; found, 1089.8; HRMS (ESI) *m*/*z*: [M + H]^+^ calcd for C_60_H_81_N_8_O_9_S, 1089.5842; found, 1089.5796.

##### (2*S*,4*S*)-1-((*S*)-2-Cyclohexyl-2-((*S*)-2-(methylamino)propanamido)acetyl)-4-(3-((8-(((*S*)-1-((2*S*,4*R*)-4-hydroxy-2-((4-(4-methylthiazol-5-yl)benzyl)carbamoyl)pyrrolidin-1-yl)-3,3-dimethyl-1-oxobutan-2-yl)amino)-8-oxooctyl)oxy)phenoxy)-*N*-((*R*)-1,2,3,4-tetrahydronaphthalen-1-yl)pyrrolidine-2-carboxamide (PROTAC **2**)

This compound was prepared using general procedure **VII** and PROTAC precursor **108** (123 mg, 100 μmol). The product possessed sufficient purity after filtration. A colorless solid was obtained. Yield (93 mg, 80%); mp 162–166 °C; ^1^H NMR (600 MHz, DMSO-*d*_6_): δ 0.92 (s, 9H), 1.03–1.19 (m, 3H), 1.21–1.47 (m, 10H), 1.48–1.82 (m, 8H), 1.85–1.91 (m, 1H), 1.99–2.14 (m, 3H), 2.22–2.29 (m, 1H), 2.42–2.47 (m, 7H), 2.63–2.73 (m, 3H), 3.60–3.68 (m, 3H), 3.81–3.93 (m, 4H), 4.17–4.24 (m, 2H), 4.31–4.48 (m, 6H), 4.53 (d, *J* = 9.4 Hz, 1H), 4.87–4.94 (m, 1H), 5.00–5.09 (m, 1H), 6.39–6.55 (m, 4H), 7.02–7.18 (m, 6H), 7.22 (d, *J* = 7.5 Hz, 1H), 7.35–7.44 (m, 6H), 7.84 (d, *J* = 9.4 Hz, 1H), 7.89 (d, *J* = 8.6 Hz, 1H), 8.57 (t, *J* = 6.1 Hz, 1H), 8.75 (d, *J* = 8.1 Hz, 1H), 8.80–8.88 (m, 1H), 9.02 (s, 1H); ^13^C NMR (151 MHz, DMSO-*d*_6_): δ 16.00, 16.03, 20.10, 25.58, 25.66, 25.83, 25.91, 26.58, 28.09, 28.71, 28.83, 28.94, 28.98, 29.88, 30.92, 34.68, 35.04, 35.42, 38.16, 40.24, 41.83, 46.81, 52.31, 55.64, 56.03, 56.46, 56.54, 58.76, 58.89, 67.60, 69.05, 75.12, 102.71, 107.52, 107.73, 125.94, 126.94, 127.63, 128.53, 128.84, 128.89, 129.69, 130.26, 131.52, 137.22, 137.40, 139.80, 147.63, 151.85, 158.24, 160.14, 168.72, 169.90, 169.93, 169.96, 172.17, 172.30; LC–MS (ESI) 98% purity, *m*/*z*: [M + H]^+^ calcd for C_63_H_87_N_8_O_9_S, 1131.63; found, 1131.8; HRMS (ESI) *m*/*z*: [M + H]^+^ calcd for C_63_H_87_N_8_O_9_S, 1131.6311; found, 1131.6272.

##### (2*S*,4*S*)-1-((*S*)-2-Cyclohexyl-2-((*S*)-2-(methylamino)propanamido)acetyl)-4-(3-(4-(4-(((*S*)-1-((2*S*,4*R*)-4-hydroxy-2-((4-(4-methylthiazol-5-yl)benzyl)carbamoyl)pyrrolidin-1-yl)-3,3-dimethyl-1-oxobutan-2-yl)amino)-4-oxobutoxy)butoxy)phenoxy)-*N*-((*R*)-1,2,3,4-tetrahydronaphthalen-1-yl)pyrrolidine-2-carboxamide (PROTAC **3**)

This compound was prepared using general procedure **VII** and PROTAC precursor **109** (80 mg, 64 μmol). After filtration of the solid material, the crude product was purified by column chromatography (CH_2_Cl_2_/MeOH + 7 N NH_3_ 15:1) to give a colorless solid. Yield (43 mg, 58%); *R*_f_ = 0.33 (CH_2_Cl_2_/MeOH + 7 N NH_3_ 15:1); mp 84–86 °C; ^1^H NMR (600 MHz, DMSO-*d*_6_): δ 0.91 (s, 9H), 0.95–1.19 (m, 7H), 1.49–1.83 (m, 17H), 1.85–1.93 (m, 1H), 1.96–2.11 (m, 2H), 2.15 (s, 3H), 2.16–2.34 (m, 3H), 2.42 (s, 3H), 2.49–2.56 (m, 1H), 2.62–2.74 (m, 2H), 2.88–2.99 (m, 1H), 3.28–3.35 (m, 2H), 3.44–3.51 (m, 2H), 3.59–3.68 (m, 3H), 3.85–3.94 (m, 2H), 4.17–4.28 (m, 2H), 4.31–4.49 (m, 5H), 4.52 (d, *J* = 9.4 Hz, 1H), 4.87–5.06 (m, 2H), 5.12 (d, *J* = 3.6 Hz, 1H), 6.39–6.44 (m, 1H), 6.44–6.54 (m, 2H), 7.04–7.18 (m, 4H), 7.23 (d, *J* = 7.4 Hz, 1H), 7.34–7.44 (m, 4H), 7.75–7.85 (m, 2H), 7.92 (d, *J* = 8.6 Hz, 1H), 8.52 (t, *J* = 6.1 Hz, 1H), 8.95 (s, 1H); ^13^C NMR (151 MHz, DMSO-*d*_6_): δ 16.13, 19.25, 20.13, 25.63, 25.80, 25.83, 26.01, 26.09, 26.58, 28.01, 28.95, 29.28, 29.89, 31.89, 34.44, 34.62, 35.44, 38.13, 40.22, 41.89, 46.87, 52.23, 54.60, 56.55, 56.64, 58.75, 58.94, 59.30, 67.52, 69.10, 69.68, 69.80, 75.16, 102.62, 107.57, 107.87, 125.97, 126.97, 127.66, 128.55, 128.87, 128.90, 129.86, 130.28, 131.40, 137.24, 137.39, 139.70, 147.94, 151.67, 158.29, 160.13, 169.92, 170.13, 170.68, 172.13, 172.21, 174.71; LC–MS (ESI) 98% purity, *m*/*z*: [M + H]^+^ calcd for C_63_H_87_N_8_O_10_S, 1147.63; found, 1148.0; HRMS (ESI) *m*/*z*: [M + H]^+^ calcd for C_63_H_87_N_8_O_10_S, 1147.6260; found, 1147.6232.

##### (2*S*,4*S*)-1-((*S*)-2-Cyclohexyl-2-((*S*)-2-(methylamino)propanamido)acetyl)-4-(3-(2-(2-(2-(((*S*)-1-((2*S*,4*R*)-4-hydroxy-2-((4-(4-methylthiazol-5-yl)benzyl)carbamoyl)pyrrolidin-1-yl)-3,3-dimethyl-1-oxobutan-2-yl)amino)-2-oxoethoxy)ethoxy)ethoxy)phenoxy)-*N*-((*R*)-1,2,3,4-tetrahydronaphthalen-1-yl)pyrrolidine-2-carboxamide (PROTAC **4**)

This compound was prepared using general procedure **VII** and PROTAC precursor **110** (78 mg, 63 μmol). The product possessed sufficient purity after filtration. A colorless solid was obtained. Yield (71 mg, 47%); mp 164–166 °C; ^1^H NMR (600 MHz, DMSO-*d*_6_): δ 0.92 (s, 9H), 0.93–1.17 (m, 7H), 1.33 (d, *J* = 6.9 Hz, 3H), 1.56–1.82 (m, 6H), 1.84–1.93 (m, 1H), 2.01–2.09 (m, 2H), 2.40–2.48 (m, 7H), 2.49–2.56 (m, 1H), 2.63–2.75 (m, 2H), 3.57–3.68 (m, 7H), 3.69–3.81 (m, 2H), 3.81–3.96 (m, 1H), 3.97 (s, 2H), 4.01–4.09 (m, 2H), 4.19–4.30 (m, 2H), 4.31–4.46 (m, 5H), 4.55 (d, *J* = 9.6 Hz, 1H), 4.87–4.94 (m, 1H), 5.01 (p, *J* = 5.5 Hz, 1H), 6.39–6.56 (m, 3H), 7.02–7.17 (m, 5H), 7.22 (d, *J* = 7.6 Hz, 1H), 7.35–7.45 (m, 6H), 7.90 (d, *J* = 8.5 Hz, 1H), 8.58 (t, *J* = 6.0 Hz, 1H), 8.73 (d, *J* = 8.1 Hz, 1H), 8.78–8.86 (m, 1H), 8.97 (s, 1H); ^13^C NMR (151 MHz, DMSO-*d*_6_): δ 15.92, 16.00, 20.05, 25.62, 25.78, 25.87, 26.34, 26.44, 28.04, 28.89, 28.93, 29.84, 30.88, 34.61, 35.84, 38.06, 40.24, 41.84, 46.77, 52.23, 55.59, 55.88, 56.01, 56.71, 58.67, 58.90, 67.25, 69.01, 69.13, 69.79, 69.89, 70.60, 75.01, 102.57, 107.38, 108.07, 125.88, 126.88, 127.61, 128.47, 128.83, 129.79, 130.22, 131.36, 137.17, 137.37, 139.62, 147.75, 151.65, 158.22, 159.84, 168.66, 168.75, 169.31, 169.84, 169.93, 171.92; LC–MS (ESI) 96% purity, *m*/*z*: [M + H]^+^ calcd for C_61_H_82_N_8_O_11_S, 1131.59; found, 1135.7; HRMS (ESI) *m*/*z*: [M + H]^+^ calcd for C_61_H_82_N_8_O_11_S, 1135.5897; found, 1135.5877.

##### (2*S*,4*R*)-1-((*S*)-2-(*tert*-Butyl)-14-(3-(((3*S*,5*S*)-1-((*S*)-2-cyclohexyl-2-((*S*)-2-(methylamino)propanamido)acetyl)-5-(((*R*)-1,2,3,4-tetrahydronaphthalen-1-yl)carbamoyl)pyrrolidin-3-yl)oxy)phenoxy)-4-oxo-6,9,12-trioxa-3-azatetradecanoyl)-4-hydroxy-*N*-(4-(4-methylthiazol-5-yl)benzyl)pyrrolidine-2-carboxamide (PROTAC **5**)

This compound was prepared using general procedure **VII** and PROTAC precursor **111** (80 mg, 63 μmol). After filtration of the solid material, the crude product was purified by column chromatography (CH_2_Cl_2_/MeOH + 7 N NH_3_ 19:1) to give a colorless solid. Yield (35 mg, 47%); *R*_f_ = 0.17 (CH_2_Cl_2_/MeOH + 7 N NH_3_ 19:1); mp 84–86 °C; ^1^H NMR (600 MHz, DMSO-*d*_6_): δ 0.92 (s, 9H), 1.03–1.11 (m, 6H), 1.51–1.69 (m, 7H), 1.71–1.81 (m, 3H), 1.85–1.92 (m, 1H), 2.01–2.09 (m, 2H), 2.15 (s, 2H), 2.18 (s, 1H), 2.42 (s, 3H), 2.51–2.54 (m, 1H), 2.63–2.70 (m, 2H), 2.87–3.01 (m, 1H), 3.55–3.71 (m, 12H), 3.93–4.02 (m, 4H), 4.20–4.29 (m, 3H), 4.32–4.46 (m, 6H), 4.54 (d, *J* = 9.5 Hz, 1H), 4.90 (d, *J* = 6.5 Hz, 1H), 5.01 (t, *J* = 5.4 Hz, 1H), 5.13–5.17 (m, 1H), 6.42–6.53 (m, 3H), 7.02–7.18 (m, 5H), 7.22 (d, *J* = 7.6 Hz, 1H), 7.38 (d, *J* = 7.6 Hz, 6H), 7.83 (d, *J* = 8.5 Hz, 1H), 7.91 (d, *J* = 8.5 Hz, 1H), 8.56 (t, *J* = 6.2 Hz, 1H), 8.94 (s, 1H); ^13^C NMR (151 MHz, DMSO-*d*_6_): δ 16.10, 19.27, 20.12, 25.64, 25.83, 26.00, 26.38, 26.48, 28.00, 28.95, 29.26, 29.89, 34.45, 34.61, 35.92, 38.09, 40.22, 41.92, 46.86, 52.22, 54.59, 55.94, 56.77, 58.72, 58.98, 59.31, 67.34, 69.10, 69.12, 69.81, 69.83, 70.09, 70.14, 70.70, 75.11, 102.48, 107.54, 108.13, 125.97, 126.97, 127.69, 128.56, 128.90, 129.91, 130.31, 131.37, 137.24, 137.40, 139.63, 147.96, 151.66, 158.30, 159.93, 168.88, 169.38, 170.13, 170.67, 172.03; LC–MS (ESI) 98% purity, *m*/*z*: [M + H]^+^ calcd for C_63_H_87_N_8_O_12_S, 1179.62; found, 1180.0; HRMS (ESI) *m*/*z*: [M + H]^+^ calcd for C_63_H_87_N_8_O_12_S, 1179.6159; found, 1179.6122.

##### (2*S*,4*S*)-1-((*S*)-2-Cyclohexyl-2-((*S*)-2-(methylamino)propanamido)acetyl)-4-(3-((6-((6-(2-(((*S*)-1-((2*S*,4*R*)-4-hydroxy-2-((4-(4-methylthiazol-5-yl)benzyl)carbamoyl)pyrrolidin-1-yl)-3,3-dimethyl-1-oxobutan-2-yl)amino)-2-oxoethoxy)hexyl)oxy)hexyl)oxy)phenoxy)-*N*-((*R*)-1,2,3,4-tetrahydronaphthalen-1-yl)pyrrolidine-2-carboxamide (PROTAC **6**)

This compound was prepared using general procedure **VII** and PROTAC precursor **112** (101 mg, 75 μmol). The product possessed sufficient purity after filtration. A colorless solid was obtained. Yield (73 mg, 75%); mp 142–144 °C; ^1^H NMR (600 MHz, DMSO-*d*_6_): δ 0.92 (s, 9H), 0.95–1.15 (m, 5H), 1.26–1.92 (m, 30H), 2.02–2.10 (m, 2H), 2.41–2.47 (m, 6H), 2.50–2.57 (m, 1H), 2.63–2.72 (m, 2H), 3.24–3.34 (m, 4H), 3.42–3.47 (m, 2H), 3.56–3.61 (m, 2H), 3.63–3.67 (m, 2H), 3.81–3.93 (m, 6H), 4.17–4.29 (m, 2H), 4.31–4.46 (m, 5H), 4.54 (d, *J* = 9.6 Hz, 1H), 4.85–4.94 (m, 1H), 5.01–5.07 (m, 1H), 6.39–6.45 (m, 1H), 6.46–6.54 (m, 2H), 7.02–7.18 (m, 4H), 7.22 (d, *J* = 7.5 Hz, 1H), 7.33 (d, *J* = 9.6 Hz, 1H), 7.35–7.46 (m, 4H), 7.89 (d, *J* = 8.5 Hz, 1H), 8.62 (t, *J* = 6.1 Hz, 1H), 8.75 (d, *J* = 8.1 Hz, 1H), 8.80–8.87 (m, 1H), 9.00 (s, 1H); ^13^C NMR (151 MHz, DMSO-*d*_6_): δ 16.27, 20.36, 25.85, 25.95, 26.01, 26.10, 26.18, 26.62, 28.37, 29.11, 29.21, 29.25, 29.52, 29.67, 30.14, 31.18, 34.95, 36.33, 38.36, 40.51, 42.13, 47.08, 52.58, 55.90, 56.05, 56.30, 57.07, 59.02, 59.23, 67.82, 69.34, 69.87, 70.32, 71.32, 75.39, 103.00, 107.78, 107.98, 126.21, 127.21, 127.94, 128.80, 129.14, 130.04, 130.52, 131.74, 137.48, 137.67, 139.97, 147.96, 152.06, 158.50, 160.40, 168.99, 169.58, 170.18, 170.22, 172.25; LC–MS (ESI) 95% purity, *m*/*z*: [M + H]^+^ calcd for C_69_H_99_N_8_O_11_S, 1247.71; found, 1248.1; HRMS (ESI) *m*/*z*: [M + H]^+^ calcd for C_69_H_99_N_8_O_11_S, 1247.7149; found, 1247.7130.

##### (2*S*,4*S*)-1-((*S*)-2-Cyclohexyl-2-((*S*)-2-(methylamino)propanamido)acetyl)-4-(3-((6-((5-((5-(((*S*)-1-((2*S*,4*R*)-4-hydroxy-2-((4-(4-methylthiazol-5-yl)benzyl)carbamoyl)pyrrolidin-1-yl)-3,3-dimethyl-1-oxobutan-2-yl)amino)-5-oxopentyl)oxy)pentyl)oxy)hexyl)oxy)phenoxy)-*N*-((*R*)-1,2,3,4-tetrahydronaphthalen-1-yl)pyrrolidine-2-carboxamide (PROTAC **7**)

This compound was prepared using general procedure **VII** and PROTAC precursor **113** (80 mg, 58 μmol). After filtration of the solid material, the crude product was purified by column chromatography (CH_2_Cl_2_/MeOH + 7 N NH_3_ 19:1) to give a colorless solid. Yield (38 mg, 52%); *R*_f_ = 0.31 (CH_2_Cl_2_/MeOH + 7 N NH_3_ 19:1); mp 90–92 °C; ^1^H NMR (600 MHz, DMSO-*d*_6_): δ 0.91 (s, 9H), 0.97–1.11 (m, 5H), 1.24–1.83 (m, 22H), 1.85–1.93 (m, 1H), 1.98–2.04 (m, 1H), 2.06–2.13 (m, 2H), 2.15 (s, 3H), 2.17–2.29 (m, 2H), 2.42 (s, 3H), 2.50–2.56 (m, 1H), 2.62–2.74 (m, 2H), 2.94 (q, *J* = 6.8 Hz, 1H), 3.24–3.33 (m, 10H), 3.40–3.51 (m, 4H), 3.59–3.68 (m, 3H), 3.88 (t, *J* = 6.4 Hz, 2H), 4.17–4.31 (m, 2H), 4.31–4.46 (m, 6H), 4.52 (d, *J* = 9.4 Hz, 1H), 4.87–4.93 (m, 1H), 4.98–5.05 (m, 1H), 5.12 (d, *J* = 3.6 Hz, 1H), 6.40 (t, *J* = 2.4 Hz, 1H), 6.45–6.53 (m, 2H), 7.01–7.18 (m, 5H), 7.21–7.25 (m, 1H), 7.34–7.44 (m, 5H), 7.79 (d, *J* = 6.8 Hz, 1H), 7.80 (d, *J* = 7.7 Hz, 1H), 7.91 (d, *J* = 8.6 Hz, 1H), 8.52 (t, *J* = 6.1 Hz, 1H), 8.95 (s, 1H); ^13^C NMR (151 MHz, DMSO-*d*_6_): δ 16.14, 19.28, 20.14, 22.51, 22.74, 25.60, 25.65, 25.71, 25.84, 26.02, 26.60, 28.03, 28.87, 28.97, 29.04, 29.28, 29.42, 29.91, 34.47, 34.63, 34.89, 35.44, 38.14, 40.23, 41.91, 46.89, 52.26, 54.61, 56.57, 58.78, 58.95, 59.33, 67.60, 69.11, 69.88, 70.10, 70.14, 75.20, 102.63, 107.63, 107.82, 125.98, 126.98, 127.67, 128.57, 128.87, 128.91, 129.87, 130.28, 131.42, 137.25, 137.40, 139.71, 147.95, 151.68, 158.29, 160.18, 169.96, 170.14, 170.71, 172.22, 172.34, 174.75; LC–MS (ESI) 97% purity, *m*/*z*: [M + H]^+^ calcd for C_71_H_103_N_8_O_11_S, 1275.75; found, 1276.1; HRMS (ESI) *m*/*z*: [M + H]^+^ calcd for C_71_H_103_N_8_O_11_S, 1275.7462; found, 1275.7422.

##### (2*S*,4*S*)-1-((*S*)-2-Cyclohexyl-2-((*S*)-2-(methylamino)propanamido)acetyl)-4-(3-((6-((6-((6-(((*S*)-1-((2*S*,4*R*)-4-hydroxy-2-((4-(4-methylthiazol-5-yl)benzyl)carbamoyl)pyrrolidin-1-yl)-3,3-dimethyl-1-oxobutan-2-yl)amino)-6-oxohexyl)oxy)hexyl)oxy)hexyl)oxy)phenoxy)-*N*-((*R*)-1,2,3,4-tetrahydronaphthalen-1-yl)pyrrolidine-2-carboxamide (PROTAC **8**)

This compound was prepared using general procedure **VII** and PROTAC precursor **114** (48 mg, 34 μmol). After filtration of the solid material, the crude product was purified by column chromatography (CH_2_Cl_2_/MeOH + 7 N NH_3_ 19:1) to give a colorless solid. Yield (18 mg, 41%); *R*_f_ = 0.20 (CH_2_Cl_2_/MeOH + 7 N NH_3_ 19:1); mp 88–90 °C; ^1^H NMR (600 MHz, DMSO-*d*_6_): δ 0.91 (s, 10H), 0.96–1.14 (m, 7H), 1.14–1.83 (m, 28H), 1.85–1.93 (m, 1H), 1.95–2.05 (m, 1H), 2.05–2.12 (m, 2H), 2.15 (s, 3H), 2.16–2.28 (m, 2H), 2.42 (s, 3H), 2.67 (dd, *J* = 9.1, 15.4 Hz, 2H), 2.95 (p, *J* = 7.5 Hz, 1H), 3.22–3.33 (m, 10H), 3.59–3.68 (m, 3H), 3.87 (t, *J* = 6.4 Hz, 2H), 4.17–4.26 (m, 2H), 4.26–4.46 (m, 6H), 4.52 (d, *J* = 9.4 Hz, 1H), 4.86–4.93 (m, 1H), 5.02 (h, *J* = 4.9 Hz, 1H), 5.12 (d, *J* = 3.5 Hz, 1H), 6.40 (t, *J* = 2.4 Hz, 1H), 6.49 (ddd, *J* = 2.3, 8.2, 20.1 Hz, 2H), 7.01–7.19 (m, 5H), 7.20–7.25 (m, 1H), 7.34–7.46 (m, 5H), 7.75–7.82 (m, 2H), 7.92 (d, *J* = 8.6 Hz, 1H), 8.52 (t, *J* = 6.1 Hz, 1H), 8.95 (s, 1H); ^13^C NMR (151 MHz, DMSO-*d*_6_): δ 16.15, 19.27, 20.15, 25.55, 25.60, 25.64, 25.72, 25.80, 25.82, 25.85, 26.03, 26.62, 28.05, 28.88, 28.98, 29.23, 29.32, 29.42, 29.45, 29.92, 34.46, 34.64, 35.13, 35.44, 38.15, 40.24, 41.92, 46.90, 52.27, 54.63, 56.56, 56.59, 58.80, 58.96, 59.32, 67.61, 69.12, 70.09, 70.14, 75.22, 102.65, 107.65, 107.83, 125.99, 126.99, 127.69, 128.58, 128.89, 128.92, 129.89, 130.30, 131.43, 137.26, 137.40, 139.72, 147.96, 151.70, 158.29, 160.19, 169.98, 170.15, 170.73, 172.24, 172.43, 174.75; LC–MS (ESI) 97% purity, *m*/*z*: [M + H]^+^ calcd for C_73_H_107_N_8_O_11_S, 1303.77; found, 1304.1; HRMS (ESI) *m*/*z*: [M + H]^+^ calcd for C_73_H_107_N_8_O_11_S, 1303.7737; found, 1303.7775.

##### (2*S*,4*S*)-1-((*S*)-2-Cyclohexyl-2-((*S*)-2-(methylamino)propanamido)acetyl)-4-(3-((5-(((*S*)-1-((2*S*,4*R*)-4-hydroxy-2-(((*S*)-1-(4-(4-methylthiazol-5-yl)phenyl)ethyl)carbamoyl)pyrrolidin-1-yl)-3,3-dimethyl-1-oxobutan-2-yl)amino)-5-oxopentyl)oxy)phenoxy)-*N*-((*R*)-1,2,3,4-tetrahydronaphthalen-1-yl)pyrrolidine-2-carboxamide (PROTAC **9**) (**CST626**)

This compound was prepared using general procedure **VII** and PROTAC precursor **115** (100 mg, 83 μmol). The product possessed sufficient purity after filtration. A colorless solid was obtained. Yield (90 mg, 95%); mp 64–66 °C (free base, obtained after column chromatography); ^1^H NMR (600 MHz, DMSO-*d*_6_): δ 0.93 (s, 9H), 0.96–1.20 (m, 6H), 1.30–1.40 (m, 6H), 1.51–1.83 (m, 14H), 1.96–2.10 (m, 2H), 2.14–2.22 (m, 1H), 2.28–2.39 (m, 1H), 2.42–2.47 (m, 6H), 2.51–2.58 (m, 1H), 2.64–2.74 (m, 2H), 3.58–3.68 (m, 4H), 3.81–3.94 (m, 3H), 4.21 (dd, *J* = 5.9, 10.9 Hz, 1H), 4.24–4.30 (m, 1H), 4.37–4.47 (m, 3H), 4.51 (d, *J* = 9.2 Hz, 1H), 4.87–4.95 (m, 2H), 5.04 (h, *J* = 4.7 Hz, 1H), 6.43 (s, 1H), 6.46–6.56 (m, 2H), 7.03–7.19 (m, 4H), 7.23 (d, *J* = 7.5 Hz, 1H), 7.35–7.44 (m, 4H), 7.81 (dd, *J* = 3.7, 9.3 Hz, 1H), 7.88 (d, *J* = 8.5 Hz, 1H), 8.36 (d, *J* = 7.8 Hz, 1H), 8.73 (d, *J* = 8.1 Hz, 1H), 8.80–8.87 (m, 1H), 9.00 (s, 1H), 9.37 (s, 1H); ^13^C NMR (151 MHz, DMSO-*d*_6_): δ 15.95, 16.05, 20.08, 22.26, 22.58, 25.63, 25.80, 25.88, 26.62, 28.07, 28.40, 28.44, 28.91, 28.96, 29.85, 30.90, 34.65, 34.69, 35.37, 37.89, 46.80, 47.88, 52.28, 55.63, 56.02, 56.42, 56.58, 58.73, 67.36, 68.91, 75.09, 102.67, 107.50, 107.79, 125.90, 126.57, 126.91, 128.49, 128.85, 128.99, 129.77, 130.23, 131.40, 137.18, 137.38, 144.88, 147.71, 151.77, 158.24, 160.10, 168.69, 169.76, 169.90, 169.96, 170.80, 172.04; LC–MS (ESI) 99% purity, *m*/*z*: [M + H]^+^ calcd for C_61_H_83_N_8_O_9_S, 1103.60; found, 1103.8; HRMS (ESI) *m*/*z*: [M + H]^+^ calcd for C_61_H_83_N_8_O_9_S, 1103.5998; found, 1103.5980.

##### (2*S*,4*S*)-1-((*S*)-2-Cyclohexyl-2-((*R*)-2-(methylamino)propanamido)acetyl)-4-(3-((5-(((*S*)-1-((2*S*,4*R*)-4-hydroxy-2-(((*S*)-1-(4-(4-methylthiazol-5-yl)phenyl)ethyl)carbamoyl)pyrrolidin-1-yl)-3,3-dimethyl-1-oxobutan-2-yl)amino)-5-oxopentyl)oxy)phenoxy)-*N*-((*R*)-1,2,3,4-tetrahydronaphthalen-1-yl)pyrrolidine-2-carboxamide (PROTAC **10a**)

This compound was prepared using general procedure **VII** and PROTAC precursor **116** (100 mg, 84 μmol). The product possessed sufficient purity after filtration. A colorless solid was obtained. Yield (95 mg, 99%); mp 140–142 °C (dec); ^1^H NMR (600 MHz, DMSO-*d*_6_): δ 0.93 (s, 9H), 1.00–1.20 (m, 4H), 1.39 (d, *J* = 6.9 Hz, 3H), 1.51–1.82 (m, 16H), 1.86–1.93 (m, 1H), 1.99–2.06 (m, 1H), 2.06–2.12 (m, 1H), 2.14–2.22 (m, 1H), 2.29–2.37 (m, 1H), 2.44 (d, *J* = 5.6 Hz, 6H), 2.50–2.58 (m, 1H), 2.64–2.75 (m, 2H), 3.62–3.91 (m, 6H), 4.15–4.31 (m, 2H), 4.31–4.49 (m, 5H), 4.54 (d, *J* = 9.3 Hz, 1H), 4.85–4.94 (m, 1H), 5.01–5.07 (m, 1H), 6.39–6.45 (m, 1H), 6.45–6.55 (m, 2H), 7.03–7.20 (m, 4H), 7.23 (d, *J* = 7.5 Hz, 1H), 7.35–7.43 (m, 4H), 7.84–7.89 (m, 2H), 8.55 (t, *J* = 6.1 Hz, 1H), 8.79–8.90 (m, 2H), 9.00 (s, 1H); ^13^C NMR (151 MHz, DMSO-*d*_6_): δ 16.02, 16.13, 16.79, 20.09, 22.27, 25.59, 25.73, 25.91, 26.56, 28.04, 28.44, 28.90, 28.94, 29.84, 30.85, 34.68, 35.40, 38.12, 40.24, 41.83, 46.82, 52.35, 55.46, 56.03, 56.51, 58.79, 58.87, 67.36, 69.02, 75.16, 102.68, 107.49, 107.77, 125.90, 126.91, 127.61, 128.48, 128.81, 128.84, 129.72, 130.22, 131.45, 137.18, 137.39, 139.74, 147.69, 151.73, 158.23, 160.09, 168.83, 169.86, 169.90, 169.93, 172.09, 172.12; LC–MS (ESI) 99% purity, *m*/*z*: [M + H]^+^ calcd for C_60_H_81_N_8_O_9_S, 1089.58; found, 1089.8; HRMS (ESI) *m*/*z*: [M + H]^+^ calcd for C_60_H_81_N_8_O_9_S, 1089.5842; found, 1089.5823.

##### (2*S*,4*S*)-1-((*S*)-2-Cyclohexyl-2-((*S*)-2-(dimethylamino)propanamido)acetyl)-4-(3-((5-(((*S*)-1-((2*S*,4*R*)-4-hydroxy-2-(((*S*)-1-(4-(4-methylthiazol-5-yl)phenyl)ethyl)carbamoyl)pyrrolidin-1-yl)-3,3-dimethyl-1-oxobutan-2-yl)amino)-5-oxopentyl)oxy)phenoxy)-*N*-((*R*)-1,2,3,4-tetrahydronaphthalen-1-yl)pyrrolidine-2-carboxamide (PROTAC **10b**)

PROTAC **9** (0.11 g, 0.10 mmol) was dissolved in dry DMF (2.5 mL), and 10% Pd/C (11 mg) and formaldehyde 36% in an aqueous solution (25 μL, 0.30 mmol) were added. The mixture was stirred under a hydrogen atmosphere at rt for 16 h. After removal of the catalyst by filtration, it was evaporated, and the crude product was purified by column chromatography (CH_2_Cl_2_/MeOH + 7 N NH_3_ 9:1) to give a colorless solid. Yield (93 mg, 83%); *R*_f_ = 0.54 (CH_2_Cl_2_/MeOH + 7 N NH_3_ 9:1); mp 130–132 °C; ^1^H NMR (600 MHz, DMSO-*d*_6_): δ 0.93 (s, 9H), 1.04 (d, *J* = 6.8 Hz, 3H), 1.36 (d, *J* = 7.0 Hz, 3H), 1.53–1.83 (m, 17H), 1.96–2.03 (m, 1H), 2.05–2.11 (m, 1H), 2.17 (s, 6H), 2.18 (s, 1H), 2.28–2.35 (m, 1H), 2.44 (s, 3H), 2.50–2.58 (m, 1H), 2.63–2.70 (m, 1H), 2.70 (s, 1H), 2.90–2.99 (m, 1H), 3.56–3.64 (m, 3H), 3.85–3.95 (m, 3H), 4.23–4.36 (m, 2H), 4.33–4.39 (m, 1H), 4.39–4.44 (m, 1H), 4.42–4.50 (m, 1H), 4.52 (d, *J* = 9.4 Hz, 1H), 4.86–4.95 (m, 3H), 5.03 (h, *J* = 4.8 Hz, 1H), 5.07 (d, *J* = 3.6 Hz, 1H), 6.44 (t, *J* = 2.4 Hz, 1H), 6.46–6.55 (m, 2H), 7.02–7.19 (m, 5H), 7.20–7.25 (m, 1H), 7.37 (d, *J* = 8.2 Hz, 2H), 7.40–7.44 (m, 2H), 7.74–7.84 (m, 3H), 8.33 (d, *J* = 7.8 Hz, 1H), 8.97 (s, 1H); ^13^C NMR (151 MHz, DMSO-*d*_6_): δ 12.79, 16.12, 20.07, 22.26, 22.56, 25.57, 25.77, 25.95, 26.61, 28.06, 28.42, 28.90, 29.25, 29.84, 34.57, 34.69, 35.35, 37.86, 41.89, 41.93, 46.77, 47.85, 52.16, 56.41, 56.55, 58.67, 58.70, 63.37, 67.32, 68.92, 75.06, 102.51, 107.48, 107.81, 125.89, 126.54, 126.87, 128.48, 128.81, 128.97, 129.85, 130.17, 131.26, 137.16, 137.38, 144.79, 147.91, 151.60, 158.26, 160.09, 169.77, 169.99, 170.52, 170.77, 172.00, 172.55; LC–MS (ESI) 98% purity, *m*/*z*: [M + H]^+^ calcd for C_62_H_85_N_8_O_9_S, 1117.62; found, 1117.9; HRMS (ESI) *m*/*z*: [M + H]^+^ calcd for C_62_H_85_N_8_O_9_S, 1117.6155; found, 1117.6143.

##### (2*S*,4*S*)-1-((*S*)-2-Cyclohexyl-2-((*S*)-2-(*N*-methylacetamido)propanamido)acetyl)-4-(3-((5-(((*S*)-1-((2*S*,4*R*)-4-hydroxy-2-(((*S*)-1-(4-(4-methylthiazol-5-yl)phenyl)ethyl)carbamoyl)pyrrolidin-1-yl)-3,3-dimethyl-1-oxobutan-2-yl)amino)-5-oxopentyl)oxy)phenoxy)-*N*-((*R*)-1,2,3,4-tetrahydronaphthalen-1-yl)pyrrolidine-2-carboxamide (PROTAC **10c**)

PROTAC **9** (0.11 g, 0.10 mmol) was dissolved in dry CH_2_Cl_2_ (2.5 mL) and cooled to 0 °C, and DIPEA (26 μL, 0.15 mmol) and acetic anhydride (15 μL, 0.15 mmol) were added. The mixture was stirred at rt for 16 h. After removal of the volatiles, the crude product was purified by flash chromatography (gradient from 0 to 10% MeOH in CH_2_Cl_2_) to give a colorless solid. Yield (101 mg, 88%); *R*_f_ = 0.44 (CH_2_Cl_2_/MeOH + 7 N NH_3_ 9:1); mp 86–88 °C; ^1^H NMR (600 MHz, DMSO-*d*_6_): δ 0.93 (s, 9H), 1.08 (d, *J* = 25.9 Hz, 2H), 1.13–1.16 (m, 3H), 1.19–1.26 (m, 1H), 1.36 (d, *J* = 7.0 Hz, 3H), 1.53–1.74 (m, 14H), 1.77 (s, 3H), 1.95–2.03 (m, 1H), 2.03–2.12 (m, 2H), 2.14–2.22 (m, 1H), 2.28–2.35 (m, 1H), 2.44 (s, 3H), 2.69 (s, 4H), 2.81 (s, 2H), 3.55–3.66 (m, 3H), 3.89–3.93 (m, 2H), 4.21–4.35 (m, 3H), 4.40–4.46 (m, 2H), 4.52 (d, *J* = 9.4 Hz, 1H), 4.88–4.94 (m, 2H), 4.96–5.05 (m, 2H), 5.08 (d, *J* = 3.6 Hz, 1H), 6.41–6.57 (m, 3H), 7.04–7.19 (m, 5H), 7.24 (d, *J* = 7.5 Hz, 1H), 7.33–7.47 (m, 5H), 7.79–7.86 (m, 3H), 8.33 (d, *J* = 7.8 Hz, 1H), 8.96 (s, 1H); LC–MS (ESI) 99% purity, *m*/*z*: [M + H]^+^ calcd for C_63_H_85_N_8_O_10_S, 1145.61; found, 1146.0; HRMS (ESI) *m*/*z*: [M + H]^+^ calcd for C_63_H_85_N_8_O_10_S, 1145.6104; found, 1145.6100.

##### (2*S*,4*S*)-1-((*S*)-2-Cyclohexyl-2-((*S*)-2-(methylamino)propanamido)acetyl)-4-(3-((5-(((*S*)-1-((2*S*,4*S*)-4-hydroxy-2-((4-(4-methylthiazol-5-yl)benzyl)carbamoyl)pyrrolidin-1-yl)-3,3-dimethyl-1-oxobutan-2-yl)amino)-5-oxopentyl)oxy)phenoxy)-*N*-((*R*)-1,2,3,4-tetrahydronaphthalen-1-yl)pyrrolidine-2-carboxamide (PROTAC **11**)

This compound was prepared using general procedure **VII** and PROTAC precursor **117** (150 mg, 0.126 mmol). The crude product was purified by column chromatography (CH_2_Cl_2_/MeOH/NH_4_OH 9:1:0.1) to give a colorless solid. Yield (64 mg, 47%); *R*_f_ = 0.36 (CH_2_Cl_2_/MeOH/NH_4_OH 9:1:0.1); mp 98–100 °C; ^1^H NMR (400 MHz, CDCl_3_): δ 0.78–0.98 (m, 6H), 1.06 (s, 9H), 1.27 (d, *J* = 6.9 Hz, 3H), 1.36–1.70 (m, 10H), 1.73–1.85 (m, 6H), 1.97–2.07 (m, 2H), 2.10–2.20 (m, 1H), 2.35 (s, 3H), 2.42–2.47 (m, 1H), 2.49 (s, 3H), 2.68–2.77 (m, 2H), 2.85 (d, *J* = 14.0 Hz, 1H), 3.03 (q, *J* = 7.1 Hz, 1H), 3.59–3.66 (m, 1H), 3.73–3.85 (m, 3H), 4.07–4.13 (m, 1H), 4.21–4.36 (m, 3H), 4.40 (d, *J* = 7.4 Hz, 1H), 4.49–4.65 (m, 2H), 4.68–4.78 (m, 2H), 4.94–4.98 (m, 1H), 5.11 (d, *J* = 6.2 Hz, 1H), 6.15 (d, *J* = 7.1 Hz, 1H), 6.34–6.52 (m, 3H), 6.62 (d, *J* = 8.4 Hz, 1H), 7.01–7.06 (m, 2H), 7.08–7.15 (m, 2H), 7.27–7.36 (m, 5H), 7.49 (t, *J* = 6.1 Hz, 1H), 7.64–7.71 (m, 1H), 8.64 (s, 1H); ^13^C NMR (101 MHz, CDCl_3_): δ 16.26, 19.69, 20.19, 22.45, 25.62, 25.75, 25.99, 26.34, 26.82, 28.50, 28.68, 29.41, 29.89, 30.12, 33.55, 34.24, 35.30, 35.68, 37.74, 40.63, 42.90, 47.78, 53.79, 54.93, 55.14, 58.71, 59.53, 60.19, 60.36, 67.72, 69.73, 76.33, 102.91, 107.58, 108.61, 126.34, 127.27, 127.68, 128.77, 129.45, 130.25, 130.67, 131.83, 136.71, 137.57, 138.69, 148.49, 150.35, 158.10, 160.28, 169.67, 171.21, 171.31, 172.69, 174.09, 175.37; **HPLC** (95% H_2_O (with 0.1% TFA) to 95% MeCN in 10 min, then 95% MeCN for 4 min), *t*_R_ = 6.50 min, 97% purity, detection at 254 nm; HRMS (ESI) *m*/*z*: [M + H]^+^ calcd for C_60_H_81_O_9_N_8_S, 1089.5842; found, 1089.5818.

##### *tert*-Butyl ((*S*)-1-(((*S*)-1-Cyclohexyl-2-((2*S*,4*S*)-4-(3-((5-(2-(((2*S*,4*R*)-4-hydroxy-1-((*S*)-3-methyl-2-(1-oxoisoindolin-2-yl)butanoyl)pyrrolidine-2-carboxamido)methyl)-5-(4-methylthiazol-5-yl)phenoxy)pentyl)oxy)phenoxy)-2-(((*R*)-1,2,3,4-tetrahydronaphthalen-1-yl)carbamoyl)pyrrolidin-1-yl)-2-oxoethyl)amino)-1-oxopropan-2-yl)(methyl)carbamate (**118**)

This compound was prepared using general procedure **II**, VHL2 ligand–linker conjugate **83** (196 mg, 0.30 mmol), and IAP ligand **65**. The crude product was purified by flash chromatography (gradient from 0 to 5% MeOH in CH_2_Cl_2_) to give a colorless solid. Yield (70 mg, 18%); *R*_f_ = 0.23 (CH_2_Cl_2_/MeOH 19:1); mp 130–134 °C. Due to the presence of the *N*-Boc protecting group resulting in an additional set of rotamers, NMR data is only provided for the deprotected final PROTAC. LC–MS (ESI) 97% purity, *m*/*z*: [M + H]^+^ calcd for C_72_H_93_N_8_O_12_S, 1293.66; found, 1294.3; HRMS (ESI) *m*/*z*: [M + H]^+^ calcd for C_72_H_93_N_8_O_12_S, 1293.6628; found, 1293.6611.

##### *tert*-Butyl ((*S*)-1-(((*S*)-1-Cyclohexyl-2-((2*S*,4*S*)-4-(3-((8-(2-(((2*S*,4*R*)-4-hydroxy-1-((*S*)-3-methyl-2-(1-oxoisoindolin-2-yl)butanoyl)pyrrolidine-2-carboxamido)methyl)-5-(4-methylthiazol-5-yl)phenoxy)octyl)oxy)phenoxy)-2-(((*R*)-1,2,3,4-tetrahydronaphthalen-1-yl)carbamoyl)pyrrolidin-1-yl)-2-oxoethyl)amino)-1-oxopropan-2-yl)(methyl)carbamate (**119**)

This compound was prepared using general procedure **II**, VHL2 ligand–linker conjugate **84** (209 mg, 0.30 mmol), and IAP ligand **65**. The crude product was purified by column chromatography (CH_2_Cl_2_/MeOH 29:1) to give a colorless solid. Yield (208 mg, 52%); *R*_f_ = 0.24 (CH_2_Cl_2_/MeOH 19:1); mp 128–130 °C. Due to the presence of the *N*-Boc protecting group resulting in an additional set of rotamers, NMR data is only provided for the deprotected final PROTAC. LC–MS (ESI) 98% purity, *m*/*z*: [M + H]^+^ calcd for C_75_H_99_N_8_O_12_S, 1335.71; found, 1336.2; HRMS (ESI) *m*/*z*: [M + H]^+^ calcd for C_75_H_99_N_8_O_12_S, 1335.7098; found, 1335.7072.

##### *tert*-Butyl ((*S*)-1-(((*S*)-1-Cyclohexyl-2-((2*S*,4*S*)-4-(3-(4-(4-(2-(((2*S*,4*R*)-4-hydroxy-1-((*S*)-3-methyl-2-(1-oxoisoindolin-2-yl)butanoyl)pyrrolidine-2-carboxamido)methyl)-5-(4-methylthiazol-5-yl)phenoxy)butoxy)butoxy)phenoxy)-2-(((*R*)-1,2,3,4-tetrahydronaphthalen-1-yl)carbamoyl)pyrrolidin-1-yl)-2-oxoethyl)amino)-1-oxopropan-2-yl)(methyl)carbamate (**120**)

This compound was prepared using general procedure **II**, VHL2 ligand–linker conjugate **85** (213 mg, 0.30 mmol), and IAP ligand **65**. The crude product was purified by column chromatography (CH_2_Cl_2_/MeOH 29:1) to give a colorless solid. Yield (85 mg, 21%); *R*_f_ = 0.19 (CH_2_Cl_2_/MeOH 19:1); mp 114–118 °C. Due to the presence of the *N*-Boc protecting group resulting in an additional set of rotamers, NMR data is only provided for the deprotected final PROTAC. LC–MS (ESI) 96% purity, *m*/*z*: [M + H]^+^ calcd for C_75_H_99_N_8_O_13_S, 1351.71; found, 1352.2; HRMS (ESI) *m*/*z*: [M + H]^+^ calcd for C_75_H_99_N_8_O_13_S, 1351.7047; found, 1351.7051.

##### *tert*-Butyl ((*S*)-1-(((*S*)-1-Cyclohexyl-2-((2*S*,4*S*)-4-(3-(2-(2-(2-(2-(((2*S*,4*R*)-4-hydroxy-1-((*S*)-3-methyl-2-(1-oxoisoindolin-2-yl)butanoyl)pyrrolidine-2-carboxamido)methyl)-5-(4-methylthiazol-5-yl)phenoxy)ethoxy)ethoxy)ethoxy)phenoxy)-2-(((*R*)-1,2,3,4-tetrahydronaphthalen-1-yl)carbamoyl)pyrrolidin-1-yl)-2-oxoethyl)amino)-1-oxopropan-2-yl)(methyl)carbamate (**121**)

This compound was prepared using general procedure **II**, VHL2 ligand–linker conjugate **86** (210 mg, 0.30 mmol), and IAP ligand **65**. The crude product was purified by column chromatography (CH_2_Cl_2_/MeOH 29:1) to give a colorless solid. Yield (104 mg, 26%); *R*_f_ = 0.12 (CH_2_Cl_2_/MeOH 39:1); mp 96–98 °C. Due to the presence of the *N*-Boc protecting group resulting in an additional set of rotamers, NMR data is only provided for the deprotected final PROTAC. LC–MS (ESI) 97% purity, *m*/*z*: [M + H]^+^ calcd for C_73_H_95_N_8_O_14_S, 1339.67; found, 1339.7; HRMS (ESI) *m*/*z*: [M + H]^+^ calcd for C_73_H_95_N_8_O_14_S, 1339.6683; found, 1339.6650.

##### *tert*-Butyl ((*S*)-1-(((*S*)-1-Cyclohexyl-2-((2*S*,4*S*)-4-(3-(2-(2-(2-(2-(2-(((2*S*,4*R*)-4-hydroxy-1-((*S*)-3-methyl-2-(1-oxoisoindolin-2-yl)butanoyl)pyrrolidine-2-carboxamido)methyl)-5-(4-methylthiazol-5-yl)phenoxy)ethoxy)ethoxy)ethoxy)ethoxy)phenoxy)-2-(((*R*)-1,2,3,4-tetrahydronaphthalen-1-yl)carbamoyl)pyrrolidin-1-yl)-2-oxoethyl)amino)-1-oxopropan-2-yl)(methyl)carbamate (**122**)

This compound was prepared using general procedure **II**, VHL2 ligand–linker conjugate **87** (223 mg, 0.30 mmol), and IAP ligand **65**. The crude product was purified by flash chromatography (gradient from 0 to 5% MeOH in CH_2_Cl_2_) to give a colorless solid. Yield (112 mg, 27%); *R*_f_ = 0.15 (CH_2_Cl_2_/MeOH 29:1); mp 96–98 °C. Due to the presence of the *N*-Boc protecting group resulting in an additional set of rotamers, NMR data is only provided for the deprotected final PROTAC. LC–MS (ESI) 96% purity, *m*/*z*: [M + H]^+^ calcd for C_75_H_99_N_8_O_15_S, 1383.69; found, 1384.0; HRMS (ESI) *m*/*z*: [M + H]^+^ calcd for C_75_H_99_N_8_O_15_S, 1383.6945; found, 1383.6920.

##### *tert*-Butyl ((*S*)-1-(((*S*)-1-Cyclohexyl-2-((2*S*,4*S*)-4-(3-((6-((6-(2-(2-(((2*S*,4*R*)-4-hydroxy-1-((*S*)-3-methyl-2-(1-oxoisoindolin-2-yl)butanoyl)pyrrolidine-2-carboxamido)methyl)-5-(4-methylthiazol-5-yl)phenoxy)ethoxy)hexyl)oxy)hexyl)oxy)phenoxy)-2-(((*R*)-1,2,3,4-tetrahydronaphthalen-1-yl)carbamoyl)pyrrolidin-1-yl)-2-oxoethyl)amino)-1-oxopropan-2-yl)(methyl)carbamate (**123**)

This compound was prepared using general procedure **II**, VHL2 ligand–linker conjugate **88** (243 mg, 0.30 mmol), and IAP ligand **65**. The crude product was purified by flash chromatography (gradient from 0 to 6% MeOH in CH_2_Cl_2_) to give a colorless resin. Yield (100 mg, 23%); *R*_f_ = 0.21 (CH_2_Cl_2_/MeOH 19:1). Due to the presence of the *N*-Boc protecting group resulting in an additional set of rotamers, NMR data is only provided for the deprotected final PROTAC. LC–MS (ESI) 98% purity, *m*/*z*: [M + H]^+^ calcd for C_81_H_111_N_8_O_14_S, 1451.79; found, 1452.7; HRMS (ESI) *m*/*z*: [M + H]^+^ calcd for C_81_H_111_N_8_O_14_S, 1451.7935; found, 1451.7932.

##### *tert*-Butyl ((*S*)-1-(((*S*)-1-Cyclohexyl-2-((2*S*,4*S*)-4-(3-((6-((5-((5-(2-(((2*S*,4*R*)-4-hydroxy-1-((*S*)-3-methyl-2-(1-oxoisoindolin-2-yl)butanoyl)pyrrolidine-2-carboxamido)methyl)-5-(4-methylthiazol-5-yl)phenoxy)pentyl)oxy)pentyl)oxy)hexyl)oxy)phenoxy)-2-((®-1,2,3,4-tetrahydronaphthalen-1-yl)carbamoyrrolidinedin-1-yl)-2-oxoethyl)amino)-1-oxopropan-2-yl)(methyl)carbamate (**124**)

This compound was prepared using general procedure **II**, VHL2 ligand–linker conjugate **89** (252 mg, 0.30 mmol), and IAP ligand **65**. The crude product was purified by flash chromatography (gradient from 0 to 5% MeOH in CH_2_Cl_2_) to give a colorless resin. Yield (120 mg, 27%); *R*_f_ = 0.29 (CH_2_Cl_2_/MeOH 19:1). Due to the presence of the *N*-Boc protecting group resulting in an additional set of rotamers, NMR data is only provided for the deprotected final PROTAC. LC–MS (ESI) 93% purity, *m*/*z*: [M + H]^+^ calcd for C_83_H_115_N_8_O_14_S, 1479.82; found, 1479.8; HRMS (ESI) *m*/*z*: [M + H]^+^ calcd for C_83_H_115_N_8_O_14_S, 1479.8248; found, 1479.8238.

##### *tert*-Butyl ((*S*)-1-(((*S*)-1-Cyclohexyl-2-((2*S*,4*S*)-4-(3-((6-((6-((6-(2-(((2*S*,4*R*)-4-hydroxy-1-((*S*)-3-methyl-2-(1-oxoisoindolin-2-yl)butanoyl)pyrrolidine-2-carboxamido)methyl)-5-(4-methylthiazol-5-yl)phenoxy)hexyl)oxy)hexyl)oxy)hexyl)oxy)phenoxy)-2-(((*R*)-1,2,3,4-tetrahydronaphthalen-1-yl)carbamoyl)pyrrolidin-1-yl)-2-oxoethyl)amino)-1-oxopropan-2-yl)(methyl)carbamate (**125**)

This compound was prepared using general procedure **II**, VHL2 ligand–linker conjugate **90** (260 mg, 0.30 mmol), and IAP ligand **65**. The crude product was purified by flash chromatography (gradient from 0 to 5% MeOH in CH_2_Cl_2_) to give a colorless resin. Yield (109 mg, 24%); *R*_f_ = 0.21 (CH_2_Cl_2_/MeOH 19:1). Due to the presence of the *N*-Boc protecting group resulting in an additional set of rotamers, NMR data is only provided for the deprotected final PROTAC. LC–MS (ESI) 84% purity, *m*/*z*: [M + H]^+^ calcd for C_85_H_119_N_8_O_14_S, 1507.86; found, 1507.5; HRMS (ESI) *m*/*z*: [M + H]^+^ calcd for C_85_H_119_N_8_O_14_S, 1507.8561; found, 1507.8562.

##### (2*S*,4*S*)-1-((*S*)-2-Cyclohexyl-2-((*S*)-2-(methylamino)propanamido)acetyl)-4-(3-((5-(2-(((2*S*,4*R*)-4-hydroxy-1-((*S*)-3-methyl-2-(1-oxoisoindolin-2-yl)butanoyl)pyrrolidine-2-carboxamido)methyl)-5-(4-methylthiazol-5-yl)phenoxy)pentyl)oxy)phenoxy)-*N*-((*R*)-1,2,3,4-tetrahydronaphthalen-1-yl)pyrrolidine-2-carboxamide (PROTAC **12**)

This compound was prepared using general procedure **VII** and PROTAC precursor **118** (35 mg, 27 μmol). The product possessed sufficient purity after filtration. A colorless solid was obtained. Yield (30 mg, 90%); mp 164–168 °C; ^1^H NMR (600 MHz, DMSO-*d*_6_): δ 0.72 (d, *J* = 6.7 Hz, 3H), 0.95 (d, *J* = 6.5 Hz, 3H), 0.95–1.24 (m, 6H), 1.33 (d, *J* = 6.9 Hz, 3H), 1.50–1.86 (m, 15H), 1.88–1.95 (m, 1H), 2.00–2.11 (m, 2H), 2.25–2.57 (m, 8H), 2.61–2.74 (m, 2H), 3.66 (dd, *J* = 5.8, 10.5 Hz, 2H), 3.76 (dd, *J* = 4.5, 10.6 Hz, 1H), 3.85 (q, *J* = 6.6 Hz, 1H), 3.94 (t, *J* = 6.4 Hz, 2H), 4.04–4.09 (m, 2H), 4.15–4.62 (m, 9H), 4.70 (d, *J* = 10.8 Hz, 1H), 4.86–4.94 (m, 1H), 5.01–5.07 (m, 1H), 6.33–6.58 (m, 3H), 6.92–7.20 (m, 6H), 7.22 (d, *J* = 7.5 Hz, 1H), 7.33 (d, *J* = 7.7 Hz, 1H), 7.46–7.52 (m, 1H), 7.60 (d, *J* = 4.3 Hz, 2H), 7.70 (d, *J* = 7.6 Hz, 1H), 7.86 (d, *J* = 8.5 Hz, 1H), 8.36 (t, *J* = 5.9 Hz, 1H), 8.73 (d, *J* = 8.1 Hz, 1H), 8.79–8.86 (m, 1H), 8.99 (s, 1H), 9.26–9.33 (m, 1H); ^13^C NMR (151 MHz, DMSO-*d*_6_): δ 15.93, 16.12, 18.76, 19.05, 20.07, 22.40, 25.62, 25.79, 25.87, 28.06, 28.54, 28.56, 28.89, 28.96, 29.84, 30.89, 34.63, 37.21, 38.23, 46.79, 46.97, 52.30, 55.54, 55.61, 56.02, 57.96, 58.75, 58.87, 67.57, 67.84, 68.76, 75.14, 102.74, 107.60, 107.73, 111.91, 120.97, 123.18, 123.77, 125.89, 126.89, 127.21, 127.88, 128.07, 128.47, 128.84, 130.22, 131.10, 131.53, 131.75, 137.18, 137.37, 142.35, 147.95, 151.66, 156.11, 158.22, 160.11, 167.66, 168.28, 168.68, 169.89, 169.94, 171.71; LC–MS (ESI) 98% purity, *m*/*z*: [M + H]^+^ calcd for C_67_H_85_N_8_O_10_S, 1193.61; found, 1191.9; HRMS (ESI) *m*/*z*: [M + H]^+^ calcd for C_67_H_85_N_8_O_10_S, 1193.6104; found, 1193.6079.

##### (2*S*,4*S*)-1-((*S*)-2-Cyclohexyl-2-((*S*)-2-(methylamino)propanamido)acetyl)-4-(3-((8-(2-(((2*S*,4*R*)-4-hydroxy-1-((*S*)-3-methyl-2-(1-oxoisoindolin-2-yl)butanoyl)pyrrolidine-2-carboxamido)methyl)-5-(4-methylthiazol-5-yl)phenoxy)octyl)oxy)phenoxy)-*N*-((*R*)-1,2,3,4-tetrahydronaphthalen-1-yl)pyrrolidine-2-carboxamide (PROTAC **13**)

This compound was prepared using general procedure **VII** and PROTAC precursor **119** (100 mg, 75 μmol). The product possessed sufficient purity after filtration. A colorless solid was obtained. Yield (94 mg, 99%); mp 212–214 °C; ^1^H NMR (600 MHz, DMSO-*d*_6_): δ 0.72 (d, *J* = 6.7 Hz, 3H), 0.95 (d, *J* = 6.6 Hz, 3H), 0.97–1.18 (m, 6H), 1.31–1.48 (m, 11H), 1.53–1.82 (m, 8H), 1.88–1.95 (m, 1H), 2.00–2.11 (m, 2H), 2.27–2.40 (m, 1H), 2.44–2.47 (m, 6H), 2.63–2.74 (m, 2H), 3.63–3.73 (m, 6H), 3.76 (dd, *J* = 4.5, 10.6 Hz, 2H), 3.82–3.93 (m, 3H), 4.03 (t, *J* = 6.4 Hz, 2H), 4.16–4.25 (m, 2H), 4.26–4.34 (m, 2H), 4.32–4.47 (m, 4H), 4.53 (d, *J* = 18.1 Hz, 1H), 4.70 (d, *J* = 10.8 Hz, 1H), 4.91 (q, *J* = 7.1 Hz, 1H), 5.04 (p, *J* = 5.1 Hz, 1H), 6.40–6.45 (m, 1H), 6.46–6.55 (m, 2H), 6.96–7.01 (m, 2H), 7.01–7.19 (m, 5H), 7.22 (d, *J* = 7.5 Hz, 1H), 7.32 (d, *J* = 7.7 Hz, 1H), 7.45–7.52 (m, 1H), 7.55–7.63 (m, 2H), 7.70 (d, *J* = 7.5 Hz, 1H), 7.85 (d, *J* = 8.6 Hz, 1H), 8.36 (t, *J* = 5.9 Hz, 1H), 8.73 (d, *J* = 8.1 Hz, 1H), 8.80–8.85 (m, 1H), 9.00 (s, 1H), 9.30–9.35 (m, 1H); ^13^C NMR (151 MHz, DMSO-*d*_6_): δ 15.92, 16.08, 18.76, 19.03, 20.05, 25.61, 25.67, 25.71, 25.77, 25.86, 28.05, 28.52, 28.81, 28.88, 28.92, 28.95, 29.83, 30.87, 34.61, 37.16, 38.23, 46.77, 46.96, 52.29, 55.54, 55.59, 56.00, 57.94, 58.74, 58.85, 67.59, 67.90, 68.75, 75.13, 102.74, 107.52, 107.69, 111.87, 120.91, 123.16, 123.75, 125.88, 126.88, 127.20, 127.83, 128.05, 128.47, 128.82, 130.20, 131.03, 131.53, 131.56, 131.72, 137.16, 137.35, 142.33, 147.85, 151.69, 156.11, 158.19, 160.10, 167.63, 168.26, 168.66, 169.90, 171.68; LC–MS (ESI) 98% purity, *m*/*z*: [M + H]^+^ calcd for C_70_H_91_N_8_O_10_S, 1235.66; found, 1235.7; HRMS (ESI) *m*/*z*: [M + H]^+^ calcd for C_70_H_91_N_8_O_10_S, 1235.6573; found, 1235.6530.

##### (2*S*,4*S*)-1-((*S*)-2-Cyclohexyl-2-((*S*)-2-(methylamino)propanamido)acetyl)-4-(3-(4-(4-(2-(((2*S*,4*R*)-4-hydroxy-1-((*S*)-3-methyl-2-(1-oxoisoindolin-2-yl)butanoyl)pyrrolidine-2-carboxamido)methyl)-5-(4-methylthiazol-5-yl)phenoxy)butoxy)butoxy)phenoxy)-*N*-((*R*)-1,2,3,4-tetrahydronaphthalen-1-yl)pyrrolidine-2-carboxamide (PROTAC **14**)

This compound was prepared using general procedure **VII** and PROTAC precursor **120** (50 mg, 37 μmol). The product possessed sufficient purity after filtration. A colorless solid was obtained. Yield (42 mg, 88%); mp 170–174 °C; ^1^H NMR (600 MHz, DMSO-*d*_6_): δ 0.72 (d, *J* = 6.7 Hz, 3H), 0.95 (d, *J* = 6.6 Hz, 3H), 0.96–1.15 (m, 6H), 1.33 (d, *J* = 6.9 Hz, 3H), 1.50–1.83 (m, 12H), 1.88–1.96 (m, 1H), 2.00–2.11 (m, 2H), 2.27–2.34 (m, 1H), 2.44–2.47 (m, 6H), 2.50–2.59 (m, 1H), 2.61–2.74 (m, 2H), 3.48–3.58 (m, 10H), 3.63–3.69 (m, 2H), 3.73–3.93 (m, 4H), 4.06 (t, *J* = 6.3 Hz, 2H), 4.17–4.53 (m, 10H), 4.70 (d, *J* = 10.7 Hz, 1H), 4.87–4.94 (m, 1H), 5.04 (p, *J* = 5.2 Hz, 1H), 6.42 (q, *J* = 3.5 Hz, 1H), 6.46–6.54 (m, 2H), 6.99 (s, 1H), 7.04–7.18 (m, 4H), 7.22 (d, *J* = 7.5 Hz, 1H), 7.33 (d, *J* = 7.7 Hz, 1H), 7.45–7.53 (m, 1H), 7.60 (d, *J* = 5.4 Hz, 2H), 7.70 (d, *J* = 7.5 Hz, 1H), 7.86 (d, *J* = 8.5 Hz, 1H), 8.36 (t, *J* = 5.9 Hz, 1H), 8.73 (d, *J* = 8.1 Hz, 1H), 8.79–8.86 (m, 1H), 8.98 (s, 1H); ^13^C NMR (151 MHz, DMSO-*d*_6_): δ 15.93, 16.11, 18.77, 19.04, 20.06, 25.62, 25.79, 25.86, 26.07, 28.06, 28.53, 28.89, 28.96, 29.84, 30.89, 34.63, 37.20, 38.23, 40.23, 46.79, 46.97, 52.30, 55.55, 55.61, 56.02, 57.96, 58.74, 58.87, 67.45, 67.76, 68.77, 69.76, 69.85, 75.13, 102.77, 107.49, 107.74, 111.87, 120.95, 123.18, 123.76, 125.89, 126.89, 127.19, 127.89, 128.07, 128.48, 128.84, 130.21, 131.08, 131.53, 131.74, 137.18, 137.36, 142.35, 147.93, 151.66, 156.08, 158.21, 160.07, 167.65, 168.27, 168.68, 169.90, 169.92, 171.70; LC–MS (ESI) 99% purity, *m*/*z*: [M + H]^+^ calcd for C_70_H_91_N_8_O_11_S, 1251.65; found, 1252.0; HRMS (ESI) *m*/*z*: [M + H]^+^ calcd for C_70_H_91_N_8_O_11_S, 1251.6523; found, 1251.6488.

##### (2*S*,4*S*)-1-((*S*)-2-Cyclohexyl-2-((*S*)-2-(methylamino)propanamido)acetyl)-4-(3-(2-(2-(2-(2-(((2*S*,4*R*)-4-hydroxy-1-((*S*)-3-methyl-2-(1-oxoisoindolin-2-yl)butanoyl)pyrrolidine-2-carboxamido)methyl)-5-(4-methylthiazol-5-yl)phenoxy)ethoxy)ethoxy)ethoxy)phenoxy)-*N*-((*R*)-1,2,3,4-tetrahydronaphthalen-1-yl)pyrrolidine-2-carboxamide (PROTAC **15**)

This compound was prepared using general procedure **VII** and PROTAC precursor **121** (58 mg, 43 μmol). The product possessed sufficient purity after filtration. A colorless solid was obtained. Yield (54 mg, 98%); mp 206–210 °C; ^1^H NMR (600 MHz, DMSO-*d*_6_): δ 0.72 (d, *J* = 6.5 Hz, 3H), 0.91–1.13 (m, 7H), 1.31–1.37 (m, 3H), 1.53–1.83 (m, 12H), 1.87–1.94 (m, 1H), 2.00–2.10 (m, 2H), 2.27–2.39 (m, 1H), 2.41–2.47 (m, 6H), 2.51–2.71 (m, 5H), 3.67–3.81 (m, 8H), 3.81–3.94 (m, 2H), 4.02 (t, *J* = 4.6 Hz, 3H), 4.15–4.27 (m, 5H), 4.31 (dd, *J* = 5.2, 16.4 Hz, 3H), 4.37–4.46 (m, 4H), 4.70 (d, *J* = 10.7 Hz, 1H), 4.90 (q, *J* = 7.4 Hz, 1H), 6.46 (s, 1H), 6.48 (d, *J* = 8.2 Hz, 1H), 6.53 (d, *J* = 8.8 Hz, 1H), 6.98–7.18 (m, 7H), 7.22 (d, *J* = 7.6 Hz, 1H), 7.33 (d, *J* = 7.9 Hz, 1H), 7.46–7.52 (m, 1H), 7.60 (d, *J* = 4.4 Hz, 2H), 7.70 (d, *J* = 7.7 Hz, 1H), 7.89 (d, *J* = 8.6 Hz, 1H), 8.36 (d, *J* = 6.5 Hz, 1H), 8.73 (d, *J* = 8.2 Hz, 1H), 8.99 (d, *J* = 2.7 Hz, 1H); ^13^C NMR (151 MHz, DMSO-*d*_6_): δ 15.93, 16.10, 18.76, 19.03, 20.05, 25.61, 25.78, 25.86, 28.06, 28.53, 28.88, 28.92, 29.83, 30.87, 34.61, 37.23, 38.23, 40.23, 46.77, 46.96, 52.27, 55.55, 55.61, 56.00, 57.94, 58.70, 58.87, 67.29, 68.09, 68.76, 69.10, 69.19, 70.11, 70.26, 75.06, 102.61, 107.44, 108.01, 112.35, 121.25, 123.16, 123.75, 125.88, 126.88, 127.42, 127.89, 128.05, 128.47, 128.82, 130.23, 131.06, 131.49, 131.53, 131.73, 137.16, 137.37, 142.34, 147.93, 151.70, 156.04, 158.22, 159.88, 167.63, 168.26, 168.66, 169.87, 169.93, 171.71; LC–MS (ESI) 98% purity, *m*/*z*: [M + H]^+^ calcd for C_68_H_87_N_8_O_12_S, 1239.62; found, 1240.0; HRMS (ESI) *m*/*z*: [M + H]^+^ calcd for C_68_H_87_N_8_O_12_S, 1239.6159; found, 1239.6109.

##### (2*S*,4*S*)-1-((*S*)-2-Cyclohexyl-2-((*S*)-2-(methylamino)propanamido)acetyl)-4-(3-(2-(2-(2-(2-(2-(((2*S*,4*R*)-4-hydroxy-1-((*S*)-3-methyl-2-(1-oxoisoindolin-2-yl)butanoyl)pyrrolidine-2-carboxamido)methyl)-5-(4-methylthiazol-5-yl)phenoxy)ethoxy)ethoxy)ethoxy)ethoxy)phenoxy)-*N*-((*R*)-1,2,3,4-tetrahydronaphthalen-1-yl)pyrrolidine-2-carboxamide (PROTAC **16**)

This compound was prepared using general procedure **VII** and PROTAC precursor **122** (55 mg, 40 μmol). The product possessed sufficient purity after filtration. A colorless solid was obtained. Yield (51 mg, 99%); mp 144–148 °C; ^1^H NMR (600 MHz, DMSO-*d*_6_): δ 0.72 (d, *J* = 6.6 Hz, 3H), 0.93–1.17 (m, 8H), 1.33 (d, *J* = 6.9 Hz, 3H), 1.49–1.85 (m, 10H), 1.87–1.94 (m, 1H), 2.00–2.09 (m, 2H), 2.26–2.38 (m, 1H), 2.40–2.48 (m, 6H), 2.50–2.58 (m, 1H), 2.63–2.74 (m, 2H), 3.51–3.72 (m, 13H), 3.73–3.80 (m, 3H), 3.80–3.93 (m, 1H), 3.96–4.05 (m, 2H), 4.14–4.56 (m, 11H), 4.70 (d, *J* = 10.7 Hz, 1H), 4.85–4.93 (m, 1H), 5.03 (p, *J* = 5.3 Hz, 1H), 6.36–6.55 (m, 3H), 6.95–7.02 (m, 1H), 7.02–7.24 (m, 6H), 7.33 (d, *J* = 7.8 Hz, 1H), 7.43–7.52 (m, 1H), 7.54–7.63 (m, 2H), 7.70 (d, *J* = 7.5 Hz, 1H), 7.88 (d, *J* = 8.5 Hz, 1H), 8.35 (t, *J* = 6.0 Hz, 1H), 8.73 (d, *J* = 8.1 Hz, 1H), 8.79–8.86 (m, 1H), 8.99 (s, 1H); ^13^C NMR (151 MHz, DMSO-*d*_6_): δ 15.93, 16.10, 18.77, 19.04, 20.05, 25.62, 25.79, 25.87, 28.06, 28.54, 28.89, 28.93, 29.83, 30.89, 34.62, 37.22, 38.25, 40.24, 46.78, 46.97, 52.27, 55.56, 55.61, 56.01, 57.95, 58.70, 58.87, 67.31, 68.10, 68.77, 69.06, 69.15, 70.01, 70.07, 70.24, 75.06, 102.60, 107.45, 108.02, 112.35, 121.25, 123.17, 123.76, 125.89, 126.90, 127.42, 127.88, 128.06, 128.49, 128.83, 130.24, 131.07, 131.50, 131.53, 131.74, 137.18, 137.37, 142.35, 147.95, 151.70, 156.05, 158.23, 159.88, 167.65, 168.27, 168.68, 169.88, 169.93, 171.72; LC–MS (ESI) 99% purity, *m*/*z*: [M + H]^+^ calcd for C_70_H_91_N_8_O_13_S, 1283.64; found, 1283.8; HRMS (ESI) *m*/*z*: [M + H]^+^ calcd for C_70_H_91_N_8_O_13_S, 1283.6421; found, 1283.6380.

##### (2*S*,4*S*)-1-((*S*)-2-Cyclohexyl-2-((*S*)-2-(methylamino)propanamido)acetyl)-4-(3-((6-((6-(2-(2-(((2*S*,4*R*)-4-hydroxy-1-((*S*)-3-methyl-2-(1-oxoisoindolin-2-yl)butanoyl)pyrrolidine-2-carboxamido)methyl)-5-(4-methylthiazol-5-yl)phenoxy)ethoxy)hexyl)oxy)hexyl)oxy)phenoxy)-*N*-((*R*)-1,2,3,4-tetrahydronaphthalen-1-yl)pyrrolidine-2-carboxamide (PROTAC **17**)

This compound was prepared using general procedure **VII** and PROTAC precursor **123** (35 mg, 24 μmol). The product possessed sufficient purity after filtration. A colorless solid was obtained. Yield (29 mg, 86%); mp 118–122 °C; ^1^H NMR (600 MHz, DMSO-*d*_6_): δ 0.72 (d, *J* = 6.7 Hz, 3H), 0.95 (d, *J* = 6.5 Hz, 3H), 0.96–1.15 (m, 5H), 1.19–1.85 (m, 30H), 1.87–1.95 (m, 1H), 2.00–2.11 (m, 2H), 2.27–2.40 (m, 1H), 2.40–2.48 (m, 6H), 2.50–2.59 (m, 1H), 2.64–2.75 (m, 2H), 3.25–3.33 (m, 4H), 3.46 (t, *J* = 6.5 Hz, 2H), 3.63–3.93 (m, 8H), 4.14–4.56 (m, 11H), 4.70 (d, *J* = 10.8 Hz, 1H), 4.87–4.94 (m, 1H), 5.04 (p, *J* = 5.1 Hz, 1H), 6.39–6.44 (m, 1H), 6.46–6.54 (m, 2H), 7.00 (dd, *J* = 1.6, 7.8 Hz, 1H), 7.03–7.19 (m, 5H), 7.22 (d, *J* = 7.4 Hz, 1H), 7.33 (d, *J* = 7.8 Hz, 1H), 7.43–7.52 (m, 1H), 7.61 (q, *J* = 4.1 Hz, 2H), 7.70 (d, *J* = 7.5 Hz, 1H), 7.86 (d, *J* = 8.6 Hz, 1H), 8.34 (t, *J* = 6.0 Hz, 1H), 8.73 (d, *J* = 8.1 Hz, 1H), 8.78–8.86 (m, 1H), 8.99 (s, 1H); ^13^C NMR (151 MHz, DMSO-*d*_6_): δ 15.91, 16.12, 18.76, 19.03, 20.04, 25.53, 25.61, 25.64, 25.72, 25.77, 25.86, 28.05, 28.53, 28.80, 28.89, 28.94, 29.36, 29.83, 30.88, 34.62, 37.20, 38.22, 40.24, 46.77, 46.96, 52.28, 55.53, 55.59, 56.00, 57.94, 58.73, 58.86, 67.53, 68.12, 68.75, 68.81, 70.00, 70.07, 70.61, 75.12, 102.73, 107.50, 107.68, 112.40, 121.22, 123.16, 123.74, 125.87, 126.87, 127.40, 127.85, 128.05, 128.47, 128.82, 130.19, 131.07, 131.47, 131.53, 131.72, 137.16, 137.35, 142.33, 147.95, 151.64, 156.08, 158.19, 160.09, 167.62, 168.24, 168.66, 169.89, 171.69; LC–MS (ESI) 98% purity, *m*/*z*: [M + H]^+^ calcd for C_76_H_103_N_8_O_12_S, 1351.74; found, 1352.1; HRMS (ESI) *m*/*z*: [M + H]^+^ calcd for C_76_H_103_N_8_O_12_S, 1351.7411; found, 1351.7349.

##### (2*S*,4*S*)-1-((*S*)-2-Cyclohexyl-2-((*S*)-2-(methylamino)propanamido)acetyl)-4-(3-((6-((5-((5-(2-(((2*S*,4*R*)-4-hydroxy-1-((*S*)-3-methyl-2-(1-oxoisoindolin-2-yl)butanoyl)pyrrolidine-2-carboxamido)methyl)-5-(4-methylthiazol-5-yl)phenoxy)pentyl)oxy)pentyl)oxy)hexyl)oxy)phenoxy)-*N*-((*R*)-1,2,3,4-tetrahydronaphthalen-1-yl)pyrrolidine-2-carboxamide (PROTAC **18**)

This compound was prepared using general procedure **VII** and PROTAC precursor **124** (60 mg, 40 μmol). After filtration of the solid material, the crude product was purified by column chromatography (CH_2_Cl_2_/MeOH + 7 N NH_3_ 9:1) followed by preparative HPLC (100% MeOH) to give a colorless solid. Yield (48 mg, 87%); mp 98–102 °C; ^1^H NMR (600 MHz, DMSO-*d*_6_): δ 0.73 (d, *J* = 6.7 Hz, 3H), 0.85 (t, *J* = 6.9 Hz, 2H), 0.95 (d, *J* = 6.5 Hz, 3H), 1.07 (d, *J* = 6.9 Hz, 3H), 1.19–1.82 (m, 30H), 1.88–1.95 (m, 1H), 1.99–2.11 (m, 2H), 2.16 (s, 3H), 2.17–2.24 (m, 1H), 2.31 (s, 1H), 2.46 (s, 4H), 2.50–2.56 (m, 1H), 2.64–2.75 (m, 2H), 2.87–2.97 (m, 1H), 3.30–3.39 (m, 8H), 3.61 (dd, *J* = 4.5, 10.7 Hz, 1H), 3.65–3.70 (m, 1H), 3.74–3.79 (m, 1H), 3.82–3.92 (m, 2H), 4.03 (t, *J* = 6.3 Hz, 2H), 4.19–4.57 (m, 10H), 4.70 (d, *J* = 10.8 Hz, 1H), 4.88–4.94 (m, 1H), 4.99–5.08 (m, 2H), 6.42 (t, *J* = 2.4 Hz, 1H), 6.45–6.53 (m, 2H), 6.95–7.01 (m, 2H), 7.01–7.17 (m, 5H), 7.24 (d, *J* = 7.5 Hz, 1H), 7.32 (d, *J* = 7.7 Hz, 1H), 7.46–7.52 (m, 1H), 7.55–7.63 (m, 2H), 7.70 (d, *J* = 7.6 Hz, 1H), 7.82 (d, *J* = 8.6 Hz, 1H), 7.90 (d, *J* = 8.7 Hz, 1H), 8.33 (t, *J* = 6.0 Hz, 1H), 8.97 (s, 1H); ^13^C NMR (151 MHz, DMSO-*d*_6_): δ 14.10, 16.15, 18.76, 19.03, 19.22, 20.06, 22.21, 22.56, 22.67, 25.52, 25.58, 25.64, 25.76, 25.95, 27.95, 28.54, 28.65, 28.81, 28.90, 29.11, 29.20, 29.35, 29.83, 31.10, 34.41, 34.57, 37.17, 38.21, 40.24, 46.77, 46.96, 52.15, 54.47, 55.54, 57.94, 58.65, 58.85, 59.28, 67.51, 67.85, 68.77, 70.00, 70.05, 70.08, 75.05, 102.51, 107.50, 107.73, 111.86, 120.90, 123.16, 123.74, 125.87, 126.85, 127.15, 127.83, 128.04, 128.51, 128.79, 130.15, 131.12, 131.46, 131.54, 131.71, 137.14, 137.38, 142.34, 148.03, 151.54, 156.10, 158.24, 160.10, 167.62, 168.26, 170.01, 170.60, 171.66, 174.58; LC–MS (ESI) 98% purity, *m*/*z*: [M + H]^+^ calcd for C_78_H_107_N_8_O_12_S, 1379.77; found, 1379.6; HRMS (ESI) *m*/*z*: [M + H]^+^ calcd for C_78_H_107_N_8_O_12_S, 1379.7724; found, 1379.7662.

##### (2*S*,4*S*)-1-((*S*)-2-Cyclohexyl-2-((*S*)-2-(methylamino)propanamido)acetyl)-4-(3-((6-((6-((6-(2-(((2*S*,4*R*)-4-hydroxy-1-((*S*)-3-methyl-2-(1-oxoisoindolin-2-yl)butanoyl)pyrrolidine-2-carboxamido)methyl)-5-(4-methylthiazol-5-yl)phenoxy)hexyl)oxy)hexyl)oxy)hexyl)oxy)phenoxy)-*N*-((*R*)-1,2,3,4-tetrahydronaphthalen-1-yl)pyrrolidine-2-carboxamide (PROTAC **19**)

This compound was prepared using general procedure **VII** and PROTAC precursor **125** (32 mg, 21 μmol). After filtration of the solid material, the crude product was purified by preparative HPLC (gradient from 60 to 100% ACN +0.05% TFA) to give a colorless solid. Yield (23 mg, 75%); mp 102–104 °C; ^1^H NMR (600 MHz, DMSO-*d*_6_): δ 0.73 (d, *J* = 6.7 Hz, 3H), 0.95 (d, *J* = 6.5 Hz, 3H), 0.97–1.13 (m, 5H), 1.25–1.29 (m, 5H), 1.31 (d, *J* = 6.8 Hz, 3H), 1.33–1.82 (m, 25H), 1.87–1.96 (m, 1H), 1.98–2.12 (m, 2H), 2.27–2.43 (m, 2H), 2.45 (s, 3H), 2.50–2.74 (m, 3H), 3.26–3.36 (m, 6H), 3.69 (s, 8H), 3.76 (dd, *J* = 4.5, 10.6 Hz, 1H), 3.82–3.93 (m, 3H), 4.03 (t, *J* = 6.3 Hz, 2H), 4.15–4.35 (m, 5H), 4.38–4.57 (m, 5H), 4.70 (d, *J* = 10.8 Hz, 1H), 4.85–4.94 (m, 1H), 5.00–5.08 (m, 1H), 6.41 (t, *J* = 2.4 Hz, 1H), 6.45–6.56 (m, 2H), 6.96–7.00 (m, 2H), 7.03–7.20 (m, 6H), 7.22 (d, *J* = 7.5 Hz, 1H), 7.32 (d, *J* = 7.6 Hz, 1H), 7.47–7.52 (m, 1H), 7.57–7.63 (m, 2H), 7.70 (d, *J* = 7.5 Hz, 1H), 7.85 (d, *J* = 8.5 Hz, 1H), 8.33 (t, *J* = 6.0 Hz, 1H), 8.71 (d, *J* = 8.1 Hz, 1H), 8.74–8.81 (m, 1H), 8.97 (s, 1H).; ^13^C NMR (151 MHz, DMSO-*d*_6_): δ 15.86, 16.14, 18.77, 19.04, 20.04, 25.53, 25.56, 25.61, 25.65, 25.72, 25.78, 25.87, 28.04, 28.54, 28.81, 28.90, 28.97, 29.35, 29.38, 29.84, 30.93, 34.64, 37.18, 38.22, 40.23, 46.79, 46.97, 52.29, 55.54, 55.59, 56.02, 57.96, 58.74, 58.86, 67.54, 67.85, 68.77, 70.01, 70.06, 75.13, 102.78, 107.51, 107.68, 111.86, 120.92, 123.18, 123.76, 125.89, 126.90, 127.17, 127.87, 128.07, 128.48, 128.85, 130.20, 131.13, 131.49, 131.54, 131.74, 137.19, 137.35, 142.35, 148.01, 151.59, 156.12, 158.17, 158.20, 158.41, 160.10, 167.65, 168.28, 168.72, 169.88, 171.68; LC–MS (ESI) 99% purity, *m*/*z*: [M + H]^+^ calcd for C_80_H_111_N_8_O_12_S, 1407.80; found, 1408.8; HRMS (ESI) *m*/*z*: [M + H]^+^ calcd for C_80_H_111_N_8_O_12_S, 1407.8037; found, 1407.8026.

##### *tert*-Butyl ((2*S*)-1-(((1*S*)-1-Cyclohexyl-2-((2*S*,4*S*)-4-(3-((5-((2-(2,6-dioxopiperidin-3-yl)-1,3-dioxoisoindolin-4-yl)amino)pentyl)oxy)phenoxy)-2-(((*R*)-1,2,3,4-tetrahydronaphthalen-1-yl)carbamoyl)pyrrolidin-1-yl)-2-oxoethyl)amino)-1-oxopropan-2-yl)(methyl)carbamate (**126**)

This compound was prepared using general procedure **VIII**, **99** (75 mg, 98 μmol), and **72** (27 mg, 98 μmol). The crude product was purified by column chromatography (EtOAc/n-hexanes 4:1) to give a yellow resin. Yield (51 mg, 51%); *R*_f_ = 0.30 (EtOAc/n-hexanes 4:1). Due to the presence of the *N*-Boc protecting group resulting in an additional set of rotamers, NMR data is only provided for the deprotected final PROTAC. HRMS (ESI) *m*/*z*: [M + H]^+^ calcd for C_56_H_72_O_11_N_7_, 1018.5284; found, 1018.5250.

##### *tert*-Butyl ((2*S*)-1-(((1*S*)-1-Cyclohexyl-2-((2*S*,4*S*)-4-(3-((8-((2-(2,6-dioxopiperidin-3-yl)-1,3-dioxoisoindolin-4-yl)amino)octyl)oxy)phenoxy)-2-(((*R*)-1,2,3,4-tetrahydronaphthalen-1-yl)carbamoyl)pyrrolidin-1-yl)-2-oxoethyl)amino)-1-oxopropan-2-yl)(methyl)carbamate (**127**)

This compound was prepared using general procedure **VIII**, **100** (70 mg, 87 μmol) and **72** (24 mg, 87 μmol). The crude product was purified by column chromatography (EtOAc/*n*-hexanes 4:1) to give a yellow resin. Yield (55 mg, 59%); *R*_f_ = 0.32 (EtOAc/*n*-hexanes 4:1). Due to the presence of the *N*-Boc protecting group resulting in an additional set of rotamers, NMR data is only provided for the deprotected final PROTAC. HRMS (ESI) *m*/*z*: [M + H]^+^ calcd for C_59_H_77_O_11_N_7_, 1060.5754; found, 1060.5736.

##### *tert*-Butyl ((2*S*)-1-(((1*S*)-1-Cyclohexyl-2-((2*S*,4*S*)-4-(3-(4-(4-((2-(2,6-dioxopiperidin-3-yl)-1,3-dioxoisoindolin-4-yl)amino)butoxy)butoxy)phenoxy)-2-((*R*-1,2,3,4-tetrahydronaphthalen-1-yl)carbamoylpyrrolidin-1-yl)-2-oxoethyl)amino-1-oxopropan-2-yl)-(methyl)carbamate (**128**)

This compound was prepared using general procedure **VIII**, **101** (94 mg, 115 μmol) and **72** (32 mg, 115 μmol). The crude product was purified by column chromatography (CH_2_Cl_2_/MeOH 20:1) to give a yellow resin. Yield (72 mg, 58%); *R*_f_ = 0.35 (CH_2_Cl_2_/MeOH 9:1). Due to the presence of the *N*-Boc protecting group resulting in an additional set of rotamers, NMR data is only provided for the deprotected final PROTAC. HRMS (ESI) *m*/*z*: [M + H]^+^ calcd for C_59_H_78_O_12_N_7_, 1076.5703; found, 1076.5691.

##### *tert*-Butyl ((2*S*)-1-(((1*S*)-1-Cyclohexyl-2-((2*S*,4*S*)-4-(3-(2-(2-(2-((2-(2,6-dioxopiperidin-3-yl)-1,3-dioxoisoindolin-4-yl)amino)ethoxy)ethoxy)ethoxy)phenoxy)-2-(((*R*)-1,2,3,4-tetrahydronaphthalen-1-yl)carbamoyl)pyrrolidin-1-yl)-2-oxoethyl)amino)-1-oxopropan-2-yl)(methyl)carbamate (**129**)

This compound was prepared using general procedure **VIII**, **102** (71 mg, 88 μmol), and **72** (24 mg, 88 μmol). The crude product was purified by column chromatography (CH_2_Cl_2_/MeOH 20:1) to give a yellow resin. Yield (72 mg, 58%); *R*_f_ = 0.35 (CH_2_Cl_2_/MeOH 9:1). Due to the presence of the *N*-Boc protecting group resulting in an additional set of rotamers, NMR data is only provided for the deprotected final PROTAC. HRMS (ESI) *m*/*z*: [M + H]^+^ calcd for C_57_H_74_O_13_N_7_, 1064.5339; found, 1064.5325.

##### *tert*-Butyl ((2*S*)-1-(((1*S*)-1-Cyclohexyl-2-((2*S*,4*S*)-4-(3-(2-(2-(2-(2-((2-(2,6-dioxopiperidin-3-yl)-1,3-dioxoisoindolin-4-yl)amino)ethoxy)ethoxy)ethoxy)ethoxy)phenoxy)-2-(((*R*)-1,2,3,4-tetrahydronaphthalen-1-yl)carbamoyl)pyrrolidin-1-yl)-2-oxoethyl)amino)-1-oxopropan-2-yl)(methyl)carbamate (**130**)

This compound was prepared using general procedure **VIII**, **103** (100 mg, 117 μmol), and **72** (32 mg, 117 μmol). The crude product was purified by column chromatography (CH_2_Cl_2_/MeOH 20:1) to give a yellow resin. Yield (30 mg, 23%); *R*_f_ = 0.35 (CH_2_Cl_2_/MeOH 9:1). Due to the presence of the *N*-Boc protecting group resulting in an additional set of rotamers, NMR data is only provided for the deprotected final PROTAC. HRMS (ESI) *m*/*z*: [M + H]^+^ calcd for C_59_H_78_O_14_N_7_, 1108.5601; found, 1108.5585.

##### *tert*-Butyl ((2*S*)-1-(((1*S*)-1-Cyclohexyl-2-((2*S*,4*S*)-4-(3-(2-((6-((6-((2-(2,6-dioxopiperidin-3-yl)-1,3-dioxoisoindolin-4-yl)amino)hexyl)oxy)hexyl)oxy)ethoxy)phenoxy)-2-((*R*-1,2,3,4-tetrahydronaphthalen-1-yl)carbamoylpyrrolidin-1-yl)-2-oxoethyl)amino)-1-oxopropan-2-yl)(methyl)carbamate (**131**)

This compound was prepared using general procedure **VIII**, **104** (95 mg, 103 μmol), and **72** (28 mg 98 μmol). The crude product was purified by column chromatography (CH_2_Cl_2_/MeOH 30:1) to give a yellow resin. Yield (86 mg, 71%); *R*_f_ = 0.18 (CH_2_Cl_2_/MeOH 20:1). Due to the presence of the *N*-Boc protecting group resulting in an additional set of rotamers, NMR data is only provided for the deprotected final PROTAC. HRMS (ESI) *m*/*z*: [M + H]^+^ calcd for C_65_H_90_O_13_N_7_, 1176.6591; found, 1176.6571.

##### *tert*-Butyl ((2*S*)-1-(((1*S*)-1-Cyclohexyl-2-((2*S*,4*S*)-4-(3-((5-((5-((6-((2-(2,6-dioxopiperidin-3-yl)-1,3-dioxoisoindolin-4-yl)amino)hexyl)oxy)pentyl)oxy)pentyl)oxy)phenoxy)-2-(((*R*)-1,2,3,4-tetrahydronaphthalen-1-yl)carbamoyl)pyrrolidin-1-yl)-2-oxoethyl)amino)-1-oxopropan-2-yl)(methyl)carbamate (**132**)

This compound was prepared using general procedure **VIII**, **105** (65 mg, 69 μmol), and **72** (21 mg, 69 μmol). The crude product was purified by column chromatography (CH_2_Cl_2_/MeOH 50:1) to give a yellow resin. Yield (47 mg, 57%); *R*_f_ = 0.45 (CH_2_Cl_2_/MeOH 9:1). Due to the presence of the *N*-Boc protecting group resulting in an additional set of rotamers, NMR data is only provided for the deprotected final PROTAC. HRMS (ESI) *m*/*z*: [M + H]^+^ calcd for C_67_H_94_O_13_N_7_, 1204.6904; found, 1204.6904.

##### *tert*-Butyl ((2*S*)-1-(((1*S*)-1-Cyclohexyl-2-((2*S*,4*S*)-4-(3-((6-((6-((6-((2-(2,6-dioxopiperidin-3-yl)-1,3-dioxoisoindolin-4-yl)amino)hexyl)oxy)hexyl)oxy)hexyl)oxy)phenoxy)-2-(((*R*)-1,2,3,4-tetrahydronaphthalen-1-yl)carbamoyl)pyrrolidin-1-yl)-2-oxoethyl)amino)-1-oxopropan-2-yl)(methyl)carbamate (**133**)

This compound was prepared using general procedure **VIII**, **106** (142 mg, 145 μmol), and **72** (40 mg, 145 μmol). The crude product was purified by column chromatography (CH_2_Cl_2_/MeOH 50:1) to give a yellow resin. Yield (51 mg, 29%); *R*_f_ = 0.35 (CH_2_Cl_2_/MeOH 20:1). Due to the presence of the *N*-Boc protecting group resulting in an additional set of rotamers, NMR data is only provided for the deprotected final PROTAC. HRMS (ESI) *m*/*z*: [M + H]^+^ calcd for C_69_H_98_O_13_N_7_, 1232.7217; found, 1232.7205.

##### *tert*-Butyl ((2*S*)-1-(((1*S*)-1-Cyclohexyl-2-((2S,4S)-4-(3-(2-((6-((6-((2-(1-methyl-2,6-dioxopiperidin-3-yl)-1,3-dioxoisoindolin-4-yl)amino)hexyl)oxy)hexyl)oxy)ethoxy)phenoxy)-2-(((*R*)-1,2,3,4-tetrahydronaphthalen-1-yl)carbamoyl)pyrrolidin-1-yl)-2-oxoethyl)amino)-1-oxopropan-2-yl)(methyl)carbamate (**134**)

This compound was prepared using general procedure **VIII**, **104** (92 mg, 100 μmol), and **73** (29 mg, 100 μmol). The crude product was purified by column chromatography (CH_2_Cl_2_/MeOH 20:1) to give a yellow resin. Yield (65 mg, 55%); *R*_f_ = 0.23 (CH_2_Cl_2_/MeOH 20:1). Due to the presence of the *N*-Boc protecting group resulting in an additional set of rotamers, NMR data is only provided for the deprotected final PROTAC. HRMS (ESI) *m*/*z*: [M + H]^+^ calcd for C_66_H_92_O_13_N_7_, 1190.6748; found, 1190.6743.

##### *tert*-Butyl ((2*S*)-1-(((1*S*)-1-cyclohexyl-2-((2*S*,4*S*)-4-(3-((6-((6-((6-((2-(1-methyl-2,6-dioxopiperidin-3-yl)-1,3-dioxoisoindolin-4-yl)amino)hexyl)oxy)hexyl)oxy)hexyl)oxy)phenoxy)-2-((*R*-1,2,3,4-tetrahydronaphthalen-1-yl)carbamoylpyrrolidin-1-yl)-2-oxoethyl)amino)-1-oxopropan-2-yl)(methyl)carbamate (**135**)

This compound was prepared using general procedure **VIII**, **106** (125 mg, 128 μmol), and **73** (37 mg, 128 μmol). The crude product was purified by column chromatography (CH_2_Cl_2_/MeOH 50:1) to give a yellow resin. Yield (83 mg, 52%); *R*_f_ = 0.30 (CH_2_Cl_2_/MeOH 20:1). Due to the presence of the *N*-Boc protecting group resulting in an additional set of rotamers, NMR data is only provided for the deprotected final PROTAC. HRMS (ESI) *m*/*z*: [M + H]^+^ calcd for C_70_H_100_O_13_N_7_, 1246.7374; found, 1246.7360.

##### (2*S*,4*S*)-1-((*S*)-2-Cyclohexyl-2-((*S*)-2-(methylamino)propanamido)acetyl)-4-(3-((5-((2-(2,6-dioxopiperidin-3-yl)-1,3-dioxoisoindolin-4-yl)amino)pentyl)oxy)phenoxy)-*N*-((*R*)-1,2,3,4-tetrahydronaphthalen-1-yl)pyrrolidine-2-carboxamide (PROTAC **20**)

This compound was prepared using general procedure **VII** and PROTAC precursor **126** (51 mg, 50 μmol). The crude product was purified by column chromatography (CH_2_Cl_2_/MeOH/NH_4_OH 9:1:0.1) to give a yellow solid. Yield (35 mg, 76%); *R*_f_ = 0.45 (CH_2_Cl_2_/MeOH/NH_4_OH 9:1:0.1); mp 64–69 °C; ^1^H NMR (400 MHz, CDCl_3_): δ 0.82–1.00 (m, 5H), 1.28 (d, *J* = 6.9 Hz, 3H), 1.43–1.68 (m, 8H), 1.70–1.85 (m, 6H), 1.97–2.14 (m, 2H), 2.35 (s, 3H), 2.69–2.78 (m, 4H), 2.82–2.90 (m, 2H), 3.02–3.10 (m, 1H), 3.30 (q, *J* = 6.6 Hz, 2H), 3.60–3.67 (m, 2H), 3.74–3.83 (m, 3H), 3.92 (t, *J* = 6.2 Hz, 1H), 4.22–4.31 (m, 1H), 4.39–4.47 (m, 1H), 4.71–4.78 (m, 1H), 4.84–4.96 (m, 2H), 5.08–5.16 (m, 1H), 6.25 (t, *J* = 5.7 Hz, 1H), 6.36–6.42 (m, 2H), 6.48–6.55 (m, 1H), 6.61–6.72 (m, 1H), 6.88 (d, *J* = 8.6 Hz, 1H), 7.02–7.18 (m, 5H), 7.29 (d, *J* = 7.3 Hz, 2H), 7.45–7.52 (m, 1H), 7.67 (d, *J* = 8.9 Hz, 1H); ^13^C NMR (101 MHz, CDCl_3_, only the peaks for the major rotamer are given): δ 19.65, 20.20, 22.93, 23.73, 25.66, 25.77, 26.03, 28.73, 29.04, 29.16, 29.42, 29.89, 30.12, 31.59, 33.57, 35.22, 40.63, 42.65, 43.03, 47.74, 49.02, 53.81, 54.96, 60.22, 61.86, 67.73, 71.27, 72.42, 102.90, 107.61, 108.50, 110.06, 111.57, 116.76, 126.31, 127.21, 128.86, 129.21, 130.22, 132.64, 136.27, 136.81, 137.57, 147.08, 158.09, 160.40, 167.77, 168.55, 169.65, 171.25, 172.69, 175.24; **HPLC** (95% H_2_O (with 0.1% TFA) to 95% MeCN in 10 min, then 95% MeCN for 4 min), *t*_R_ = 7.19 min, 95% purity, detection at 254 nm; HRMS (ESI) *m*/*z*: [M + H]^+^ calcd for C_51_H_64_O_9_N_7_, 918.4760; found, 918.4735.

##### (2*S*,4*S*)-1-((*S*)-2-Cyclohexyl-2-((*S*)-2-(methylamino)propanamido)acetyl)-4-(3-((8-((2-(2,6-dioxopiperidin-3-yl)-1,3-dioxoisoindolin-4-yl)amino)octyl)oxy)phenoxy)-*N*-((*R*)-1,2,3,4-tetrahydronaphthalen-1-yl)pyrrolidine-2-carboxamide (PROTAC **21**)

This compound was prepared using general procedure **VII** and PROTAC precursor **127** (54 mg, 51 μmol). The crude product was purified by column chromatography (CH_2_Cl_2_/MeOH/NH_4_OH 15:1:0.1) to give a yellow solid. Yield (42 mg, 86%); *R*_f_ = 0.55 (CH_2_Cl_2_/MeOH/NH_4_OH 9:1:0.1); mp 83–85 °C; ^1^H NMR (400 MHz, CDCl_3_): δ 0.83–1.00 (m, 5H), 1.27 (d, *J* = 6.9 Hz, 3H), 1.34–1.54 (m, 8H), 1.54–1.70 (m, 8H), 1.72–1.84 (m, 6H), 1.98–2.14 (m, 2H), 2.29–2.35 (m, 1H), 2.36 (s, 3H), 2.67–2.81 (m, 4H), 2.82–2.91 (m, 2H), 3.01–3.08 (m, 1H), 3.25 (q, *J* = 6.6 Hz, 2H), 3.81 (d, *J* = 11.5 Hz, 1H), 3.85–3.90 (m, 2H), 4.21–4.29 (m, 1H), 4.38–4.46 (m, 1H), 4.71–4.78 (m, 1H), 4.85–4.96 (m, 2H), 5.13 (q, *J* = 5.9 Hz, 1H), 6.22 (t, *J* = 5.5 Hz, 1H), 6.36–6.41 (m, 2H), 6.50–6.54 (m, 1H), 6.64 (d, *J* = 9.2 Hz, 1H), 6.87 (d, *J* = 8.5 Hz, 1H), 7.02–7.17 (m, 5H), 7.30 (d, *J* = 7.8 Hz, 1H), 7.45–7.50 (m, 1H), 7.63 (dd, *J* = 9.0, 3.0 Hz, 1H), 8.38 (br s, 1H); ^13^C NMR (101 MHz, CDCl_3_, only the peaks for the major rotamer are given): δ 19.70, 20.21, 22.93, 25.65, 25.77, 26.03, 26.08, 26.98, 28.69, 29.34, 29.43, 29.90, 30.14, 31.56, 33.62, 35.28, 40.67, 42.76, 47.75, 49.00, 53.76, 54.91, 60.24, 60.40, 68.03, 102.88, 103.03, 107.93, 108.17, 109.97, 111.48, 116.77, 126.34, 127.22, 128.90, 129.19, 130.18, 132.63, 136.24, 136.78, 137.54, 147.15, 158.08, 160.58, 167.77, 168.53, 169.66, 171.20, 172.74, 175.37; **HPLC** (95% H_2_O (with 0.1% TFA) to 95% MeCN in 10 min, then 95% MeCN for 4 min), *t*_R_ = 8.04 min, 97% purity, detection at 254 nm; HRMS (ESI) *m*/*z*: [M + H]^+^ calcd for C_54_H_70_O_9_N_7_, 960.5230; found, 960.5201.

##### (2*S*,4*S*)-1-((*S*)-2-Cyclohexyl-2-((*S*)-2-(methylamino)propanamido)acetyl)-4-(3-(4-(4-((2-(2,6-dioxopiperidin-3-yl)-1,3-dioxoisoindolin-4-yl)amino)butoxy)butoxy)phenoxy)-*N*-((*R*)-1,2,3,4-tetrahydronaphthalen-1-yl)pyrrolidine-2-carboxamide (PROTAC **22**)

This compound was prepared using general procedure **VII** and PROTAC precursor **128** (71 mg, 66 μmol). The crude product was purified by column chromatography (CH_2_Cl_2_/MeOH/NH_4_OH 15:1:0.1) to give a yellow solid. Yield (59 mg, 92%); *R*_f_ = 0.42 (CH_2_Cl_2_/MeOH/NH_4_OH 9:1:0.1); mp 80–81 °C; ^1^H NMR (400 MHz, CDCl_3_): δ 0.83–0.99 (m, 5H), 1.28 (d, *J* = 6.8 Hz, 3H), 1.39–1.65 (m, 7H), 1.65–1.88 (m, 12H), 1.99–2.13 (m, 2H), 2.35 (s, 3H), 2.67–2.89 (m, 6H), 3.02–3.10 (m, 1H), 3.30 (q, *J* = 6.5 Hz, 2H), 3.44–3.50 (m, 4H), 3.80 (d, *J* = 11.7 Hz, 1H), 3.91 (d, *J* = 5.4 Hz, 2H), 4.23–4.29 (m, 1H), 4.39–4.48 (m, 1H), 4.71–4.78 (m, 1H), 4.81–4.96 (m, 2H), 5.13 (q, *J* = 6.1 Hz, 1H), 6.26 (t, *J* = 5.7 Hz, 1H), 6.36–6.41 (m, 2H), 6.49–6.54 (m, 1H), 6.59–6.73 (m, 1H), 6.88 (d, *J* = 8.5 Hz, 1H), 7.02–7.16 (m, 5H), 7.30 (d, *J* = 7.1 Hz, 1H), 7.47 (d, *J* = 8.5 Hz, 1H), 7.61–7.70 (m, 1H), 8.52 (br s, 1H); ^13^C NMR (101 MHz, CDCl_3_, only the peaks for the major rotamer are given): δ 19.65, 20.20, 22.93, 25.66, 25.77, 26.04, 26.28, 26.42, 26.48, 27.21, 28.69, 29.42, 29.88, 30.13, 31.58, 33.62, 35.20, 40.68, 42.61, 47.73, 49.01, 53.75, 54.93, 60.22, 67.80, 70.40, 70.67, 76.35, 102.94, 103.07, 107.72, 108.36, 110.01, 111.50, 116.78, 126.32, 127.21, 128.93, 129.19, 130.18, 132.64, 136.25, 136.81, 137.56, 147.08, 158.10, 160.49, 167.78, 168.58, 169.67, 171.28, 172.71, 175.31; **HPLC** (95% H_2_O (with 0.1% TFA) to 95% MeCN in 10 min, then 95% MeCN for 4 min), *t*_R_ = 7.42 min, 97% purity, detection at 254 nm; HRMS (ESI) *m*/*z*: [M + H]^+^ calcd for C_54_H_70_O_10_N_7_, 976.5179; found, 976.5161.

##### (2*S*,4*S*)-1-((*S*)-2-Cyclohexyl-2-((*S*)-2-(methylamino)propanamido)acetyl)-4-(3-(2-(2-(2-((2-(2,6-dioxopiperidin-3-yl)-1,3-dioxoisoindolin-4-yl)amino)ethoxy)ethoxy)ethoxy)phenoxy)-*N*-((*R*)-1,2,3,4-tetrahydronaphthalen-1-yl)pyrrolidine-2-carboxamide (PROTAC **23**)

This compound was prepared using general procedure **VII** and PROTAC precursor **129** (47 mg, 44 μmol). The crude product was purified by column chromatography (CH_2_Cl_2_/MeOH/NH_4_OH 15:1:0.1) to give a yellow solid. Yield (40 mg, 94%); *R*_f_ = 0.40 (CH_2_Cl_2_/MeOH/NH_4_OH 9:1:0.1); mp 80–82 °C; ^1^H NMR (400 MHz, CDCl_3_): δ 0.82–1.01 (m, 5H), 1.28 (d, *J* = 6.9 Hz, 3H), 1.37–1.71 (m, 7H), 1.74–1.84 (m, 4H), 1.99–2.10 (m, 2H), 2.33 (s, 3H), 2.62–2.89 (m, 6H), 3.02–3.13 (m, 1H), 3.40–3.51 (m, 2H), 3.68–3.76 (m, 6H), 3.77–3.82 (m, 1H), 3.82–3.88 (m, 2H), 4.02–4.10 (m, 2H), 4.23–4.32 (m, 1H), 4.40–4.48 (m, 1H), 4.72–4.77 (m, 1H), 4.80–4.93 (m, 2H), 5.09–5.17 (m, 1H), 6.36–6.44 (m, 2H), 6.47–6.56 (m, 2H), 6.70 (s, 1H), 6.90 (dd, *J* = 8.5, 1.7 Hz, 1H), 7.01–7.17 (m, 5H), 7.27–7.32 (m, 1H), 7.45 (d, *J* = 7.8 Hz, 1H), 7.62–7.75 (m, 1H), 8.69 (br s, 1H); ^13^C NMR (101 MHz, CDCl_3_, only the peaks for the major rotamer are given): δ 14.26, 19.59, 20.19, 22.86, 25.66, 25.79, 26.03, 28.70, 29.42, 29.84, 30.11, 31.58, 33.57, 35.11, 40.60, 42.53, 47.69, 49.01, 53.76, 54.97, 60.18, 67.61, 69.69, 69.96, 70.97, 102.75, 107.69, 107.88, 110.40, 111.72, 116.94, 126.31, 127.21, 128.86, 129.20, 130.17, 132.61, 136.16, 136.87, 137.58, 146.97, 158.10, 160.22, 167.80, 168.62, 169.41, 169.68, 171.40, 172.67, 175.25; **HPLC** (95% H_2_O (with 0.1% TFA) to 95% MeCN in 10 min, then 95% MeCN for 4 min), *t*_R_ = 6.63 min, 95% purity, detection at 254 nm; HRMS (ESI) *m*/*z*: [M + H]^+^ calcd for C_52_H_66_11N_7_, 964.4815; found, 964.4799.

##### (2*S*,4*S*)-1-((*S*)-2-Cyclohexyl-2-((*S*)-2-(methylamino)propanamido)acetyl)-4-(3-(2-(2-(2-(2-((2-(2,6-dioxopiperidin-3-yl)-1,3-dioxoisoindolin-4-yl)amino)ethoxy)ethoxy)ethoxy)ethoxy)phenoxy)-*N*-((*R*)-1,2,3,4-tetrahydronaphthalen-1-yl)pyrrolidine-2-carboxamide (PROTAC **24**)

This compound was prepared using general procedure **VII** and PROTAC precursor **130** (30 mg, 27 μmol). The crude product was purified by column chromatography (CH_2_Cl_2_/MeOH/NH_4_OH 15:1:0.1) to give a yellow solid. Yield (22 mg, 81%); *R*_f_ = 0.35 (CH_2_Cl_2_/MeOH/NH_4_OH 9:1:0.1); mp 76–80 °C; ^1^H NMR (400 MHz, CDCl_3_): δ 0.81–1.02 (m, 5H), 1.28 (d, *J* = 6.9 Hz, 3H), 1.38–1.70 (m, 10H), 1.74–1.84 (m, 4H), 1.99–2.13 (m, 2H), 2.36 (s, 3H), 2.62–2.78 (m, 4H), 2.78–2.90 (m, 2H), 3.05 (q, *J* = 7.5 Hz, 1H), 3.44 (q, *J* = 5.5 Hz, 2H), 3.66–3.68 (m, 3H), 3.68–3.74 (m, 5H), 3.83 (t, *J* = 4.9 Hz, 2H), 4.05 (q, *J* = 4.7 Hz, 2H), 4.24–4.31 (m, 1H), 4.44 (t, *J* = 8.0 Hz, 1H), 4.72–4.78 (m, 1H), 4.84–4.94 (m, 2H), 5.09–5.16 (m, 1H), 6.38–6.43 (m, 2H), 6.48 (q, *J* = 5.4 Hz, 1H), 6.51–6.55 (m, 1H), 6.65 (d, *J* = 7.8 Hz, 1H), 6.91 (dd, *J* = 8.5, 2.3 Hz, 1H), 7.02–7.16 (m, 5H), 7.29 (d, *J* = 7.9 Hz, 1H), 7.44–7.50 (m, 1H), 7.65 (d, *J* = 8.7 Hz, 1H), 8.57 (br s, 1H); ^13^C NMR (101 MHz, CDCl_3_, only the peaks for the major rotamer are given): δ 19.67, 20.20, 22.91, 25.66, 25.78, 26.02, 28.68, 29.42, 29.88, 30.13, 31.57, 33.54, 35.24, 40.64, 42.57, 47.73, 49.02, 53.78, 54.93, 60.20, 60.35, 67.63, 69.69, 69.81, 70.83, 70.88, 70.96, 102.95, 107.73, 108.73, 110.40, 111.77, 116.97, 126.33, 127.23, 128.85, 129.21, 130.21, 132.63, 136.18, 136.81, 137.56, 147.00, 158.08, 160.19, 167.76, 168.59, 169.42, 169.66, 171.29, 172.72, 175.35; **HPLC** (95% H_2_O (with 0.1% TFA) to 95% MeCN in 10 min, then 95% MeCN for 4 min), *t*_R_ = 6.64 min, 98% purity, detection at 254 nm; HRMS (ESI) *m*/*z*: [M + H]^+^ calcd for C_54_H_70_O_12_N_7_, 1008.5077; found, 1008.5049.

##### (2*S*,4*S*)-1-((*S*)-2-Cyclohexyl-2-((*S*)-2-(methylamino)propanamido)acetyl)-4-(3-(2-((6-((6-((2-(2,6-dioxopiperidin-3-yl)-1,3-dioxoisoindolin-4-yl)amino)hexyl)oxy)hexyl)oxy)ethoxy)phenoxy)-*N*-((*R*)-1,2,3,4-tetrahydronaphthalen-1-yl)pyrrolidine-2-carboxamide (PROTAC **25**) (**SAB 141**)

This compound was prepared using general procedure **VII** and PROTAC precursor **131** (80 mg, 68 μmol). The crude product was purified by column chromatography (CH_2_Cl_2_/MeOH/NH_4_OH 15:1:0.1) to give a yellow solid. Yield (61 mg, 83%); *R*_f_ = 0.42 (CH_2_Cl_2_/MeOH/NH_4_OH 9:1:0.1); mp 70–73 °C; ^1^H NMR (400 MHz, CDCl_3_): δ 0.82–1.00 (m, 5H), 1.28 (d, *J* = 6.9 Hz, 3H), 1.34–1.45 (m, 10H), 1.51–1.71 (m, 13H), 1.72–1.84 (m, 4H), 1.98–2.15 (m, 2H), 2.36 (s, 3H), 2.67–2.80 (m, 4H), 2.82–2.90 (m, 2H), 3.01–3.07 (m, 1H), 3.25 (q, *J* = 5.9 Hz, 2H), 3.38 (dd, *J* = 6.6, 2.7 Hz, 4H), 3.51 (d, *J* = 6.7 Hz, 2H), 3.75 (t, *J* = 4.7 Hz, 2H), 3.81 (d, *J* = 11.6 Hz, 1H), 4.04 (t, *J* = 5.1 Hz, 2H), 4.26 (dd, *J* = 11.2, 4.8 Hz, 1H), 4.42 (t, *J* = 8.3 Hz, 1H), 4.75 (dt, *J* = 9.8, 2.5 Hz, 1H), 4.85–4.96 (m, 2H), 5.13 (q, *J* = 6.1 Hz, 1H), 6.22 (t, *J* = 5.6 Hz, 1H), 6.37–6.43 (m, 2H), 6.52–6.57 (m, 1H), 6.59–6.64 (m, 1H), 6.84–6.89 (m, 1H), 7.02–7.17 (m, 5H), 7.27–7.31 (m, 1H), 7.45–7.51 (m, 1H), 7.63 (d, *J* = 8.9 Hz, 1H), 8.35 (br s, 1H); ^13^C NMR (101 MHz, CDCl_3_, only the peaks for the major rotamer are given): δ 19.71, 20.21, 22.94, 25.65, 25.77, 26.01, 26.11, 26.22, 26.94, 28.68, 29.36, 29.42, 29.76, 29.78, 29.84, 29.90, 30.13, 31.57, 33.58, 35.29, 40.64, 42.73, 47.74, 49.00, 53.77, 54.92, 60.24, 60.40, 67.58, 69.25, 70.80, 71.00, 71.72, 103.08, 107.84, 108.52, 108.60, 109.97, 111.50, 116.78, 126.36, 127.24, 128.83, 129.21, 130.19, 132.63, 136.26, 136.77, 137.55, 147.14, 158.02, 160.28, 167.79, 168.54, 169.66, 171.19, 172.74, 175.39; **HPLC** (95% H_2_O (with 0.1% TFA) to 95% MeCN in 10 min, then 95% MeCN for 4 min), *t*_R_ = 8.26 min, 96% purity, detection at 254 nm; HRMS (ESI) *m*/*z*: [M + H]^+^ calcd for C_60_H_82_O_11_N_7_, 1076.6067; found, 1076.6042.

##### (2*S*,4*S*)-1-((*S*)-2-Cyclohexyl-2-((*S*)-2-(methylamino)propanamido)acetyl)-4-(3-((5-((5-((6-((2-(2,6-dioxopiperidin-3-yl)-1,3-dioxoisoindolin-4-yl)amino)hexyl)oxy)pentyl)oxy)pentyl)oxy)phenoxy)-*N*-((*R*)-1,2,3,4-tetrahydronaphthalen-1-yl)pyrrolidine-2-carboxamide (PROTAC **26**)

This compound was prepared using general procedure **VII** and PROTAC precursor **132** (47 mg, 39 μmol). The crude product was purified by column chromatography (CH_2_Cl_2_/MeOH/NH_4_OH 15:1:0.1) to give a yellow solid. Yield (35 mg, 81%); *R*_f_ = 0.20 (CH_2_Cl_2_/MeOH/NH_4_OH 15:1:0.1); mp 63–66 °C; ^1^H NMR (400 MHz, CDCl_3_): δ 0.81–0.99 (m, 5H), 1.27 (d, *J* = 6.9 Hz, 3H), 1.34–1.53 (m, 10H), 1.54–1.71 (m, 15H), 1.72–1.84 (m, 6H), 1.99–2.15 (m, 2H), 2.36 (s, 3H), 2.66–2.81 (m, 4H), 2.82–2.91 (m, 2H), 3.00–3.07 (m, 1H), 3.25 (d, *J* = 5.8 Hz, 2H), 3.36–3.44 (m, 8H), 3.81 (d, *J* = 11.4 Hz, 1H), 3.87 (d, *J* = 6.0 Hz, 2H), 4.24 (dd, *J* = 11.5, 4.9 Hz, 1H), 4.42 (d, *J* = 8.1 Hz, 1H), 4.75 (dd, *J* = 9.8, 2.1 Hz, 1H), 4.86–4.96 (m, 2H), 5.13 (q, *J* = 6.0 Hz, 1H), 6.21 (t, *J* = 5.6 Hz, 1H), 6.34–6.40 (m, 2H), 6.50–6.53 (m, 1H), 6.61 (dd, *J* = 8.4, 3.8 Hz, 1H), 6.87 (d, *J* = 8.5 Hz, 1H), 7.02–7.17 (m, 5H), 7.30 (d, *J* = 7.5 Hz, 1H), 7.48 (t, *J* = 7.0 Hz, 1H), 7.64 (d, *J* = 8.9 Hz, 1H), 8.38 (br s, 1H); ^13^C NMR (101 MHz, CDCl_3_, only the peaks for the major rotamer are given): δ 19.71, 20.21, 22.93, 25.64, 25.76, 26.01, 26.11, 26.94, 28.68, 29.24, 29.35, 29.42, 29.66, 29.72, 29.78, 29.91, 30.13, 31.56, 33.63, 35.29, 40.65, 42.73, 47.74, 48.99, 53.76, 54.91, 60.25, 60.40, 67.98, 70.84, 70.97, 76.37, 103.06, 107.83, 108.02, 108.07, 109.96, 111.49, 116.76, 126.34, 127.23, 128.83, 129.20, 130.16, 132.62, 136.24, 136.76, 137.53, 147.13, 158.04, 160.56, 167.77, 168.51, 169.66, 171.19, 172.73, 175.39; **HPLC** (95% H_2_O (with 0.1% TFA) to 95% MeCN in 10 min, then 95% MeCN for 4 min), *t*_R_ = 8.60 min, 99% purity, detection at 254 nm; HRMS (ESI) *m*/*z*: [M + H]^+^ calcd for C_62_H_86_O_11_N_7_, 1104.6380; found, 1104.6396.

##### (2*S*,4*S*)-1-((*S*)-2-Cyclohexyl-2-((*S*)-2-(methylamino)propanamido)acetyl)-4-(3-((6-((6-((6-((2-(2,6-dioxopiperidin-3-yl)-1,3-dioxoisoindolin-4-yl)amino)hexyl)oxy)hexyl)oxy)hexyl)oxy)phenoxy)-*N*-((*R*)-1,2,3,4-tetrahydronaphthalen-1-yl)pyrrolidine-2-carboxamide (PROTAC **27**) (**SAB142**)

This compound was prepared using general procedure **VII** and PROTAC precursor **133** (50 mg, 41 μmol). The crude product was purified by column chromatography (CH_2_Cl_2_/MeOH/NH_4_OH 15:1:0.1) to give a yellow solid. Yield (45 mg, 97%); *R*_f_ = 0.25 (CH_2_Cl_2_/MeOH/NH_4_OH 15:1:0.1); mp 62–64 °C; ^1^H NMR (400 MHz, CDCl_3_): δ 0.80–0.99 (m, 5H), 1.28 (d, *J* = 6.9 Hz, 3H), 1.33–1.51 (m, 12H), 1.51–1.70 (m, 17H), 1.72–1.84 (m, 6H), 1.99–2.07 (m, 1H), 2.08–2.15 (m, 1H), 2.36 (s, 3H), 2.69–2.81 (m, 4H), 2.83–2.91 (m, 2H), 3.05 (q, *J* = 6.8 Hz, 1H), 3.25 (d, *J* = 6.2 Hz, 2H), 3.38 (dd, *J* = 6.6, 2.5 Hz, 8H), 3.82 (d, *J* = 11.0 Hz, 1H), 3.87 (d, *J* = 6.5 Hz, 2H), 4.24 (dd, *J* = 11.5, 4.8 Hz, 1H), 4.43 (d, *J* = 8.2 Hz, 1H), 4.71–4.78 (m, 1H), 4.86–4.97 (m, 2H), 5.13 (d, *J* = 6.1 Hz, 1H), 6.22 (d, *J* = 5.6 Hz, 1H), 6.34–6.41 (m, 2H), 6.49–6.54 (m, 1H), 6.59–6.65 (m, 1H), 6.87 (d, *J* = 8.6 Hz, 1H), 7.02–7.17 (m, 5H), 7.27–7.31 (m, 1H), 7.48 (dd, *J* = 8.5, 7.1 Hz, 1H), 7.65 (d, *J* = 8.9 Hz, 1H), 8.31 (br s, 1H); ^13^C NMR (101 MHz, CDCl_3_, only the peaks for the major rotamer are given): δ 19.68, 20.21, 22.94, 25.65, 25.76, 26.02, 26.11, 26.16, 26.23, 26.96, 28.69, 29.37, 29.43, 29.80, 29.85, 29.91, 30.14, 31.57, 33.66, 35.25, 40.66, 42.74, 47.75, 49.00, 53.77, 54.93, 60.27, 60.38, 68.05, 70.82, 70.94, 71.02, 71.05, 76.38, 103.08, 107.86, 108.02, 109.97, 111.50, 116.77, 126.35, 127.24, 128.84, 129.21, 130.17, 132.63, 136.25, 136.76, 137.54, 147.14, 158.04, 160.59, 167.78, 168.51, 169.64, 169.68, 171.17, 172.73, 175.32; **HPLC** (95% H_2_O (with 0.1% TFA) to 95% MeCN in 10 min, then 95% MeCN for 4 min), *t*_R_ = 9.08 min, 99% purity, detection at 254 nm; HRMS (ESI) *m*/*z*: [M + H]^+^ calcd for C_64_H_90_O_11_N_7_, 1132.6693; found, 1132.6669.

##### (2*S*,4*S*)-1-((*S*)-2-Cyclohexyl-2-((*S*)-2-(methylamino)propanamido)acetyl)-4-(3-(2-((6-((6-((2-(1-methyl-2,6-dioxopiperidin-3-yl)-1,3-dioxoisoindolin-4-yl)amino)hexyl)oxy)hexyl)oxy)ethoxy)phenoxy)-*N*-((*R*)-1,2,3,4-tetrahydronaphthalen-1-yl)pyrrolidine-2-carboxamide (PROTAC **28**)

This compound was prepared using general procedure **VII** and PROTAC precursor **134** (65 mg, 55 μmol). The crude product was purified by column chromatography (CH_2_Cl_2_/MeOH/NH_4_OH 9:1:0.1) to give a yellow solid. Yield (41 mg, 69%); *R*_f_ = 0.30 (CH_2_Cl_2_/MeOH/NH_4_OH 9:1:0.1); mp 58–61 °C; ^1^H NMR (400 MHz, CDCl_3_): δ 0.79–0.99 (m, 5H), 1.28 (d, *J* = 6.9 Hz, 3H), 1.33–1.49 (m, 10H), 1.57 (s, 13H), 1.74–1.84 (m, 4H), 2.00–2.12 (m, 2H), 2.36 (s, 3H), 2.69–2.81 (m, 4H), 2.89 (d, *J* = 14.5 Hz, 1H), 2.94–3.00 (m, 1H), 3.01–3.07 (m, 1H), 3.21 (s, 3H), 3.25 (q, *J* = 6.7 Hz, 2H), 3.39 (td, *J* = 6.6, 2.7 Hz, 4H), 3.51 (t, *J* = 6.7 Hz, 2H), 3.73–3.77 (m, 2H), 3.82 (d, *J* = 11.4 Hz, 1H), 4.04 (d, *J* = 4.9 Hz, 2H), 4.25 (dd, *J* = 11.5, 4.8 Hz, 1H), 4.41 (t, *J* = 8.2 Hz, 1H), 4.76 (dd, *J* = 9.8, 2.1 Hz, 1H), 4.87–4.96 (m, 2H), 5.13 (q, *J* = 7.0 Hz, 1H), 6.22 (t, *J* = 5.6 Hz, 1H), 6.37–6.44 (m, 2H), 6.53–6.57 (m, 1H), 6.59 (d, *J* = 8.5 Hz, 1H), 6.87 (d, *J* = 8.5 Hz, 1H), 7.03–7.19 (m, 5H), 7.29 (d, *J* = 7.7 Hz, 1H), 7.45–7.51 (m, 1H), 7.64 (d, *J* = 8.9 Hz, 1H); ^13^C NMR (101 MHz, CDCl_3_, only the peaks for the major rotamer are given): δ 19.71, 20.20, 22.29, 25.64, 25.76, 26.01, 26.12, 26.22, 26.98, 27.40, 28.70, 29.39, 29.43, 29.77, 29.81, 29.86, 29.91, 30.14, 32.07, 33.59, 35.30, 40.64, 42.75, 47.74, 49.76, 53.80, 54.92, 60.24, 60.41, 67.57, 69.24, 70.85, 71.04, 71.74, 76.41, 103.15, 107.91, 108.50, 110.07, 111.44, 116.71, 126.37, 127.26, 128.81, 129.22, 130.20, 132.69, 136.19, 136.77, 137.54, 147.11, 157.98, 160.29, 167.94, 169.18, 169.61, 169.83, 171.40, 172.76, 175.27; **HPLC** (95% H_2_O (with 0.1% TFA) to 95% MeCN in 10 min, then 95% MeCN for 4 min), *t*_R_ = 8.53 min, 97% purity, detection at 254 nm; HRMS (ESI) *m*/*z*: [M + H]^+^ calcd for C_61_H_84_O_11_N_7_, 1090.6223; found, 1090.6203.

##### (2*S*,4*S*)-1-((*S*)-2-Cyclohexyl-2-((*S*)-2-(methylamino)propanamido)acetyl)-4-(3-((6-((6-((6-((2-(1-methyl-2,6-dioxopiperidin-3-yl)-1,3-dioxoisoindolin-4-yl)amino)hexyl)oxy)hexyl)oxy)hexyl)oxy)phenoxy)-*N*-((*R*)-1,2,3,4-tetrahydronaphthalen-1-yl)pyrrolidine-2-carboxamide (PROTAC **29**)

This compound was prepared using general procedure **VII** and PROTAC precursor **135** (82 mg, 66 μmol). The crude product was purified by column chromatography (CH_2_Cl_2_/MeOH/NH_4_OH 20:1:0.1) to give a yellow solid. Yield (44 mg, 58%); *R*_f_ = 0.30 (CH_2_Cl_2_/MeOH/NH_4_OH 20:1:0.1); mp 56–60 °C; ^1^H NMR (400 MHz, CDCl_3_): δ 0.80–0.98 (m, 5H), 1.28 (d, *J* = 6.9 Hz, 3H), 1.32–1.49 (m, 12H), 1.50–1.71 (m, 18H), 1.72–1.84 (m, 6H), 2.00–2.12 (m, 2H), 2.36 (s, 3H), 2.70–2.81 (m, 4H), 2.85–2.93 (m, 1H), 2.94–2.99 (m, 1H), 3.00–3.05 (m, 1H), 3.20 (s, 3H), 3.22–3.30 (m, 1H), 3.38 (dd, *J* = 6.6, 2.6 Hz, 8H), 3.78–3.84 (m, 1H), 3.87 (t, *J* = 6.5 Hz, 2H), 4.23 (dd, *J* = 11.5, 4.8 Hz, 1H), 4.41 (d, *J* = 8.4 Hz, 1H), 4.76 (dd, *J* = 9.8, 2.1 Hz, 1H), 4.87–4.98 (m, 2H), 5.13 (d, *J* = 6.2 Hz, 1H), 6.22 (d, *J* = 5.6 Hz, 1H), 6.35–6.40 (m, 2H), 6.51–6.55 (m, 1H), 6.59 (d, *J* = 8.3 Hz, 1H), 6.87 (d, *J* = 8.5 Hz, 1H), 7.02–7.17 (m, 5H), 7.27–7.33 (m, 1H), 7.45–7.52 (m, 1H), 7.65 (d, *J* = 8.9 Hz, 1H); ^13^C NMR (101 MHz, CDCl_3_, only the peaks for the major rotamer are given): δ 19.59, 20.07, 22.16, 25.51, 25.62, 25.88, 25.98, 26.03, 26.03, 26.10, 26.85, 27.26, 28.56, 29.25, 29.30, 29.68, 29.74, 29.79, 30.00, 31.94, 33.52, 35.18, 40.53, 42.62, 47.62, 49.63, 53.67, 54.77, 60.14, 60.28, 67.92, 70.71, 70.82, 70.91, 76.29, 102.99, 107.76, 107.86, 111.31, 116.57, 126.23, 127.12, 128.68, 129.08, 130.04, 132.55, 136.06, 136.61, 137.40, 146.98, 157.88, 160.47, 167.81, 169.04, 169.50, 169.69, 171.26, 172.63, 175.17; **HPLC** (95% H_2_O (with 0.1% TFA) to 95% MeCN in 10 min, then 95% MeCN for 4 min), *t*_R_ = 8.40 min, 97% purity, detection at 254 nm; HRMS (ESI) *m*/*z*: [M + H]^+^ calcd for C_65_H_92_O_11_N_7_, 1146.6849; found, 1146.6812.

### Cell Lines

All cell lines were obtained from ATCC (Manassas, Virginia, USA) and the German Collection of Microorganisms and Cell Cultures GmbH (DSMZ, Braunschweig, Germany) and maintained in RPMI-1640 medium (Merck KGaA, Darmstadt, Germany) containing 10% fetal bovine serum and supplemented with 1% penicillin/streptomycin and 1% l-glutamine. NCI-H929 cells were cultured in media supplemented with 2-mercaptoethanol and sodium pyruvate. Cells were maintained at 37 °C with 5% CO_2_ in a humidified atmosphere.

For generation of lentiviral vectors, HEK293T cells were transfected with constructs along with the packaging and envelope vectors. Viral supernatants were harvested 48 h after transfection and were used to transduce cell lines. MM.1S and HG3 cells were transduced with virus containing pLKO5d.SSF.SpCas9.P2a.BSD, and cells were selected with blasticidin. Selected cells were then transduced with respective sgRNA constructs targeting VHL, cIAP1, cIAP2, XIAP, and negative control luciferase, which were cloned into pLKO5.hU6.sgRNA.dTom. Transduction success was confirmed through FACS analysis 48 h post-transduction with a minimum efficiency of 95% tomato fluorescence.

### Immunoblotting

Cells were treated with respective drugs for 16 h, and treated cells were washed and lysed in Pierce IP lysis buffer. SDS-PAGE was performed, and proteins were then transferred onto PVDF membranes. Blotted membranes were blocked with 5% milk in Tris-buffered saline/Tween20 (TBST). Primary antibodies were diluted in 5% BSA in TBST, and incubations were performed overnight at 4 °C. Secondary HRP-conjugated antibodies diluted in 5% milk were incubated for 1 h at room temperature. Detection of proteins on PVDF was carried out using the WesternBright ECL HRP substrate or the WesternBright Sirius HRP substrate (Advansta, San Jose, USA) and imaged with LAS 4000× (Fujifilm). Membranes were subjected to 10 min incubation with Restore Western Blot Stripping Buffer (Thermo Fisher Scientific, Waltham, USA), followed by TBST washes. After brief re-activation with methanol, the membranes were blocked, and further probing of proteins was carried out.

### Reagents and Antibodies

LCL-161, AZD5582, birinapant, and BV6 were obtained from MedChemExpress. Human TNF-α was obtained from Miltenyi Biotec. MG132, MLN4924, and MLN7243 were purchased from SelleckChem.

Primary antibodies used for immunoblotting include BIRC2 (BioRad; VMA00532; clone AB01/3B4), cIAP2 (Cell Signaling; 3130S; clone 58C7), XIAP (Cell Signaling; 14334S; clone D2Z8W), VHL (Cell Signaling; 68547S), CRBN (Sigma-Aldrich; SAB2106014), Ikaros (Cell Signaling; 14859S; clone D6N9Y), Aiolos (Cell Signaling; 15103S; clone D1C1E), HIF-1α (BD BioSciences, 610958; clone 54), α-tubulin (Sigma-Aldrich; T5168; clone B512), and beta-actin (Sigma-Aldrich; A1978). Secondary antibodies include anti-rabbit IgG HRP-linked antibody (Cell Signaling; 7074) and anti-mouse IgG HRP-linked antibody (Cell Signaling; 7076).

### Cell Viability Assays

Cells were seeded in 384-well plates with respective treatments, and plates were incubated at 37 °C for 96 h. Viability assays were performed with or without the addition of TNF-α at 1 ng/mL. The cell viability readout was measured using the CellTiter-Glo Luminescent Cell Viability Assay (Promega, Madison, USA) and measured with a Synergy LX Multi-Mode plate reader (BioTek, Vermont, USA). All conditions were normalized to the DMSO-treated control. Data represents the mean ± SD of biological triplicates.

### Statistical Analysis

Statistical and graphical analyses of cell viability experiments were performed with Prism version 9.1.0 (GraphPad Software, San Diego, CA, USA). Quantification of blots was performed using ImageJ software (National Institutes of Health).

### diaPASEF-Based Proteomics

#### Sample Preparation LFQ Quantitative Mass Spectrometry

MM1s cells were treated with DMSO or 0.1 μM of compound for 3 h. The cells were harvested by centrifugation and washed with phosphate-buffered saline before snap-freezing in liquid nitrogen. The cells were lysed by addition of lysis buffer (8 M urea, 50 mM NaCl, 50 mM 4-(2-hydroxyethyl)-1-piperazine-ethanesulfonic acid (EPPS) pH 8.5, protease, and phosphatase inhibitors) and homogenization by bead beating (BioSpec) for three repeats of 30 s at 2400. Bradford assay was used to determine the final protein concentration in the clarified cell lysate. 50 μg of protein for each sample was reduced, alkylated, and precipitated using methanol/chloroform as previously described,^64^ and the resulting washed precipitated protein was allowed to air dry. The precipitated protein was resuspended in 4 M urea, 50 mM HEPES pH 7.4, followed by dilution to 1 M urea with the addition of 200 mM EPPS, pH 8. Proteins were first digested with LysC (1:50; enzyme/protein) for 12 h at RT. The LysC digestion was diluted to 0.5 M urea with 200 mM EPPS pH 8, followed by digestion with trypsin (1:50; enzyme/protein) for 6 h at 37 °C. Sample digests were acidified with formic acid to a pH of 2–3 prior to desalting using C18 solid-phase extraction plates (SOLA, Thermo Fisher Scientific). Desalted peptides were dried in a vacuum centrifuge and reconstituted in 0.1% formic acid for LC–MS analysis.

Data were collected using a TimsTOF Pro2 (Bruker Daltonics, Bremen, Germany) coupled to a nanoElute LC pump (Bruker Daltonics, Bremen, Germany) via a CaptiveSpray nanoelectrospray source. Peptides were separated on a reversed-phase C_18_ column (25 cm × 75 μm ID, 1.6 μM, IonOpticks, Australia) containing an integrated captive spray emitter. Peptides were separated using a 50 min gradient of 2–30% buffer B (acetonitrile in 0.1% formic acid) with a flow rate of 250 nL/min and column temperature maintained at 50 °C.

Data-Dependent Acquisition (DDA) was performed in the Parallel Accumulation-Serial Fragmentation (PASEF) mode to determine effective ion mobility windows for downstream diaPASEF data collection.^54^ The diaPASEF parameters included 100% duty cycle using accumulation and ramp times of 50 ms each, 1 TIMS-MS scan, and 10 PASEF ramps per acquisition cycle. The TIMS-MS survey scan was acquired between 100–1700 *m*/*z* and 1/*K*_0_ of 0.7–1.3 V × s/cm^2^. Precursors with 1–5 charges were selected, and those that reached an intensity threshold of 20,000 arbitrary units were actively excluded for 0.4 min. The quadrupole isolation width was set to 2 *m*/*z* for *m*/*z* < 700 and 3 *m*/*z* for *m*/*z* > 800, with the *m*/*z* between 700 and 800 *m*/*z* being interpolated linearly. The TIMS elution voltages were calibrated linearly with three points (Agilent ESI-L Tuning Mix Ions; 622, 922, 1222 *m*/*z*) to determine the reduced ion mobility coefficients (1/*K*_0_). To perform diaPASEF, the precursor distribution in the DDA *m*/*z*-ion mobility plane was used to design an acquisition scheme for DIA data collection, which included two windows in each 50 ms diaPASEF scan. Data was acquired using 16 of these 25 Da precursor double window scans (creating 32 windows), which covered the diagonal scan line for doubly and triply charged precursors, with singly charged precursors able to be excluded by their position in the *m*/*z*-ion mobility plane. These precursor isolation windows were defined between 400–1200 *m*/*z* and 1/*K*_0_ of 0.7–1.3 V × s/cm^2^.

#### LC–MS Data Analysis

The diaPASEF raw file processing and controlling peptide and protein level false discovery rates, assembling proteins from peptides, and protein quantification from peptides were performed using library-free analysis in DIA-NN 1.8.^65^ The library-free mode performs an in silico digestion of a given protein sequence database alongside deep learning-based predictions to extract the DIA precursor data into a collection of MS^2^ spectra. The search results are then used to generate a spectral library which is then employed for the targeted analysis of the DIA data searched against the Swiss-Prot human database (January 2021). Database search criteria largely followed the default settings for directDIA including tryptic with two missed cleavages, carbamidomethylation of cysteine, and oxidation of methionine and precursor *Q*-value (FDR) cut-off of 0.01. The precursor quantification strategy was set to Robust LC (high accuracy) with RT-dependent cross-run normalization. Proteins with poor-quality data were excluded from further analysis (summed abundance across channels of <100 and the mean number of precursors used for quantification <2), and proteins with missing values were imputed by random selection from a Gaussian distribution either with a mean of the non-missing values for that treatment group or with a mean equal to the median of the background (in cases when all values for a treatment group are missing). Protein abundances were scaled using in-house scripts in the R framework (R Development Core Team, 2014), and statistical analysis was carried out using the limma package within the R framework.

#### Imputation Description

Protein level data output from diaNN was read into R and processed using in-house scripts. Summary statistics were calculated for the replicates of each protein condition group. Missing values for each group were imputed by random selection from a Gaussian distribution with a mean of the non-missing values for that group. For protein condition groups missing all values, the values were imputed by random selection from a Gaussian distribution with a mean equal to the median of the background, defined as the lowest 1% of the dataset. The standard deviation of each distribution was based on the global relative standard deviation of the dataset, and each distribution was truncated to have a minimum value of 100 and a maximum of 1.2 times the maximum value in the entire dataset.

### Molecular Descriptor Calculations

Predicted values for the topological polar surface area (TPSA) were calculated using MarvinSketch 17.28.0 (ChemAxon). Predicted values for the number of rotatable bonds were obtained using LigandScout 4.4.3.

### Log *D* Measurements

The determination of the log *D*_7.4_ values was performed by a chromatographic method as described previously.^60,66^ The system was calibrated by plotting the retention times of six different drugs (atenolol, metoprolol, labetalol, diltiazem, triphenylene, and permethrin) versus their literature-known log *D*_7.4_ in a calibration line (*R*^2^ = 0.99). Subsequently, the mean retention times of the analytes were taken to calculate their log *D*_7.4_ values with the aid of the calibration line. At least two independent measurements of each analyte were performed.

### Plasma Protein Binding Studies

Plasma protein binding (% PPB) was estimated by correlating the logarithmic retention times of the analytes on a CHIRALPAK HSA 50 × 3 mm, 5 μm column with the literature-known % PPB values (converted into log *K* values) of the following drugs: warfarin, ketoprofen, budesonide, nizatidine, indomethacin, acetylsalicylic acid, carbamazepine, piroxicam, nicardipine, and cimetidine (for details, see Valko et al.).^67^ The samples were dissolved in MeCN/DMSO 9:1 to achieve a final concentration of 0.5 mg/mL. Mobile phase A was 50 mM ammonium acetate adjusted to pH 7.4 with ammonia solution, while mobile phase B was *i*PrOH. The flow rate was set to 1.0 mL/min, the UV detector was set to 254 nm, and the column temperature was kept at 30 °C. After injecting 2 μL of the sample, a linear gradient from 100% A to 30% *i*PrOH in 5.4 min was applied. From 5.4 to 18 min, 30% *i*PrOH was kept, followed by switching back to 100% A in 1.0 min and a re-equilibration time of 6 min. With the aid of the calibration line (*R*^2^ = 0.96), the log *K* values of new substances were calculated and converted to their % PPB values. At least two independent measurements of each analyte were performed.

## Supporting Information Available

Overview on physicochemical properties of compounds, selected NMR, HPLC, and MS spectra (PDF)
Recommended compound characterization checklist (XLSX)
Molecular formula strings (CSV)
Hitlist AZD5582 vs CTRLs (CSV)
Hitlist PROTAC **9** vs CTRLs (CSV)
Hitlist PROTAC **25** vs CTRLs (CSV)
Hitlist PROTAC **27** vs CTRLs (CSV)
Normalized raw abundances for **27** and pomalidomide (XLSX)
Raw precursor abundances for **27** and pomalidomide (XLSX)
Normalized raw abundances for AZD5582, **9**, **25**, and pomalidomide (XLSX)
Raw precursor abundances for AZD5582, **9**, **25**, and pomalidomide (XLSX)

## Notes

A preprint version of this study was posted on ChemRxiv preprint server.^68^ While this manuscript was under consideration for publication, related pan-IAP degraders hijacking CRBN were published by S. Park and colleagues.^69^

## Abbreviations

AML: acute myeloid leukemia
BAIB: (diacetoxyiodo)benzene
BIR: baculoviral IAP repeat
cIAP: cellular IAP
CRBN: cereblon
DCC: *N*,*N*′-dicyclohexylcarbodiimide
DEAD: diethyl azodicarboxylate
DIPEA: *N*,*N*-diisopropylethylamine
DLBCL: diffuse large B-cell lymphoma
DMAP: 4-dimethylaminopyridine
DMF: dimethylformamide
DMSO: dimethyl sulfoxide
HATU: 1-[bis(dimethylamino)methylene]-1*H*-1,2,3-triazolo[4,5-*b*]pyridinium 3-oxide hexafluorophosphate
HIF: hypoxia-inducible factor
HPLC: high-performance liquid chromatography
IAP: inhibitor of apoptosis
IKZF1: zinc finger protein Ikaros
IKZF3: zinc finger protein Aiolos
LC–MS: liquid chromatography–mass spectrometry
MDM2: murine double minute 2
MM: multiple myeloma
NMR: nuclear magnetic resonance
PROTAC: proteolysis targeting chimera
PS-TPP: polymer-bound triphenylphosphine
RING: really interesting new gene
SMAC: second mitochondria-derived activator of caspases
TBAHS: tetrabutylammonium hydrogen sulfate
TEMPO: (2,2,6,6-tetramethylpiperidin-1-yl)oxyl
TFA: trifluoroacetic acid
THP: tetrahydropyranyl
TLC: thin-layer chromatography
TNF-α: tumor necrosis factor alpha
VHL: von Hippel-Lindau
XIAP: X-chromosome-linked IAP
