# Supplementary material for: Heterobifunctional Ligase Recruiters Enable pan-Degradation of Inhibitor of Apoptosis Proteins
Source: J Med Chem. 2023 Mar 30;66(7):4703–33. doi: 10.1021/acs.jmedchem.2c01817 (PMC10108347; doi:10.1021/acs.jmedchem.2c01817)
Supplement: Supplementary file 1 — jm2c01817_si_001.pdf [file jm2c01817_si_001.pdf]

Supplementary Information

Heterobifunctional Ligase Recruiters Enable Pan-Degradation of Inhibitor of Apoptosis Proteins

Yuen Lam Dora Ng,+[a] Aleša Bricelj,+[b] Jacqueline A. Jansen,[a]
Arunima Murgai,[a,c] Kirsten Peter,[a] Katherine A. Donovan,[d,e]
Michael Gütschow,[f] Jan Krönke,[a,c] Christian Steinebach,*[f] Izidor Sosič*[b]

[a] Department of Hematology, Oncology and Cancer Immunology, Charité - Universitätsmedizin Berlin, corporate member of Freie Universität Berlin and Humboldt-Universität zu Berlin, D-12203 Berlin, Germany.

[b] Faculty of Pharmacy, University of Ljubljana, Aškerčeva cesta 7, SI-1000 Ljubljana, Slovenia.

[c] German Cancer Consortium (DKTK) partner site Berlin and German Cancer Research Center (DKFZ), D- 69120 Heidelberg, Germany

[d] Department of Cancer Biology, Dana-Farber Cancer Institute, Boston, MA 02215, USA

[e] Biological Chemistry and Molecular Pharmacology, Harvard Medical School, Boston, MA 02215, USA

[f] Pharmaceutical Institute, Department of Pharmaceutical & Medicinal Chemistry, University of Bonn, An der Immenburg 4, D-53121 Bonn, Germany.

Table of Contents

Supplementary Tables, Schemes, and Figures 3

Selected NMR and MS spectra 34

References...78

Table of Intermediates...79

Supplementary Tables, Schemes, and Figures

	E3 ligase ligands	Negative control ligands
IAP	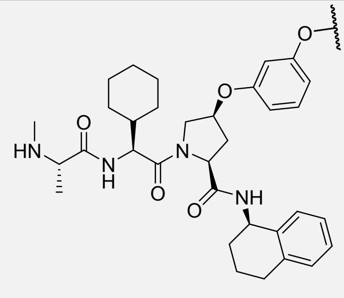<br>IAP ligand	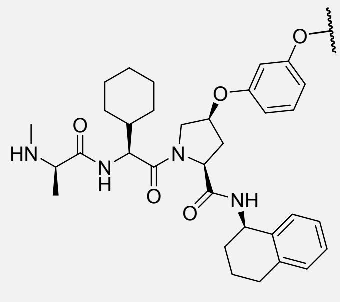 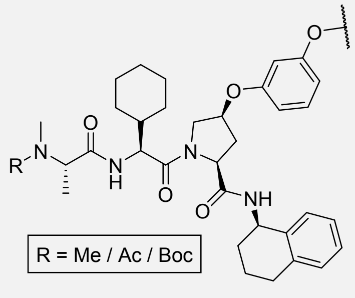<br>(–) IAP ligands
VHL1 Series	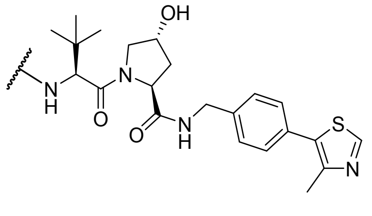<br>VHL1 ligand	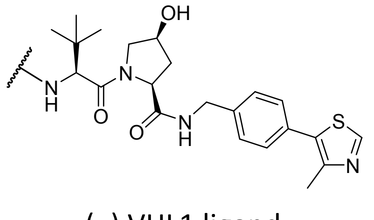<br>(–) VHL1 ligand
	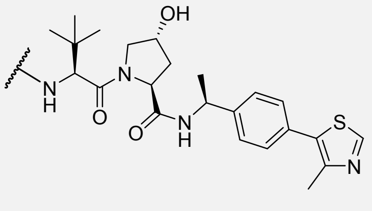<br>Me-VHL1 ligand	--
VHL2 Series	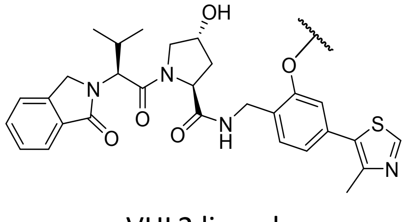<br>VHL2 ligand	--
CRBN Series	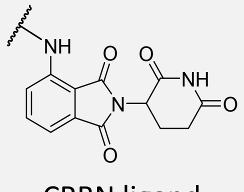<br>CRBN ligand	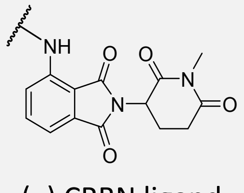<br>(–) CRBN ligand

Table S1. Overview of different E3 ligase ligands incorporated into final hetero-PROTACs.

Linker	Pattern	Structure
**L1a**	5	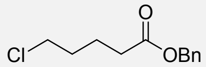
**L2a**	8	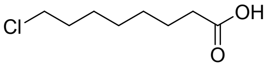
**L3a**	4-4	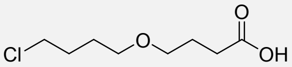
**L4a**	2-2-2	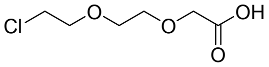
**L5a**	2-2-2-2	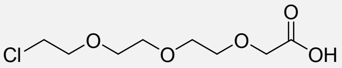
**L6a**	6-6-2	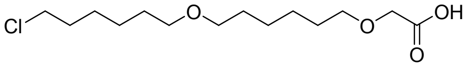
**L7a**	6-5-5	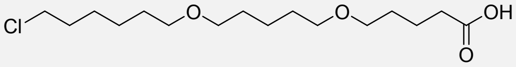
**L8a**	6-6-6	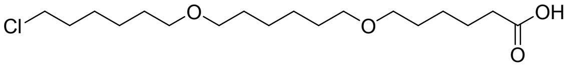

Table S2. Structures of linkers used in the IAP-VHL Series 1 PROTACs.

Linker	Pattern	Structure
**L1b**	5	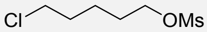
**L2b**	8	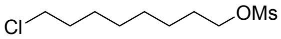
**L3b**	4-4	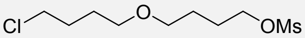
**L4b**	2-2-2	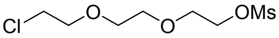
**L5b**	2-2-2-2	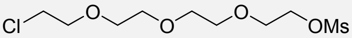
**L6b**	6-6-2	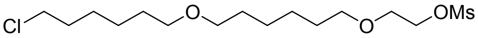
**L7b**	6-5-5	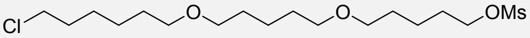
**L8b**	6-6-6	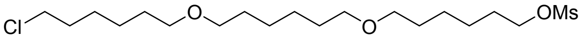

Table S3. Structures of linkers used in the IAP-VHL Series 2 and IAP-CRBN Series PROTACs.

Cmpd	MW (g/mol)	logD[a]	PPB[b] (%)	TPSA[c] ($Å^2$)	NRotB[d]	HBD[e]	HBA[f]	Structure
**1**	1089	3.8	96	249	29	6	11	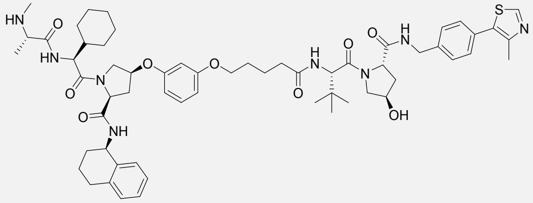
**2**	1131	4.4	96	249	32	6	11	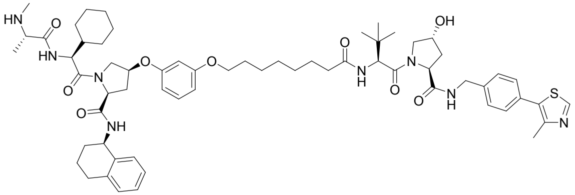
**3**	1147	4.0	95	258	33	6	12	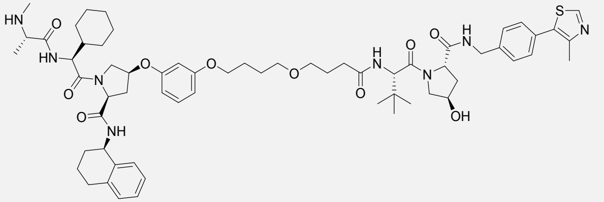
**4**	1135	3.7	95	267	32	6	13	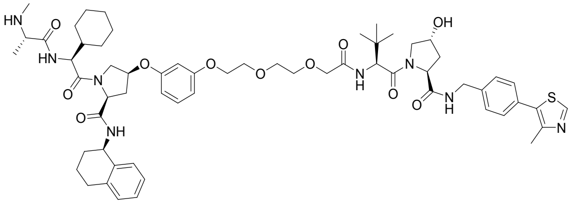
**5**	1179	3.7	94	277	35	6	14	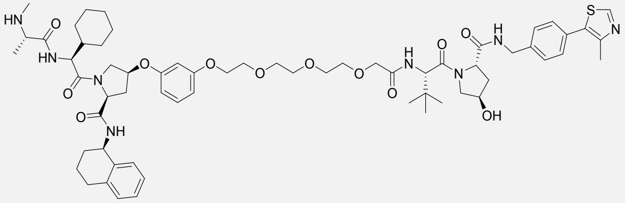

**6**	1247	5.5	n.d.	267	40	6	13	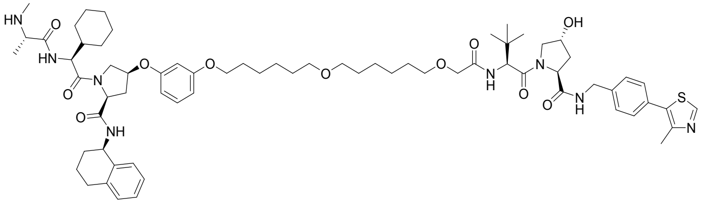
**7**	1276	5.3	96	267	42	6	13	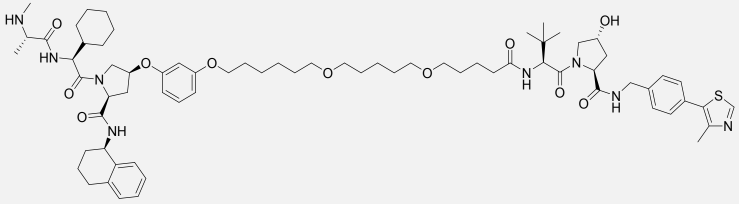
**8**	1304	5.8	n.d.[g]	267	44	6	13	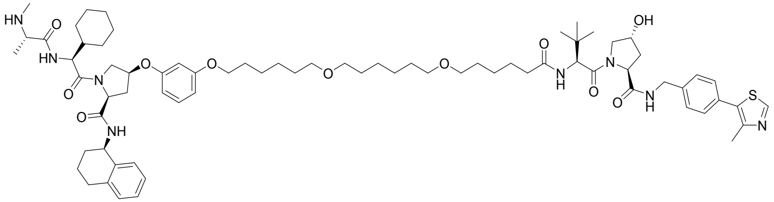
**9 (CST626)**	1103	3.4	95	249	30	6	11	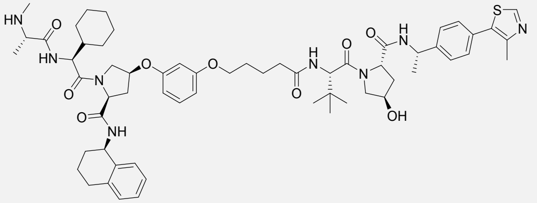
**10a**	1103	3.2	n.d.	249	30	6	11	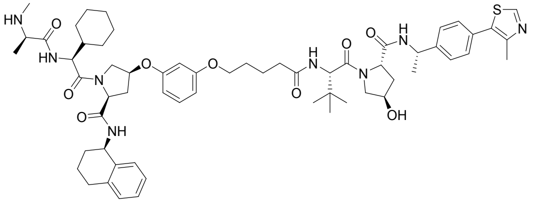

**10b**	1117	n.d.	n.d.	212	31	5	10	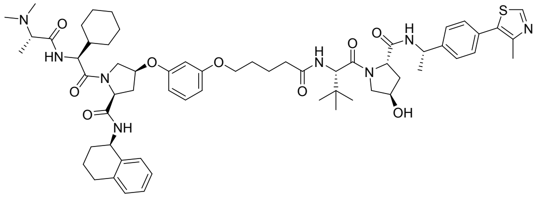
**10c**	1145	n.d.	n.d.	229	31	5	11	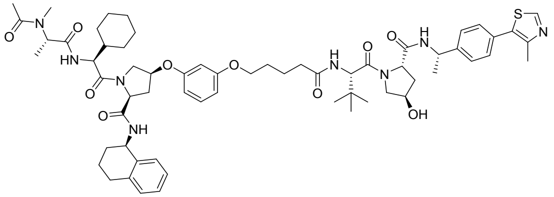
**10d (115)**	1204	n.d.	n.d.	238	31	5	11	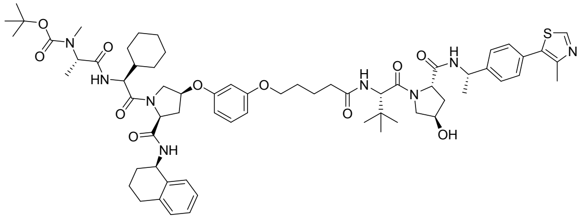
**11**	1089	3.3	n.d.	249	29	6	11	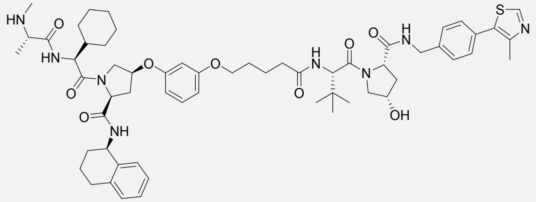

Table S4. Overview on synthesized hetero-PROTACs: IAP-VHL series 1. [a] Experimental partition coefficient at *p*H 7.4 determined by a fast-gradient HPLC method. [b] Plasma protein binding (PPB) values were estimated by an HPLC-based method. [c] Topological polar surface area is given in Å². [d] Number of rotatable bonds. Values were obtained using LigandScout 4.4.3. [e] Number of hydrogen bond donors. [f] Number of hydrogen bond acceptors. [g] Not determined. Very lipophilic compounds (logD > 5.5) may cause damage to the HSA column.

Cmpd	MW (g/mol)	logD[a]	PPB[b] (%)	TPSA[c] (Å^2)	NRotB[d]	HBD[e]	HBA[f]	Structure
**12**	1193	4.0	96	249	30	5	12	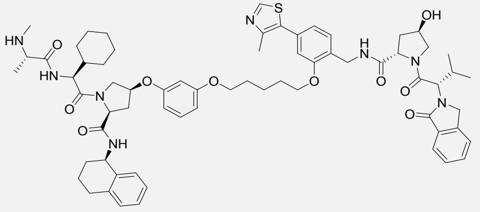
**13**	1235	4.8	97	249	33	8	12	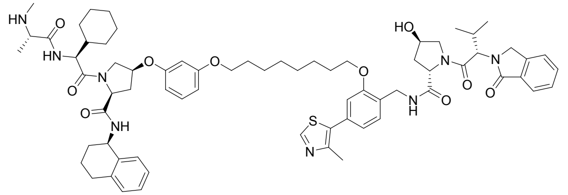
**14**	1252	4.2	96	259	34	5	13	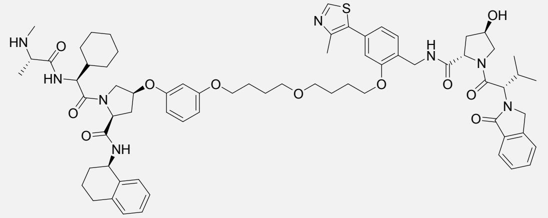
**15**	1240	3.4	95	268	33	5	14	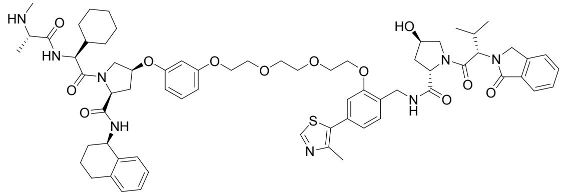
**16**	1284	3.3	95	277	36	5	15	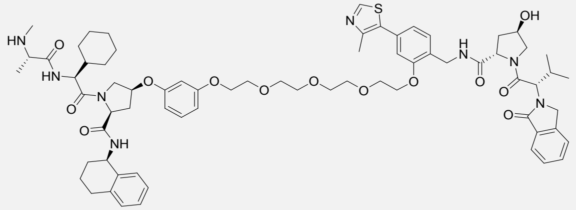

**17**	1352	5.2	97	268	41	5	14	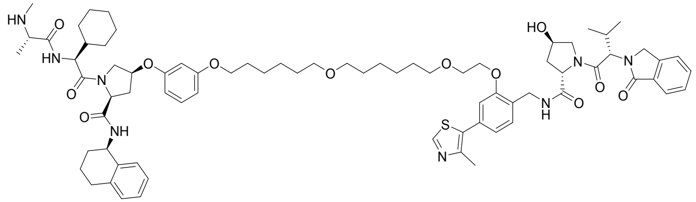
**18**	1380	5.6	n.d.	268	43	5	14	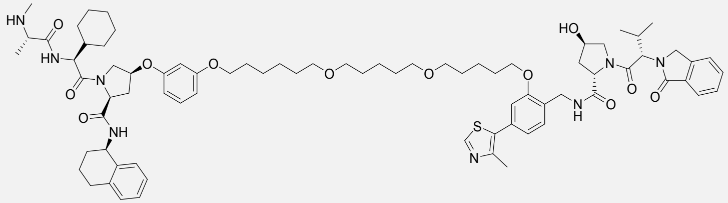
**19**	1408	6.3	n.d.	268	45	5	14	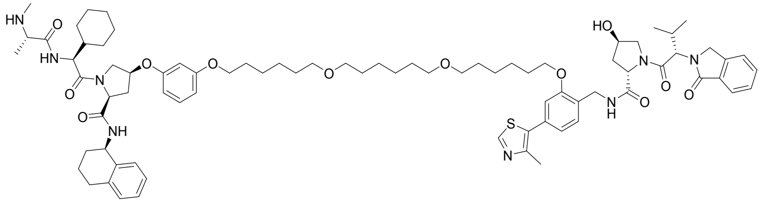

Table S5. Overview on synthesized hetero-PROTACs: IAP-VHL series 2. [a] Experimental partition coefficient at *p*H 7.4 determined by a fast-gradient HPLC method. [b] Plasma protein binding (PPB) values were estimated by an HPLC-based method. [c] Topological polar surface area is given in Å². [d] Number of rotatable bonds. Values were obtained using LigandScout 4.4.3. [e] Number of hydrogen bond donors. [f] Number of hydrogen bond acceptors. [g] Not determined. Very lipophilic compounds (logD > 5.5) may cause damage to the HSA column.

Cmpd	MW (g/mol)	logD[a]	PPB[b] (%)	TPSA[c] (Å^2)	NRotB[d]	HBD[e]	HBA[f]	Structure
**20**	918	4.6	96	204	20	5	10	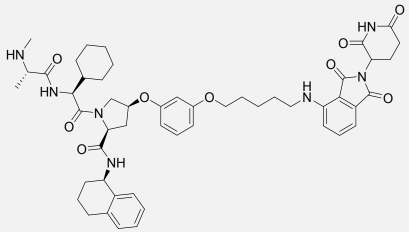
**21**	960	5.7	n.d.	204	23	5	10	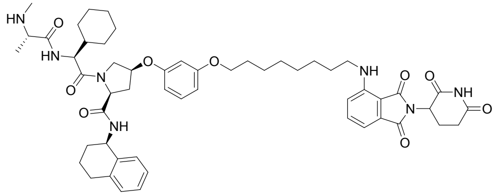
**22**	976	4.9	96	213	24	5	11	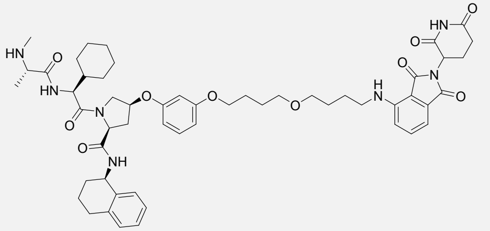
**23**	964	3.9	95	223	23	5	12	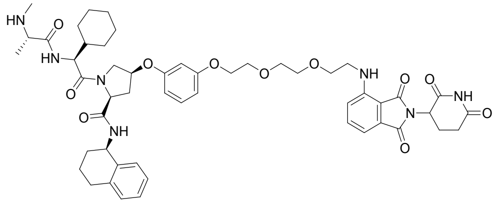

**24**	1008	3.9	94	232	26	5	13	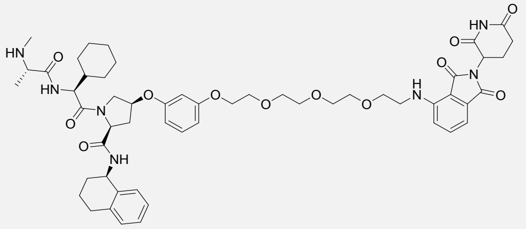
**25 (SAB141)**	1076	6.0	n.d.	223	31	5	12	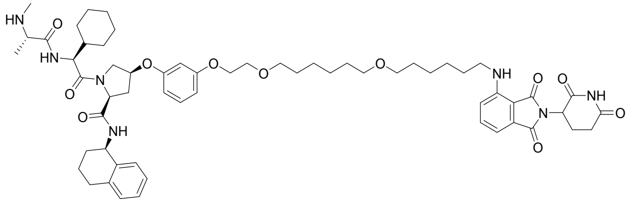
**26**	1104	6.5	n.d.	223	33	5	12	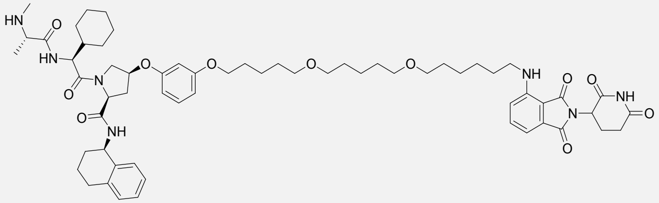
**27 (SAB142)**	1132	7.2	n.d.	223	35	5	12	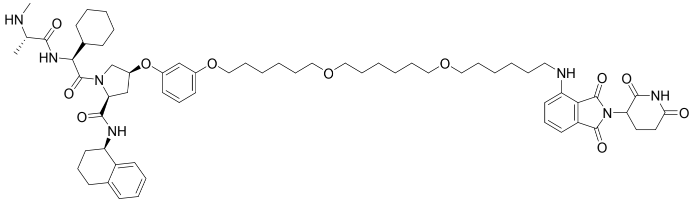
**28**	1090	6.0	n.d.	214	32	4	12	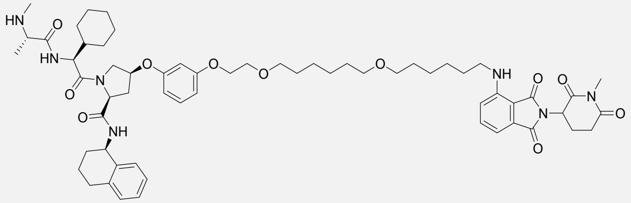

**29**	1146	7.4	n.d.	214	36	4	12	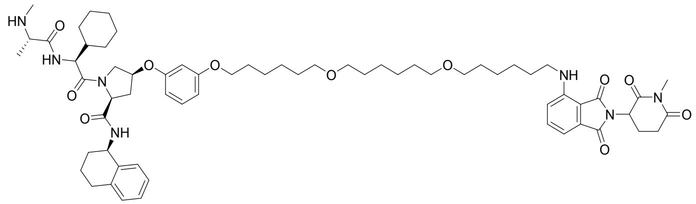

Table S6. Overview on synthesized hetero-PROTACs: IAP-CRBN series. [a] Experimental partition coefficient at *p*H 7.4 determined by a fast-gradient HPLC method. [b] Plasma protein binding (PPB) values were estimated by an HPLC-based method. [c] Topological polar surface area is given in Å². [d] Number of rotatable bonds. Values were obtained using LigandScout 4.4.3. [e] Number of hydrogen bond donors. [f] Number of hydrogen bond acceptors. [g] Not determined. Very lipophilic compounds (logD > 5.5) may cause damage to the HSA column.

Cmpd	MW (g/mol)	logD[a]	PPB[b] (%)	TPSA[c] (Å²)	NRotB[d]	HBD[e]	HBA[f]	Structure
Birinapant	807	3.4	95	195	23	8	10	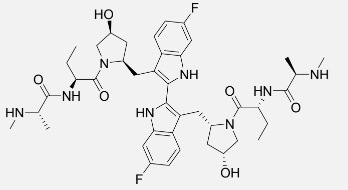
BV6	1206	3.4	n.d.[g]	239	33	8	10	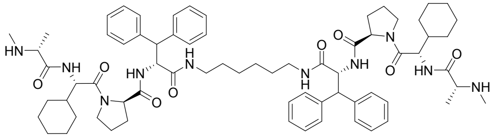
AZD5582	1015	4.1	94	200	22	6	10	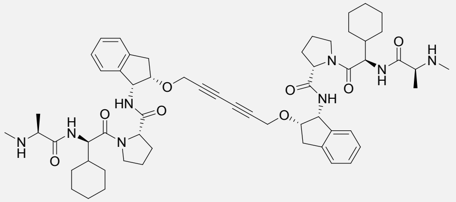
LCL161	501	3.3	88	138	10	2	6	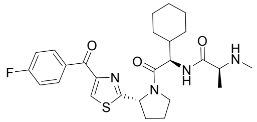
CST530	561	3.1	94	163	11	3	5	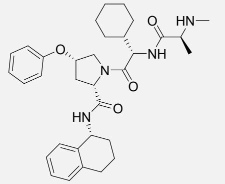

Table S7. Overview on mono- and bivalent SMAC mimetics. [a] Experimental partition coefficient at *p*H 7.4 determined by a fast-gradient HPLC method. [b] Plasma protein binding (PPB) values were estimated by an HPLC-based method. [c] Topological polar surface area is given in Å². [d] Number of rotatable bonds. Values were obtained using LigandScout 4.4.3. [e] Number of hydrogen bond donors. [f] Number of hydrogen bond acceptors. [g] Not determined.

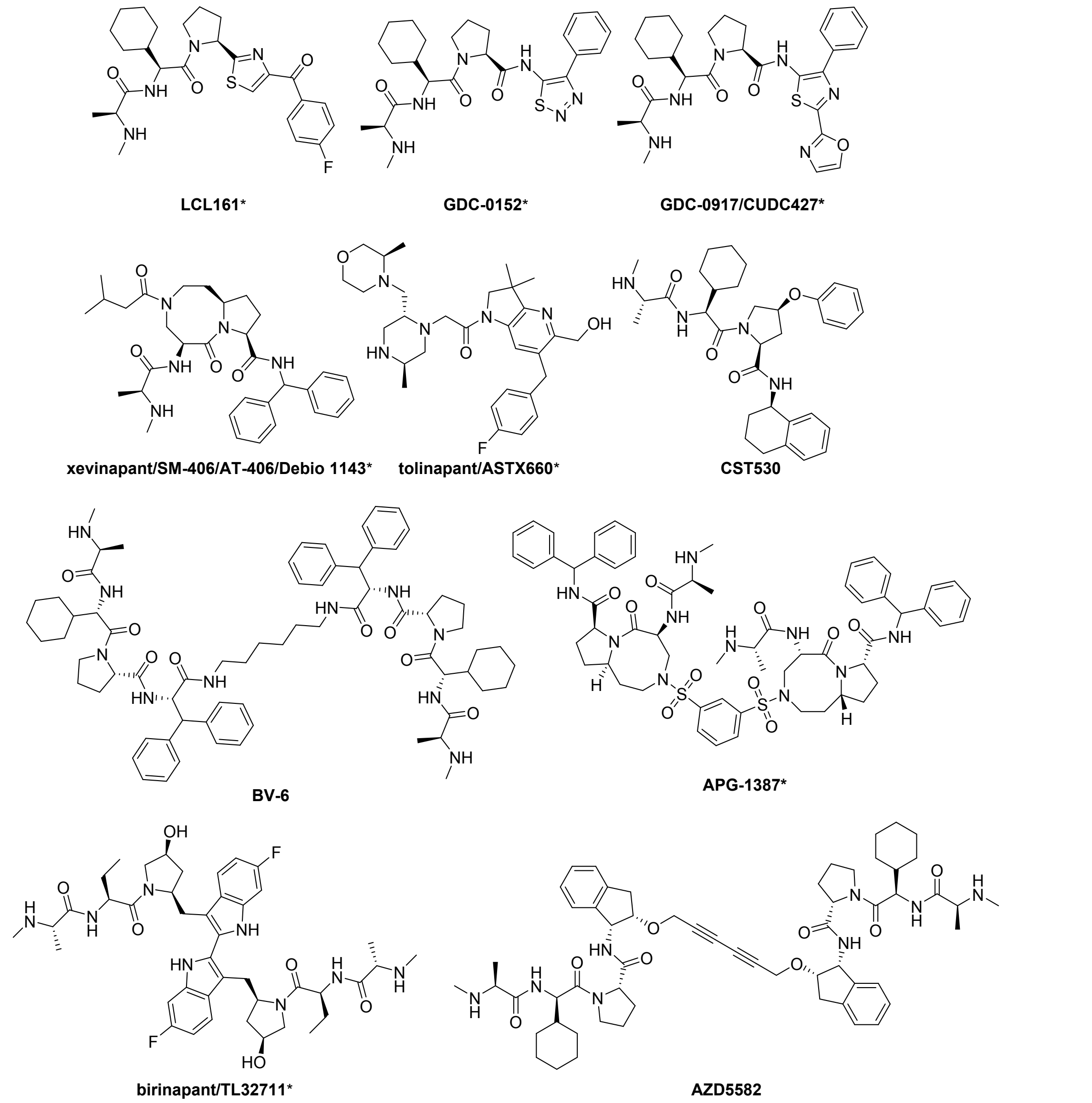

Figure S1. The most promising small molecules targeting IAPs are mimicking the IAP-binding motif of SMAC. Compounds that entered clinical trials are marked with an asterisk (please note that AEG40826 and BI 891065 are two additional clinically evaluated SMAC mimetics, but their structures are undisclosed). SMAC mimetics bind to XIAP, cIAP1, and cIAP2 and antagonize their functions, *e.g.* association with caspases and with regulators of the nuclear factor-κB signaling pathways.[1–3] They also prevent XIAP-mediated inhibition of capases-3/7/9.[4] Bivalent IAP antagonists are composed of two monovalent units that are connected through a linker. Since these can bind to two IAP proteins they are better at triggering dimerization and autodegradation of cIAPs.[5–7] Moreover, they generally display higher binding affinities than monovalent compounds for IAPs.[4,8,9]

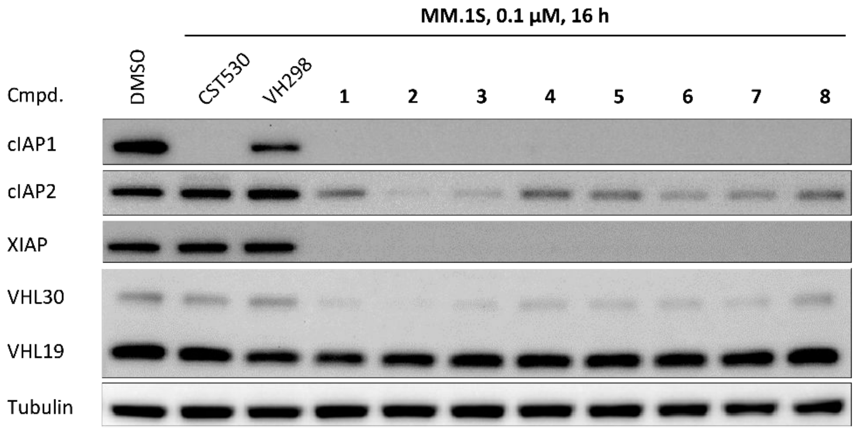

Figure S2. IAP-VHL Series 1 hetero-PROTACs induce strong cIAP1, cIAP2 (in certain cases), XIAP, as well as VHL30 degradation. MM.1S cells were treated for 16 h with 0.1 μM IAP ligand CST530, VHL ligand VH298, and hetero-PROTACs **1**-**8**.

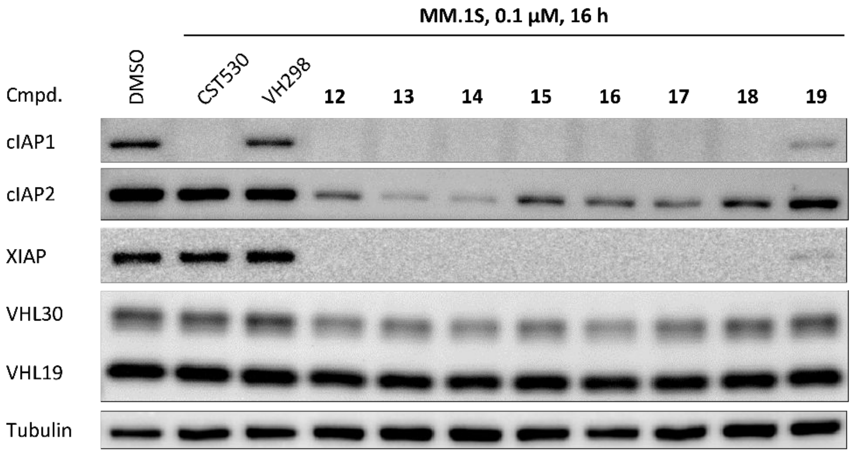

Figure S3. IAP-VHL Series 2 hetero-PROTACs induce strong cIAP1, cIAP2 (in certain cases), and XIAP degradation. MM.1S cells were treated for 16 h with 0.1 μM IAP ligand CST530, VHL ligand VH298, and hetero-PROTACs **12**-**19**.

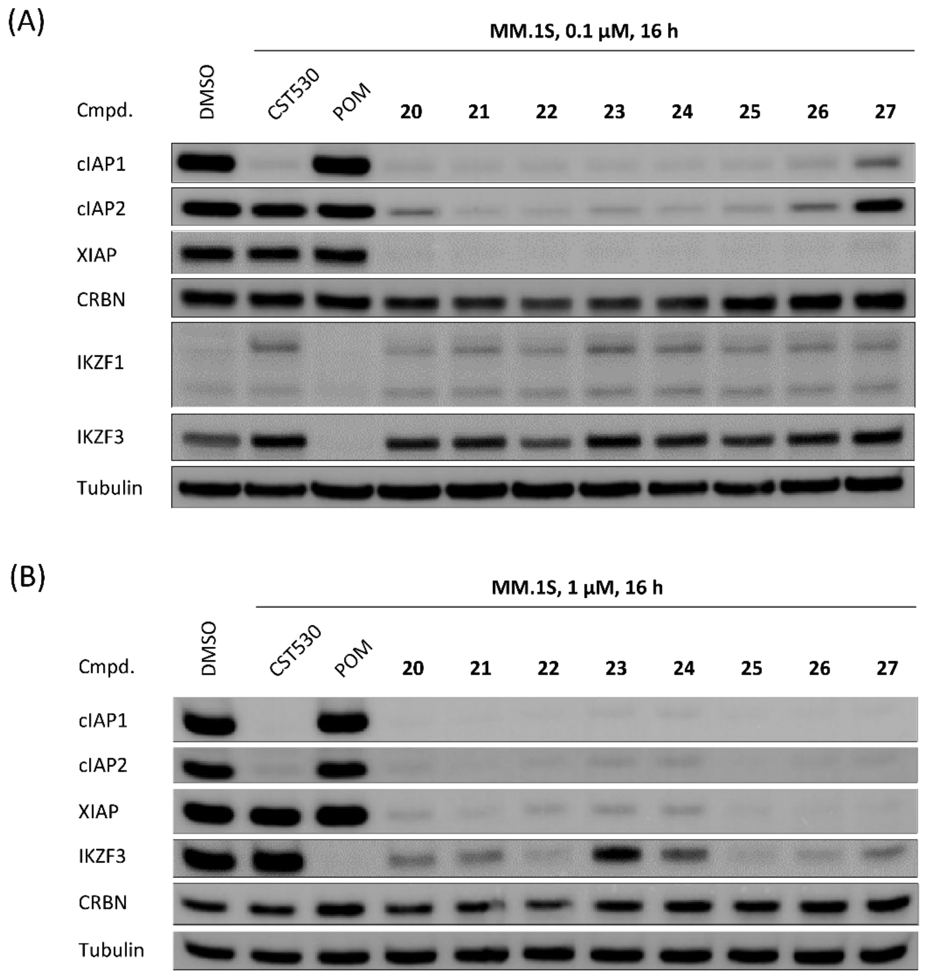

Figure S4. IAP-CRBN hetero-PROTACs induce strong degradation of IAPs. MM.1S cells were treated for 16 h with 0.1 µM (A) or 1 µM (B) IAP ligand CST530, VHL ligand VH298, and hetero-PROTACs **20**-**27**.

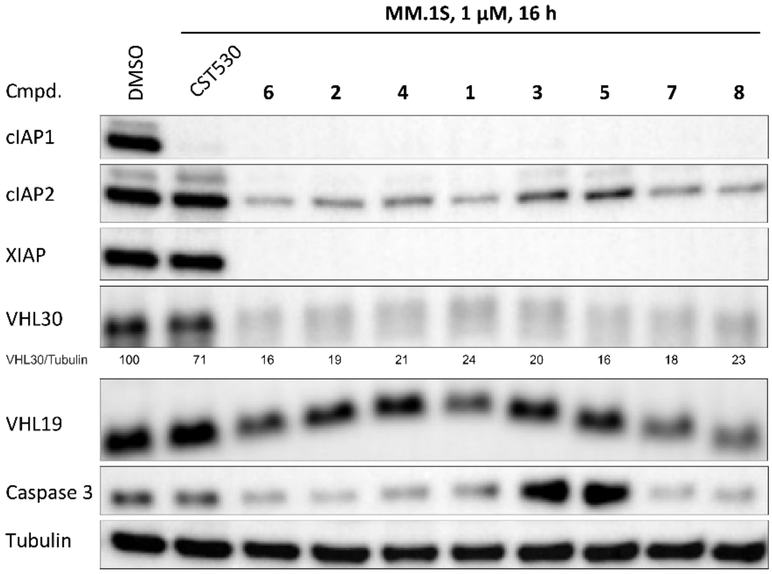

Figure S5. IAP-VHL Series 1 hetero-PROTACs **1**-**8** strongly degrade cIAP1 and XIAP, as well as VHL30 at 1 µM. MM.1S cells were treated with compounds for 16 h.

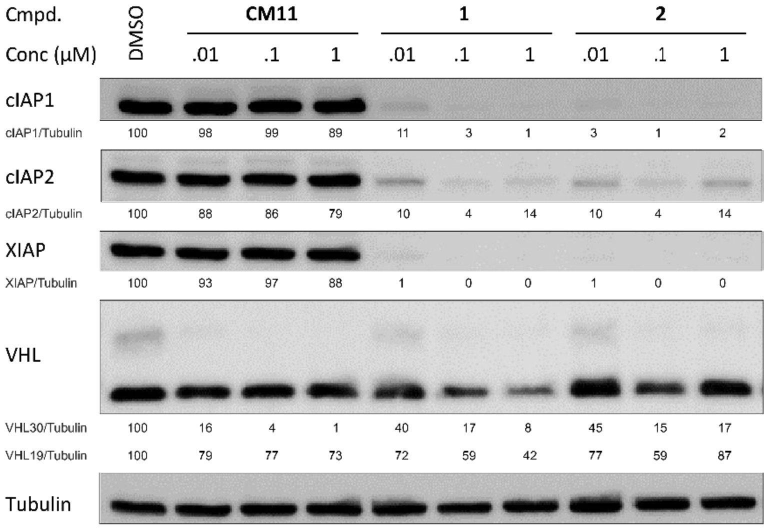

Figure S6. CM11 and hetero-PROTACs **1** and **2** induce strong and dose-dependent VHL30 degradation. A slight hook effect for VHL19 degradation is seen for hetero-PROTAC **2**. MM.1S cells were treated with PROTACs at indicated concentrations for 16 h.

(A)

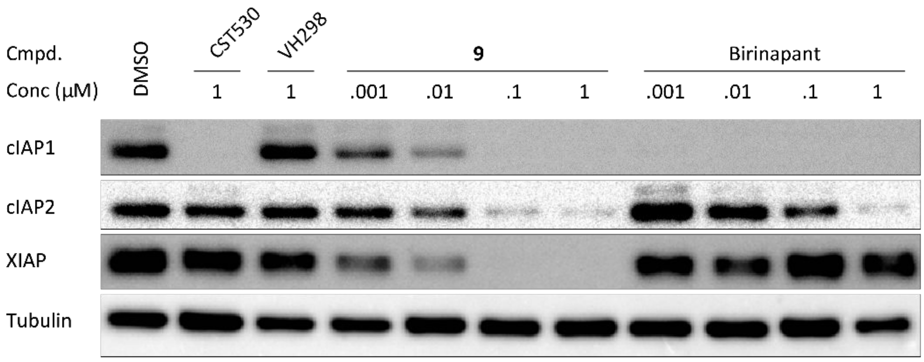

(B)

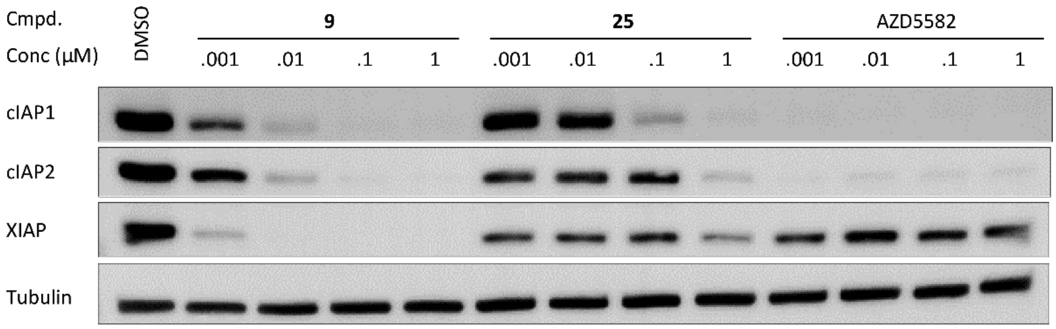

Figure S7. (A) Hetero-PROTAC **9** depletes cIAP2 more potently than birinapant, and induces XIAP degradation. MM.1S cells were treated with **9** and birinapant at indicated concentrations for 16 h. (B) AZD5582 potently degrades both cIAPs, but does not have an influence on XIAP levels. MM.1S cells were treated with hetero-PROTACs and AZD5582 at indicated concentrations for 16 h.

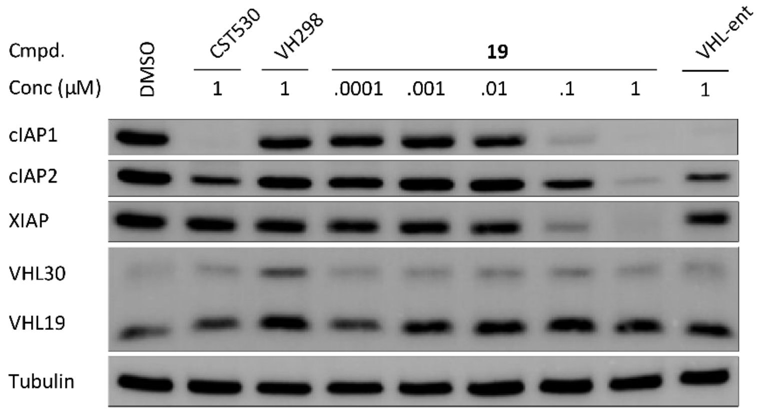

Figure S8. IAP-VHL hetero-PROTAC **19** induces IAPs degradation in a dose-dependent manner. MM.1S cells were treated with **19** at indicated concentrations for 16 h.

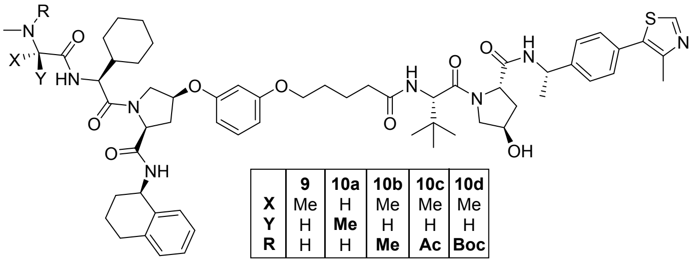

	9	10a	10b	10c	10d
**X**	Me	H	Me	Me	Me
**Y**	H	**Me**	H	H	H
**R**	H	H	**Me**	**Ac**	**Boc**

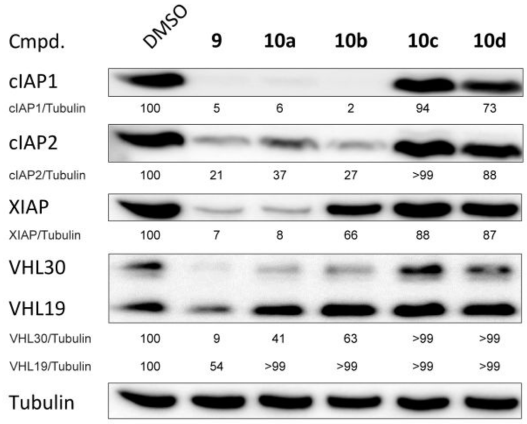

Figure S9. Testing of the IAP-VHL hetero-PROTAC **9** (CST626) and a series of putative IAP non-binding control compounds (**10a**–**10d**). MM.1S cells were treated with compounds at 1 µM for 16 h. Quantification in this representative blot refers to mean of replicates (n=3).

(A)

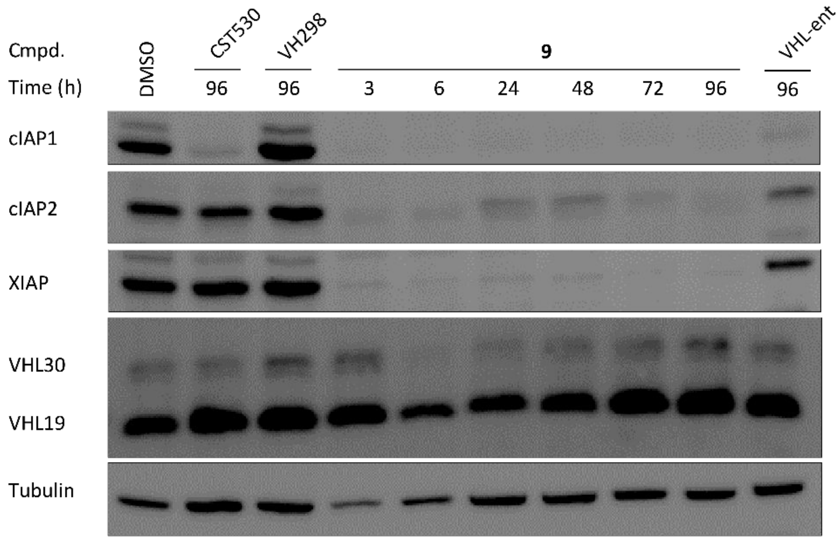

(B)

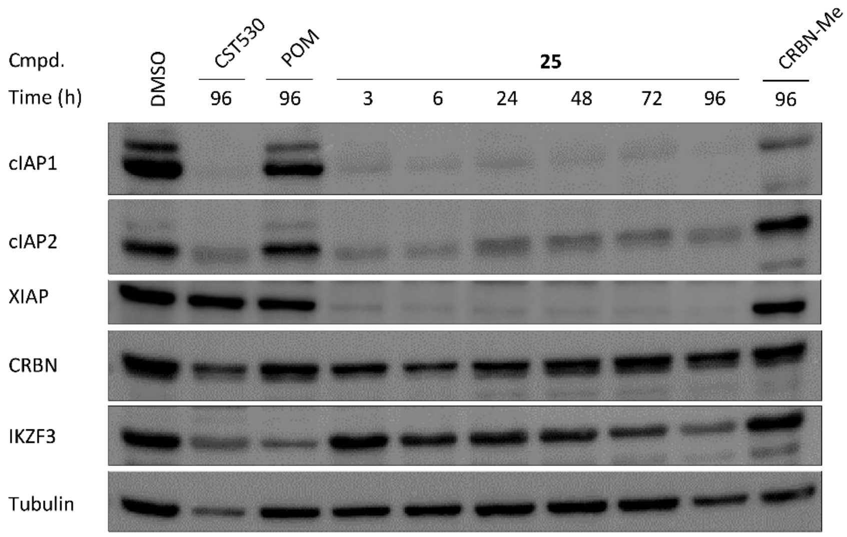

Figure S10. (A) Time course experiment with hetero-PROTAC **9**. MM.1S cells were treated with compounds at 0.1 µM for the indicated time. (B) Time course experiment with hetero-PROTAC **25**. MM.1S cells were treated with compounds at 0.1 µM for the indicated time.

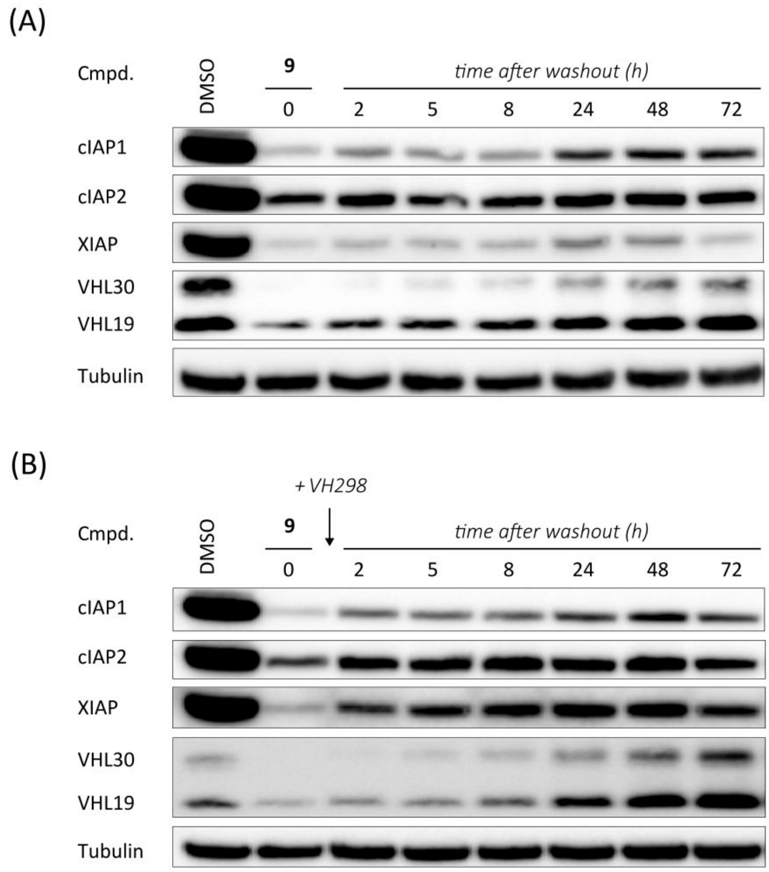

Figure S11. Persisting effects of hetero-PROTAC **9** on IAPs degradation after compound washout from the cell medium. MM.1S cells were treated with **9** at 1 µM for 16 h before washout (= 0 h), and then kept in plain media (A) in the absence or (B) in the presence of 1 µM VH298 until indicated time points.

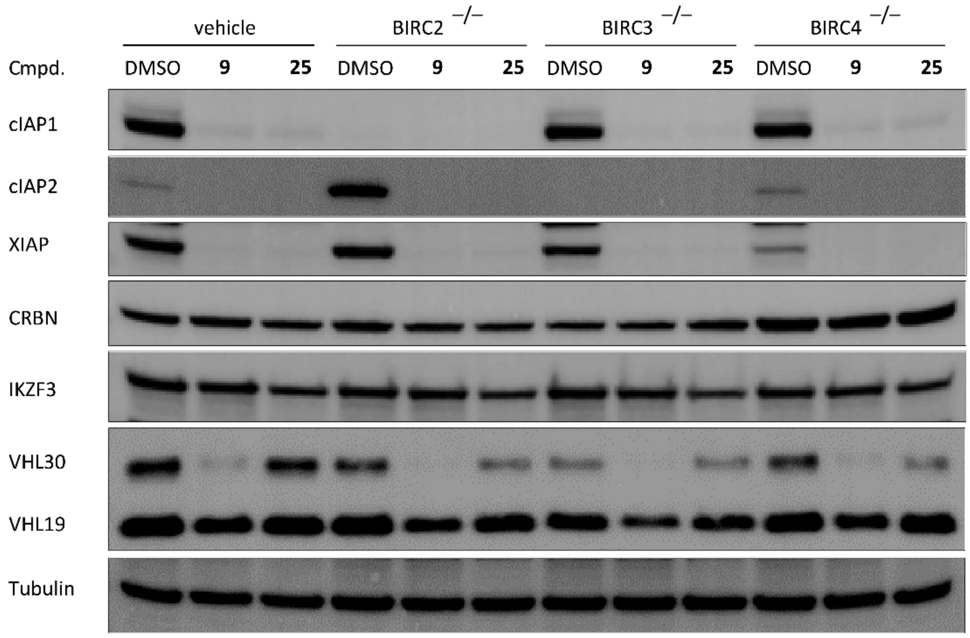

Figure S12. Knockout of individual IAPs in MM.1S cells do not influence VHL30 degradation by hetero-PROTAC **9**. Individual IAP knock-out cells were treated with hetero-PROTACs at 0.1 µM for 16 h.

(A)

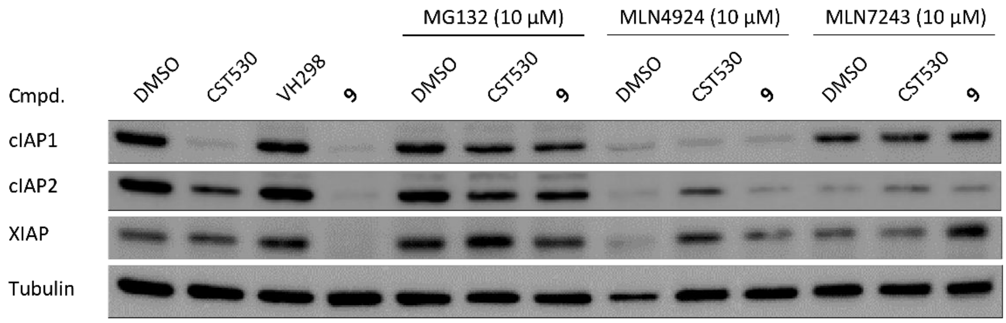

(B)

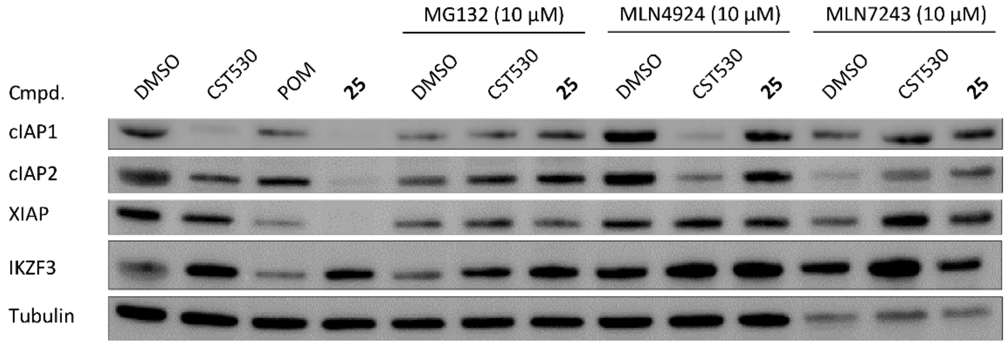

Figure S13. (A) Degradation of IAPs induced by hetero-PROTAC **9** is mediated *via* the ubiquitin-proteasome system. MG132, MLN4924, and MLN7243 prevent proteasomal degradation of IAPs. MM.1S cells were treated with 10 µM MG132, MLN4924, or MLN7243 for 1 h before the addition of **9** for additional 3 h. (B) Degradation of IAPs induced by hetero-PROTAC **25** is mediated *via* the ubiquitin-proteasome system. MG132, MLN4924, and MLN7243 prevent proteasomal degradation of IAPs. MM.1S cells were treated with 10 µM MG132, MLN4924, or MLN7243 for 1 h before the addition of **25** for further 3 h.

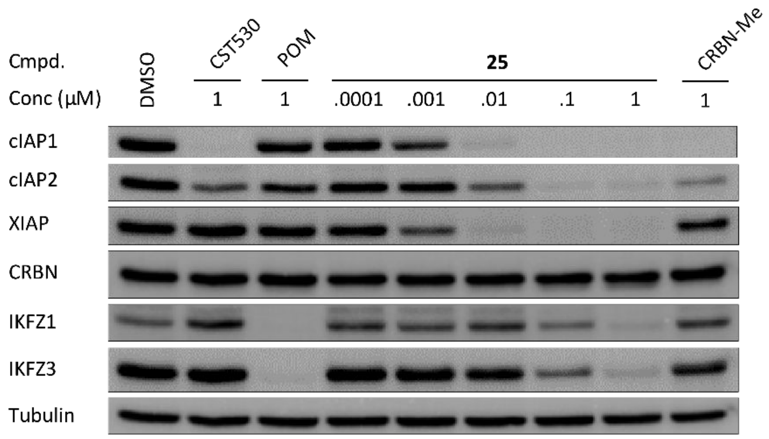

Figure S14. IAP-CRBN hetero-PROTAC **25** (SAB141) induces cIAP1, cIAP2, XIAP, IKZF1, and IKZF3 in a dose-dependent manner. CRBN-non-binding control **28** (CRBN-Me) only marginally degrades XIAP, IKZF1, and IKZF3. MM.1S cells were treated with compounds at indicated concentrations for 16 h.

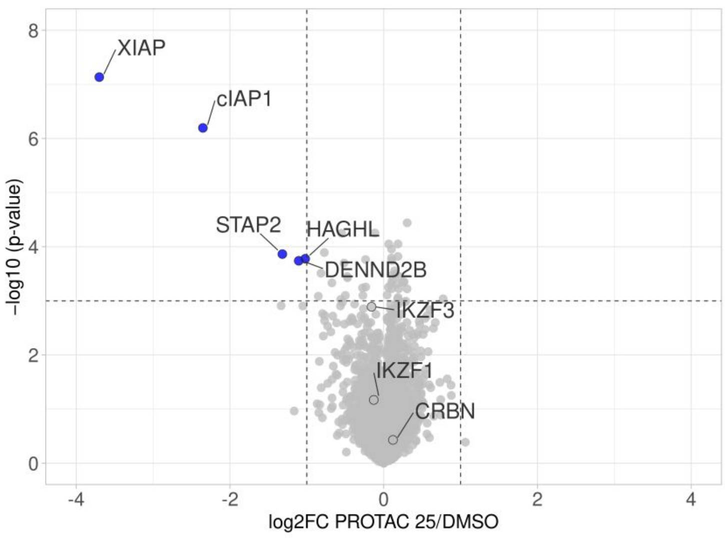

Figure S15. diaPASEF quantitative proteomics for PROTAC **25**. MM.1S cells were treated with compound **25** at 0.1 µM for 3 h. The identified proteins were plotted as log2 fold change (PROTAC/DMSO) versus –log 10 of p-value. Proteins with –log10 (p-value) >3 (p-value <0.001) and log2 fold change >1 or <–1 (translating to 2-fold up- or down-regulation) were considered to have significantly changed in abundance. Data are mean of biological duplicates.

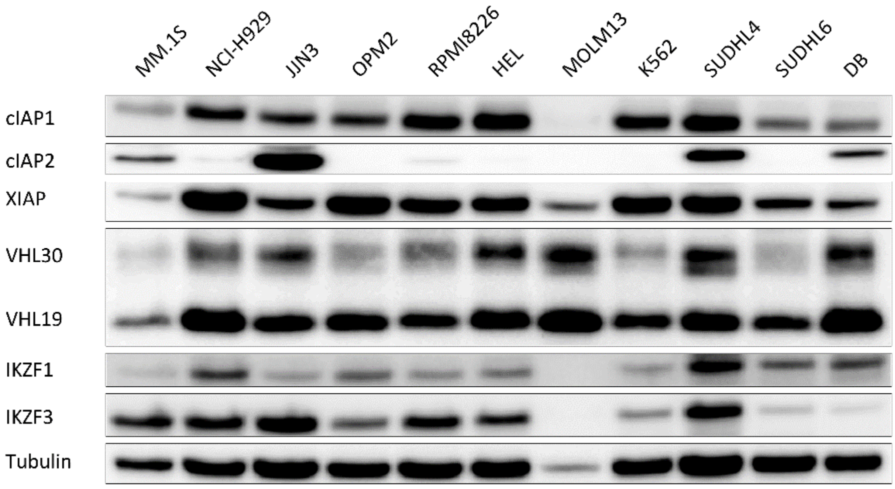

Figure S16. Baseline expression of IAPs in a panel of hematological cell lines.

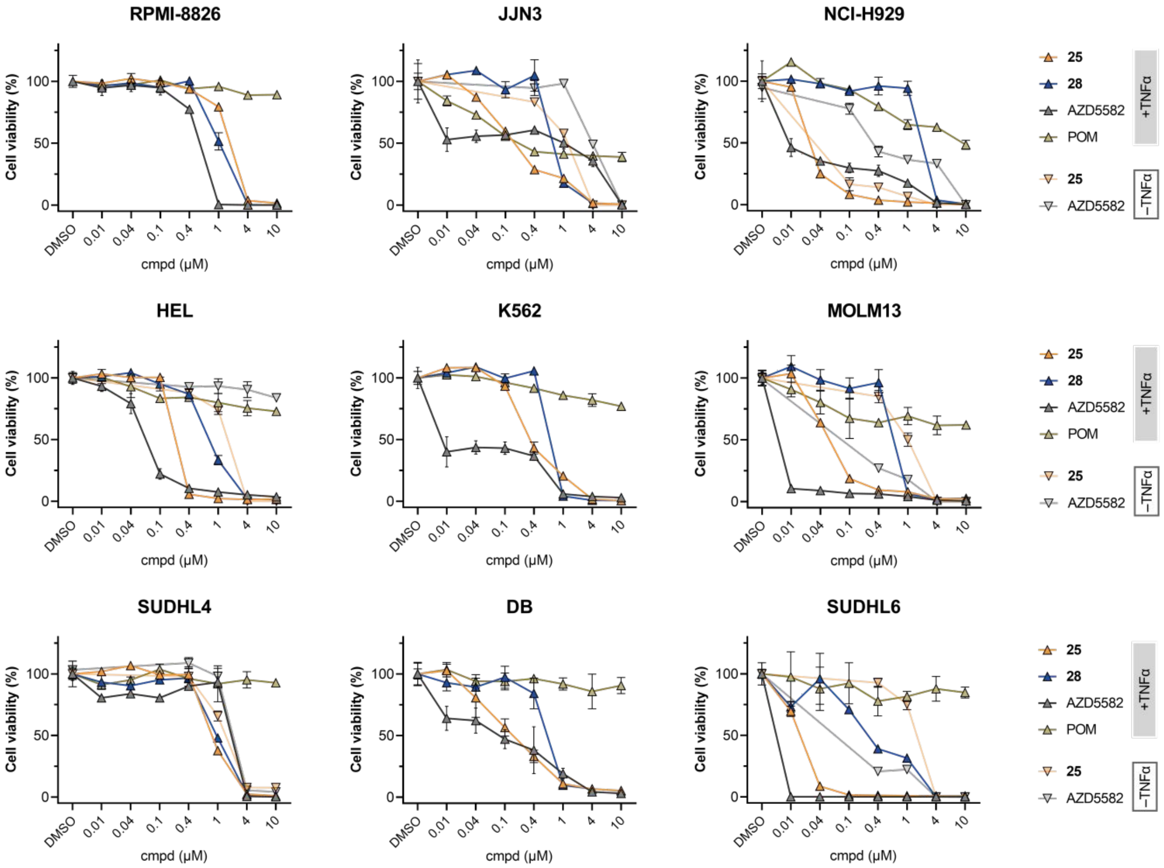

Figure S17. Cell viability screenings in 9 different hematological cancer cell lines with pan-IAP degrader **25**, its CRBN non-binding control **28**, as well as the advanced bivalent IAP antagonist AZD5582 and the CRBN binder pomalidomide (POM) as respective controls. In certain cases, viability inhibition was assessed in the presence and absence of TNF-α. Multiple myeloma, acute myeloid leukemia, and lymphoma cell lines were treated with the respective compounds at indicated concentrations for 96 h. Viability is normalized to their respective DMSO controls. Data represent means ± s.d. of at least three independent biological replicates.

cell line	disease [a]	**25**	**28**	**AZD5582**	POM
		IC$_{50}$ (µM)			
**RPMI-8826**	MM	1.52	1.01	0.47	>10
**JJN3**	MM	0.18	0.90	0.20	0.042
**NCI-H929**	MM	0.027	1.99	0.014	>10
**HEL**	AML	0.30	0.77	0.064	>10
**K562**	AML	0.38	0.84	0.019	>10
**MOLM13**	AML	0.052	0.63	0.0020	>10
**SUDHL4**	DLBCL	0.92	1.00	1.64	>10
**DB**	DLBCL	0.16	0.57	0.073	>10
**SUDHL6**	DLBCL	0.014	0.44	0.0015	>10

[a] MM, multiple myeloma; AML, acute myeloid leukemia; DLBCL, diffuse large B-cell lymphoma.

Table S8. Cell viability profiles (IC$_{50}$ values) of the CRBN-recruiting PROTAC **25**, the CRBN non-binding control **28**, as well as the bivalent IAP antagonist AZD5582 and the CRBN ligand pomalidmide (POM) for comparison. Values correspond to TNFα-challenged conditions.

Scheme S1. Synthesis of linker **L7a** (**40**).

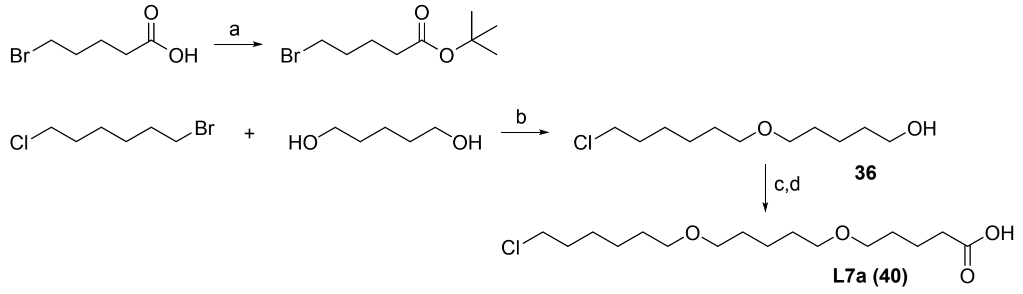

Reagents and conditions: (a) *t*BuOH, DCC, DMAP, CH_2Cl_2, rt, 18 h; (b) 50% NaOH (aq), DMSO, rt, 24 h; (c) *tert*-butyl 5-bromopentanoate, toluene, 50% NaOH (aq), TBAHS, rt, 18 h; (d) TFA, CH_2Cl_2, 40 °C, 2 h.

Scheme S2. Synthesis of linkers **L6a** (**38**) and **L8a** (**43**).

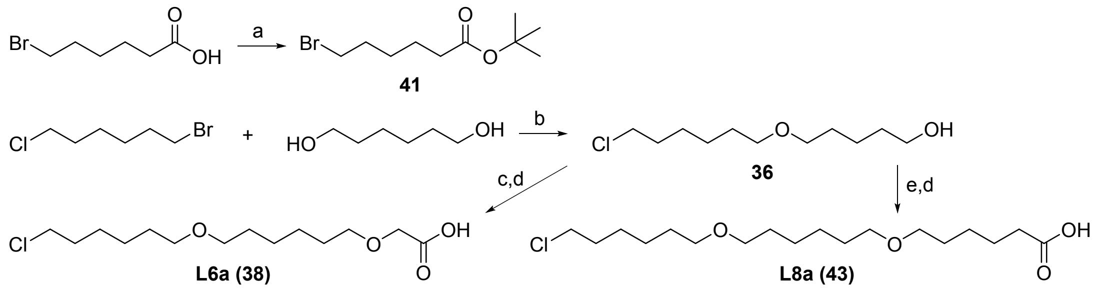

Reagents and conditions: (a) *t*BuOH, DCC, DMAP, CH_2Cl_2, rt, 18 h; (b) 50% NaOH (aq), DMSO, rt, 24 h; (c) *tert*-butyl bromoacetate, NaH, DMF/THF, 0 °C to rt, 18 h; (d) TFA, CH_2Cl_2, 40 °C, 2 h; (e) **41**, toluene, 50% NaOH (aq), TBAHS, rt, 18 h.

Scheme S3. Synthesis of the IAP-VHL Series 1 hetero-PROTACs **2**-**8**.

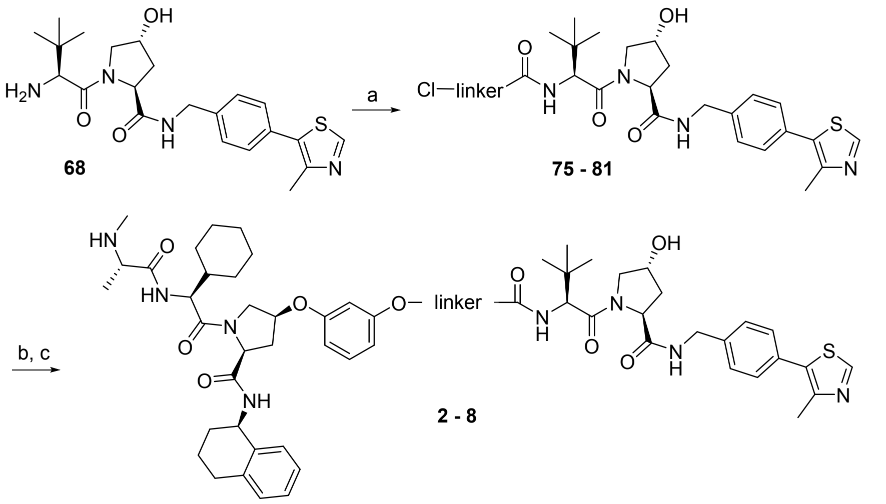

Reagents and conditions: (a) linker **L2a**-**L8a** (Table S2), HATU, DIPEA, DMF, rt, 16 h; (b) (i) NaI, acetone, 60 °C, 48 h; (ii) **65**, Cs_2CO_3, DMF, 60 °C, 16 h; (c) 1 M HCl in EtOAc, rt, 4 h.

Scheme S4. Synthesis of the IAP-VHL Series 1 hetero-PROTACs **1**, **9**, and negative controls **10a-10d** and **11**.

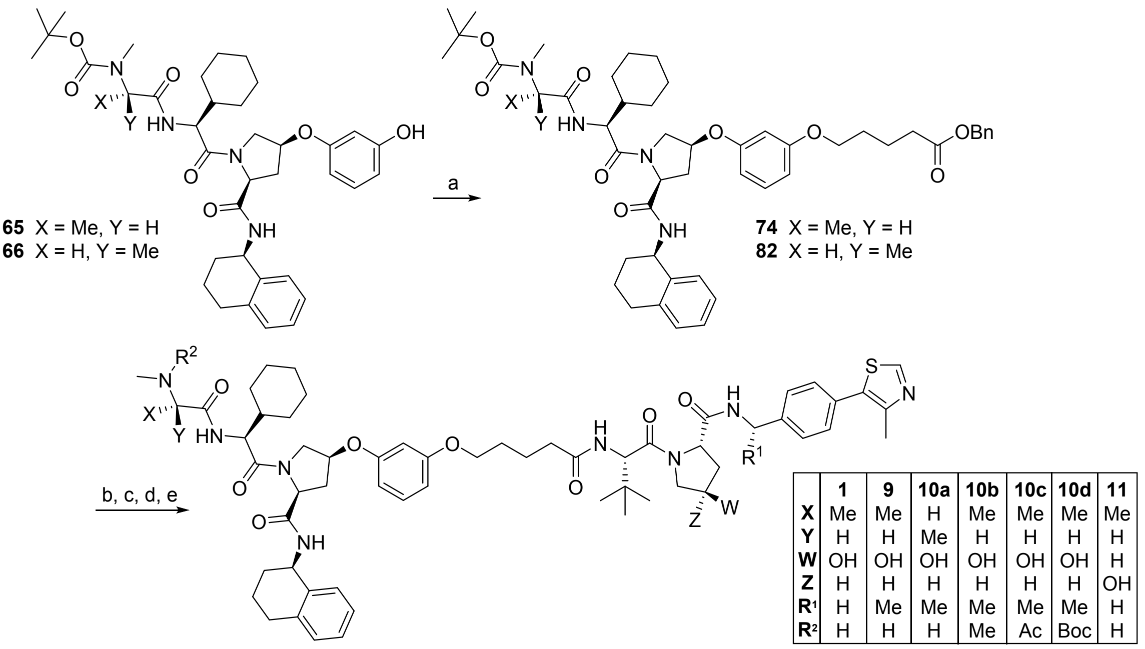

	**1**	**9**	**10a**	**10b**	**10c**	**10d**	**11**
**X**	Me	Me	H	Me	Me	Me	Me
**Y**	H	H	Me	H	H	H	H
**W**	OH	OH	OH	OH	OH	OH	H
**Z**	H	H	H	H	H	H	OH
**R¹**	H	Me	Me	Me	Me	Me	H
**R²**	H	H	H	Me	Ac	Boc	H

Reagents and conditions: (a) (i) linker **L1a** (Table S2), NaI, acetone, 60 °C, 48 h; (ii) Cs_2CO_3, DMF, 60 °C, 16 h; (b) Pd/C, H_2, EtOAc, rt, 18 h; (c) **68** or **69**, HATU, DIPEA, DMF, rt, 16 h; (d) 1 M HCl in EtOAc, rt, 4 h; (e) **9**, formaldehyde, Pd/C, H_2, DMF, rt, 16 h (for compound **10b**) or **9**, Ac_2O, DIPEA, CH_2Cl_2, 0 °C to rt, 16 h (for compound **10c**).

Scheme S5. Synthesis of linker **L6b** (**53**).

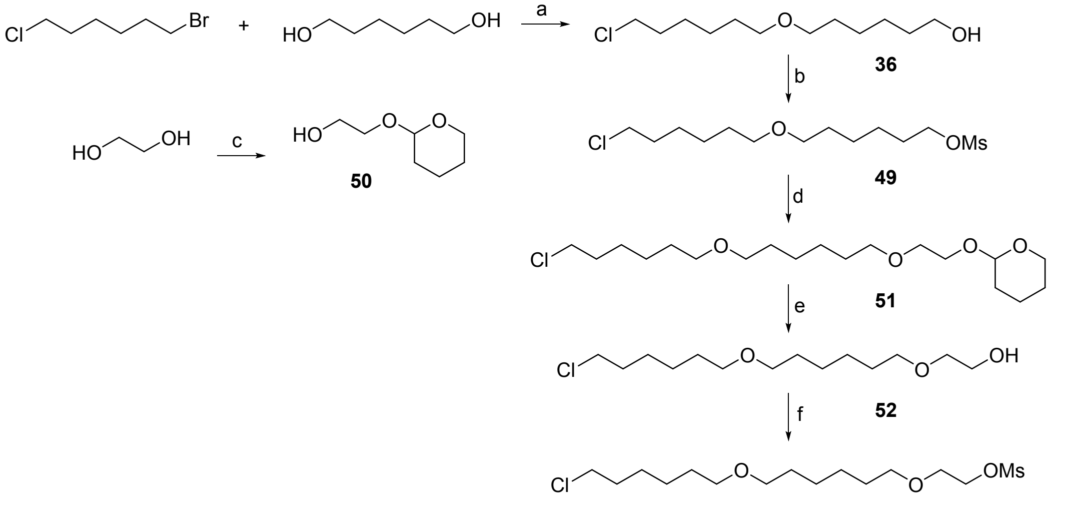

Reagents and conditions: (a) 50% NaOH (aq), DMSO, rt, 18 h; (b) MsCl, DIPEA, CH_2Cl_2, rt, 2 h; (c) 3,4-dihydro-2*H*-pyran, $CuSO_4 \times 5\ H_2O$, MeCN, rt, 4 h; (d) **50**, TBAHS, 50% NaOH (aq), toluene, rt, 18 h; (e) *p*TsOH × H_2O, MeOH, rt, 20 h; (f) MsCl, DIPEA, CH_2Cl_2, rt, 2 h.

Scheme S6. Synthesis of linker **L7b** (**54**).[10]

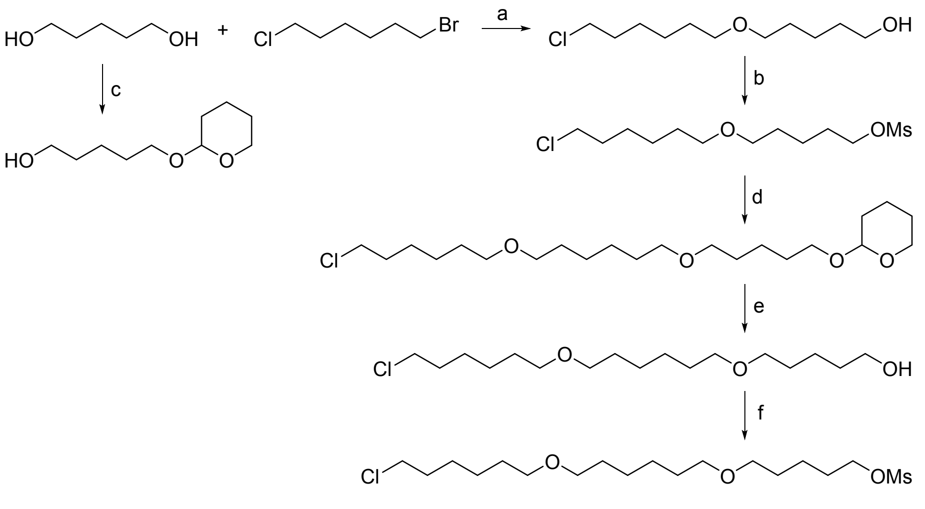

Reagents and conditions: (a) 50% NaOH (aq), DMSO, rt, 18 h; (b) MsCl, DIPEA, CH_2Cl_2, rt, 2 h; (c) 3,4-dihydro-2*H*-pyran, $CuSO_4$ × 5 H_2O, MeCN, rt, 4 h; (d) 5-((tetrahydro-2*H*-pyran-2-yl)oxy)pentan-1-ol, TBAHS, 50% NaOH (aq), toluene, rt, 20 h; (e) *p*TsOH × H_2O, MeOH, rt, 20 h; (f) MsCl, DIPEA, CH_2Cl_2, rt, 2 h.

Scheme S7. Synthesis of linker **L8b** (**59**).

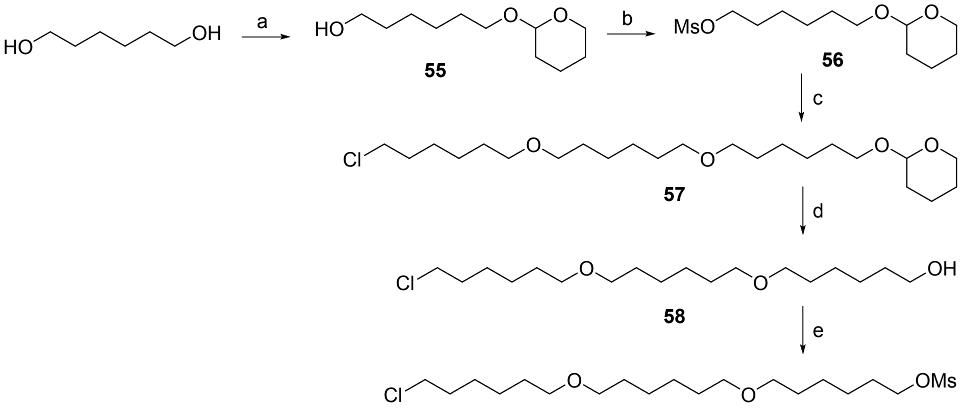

L8b (59)

Reagents and conditions: (a) 3,4-dihydro-2*H*-pyran, $CuSO_4 \times 5\ H_2O$, MeCN, rt, 4 h; (b) MsCl, DIPEA, CH_2Cl_2, rt, 2 h; (c) **36**, TBAHS, 50% NaOH (aq), toluene, rt, 18 h; (d) *p*TsOH × H_2O, MeOH, rt, 20 h; (e) MsCl, DIPEA, CH_2Cl_2, rt, 2 h.

Scheme S8. Synthesis of the IAP-VHL Series 2 hetero-PROTACs **12**-**19**.

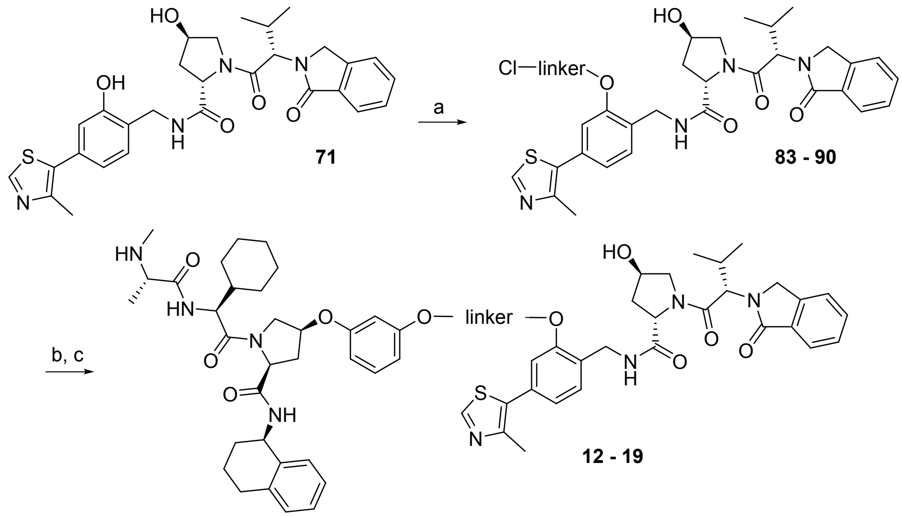

Reagents and conditions: (a) linker **L1b**-**L8b** (Table S3), Cs_2CO_3, DMF, rt, 16 h, then 60 °C, 3 h; (b) (i) NaI, acetone, 60 °C, 48 h; (ii) **65**, Cs_2CO_3, DMF, 60 °C, 16 h; (c) 1 M HCl in EtOAc, rt, 4 h.

Scheme S9. Synthesis of the IAP-CRBN Series hetero-PROTACs **20**-**29**.

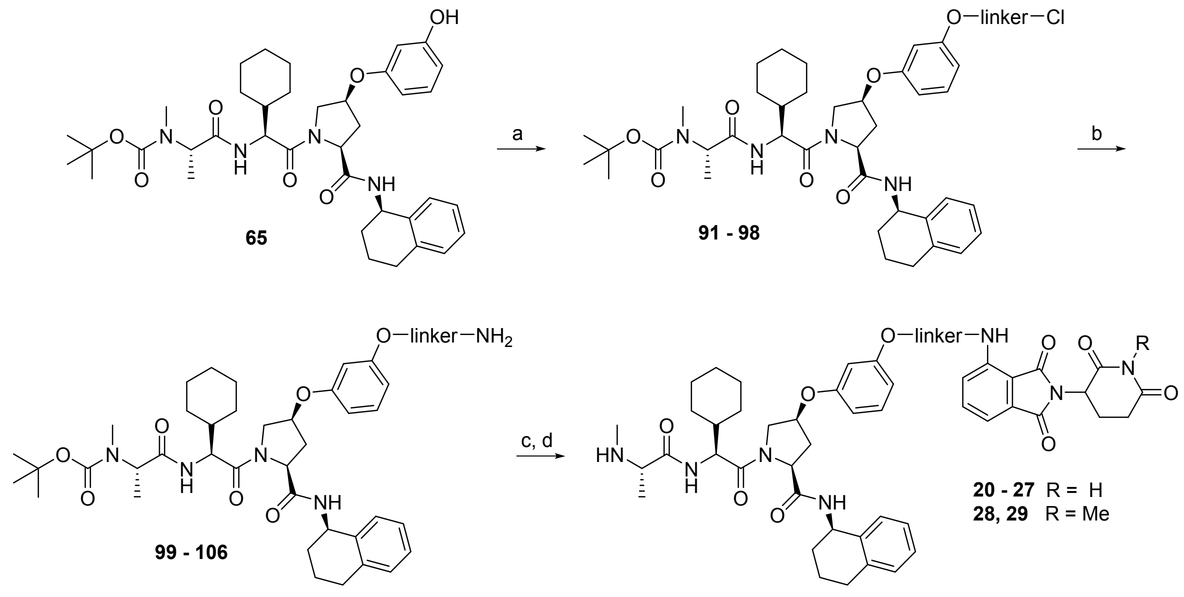

Reagents and conditions: (a) linker **L1b**-**L8b** (Table S3), K_2CO_3, DMF, 70 °C, 20 h; (b) (i) NaN_3, DMF, 80 °C, 4 h; (ii) Pd/C, H_2, MeOH, rt, 3 h; (c) **72** or **73**, DIPEA, DMSO, 90 °C, 20 h; (d) 1 M HCl in EtOAc, rt, 4 h.

Selected NMR and MS spectra

^{1}H and ^{13}C NMR spectra of PROTAC **9** (CST626)

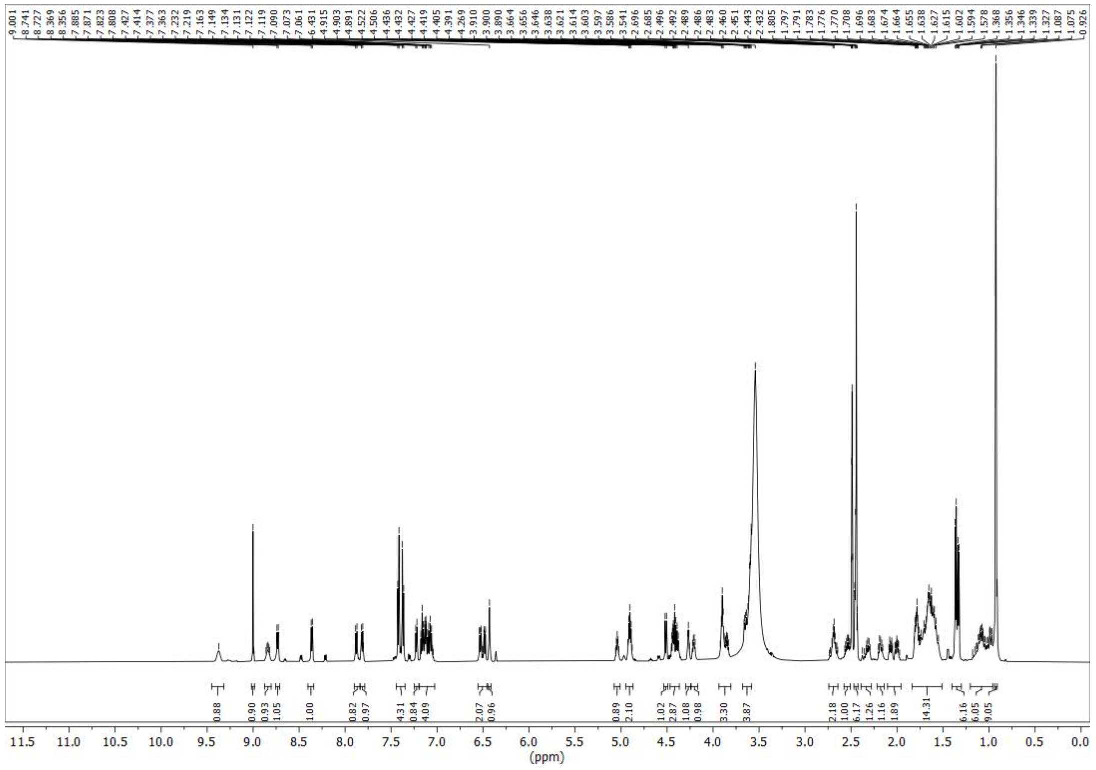

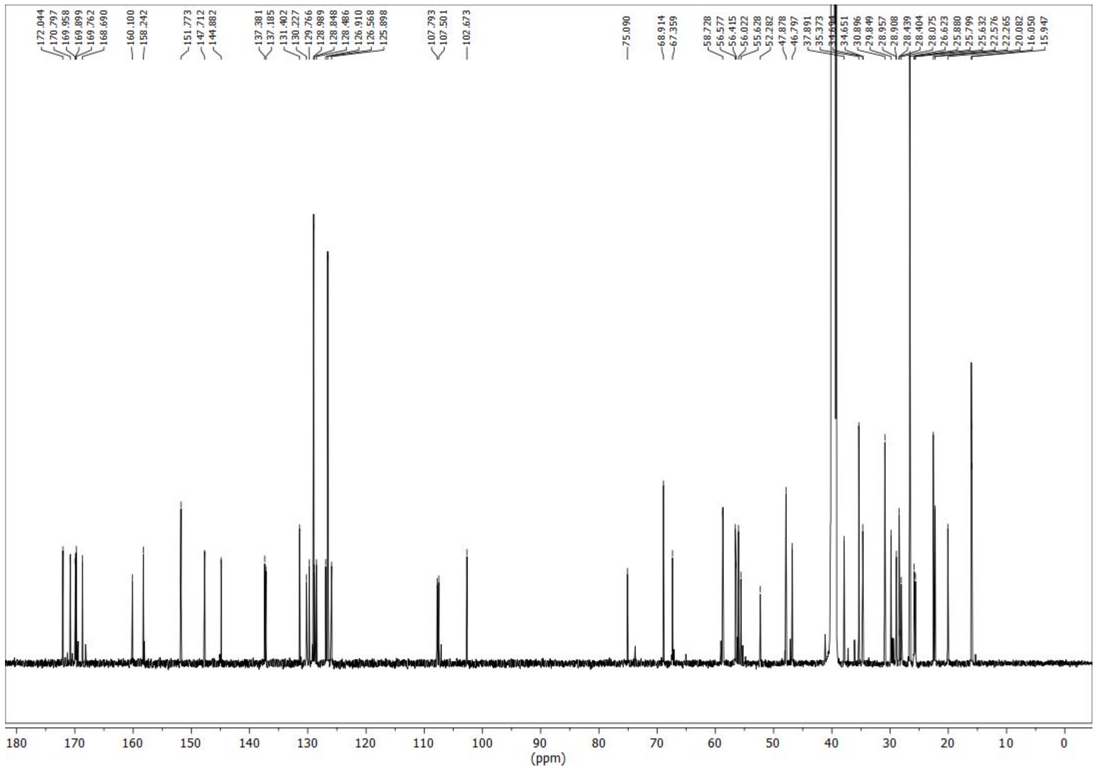

Aromatic regions of ^{1}H NMR spectra for PROTAC **9** (in a hydrochloride form) at 25 °C, 80 °C, and both spectra superimposed. A clear averaging of signals is observed at 80 °C indicating the presence of rotamers in the final PROTAC structure.

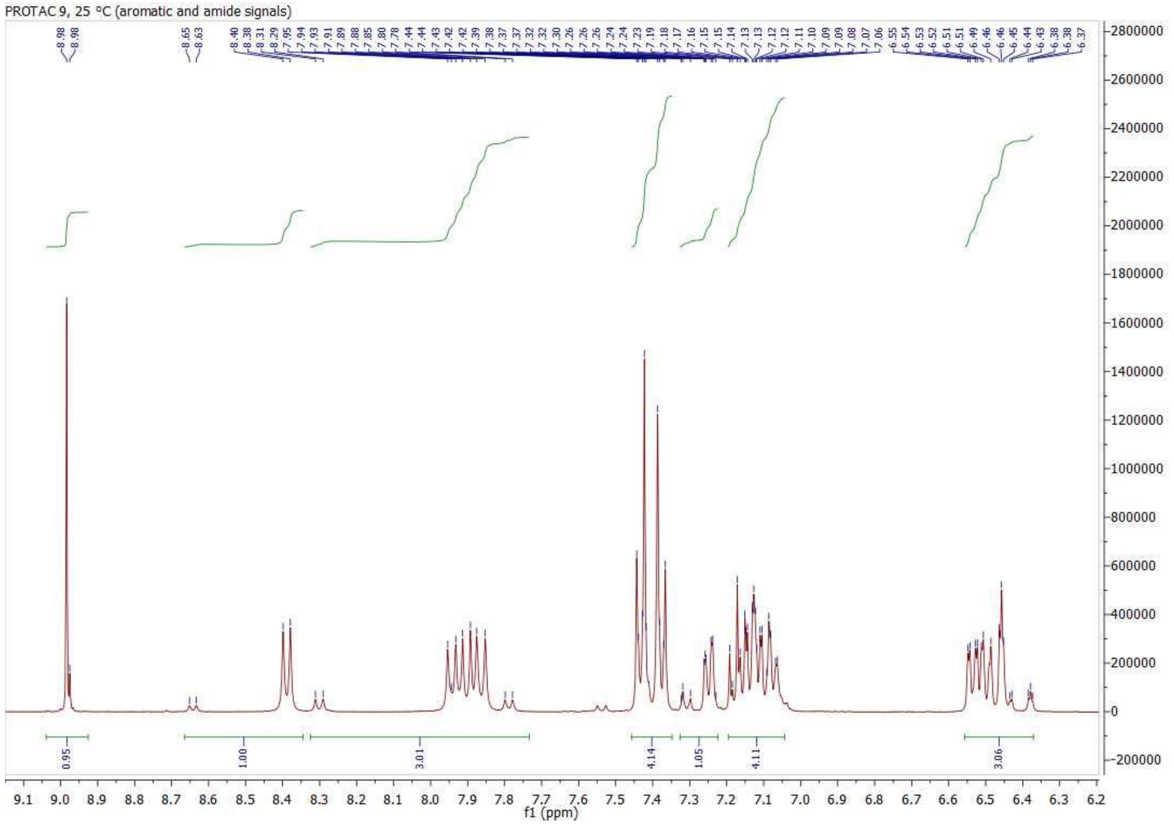

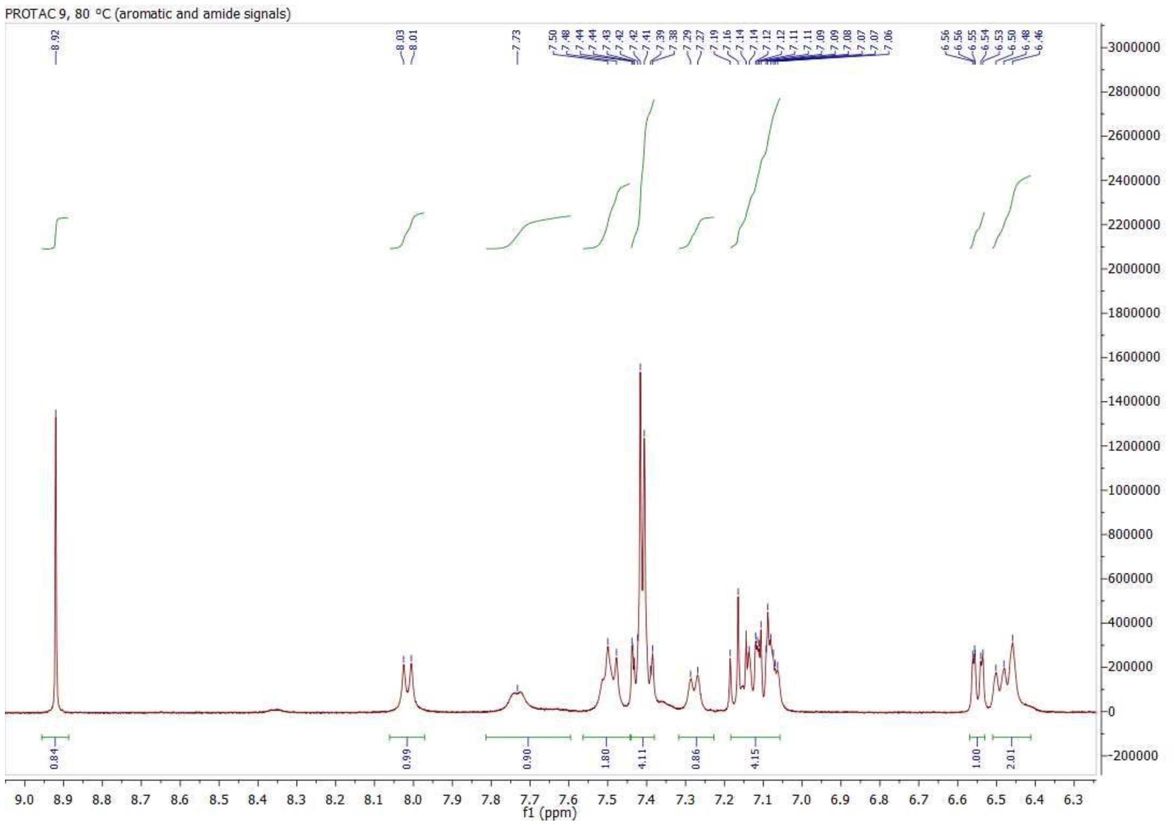

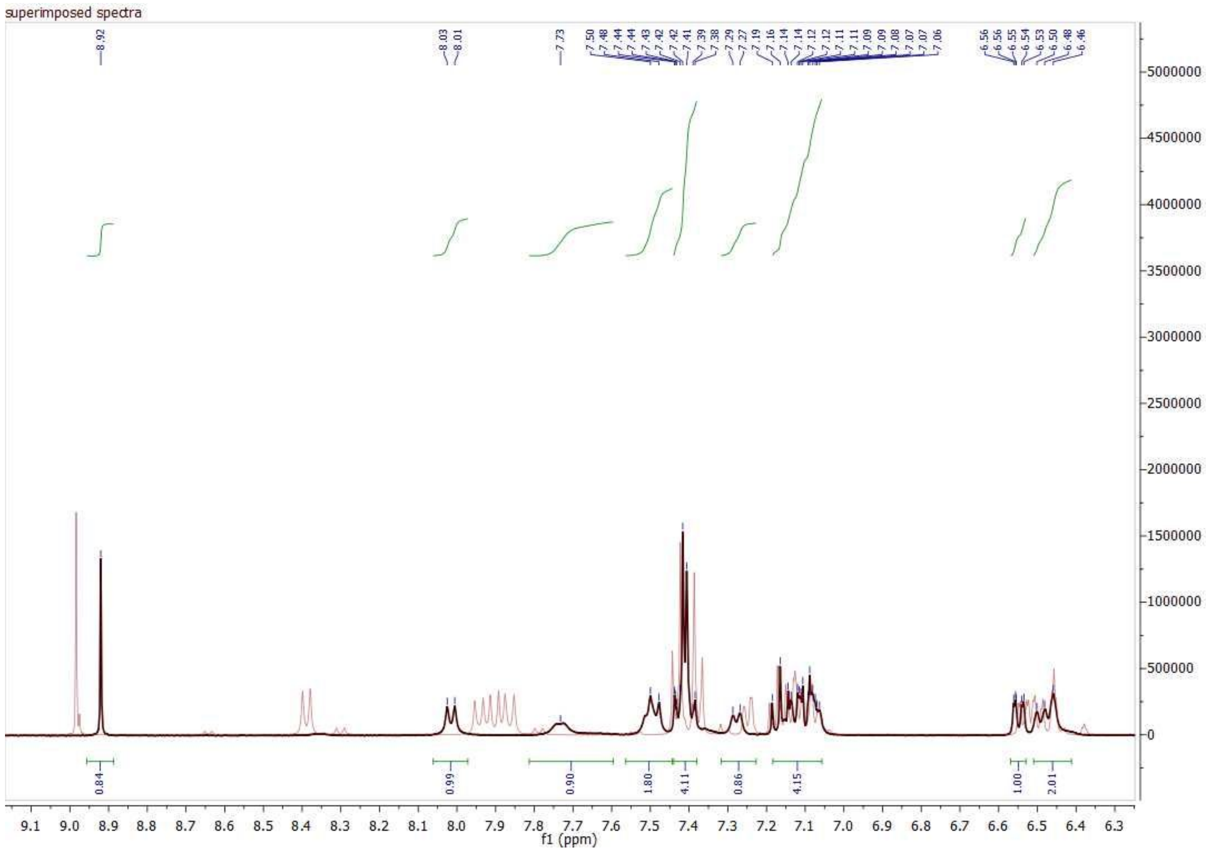
superimposed spectra
f1 (ppm)

HRMS of PROTAC **9** (CST626)

CST 626

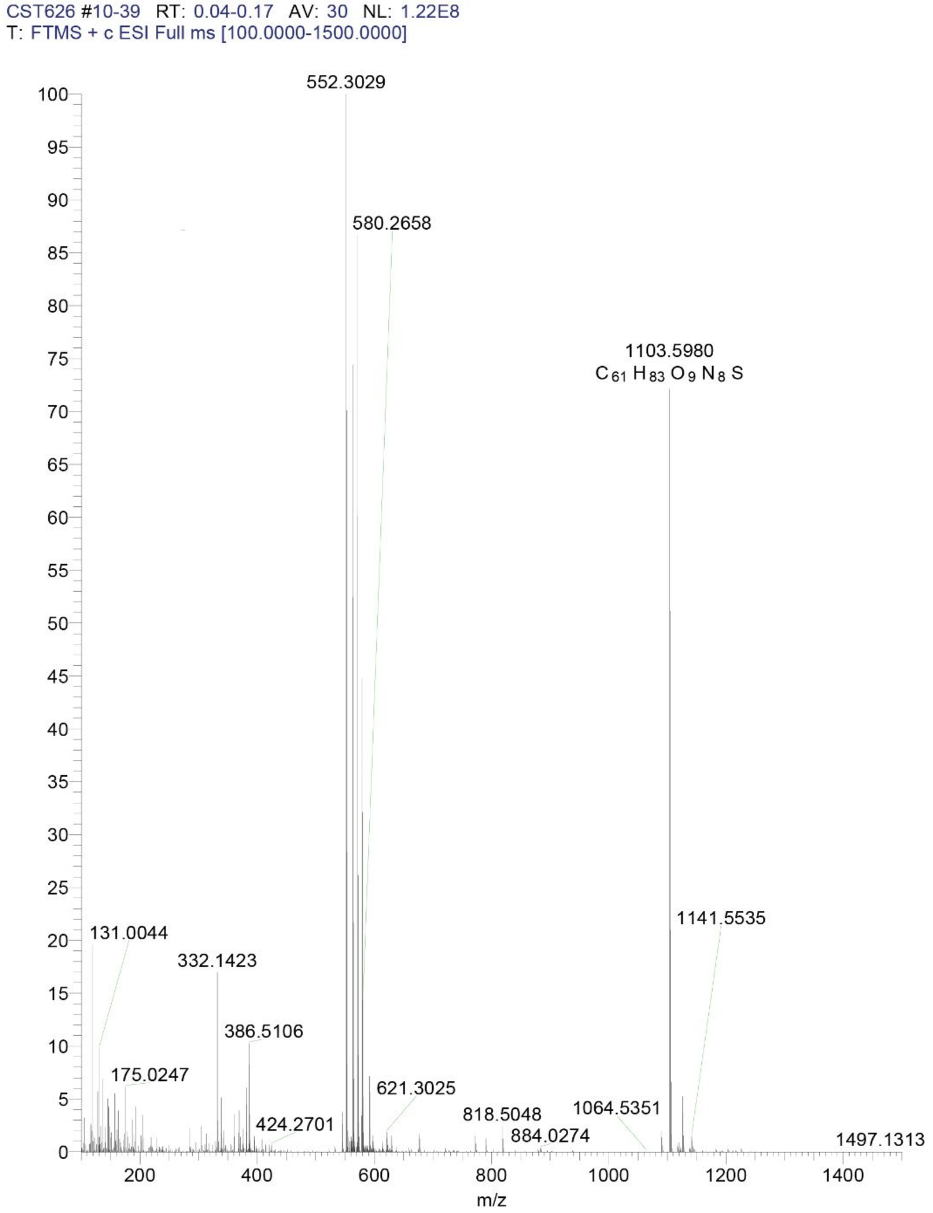

Elemental composition search on mass 1103.5980

m/z= 1098.5980-1108.5980

m/z	Theo. Mass	Delta (ppm)	RDB equiv.	Composition
1103.59798	1103.59982	-1.67	24.5	$C_{61}H_{83}O_9N_8S$

^{1}H and ^{13}C NMR spectra of PROTAC **25** (SAB141)

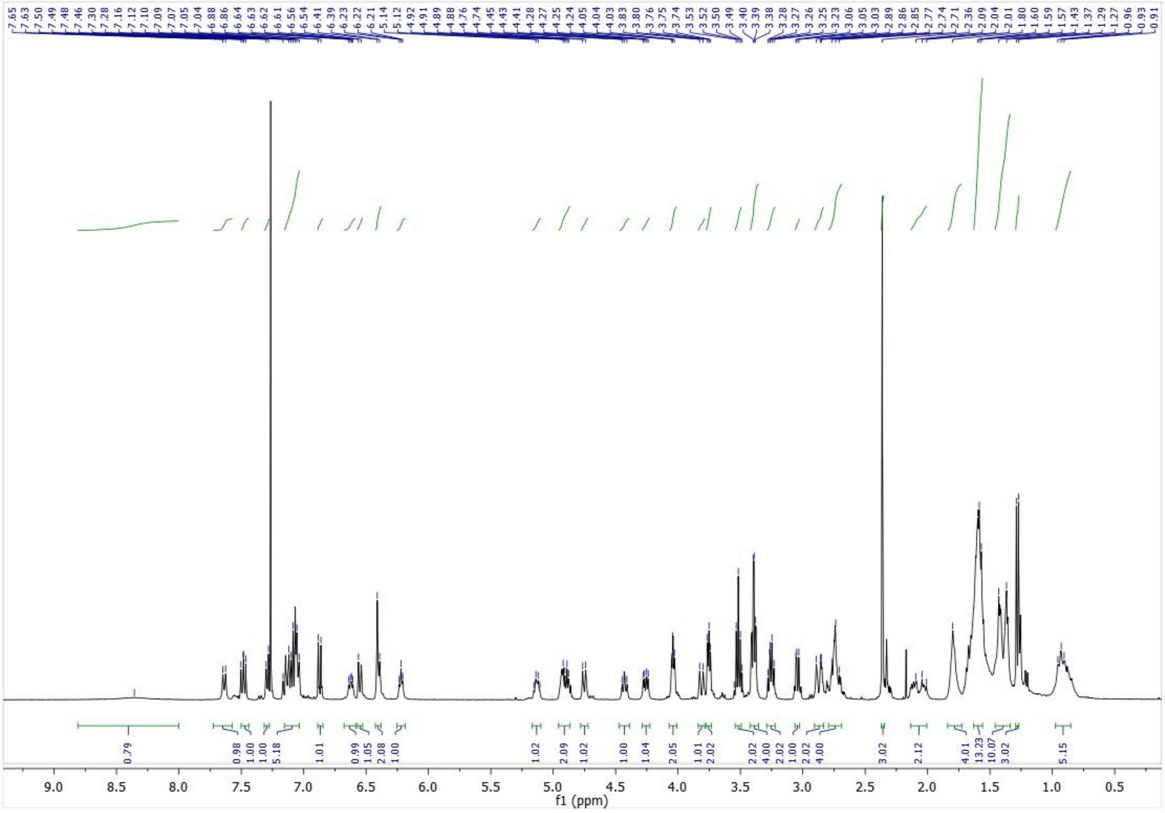

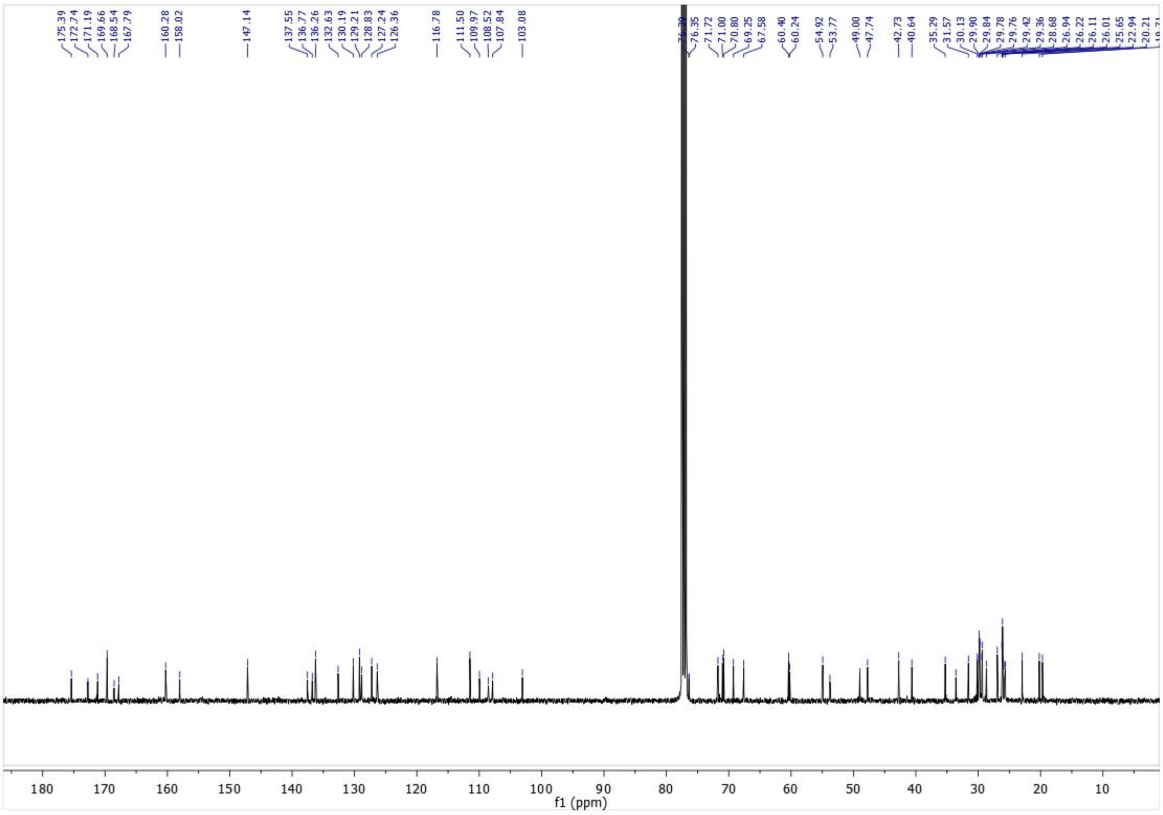

HRMS of PROTAC **25** (SAB141)

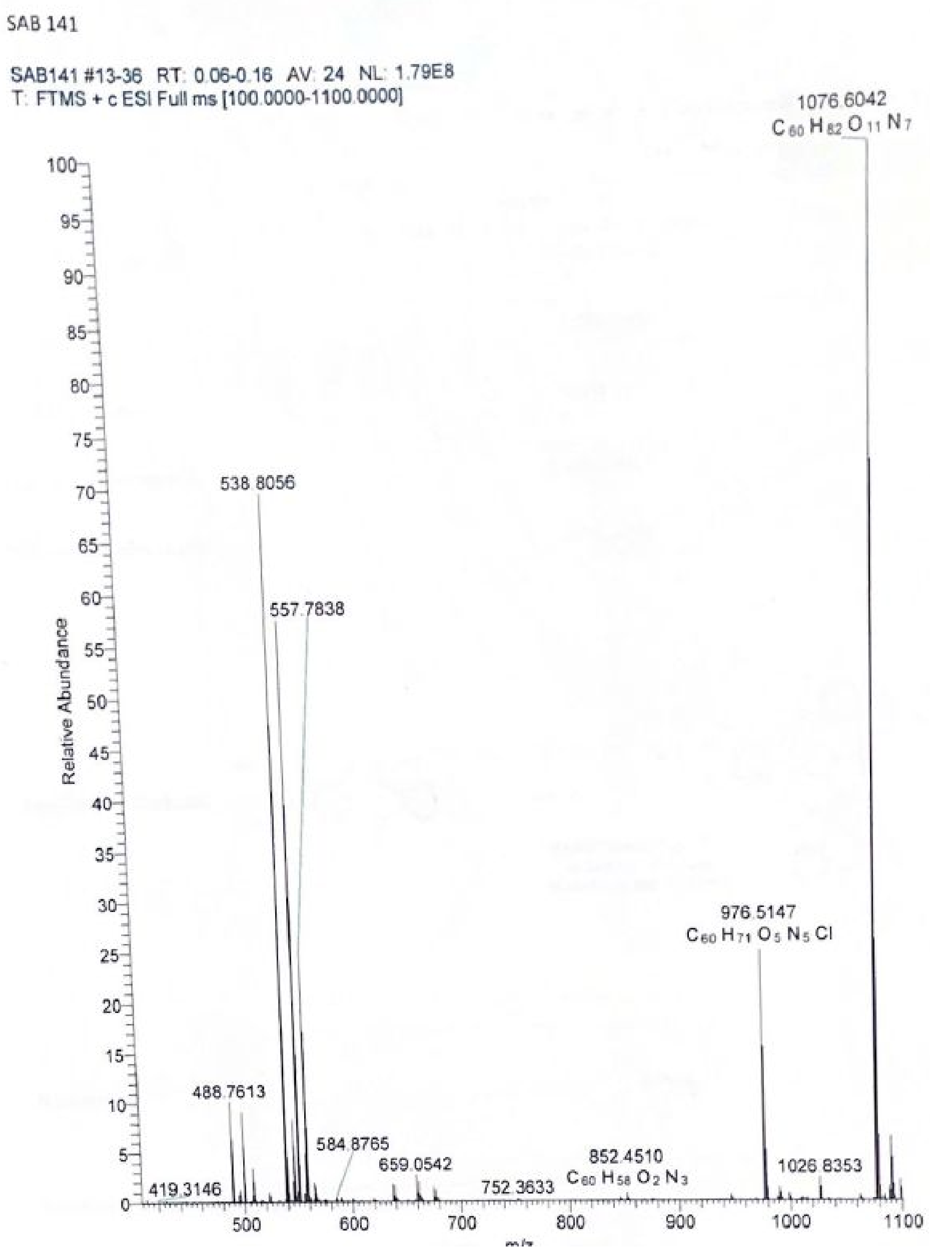

LC/MS analysis of PROTAC **1** (CST568)

Please note that at the start of LC/MS traces (up to 0,7 min), a small signal for non-retained solutes is present, most likely resulting from the difference in composition of the mobile phase and solvent mixture in which the analyte was dissolved. The integral of the signal was ignored.

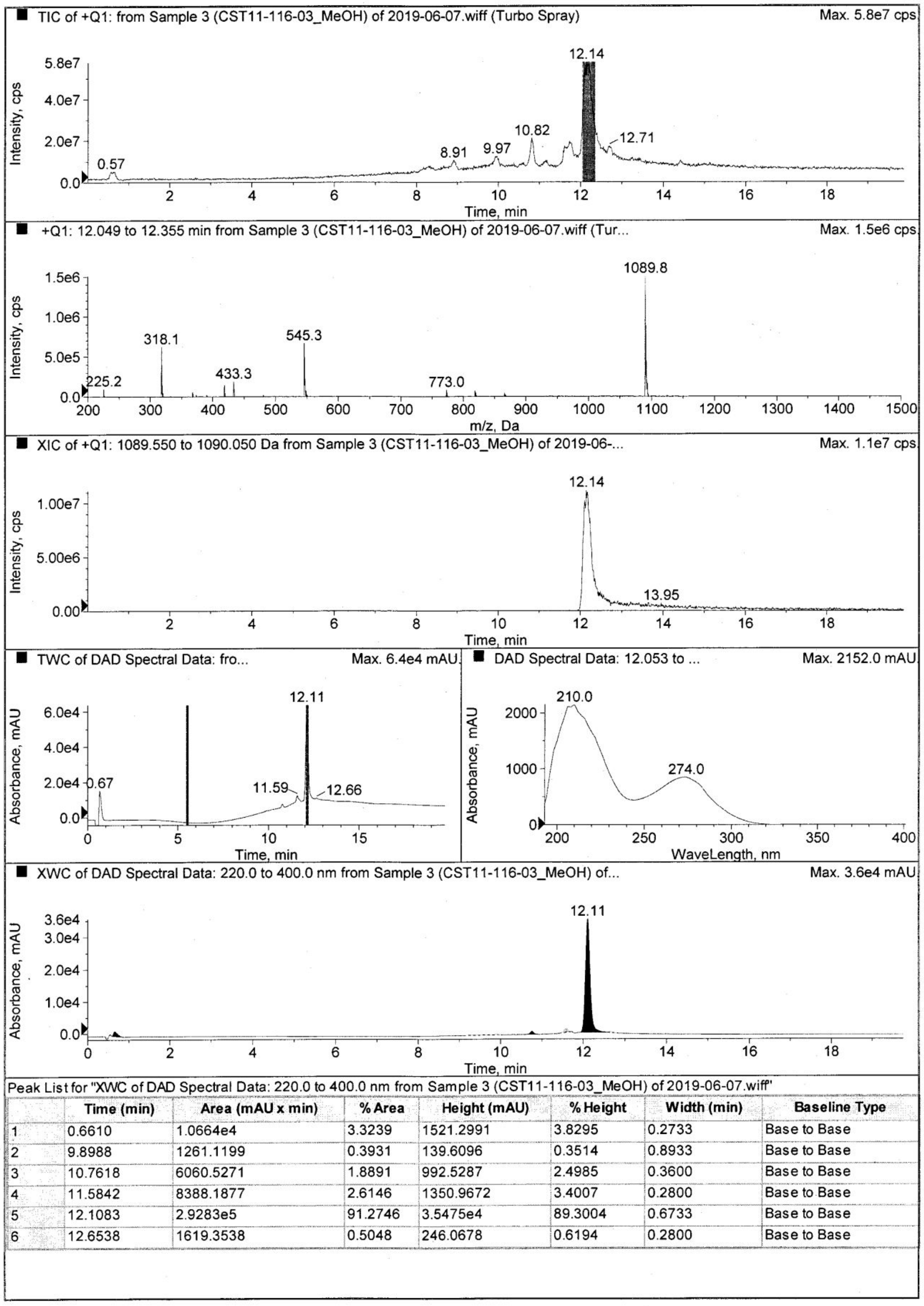

Peak List for "XWC of DAD Spectral Data: 220.0 to 400.0 nm from Sample 3 (CST11-116-03_MeOH) of 2019-06-07.wiff"

	Time (min)	Area (mAU x min)	% Area	Height (mAU)	% Height	Width (min)	Baseline Type
1	0.6610	1.0664e4	3.3239	1521.2991	3.8295	0.2733	Base to Base
2	9.8988	1261.1199	0.3931	139.6096	0.3514	0.8933	Base to Base
3	10.7618	6060.5271	1.8891	992.5287	2.4985	0.3600	Base to Base
4	11.5842	8388.1877	2.6146	1350.9672	3.4007	0.2800	Base to Base
5	12.1083	2.9283e5	91.2746	3.5475e4	89.3004	0.6733	Base to Base
6	12.6538	1619.3538	0.5048	246.0678	0.6194	0.2800	Base to Base

LC/MS analysis of PROTAC **2** (CST533)

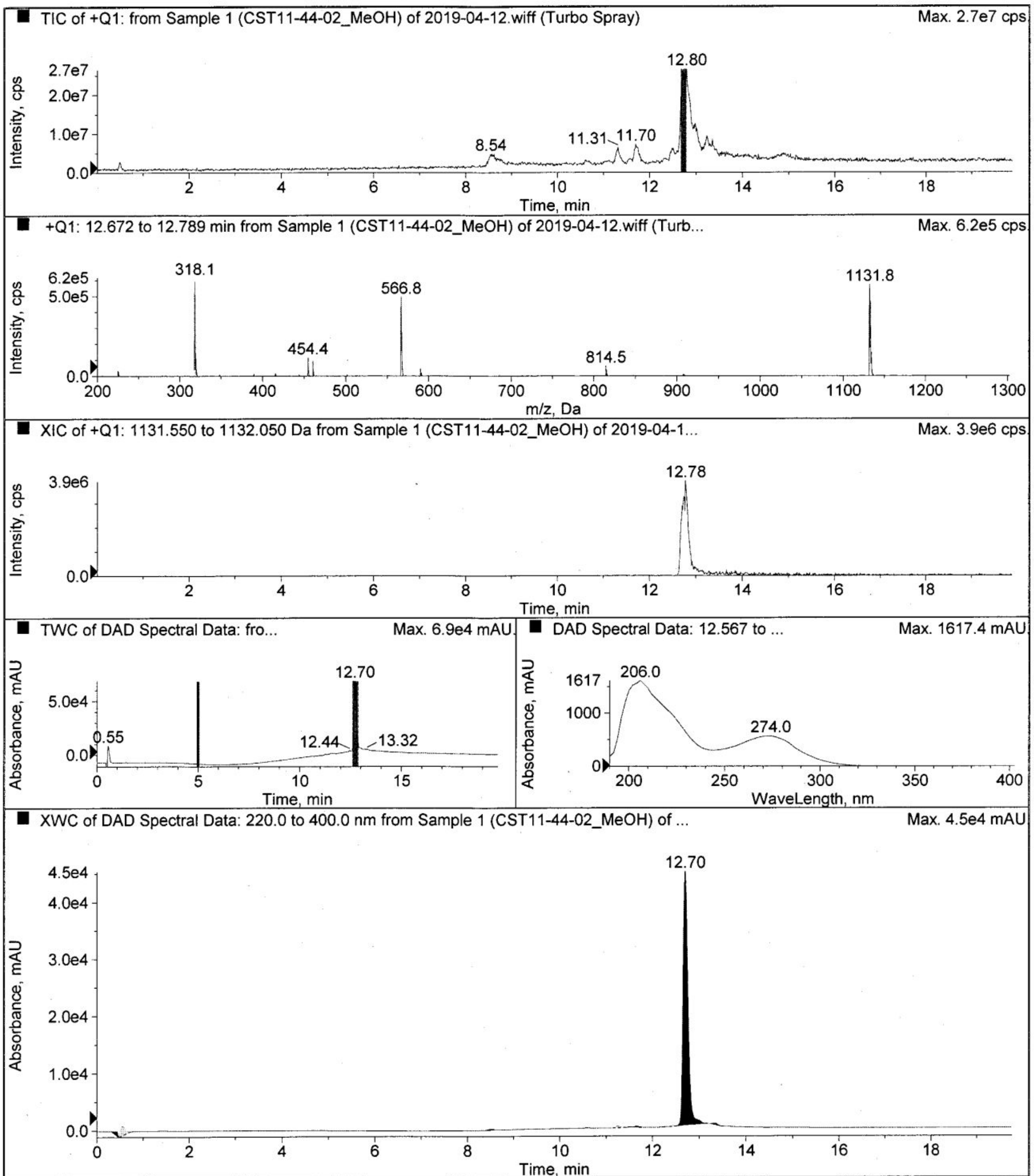

Peak List for "XWC of DAD Spectral Data: 220.0 to 400.0 nm from Sample 1 (CST11-44-02_MeOH) of 2019-04-12.wiff"

	Time (min)	Area (mAU x min)	% Area	Height (mAU)	% Height	Width (min)	Baseline Type
1	0.3521	3839.0122	1.0704	260.8431	0.5448	0.1600	Base to Base
2	0.5493	1.1945e4	3.3306	1639.3386	3.4237	0.2600	Base to Base
3	8.4890	916.8480	0.2556	132.3482	0.2764	0.2333	Base to Base
4	11.2465	1777.3864	0.4956	339.5741	0.7092	0.2400	Base to Base
5	11.6483	1055.5685	0.2943	187.7160	0.3920	0.2133	Base to Base
6	12.4409	1094.9316	0.3053	250.2517	0.5226	0.1533	Base to Base
7	12.6971	3.3598e5	93.6813	4.4692e4	93.3379	0.6067	Base to Base
8	13.1811	557.5594	0.1555	145.8427	0.3046	0.1267	Base to Base
9	13.3356	1475.4676	0.4114	234.0213	0.4887	0.2000	Base to Base

LC/MS analysis of PROTAC **3** (CST565)

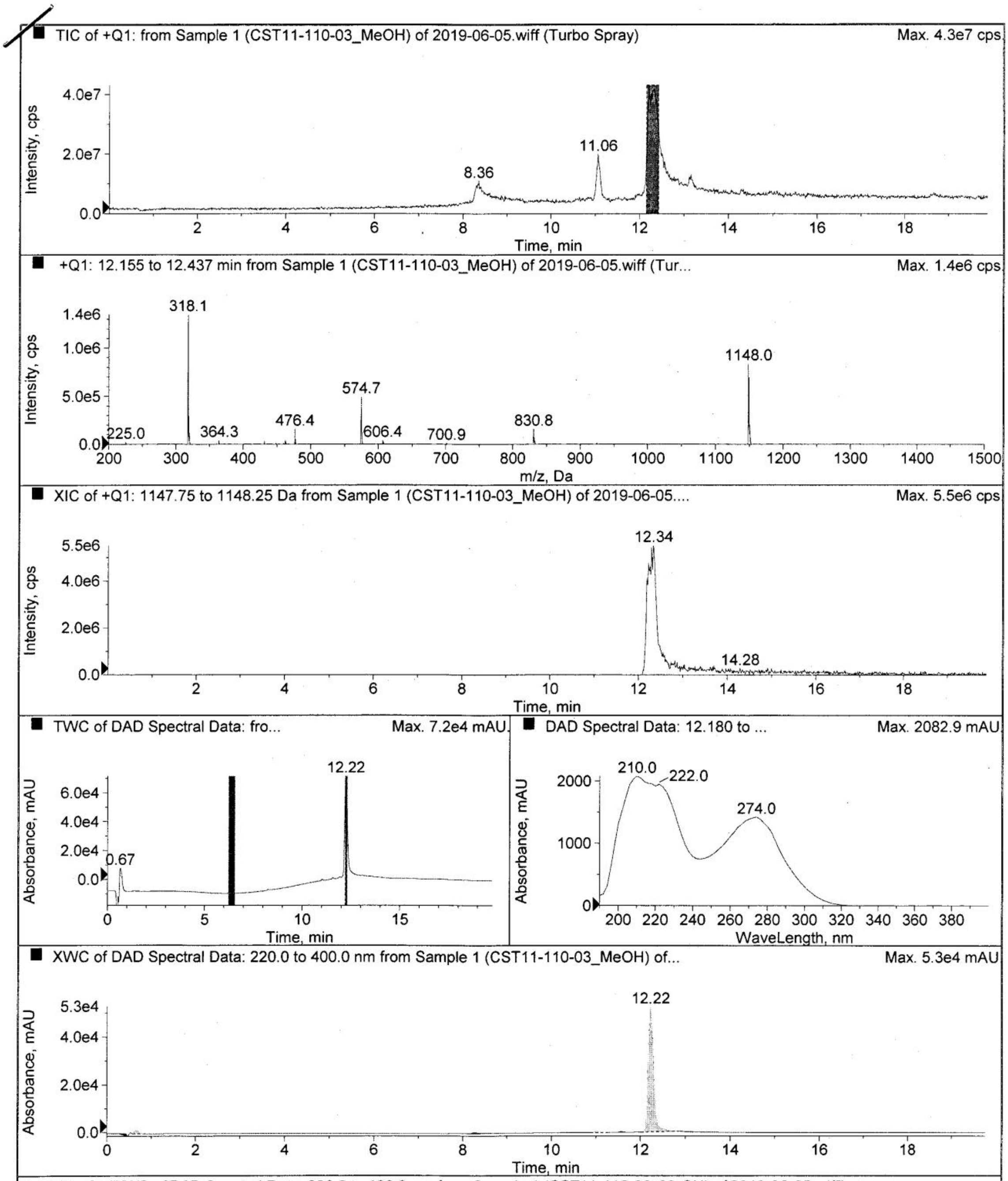

Peak List for "XWC of DAD Spectral Data: 220.0 to 400.0 nm from Sample 1 (CST11-110-03_MeOH) of 2019-06-05.wiff"

	Time (min)	Area (mAU x min)	% Area	Height (mAU)	% Height	Width (min)	Baseline Type
1	0.4107	4753.7720	1.0061	771.9288	1.3706	0.1867	Base to Base
2	0.6556	1.0815e4	2.2889	1507.9647	2.6774	0.2800	Base to Base
3	8.2628	4223.1943	0.8938	356.6044	0.6332	0.5867	Base to Base
4	11.0169	3053.0571	0.6462	545.9819	0.9694	0.3667	Base to Base
5	11.5654	2066.7561	0.4374	377.3678	0.6700	0.4333	Base to Base
6	12.2235	4.4676e5	94.5539	5.2612e4	93.4147	0.8267	Base to Base
7	13.0897	820.7133	0.1737	149.0490	0.2646	0.2200	Base to Base

LC/MS analysis of PROTAC **4** (CST536)

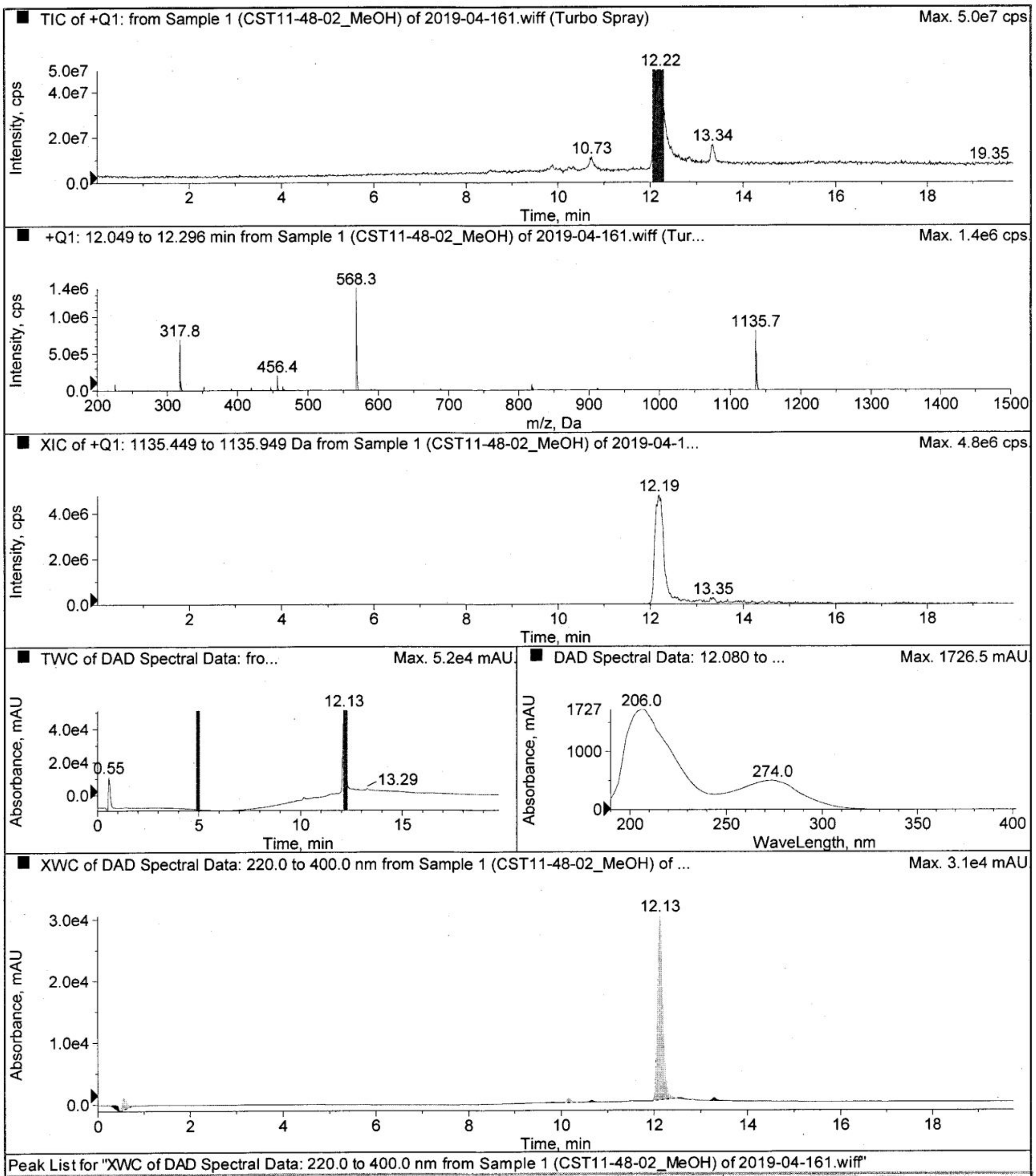

Peak List for "XWC of DAD Spectral Data: 220.0 to 400.0 nm from Sample 1 (CST11-48-02_MeOH) of 2019-04-161.wiff"

	Time (min)	Area (mAU x min)	% Area	Height (mAU)	% Height	Width (min)	Baseline Type
1	0.3749	4648.5169	1.7264	521.7619	1.5270	0.1933	Base to Base
2	0.5599	1.3090e4	4.8615	1862.0089	5.4494	0.2667	Base to Base
3	9.8215	577.4009	0.2144	92.7544	0.2715	0.3867	Base to Base
4	10.1630	4082.1807	1.5161	675.9144	1.9781	0.3800	Base to Base
5	10.6573	1600.2705	0.5943	256.7073	0.7513	0.4000	Base to Base
6	12.1256	2.4161e5	89.7302	3.0052e4	87.9521	0.5733	Base to Base
7	12.5667	689.6768	0.2561	109.6031	0.3208	0.2000	Base to Base
8	12.8046	554.7394	0.2060	108.7023	0.3181	0.1867	Base to Base
9	13.2852	2409.9852	0.8950	489.2071	1.4317	0.3600	Base to Base

LC/MS analysis of PROTAC **5** (CST564)

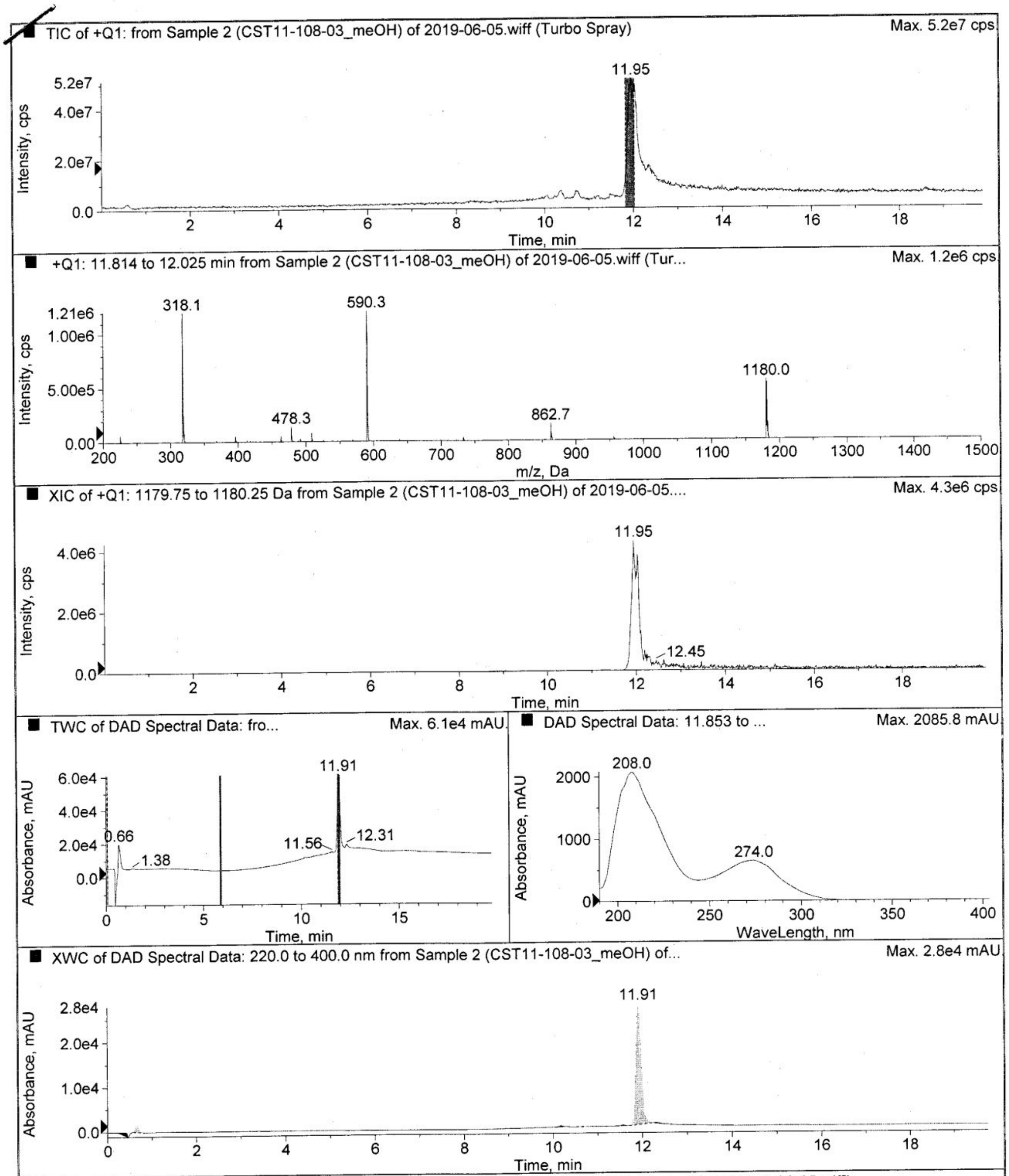

Peak List for "XWC of DAD Spectral Data: 220.0 to 400.0 nm from Sample 2 (CST11-108-03_meOH) of 2019-06-05.wiff"

	Time (min)	Area (mAU x min)	% Area	Height (mAU)	% Height	Width (min)	Baseline Type
1	0.4104	6285.5337	2.6320	843.6242	2.8006	0.2333	Base to Base
2	0.6499	8258.8157	3.4583	1281.5516	4.2544	0.2600	Base to Base
3	10.1851	1326.3861	0.5554	266.0533	0.8832	0.2200	Base to Base
4	10.6760	520.0643	0.2178	89.7684	0.2980	0.2800	Base to Base
5	11.5615	1316.2117	0.5512	212.1769	0.7044	0.3933	Base to Base
6	11.9142	2.2006e5	92.1502	2.7292e4	90.6016	0.5533	Base to Base
7	12.3066	1039.0808	0.4351	137.9034	0.4578	0.2400	Base to Base

LC/MS analysis of PROTAC **6** (CST496)

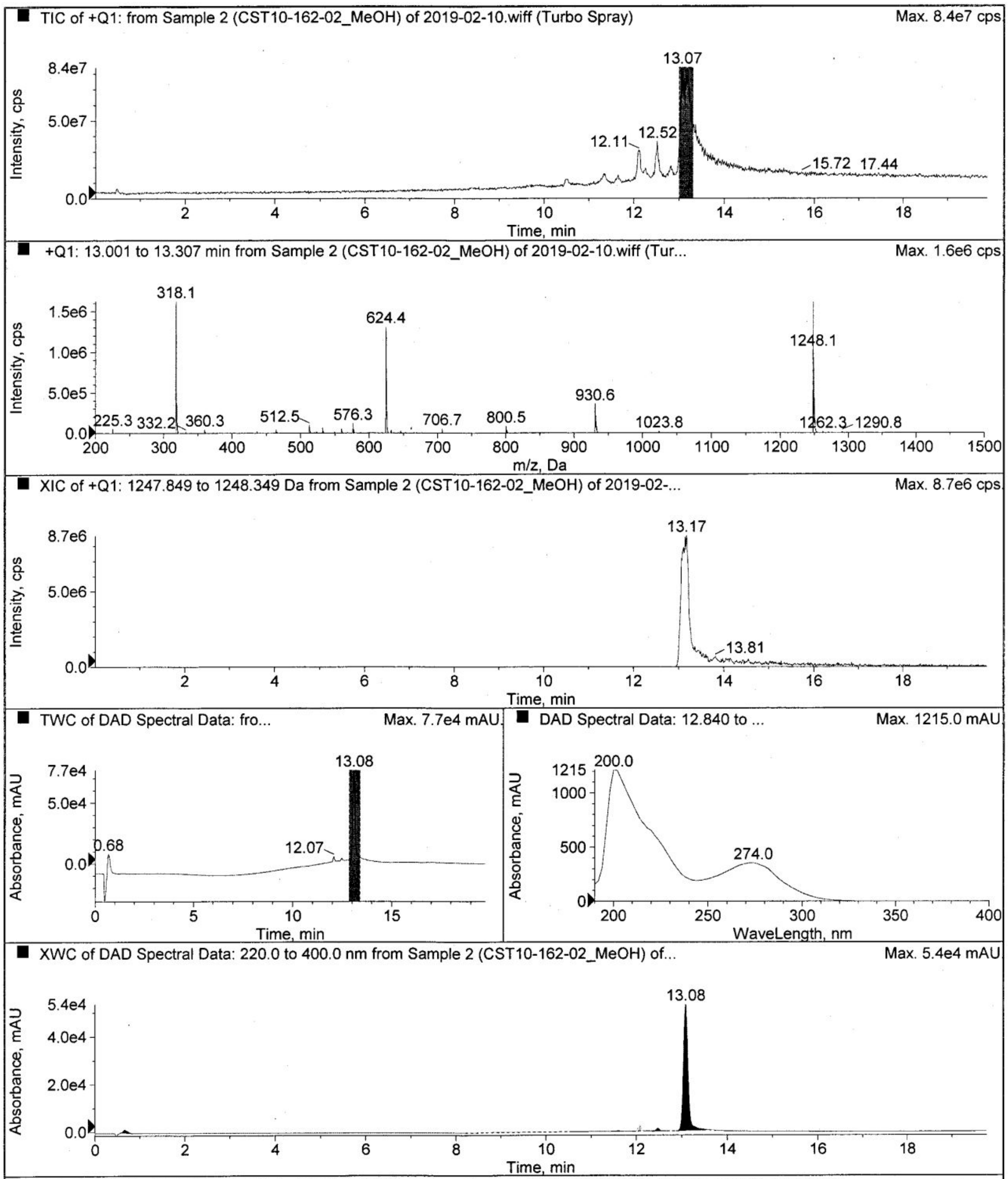

Peak List for "XWC of DAD Spectral Data: 220.0 to 400.0 nm from Sample 2 (CST10-162-02_MeOH) of 2019-02-10.wiff"

	Time (min)	Area (mAU x min)	% Area	Height (mAU)	% Height	Width (min)	Baseline Type
1	0.6661	7757.0317	1.8649	1180.6778	2.0140	0.2400	Base to Base
2	10.4546	3310.7520	0.7960	141.3379	0.2411	2.3667	Base to Base
3	11.5993	624.6833	0.1502	133.4930	0.2277	0.2267	Base to Base
4	12.0689	1.1086e4	2.6653	2516.4004	4.2925	0.2600	Base to Base
5	12.4697	5284.6675	1.2705	1144.9596	1.9531	0.3267	Base to Base
6	12.7749	545.3739	0.1311	121.8583	0.2079	0.1867	Base to Base
7	13.0801	3.8674e5	92.9804	5.3270e4	90.8690	0.7200	Base to Base
8	13.6416	588.8742	0.1416	114.1556	0.1947	0.1533	Base to Base

LC/MS analysis of PROTAC **7** (CST563)

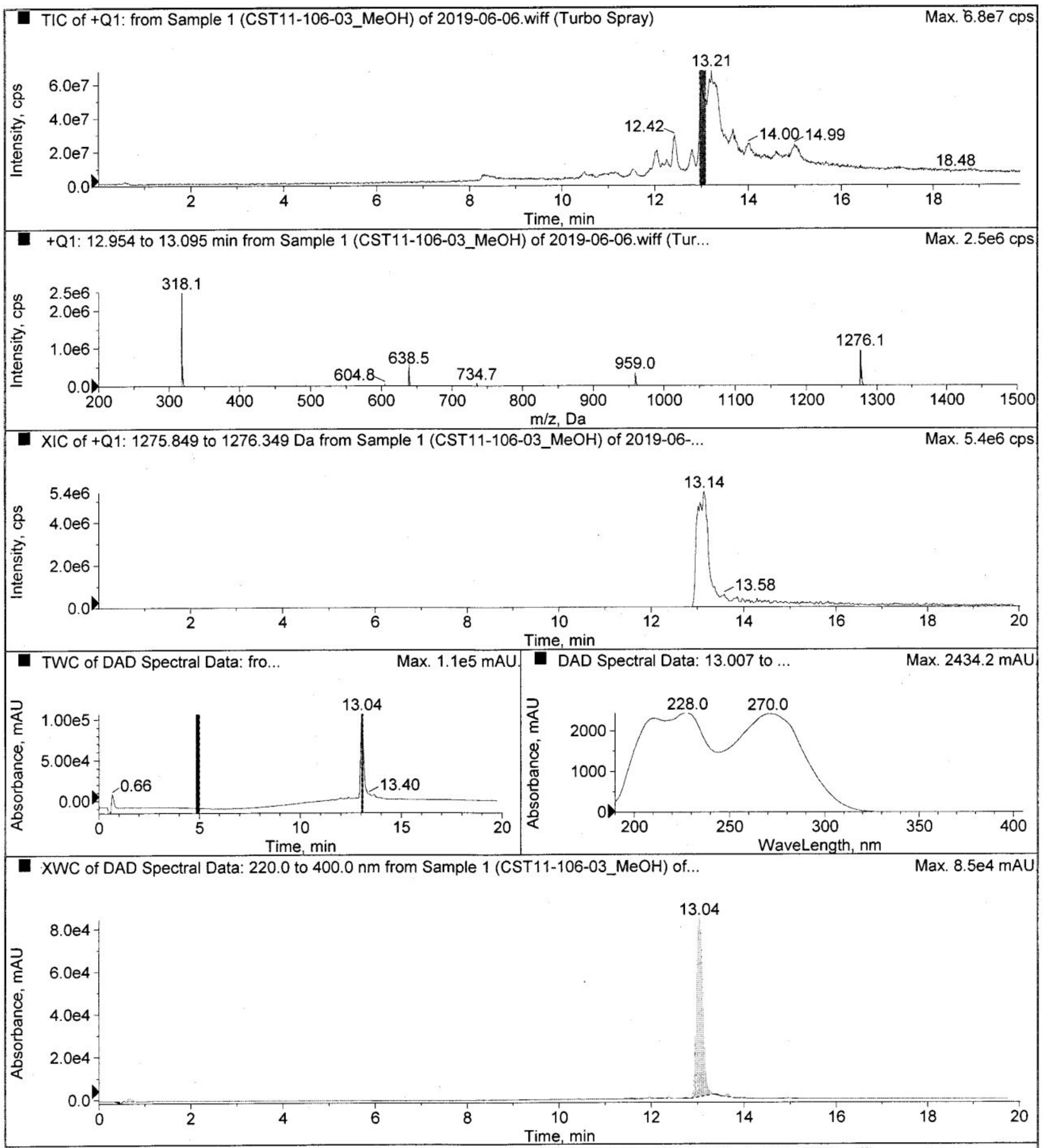

Peak List for "XWC of DAD Spectral Data: 220.0 to 400.0 nm from Sample 1 (CST11-106-03_MeOH) of 2019-06-06.wiff"

	Time (min)	Area (mAU x min)	% Area	Height (mAU)	% Height	Width (min)	Baseline Type
1	0.4174	3866.8686	0.4568	781.0977	0.8622	0.1467	Base to Base
2	0.6580	1.0402e4	1.2288	1534.9824	1.6943	0.2867	Base to Base
3	11.5798	1162.1448	0.1373	250.6919	0.2767	0.2067	Base to Base
4	11.9706	4652.8223	0.5497	859.9643	0.9492	0.2067	Base to Base
5	12.2123	1369.3952	0.1618	252.3955	0.2786	0.2133	Base to Base
6	12.3738	4822.7233	0.5697	1047.5626	1.1563	0.2400	Base to Base
7	12.7450	1500.0442	0.1772	316.0488	0.3488	0.1867	Base to Base
8	13.0370	8.0825e5	95.4813	8.3806e4	92.5037	0.5000	Base to Base
9	13.3999	2041.6020	0.2412	408.6496	0.4511	0.1533	Base to Base
10	13.6366	8433.1560	0.9962	1340.0420	1.4791	0.2467	Base to Base

LC/MS analysis of PROTAC **8** (CST566)

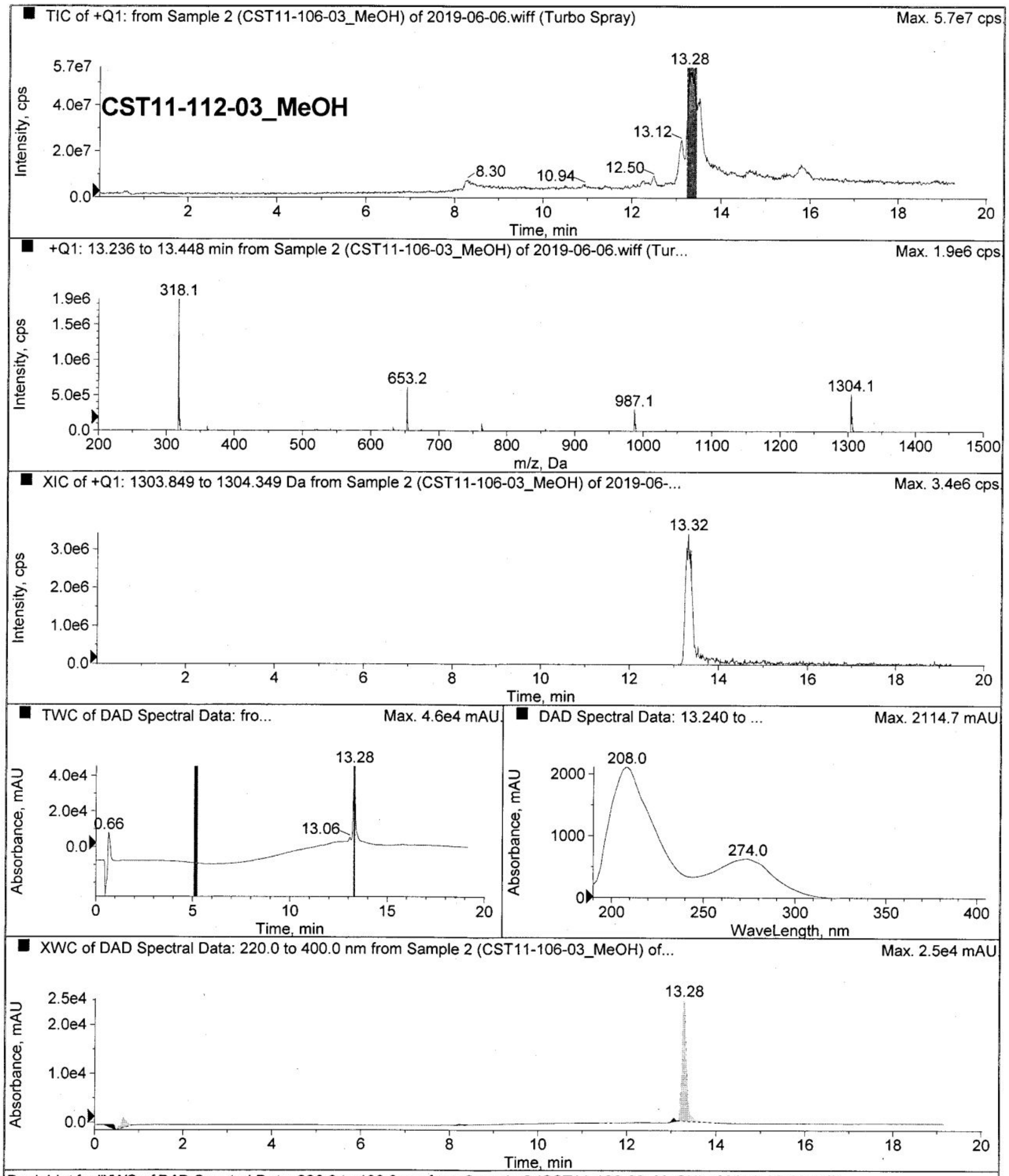

Peak List for "XWC of DAD Spectral Data: 220.0 to 400.0 nm from Sample 2 (CST11-106-03_MeOH) of 2019-06-06.wiff"

	Time (min)	Area (mAU x min)	% Area	Height (mAU)	% Height	Width (min)	Baseline Type
1	0.4147	7816.7859	3.7565	848.0628	2.9588	0.2800	Base to Base
2	0.6535	1.8154e4	8.7243	1978.2395	6.9020	0.3733	Base to Base
3	8.2257	1117.6204	0.5371	115.1273	0.4017	0.3667	Base to Base
4	11.5728	628.2973	0.3019	133.4577	0.4656	0.2200	Base to Base
5	13.0585	3472.1369	1.6686	687.5649	2.3989	0.1867	Base to Base
6	13.2795	1.7690e5	85.0117	2.4900e4	86.8730	0.9000	Base to Base

LC/MS analysis of PROTAC **9** (CST626)

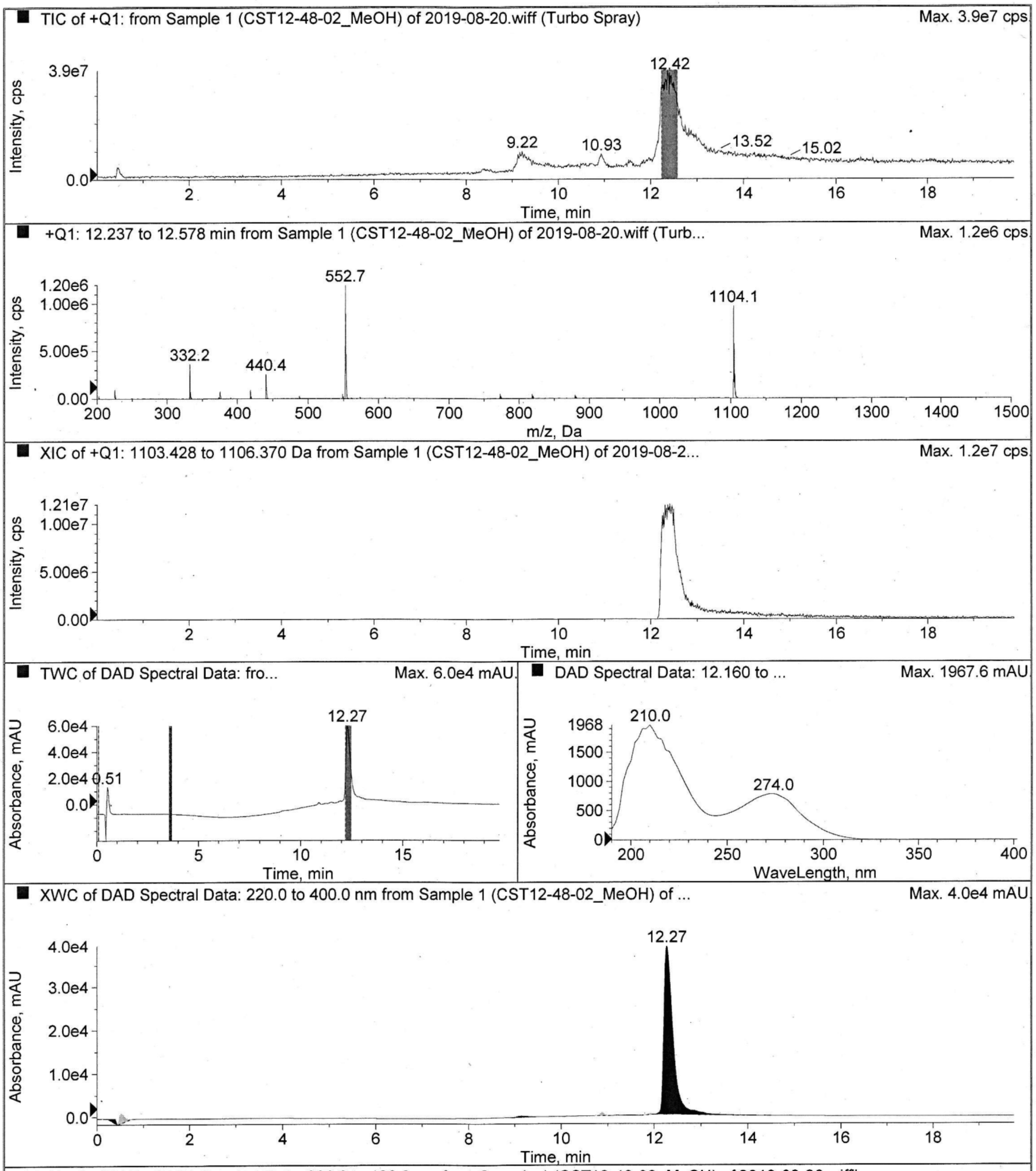

Peak List for "XWC of DAD Spectral Data: 220.0 to 400.0 nm from Sample 1 (CST12-48-02_MeOH) of 2019-08-20.wiff"

	Time (min)	Area (mAU x min)	% Area	Height (mAU)	% Height	Width (min)	Baseline Type
1	0.2824	6365.0877	1.0712	327.3959	0.7580	0.2067	Base to Base
2	0.5219	1.7273e4	2.9068	2294.3476	5.3122	0.3000	Base to Base
3	9.1371	4884.7100	0.8220	316.3482	0.7324	0.5867	Base to Base
4	10.8807	4702.3624	0.7913	716.4160	1.6587	0.4133	Base to Base
5	11.4962	1122.7480	0.1889	119.4740	0.2766	0.3667	Base to Base
6	11.9181	1835.9066	0.3090	267.2127	0.6187	0.2933	Base to Base
7	12.2695	5.5804e5	93.9107	3.9149e4	90.6433	2.5800	Base to Base

**Sequence Name:**	SingleSample		
**Data file:**	Gü3582_ACN.dx	**Project Name:**	Single Quad
**Sample name:**	Gü3582_ACN	**Operator :**	SYSTEM
**Instrument:**	Single Quad	**Acquired on:**	2022-01-10 08:28:55+01:00
**Inj. volume:**	4.000	**Location:**	P1-D6
**Acq. method:**	00-new_wasser-acn_standard_100-1500_BD-20min.amx		
**Processing method:**	MS_standard_plot.pmx		

MS1 -TIC SCAN ESI Frag=135V Gain=1,0

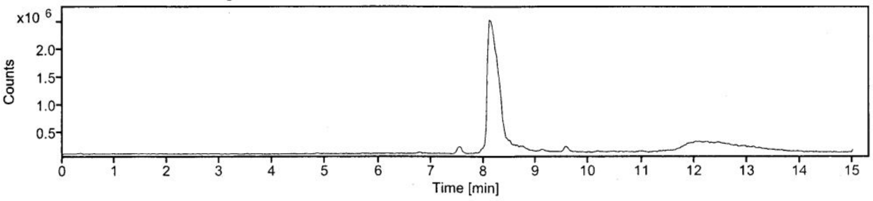

MS1 +TIC SCAN ESI Frag=135V Gain=1,0

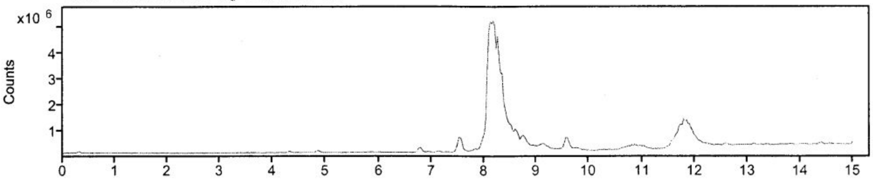

DAD1 Total WL (220.0:600.0 nm)

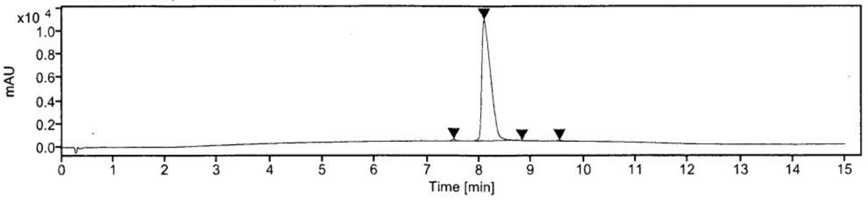

MS1 +EIC(1103.6-1104.6) ESI Frag=135V Gain=1.0

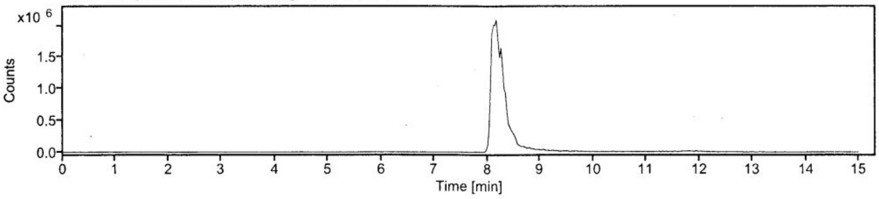

MS1 -EIC(1101.5-1102.5) ESI Frag=135V Gain=1.0

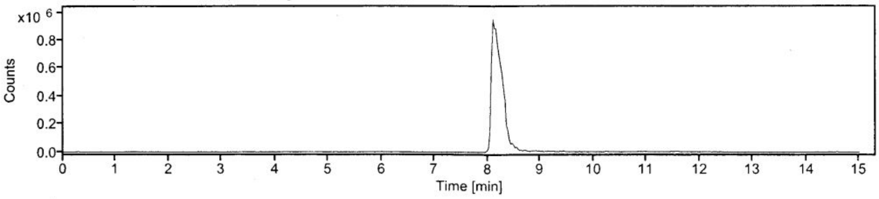

Signal: DAD1 Total WL (220.0:600.0 nm)

RT [min]	Peak MS Base Peak m/z	Area	Area%	Max Peak%	Height
7.506		772.7466	0.6283	0.635	151.202
8.090	1101.800	121761.2964	99.0061	100.000	10461.359
8.820		53.0523	0.0431	0.044	15.392
9.539		396.5268	0.3224	0.326	69.048
	Sum	122983.6222			

Signal: MS1 +TIC SCAN ESI Frag=135V Gain=1,0

RT [min]	Peak MS Base Peak m/z	Area	Area%	Max Peak%	Height
8.175	1103.900	76533723.3429	100.0000	100.000	4613321.610
	Sum	76533723.3429			

Signal: MS1 -TIC SCAN ESI Frag=135V Gain=1,0

RT [min]	Peak MS Base Peak m/z	Area	Area%	Max Peak%	Height
8.121	1101.800	32726263.5180	100.0000	100.000	2417016.232
	Sum	32726263.5180			

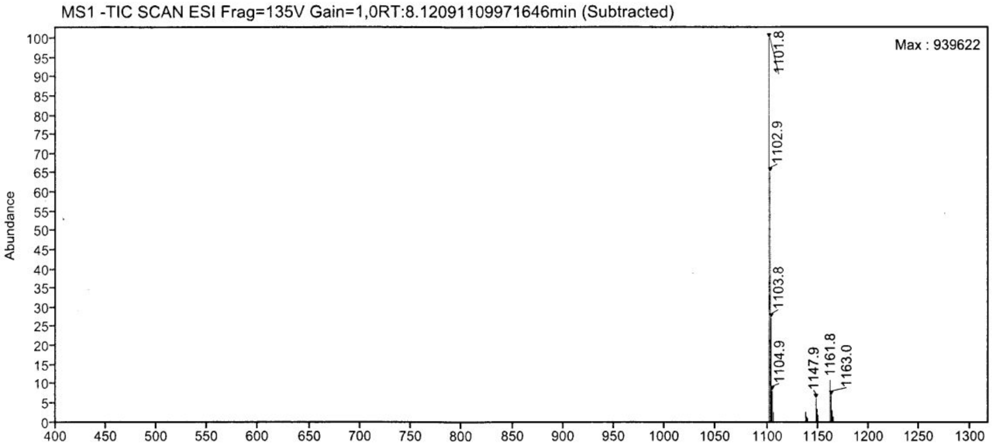

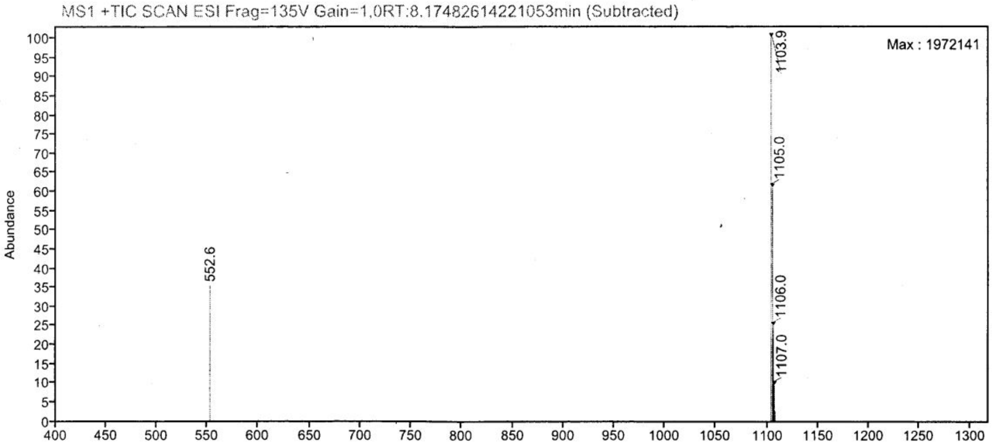

LC/MS analysis of PROTAC **10a** (CST625)

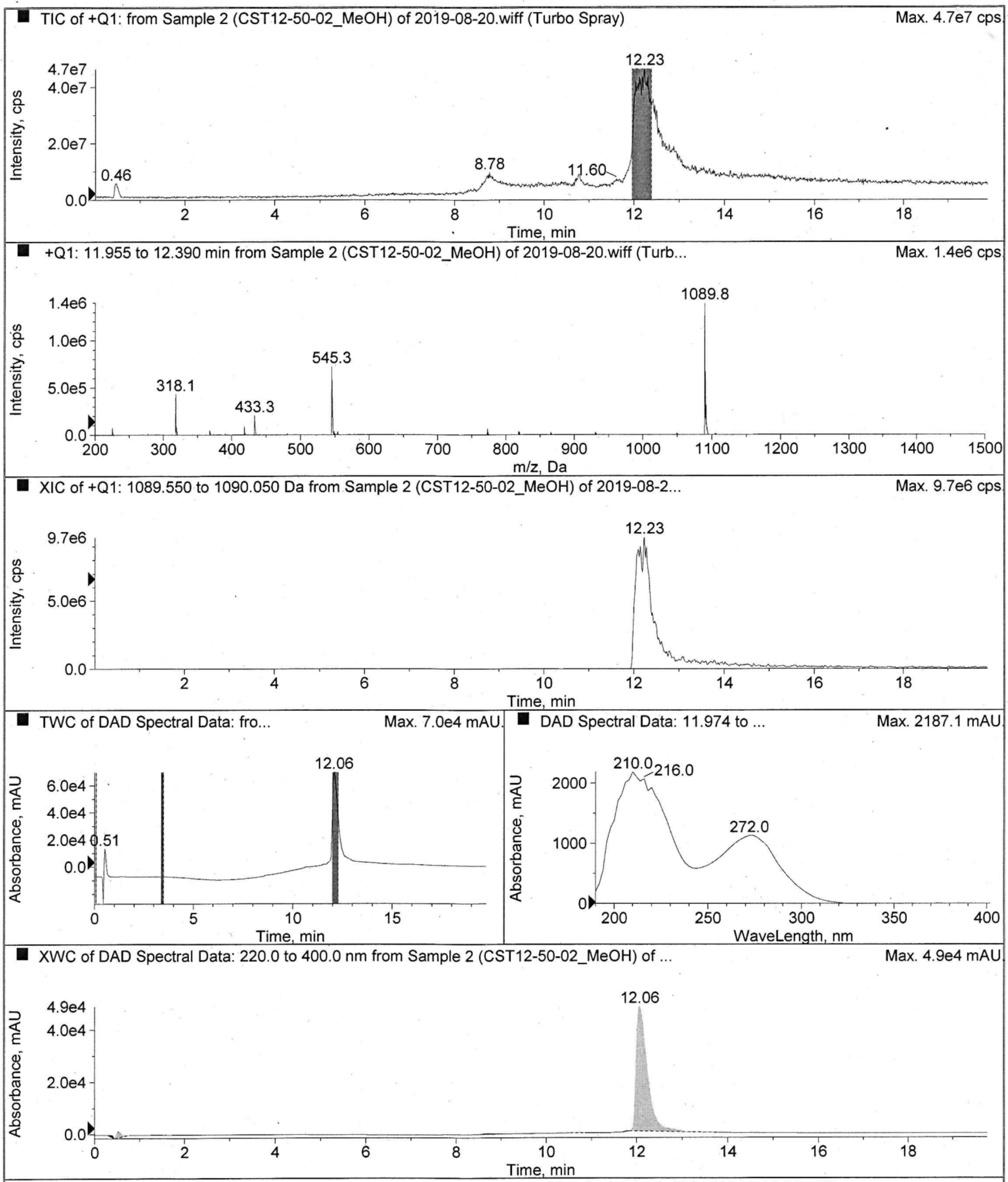

Peak List for "XWC of DAD Spectral Data: 220.0 to 400.0 nm from Sample 2 (CST12-50-02_MeOH) of 2019-08-20.wiff"

	Time (min)	Area (mAU x min)	% Area	Height (mAU)	% Height	Width (min)	Baseline Type
1	0.3117	3762.4721	0.4385	165.7917	0.3238	0.1467	Base to Base
2	0.5228	1.7778e4	2.0720	2286.4088	4.4652	0.3200	Base to Base
3	8.6893	4092.5582	0.4770	260.3007	0.5083	0.5800	Base to Base
4	10.6505	2711.1054	0.3160	300.7216	0.5873	0.3133	Base to Base
5	11.7949	1054.6117	0.1229	189.9478	0.3710	0.1600	Base to Base
6	12.0580	8.2860e5	96.5736	4.8002e4	93.7445	1.9333	Base to Base

LC/MS analysis of PROTAC **10b** (CST1124)

**Sequence Name:**	SingleSample		
**Data file:**	CST1124_ACN.dx	**Project Name:**	Single Quad
**Sample name:**	CST1124_ACN	**Operator :**	SYSTEM
**Instrument:**	Single Quad	**Acquired on:**	2023-01-19 08:57:16+01:00
**Inj. volume:**	2.000	**Location:**	P1-E2
**Acq. method:**	00-new_wasser-acn_standard_100-1500_BD-20min.amx		
**Processing method:**	MS_standard_plot.pmx		

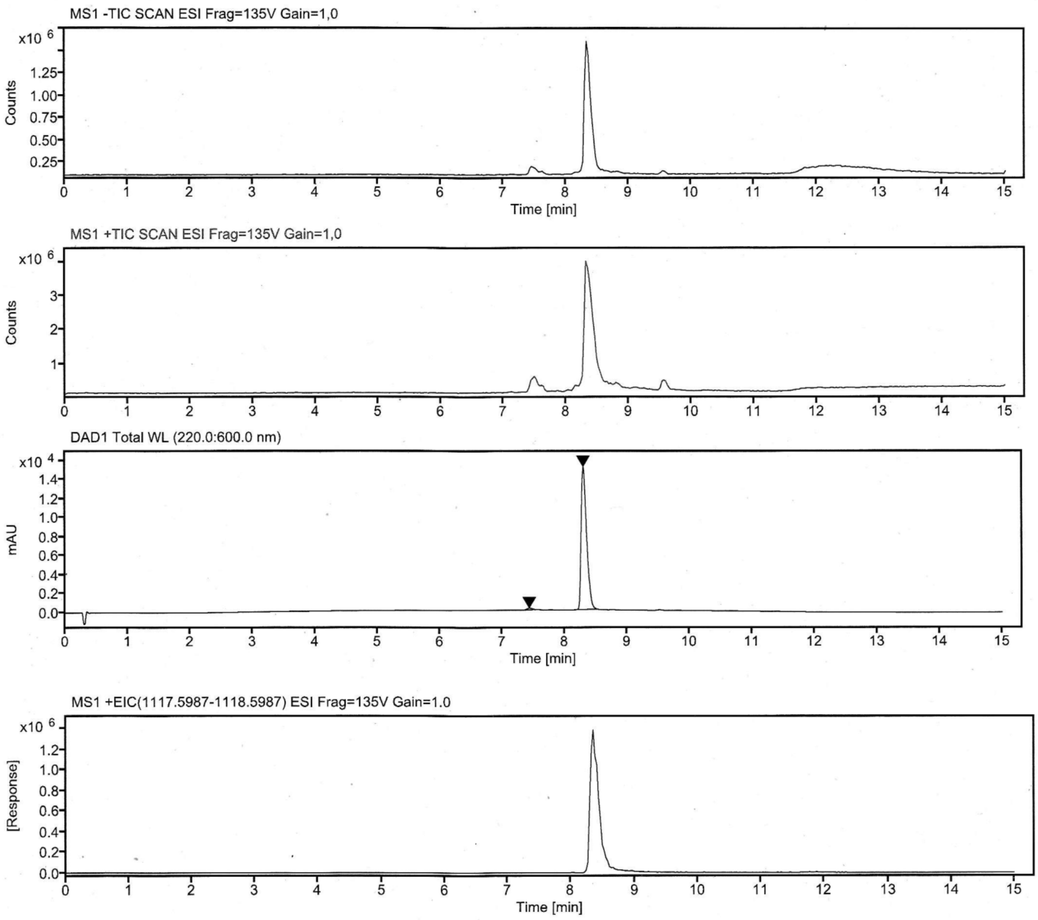

Signal: DAD1 Total WL (220.0:600.0 nm)

RT [min]	Peak MS Base Peak m/z	Area	Area%	Max Peak%	Height
7.439		1031.3861	1.1320	1.145	171.797
8.302	1117.900	90082.3453	98.8680	100.000	14984.265
	Sum	91113.7314			

Signal: MS1 +TIC SCAN ESI Frag=135V Gain=1,0

RT [min]	Peak MS Base Peak m/z	Area	Area%	Max Peak%	Height
8.331	1117.900	39751497.0730	100.0000	100.000	3965412.117
	Sum	39751497.0730			

Signal: MS1 -TIC SCAN ESI Frag=135V Gain=1,0

RT [min]	Peak MS Base Peak m/z	Area	Area%	Max Peak%	Height
8.341	1115.800	11812737.6105	100.0000	100.000	1510594.215
	Sum	11812737.6105			

MS1 +TIC SCAN ESI Frag=135V Gain=1,0RT:8.33100889653904min (Subtracted)

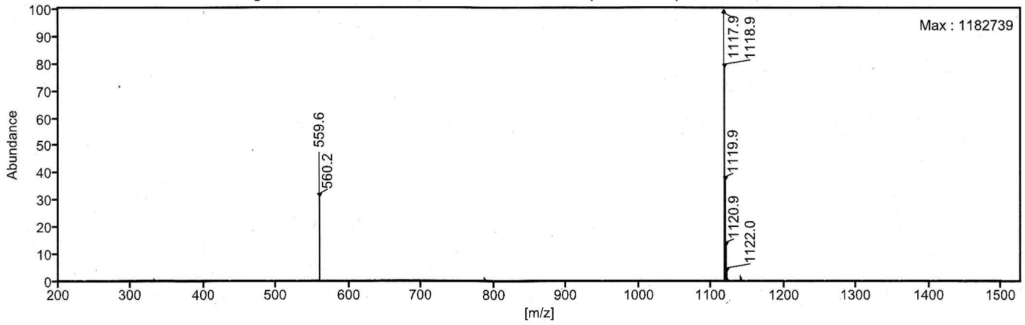

MS1 -TIC SCAN ESI Frag=135V Gain=1,0RT:8.34072823098182min (Subtracted)

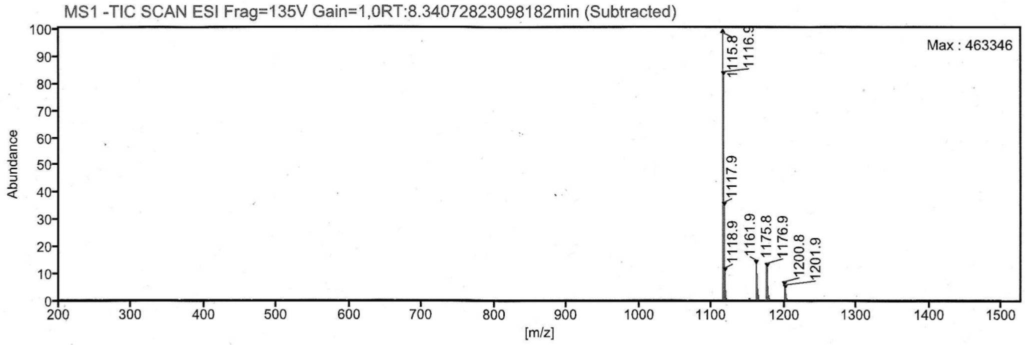

LC/MS analysis of PROTAC **10c** (CST1120)

**Sequence Name:**	SingleSample		
**Data file:**	CST1120_ACN.dx	**Project Name:**	Single Quad
**Sample name:**	CST1120_ACN	**Operator :**	SYSTEM
**Instrument:**	Single Quad	**Acquired on:**	2023-01-19 08:36:13+01:00
**Inj. volume:**	2.000	**Location:**	P1-E1
**Acq. method:**	00-new_wasser-acn_standard_100-1500_BD-20min.amx		
**Processing method:**	MS_standard_plot.pmx		

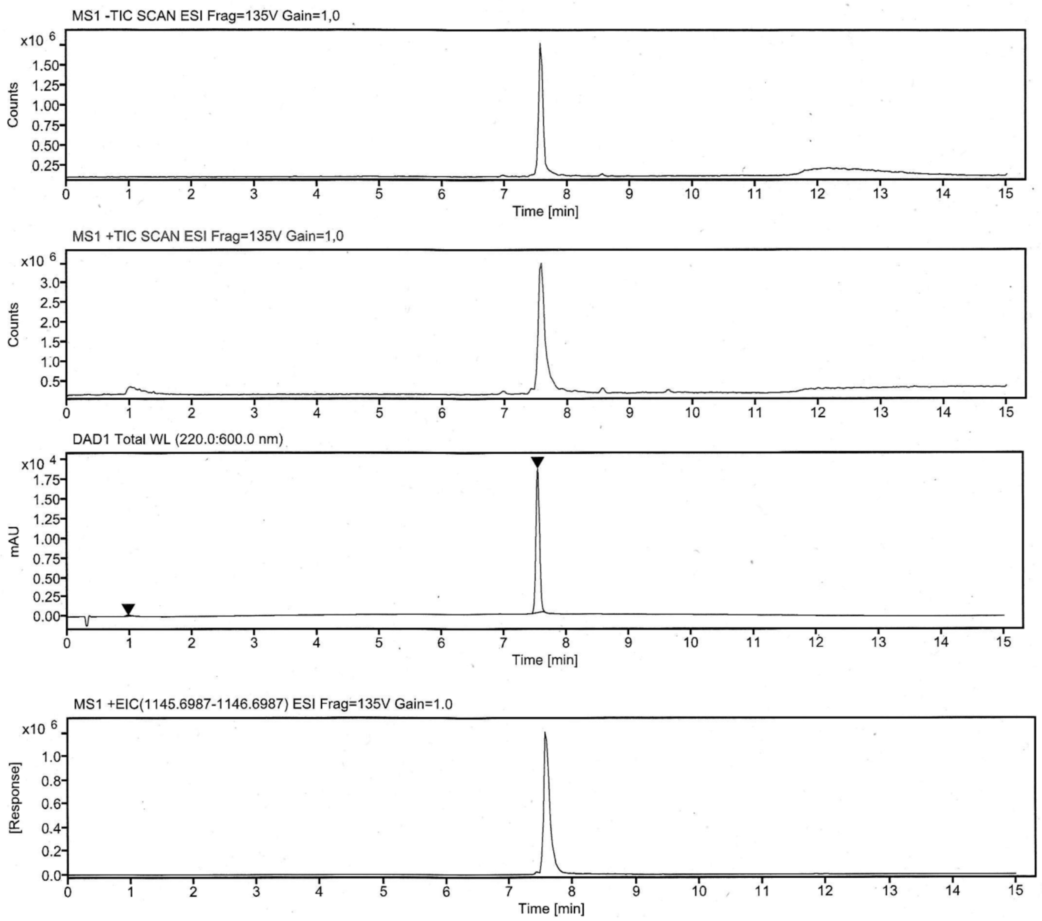

Signal: DAD1 Total WL (220.0:600.0 nm)

RT [min]	Peak MS Base Peak m/z	Area	Area%	Max Peak%	Height
0.983		622.2246	0.8216	0.828	134.586
7.545		75111.2251	99.1784	100.000	18485.118
	Sum	75733.4497			

Signal: MS1 +TIC SCAN ESI Frag=135V Gain=1,0

RT [min]	Peak MS Base Peak m/z	Area	Area%	Max Peak%	Height
7.575	1146.000	26192611.1758	100.0000	100.000	3377708.113
	Sum	26192611.1758			

Signal: MS1 -TIC SCAN ESI Frag=135V Gain=1,0

RT [min]	Peak MS Base Peak m/z	Area	Area%	Max Peak%	Height
7.578	1143.800	8769004.5807	100.0000	100.000	1707764.534
	Sum	8769004.5807			

MS1 +TIC SCAN ESI Frag=135V Gain=1,0RT:7.57535130902509min (Subtracted)

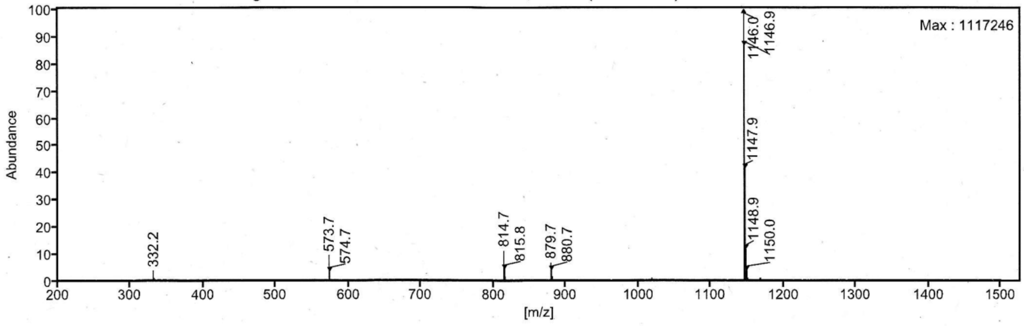

MS1 -TIC SCAN ESI Frag=135V Gain=1,0RT:7.57766932121731min (Subtracted)

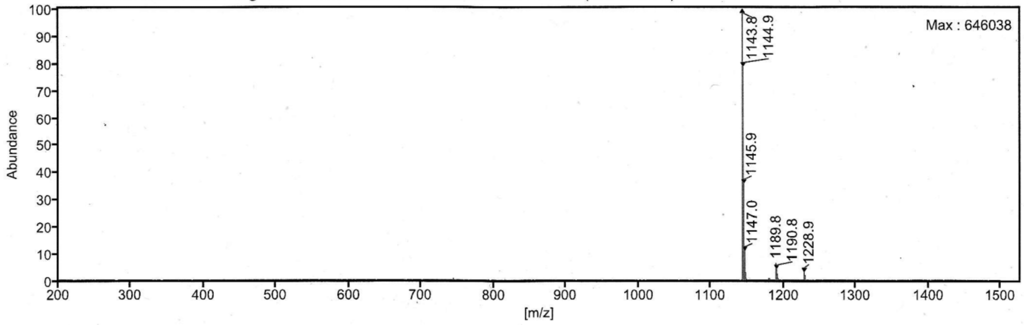

LC/MS analysis of PROTAC **10d** (CST621)

**Sequence Name:**	SingleSample		
**Data file:**	CST621_ACN.dx	**Project Name:**	Single Quad
**Sample name:**	CST621_ACN	**Operator :**	SYSTEM
**Instrument:**	Single Quad	**Acquired on:**	2022-12-12 12:36:45+01:00
**Inj. volume:**	2.000	**Location:**	P1-A1
**Acq. method:**	00-new_wasser-acn_standard_100-1500_BD-20min.amx		
**Processing method:**	MS_standard_plot.pmx		

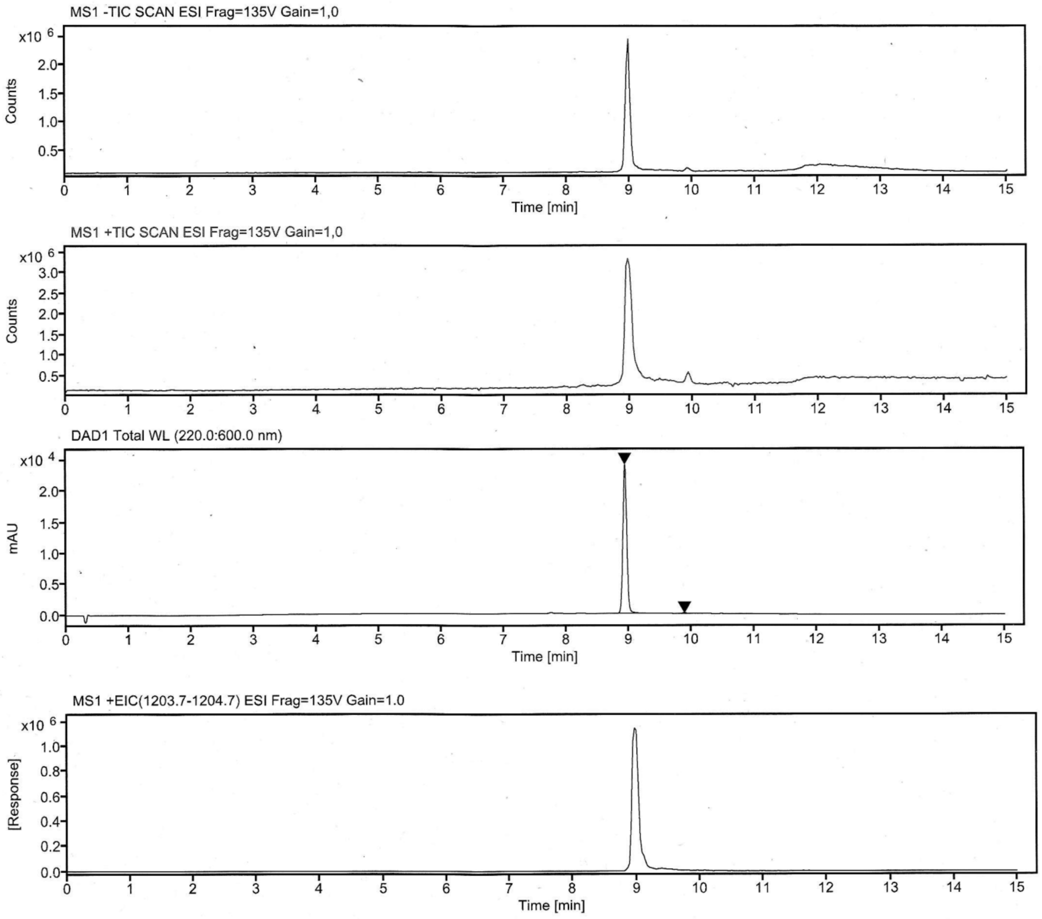

Signal: DAD1 Total WL (220.0:600.0 nm)

RT [min]	Peak MS Base Peak m/z	Area	Area%	Max Peak%	Height
8.940	1204.000	103773.2806	99.5165	100.000	23999.756
9.891		504.2221	0.4835	0.486	118.132
	Sum	104277.5027			

Signal: MS1 +TIC SCAN ESI Frag=135V Gain=1,0

RT [min]	Peak MS Base Peak m/z	Area	Area%	Max Peak%	Height
8.970	1204.000	28802011.0161	100.0000	100.000	3077470.621
	Sum	28802011.0161			

Signal: MS1 -TIC SCAN ESI Frag=135V Gain=1,0

RT [min]	Peak MS Base Peak m/z	Area	Area%	Max Peak%	Height
8.979	1202.000	14320119.8532	100.0000	100.000	2368266.109
	Sum	14320119.8532			

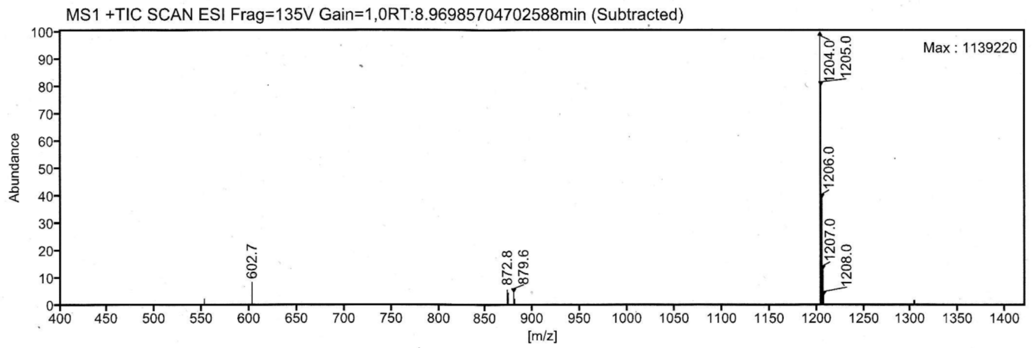

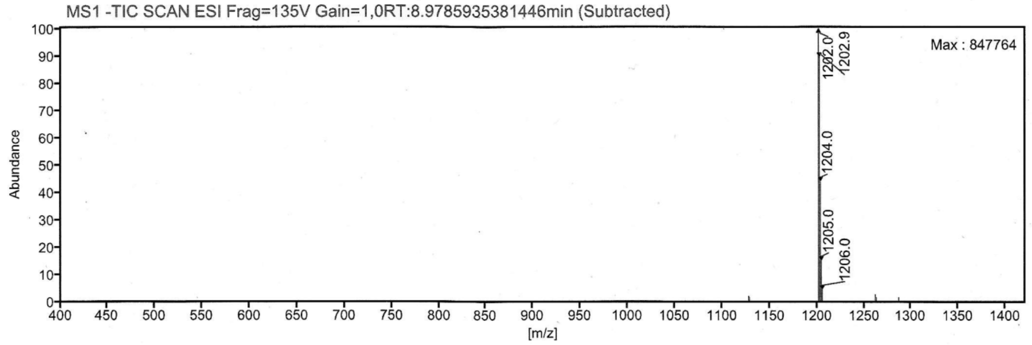

HPLC analysis of PROTAC **11** (SAB364)

Chromatogram and Results

Injection Details

Injection Name:	**SAB 364**	*Run Time (min):*	**14,50**
Vial Number:	**BA2**	*Injection Volume:*	**5,00**
Injection Type:	**Unknown**	*Channel:*	**UV_VIS_3**
Calibration Level:		*Wavelength:*	**254**
Instrument Method:	**Purity_PROTAC**	*Bandwidth:*	**16**
Processing Method:	**Quantitative**	*Dilution Factor:*	**1,0000**
Injection Date/Time:	**17.dec.**	*Sample Weight:*	**1,0000**

Chromatogram

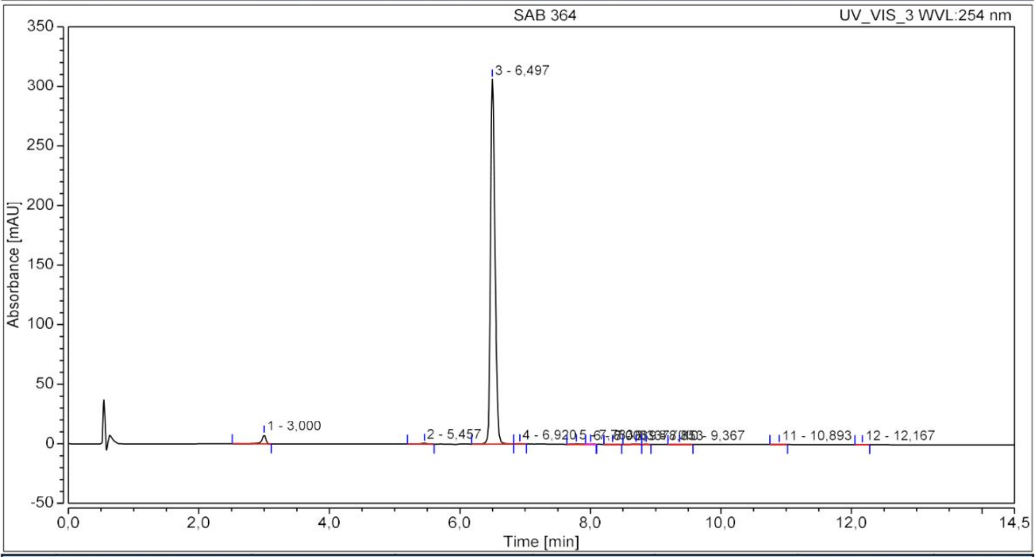

Integration Results

No.	Peak Name	Retention Time min	Area mAU*min	Height mAU	Relative Area %	Relative Height %	Amount n.a.
1		3,000	0,573	7,584	2,28	2,40	n.a.
2		5,457	0,082	0,745	0,33	0,24	n.a.
3		6,497	24,276	306,317	96,83	96,96	n.a.
4		6,920	0,034	0,318	0,13	0,10	n.a.
5		7,783	0,041	0,397	0,16	0,13	n.a.
6		8,003	0,005	0,054	0,02	0,02	n.a.
7		8,337	0,006	0,042	0,03	0,01	n.a.
8		8,700	0,018	0,169	0,07	0,05	n.a.
9		8,853	0,007	0,084	0,03	0,03	n.a.
10		9,367	0,013	0,074	0,05	0,02	n.a.
11		10,893	0,007	0,070	0,03	0,02	n.a.
12		12,167	0,007	0,064	0,03	0,02	n.a.
**Total:**			**25,070**	**315,918**	**100,00**	**100,00**	

Chromeleon (c) Dionex
Version 7.2.9.11323

LC/MS analysis of PROTAC **12** (CST613)

**Sequence Name:**	SingleSample		
**Data file:**	Gü3430_ACN.dx	**Project Name:**	Single Quad
**Sample name:**	Gü3430_ACN	**Operator :**	SYSTEM
**Instrument:**	Single Quad	**Acquired on:**	2022-04-19 07:04:44+02:00
**Inj. volume:**	3.000	**Location:**	P1-B6
**Acq. method:**	00-new_wasser-acn_standard_100-1500_BD-20min.amx		
**Processing method:**	MS_standard_plot.pmx		

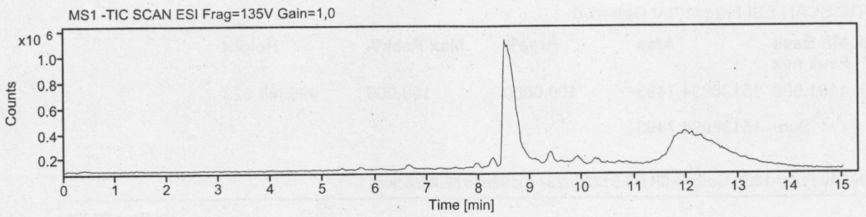

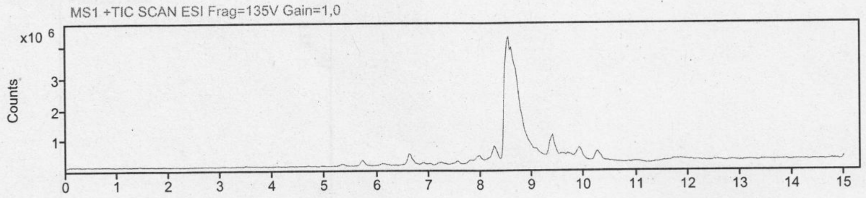

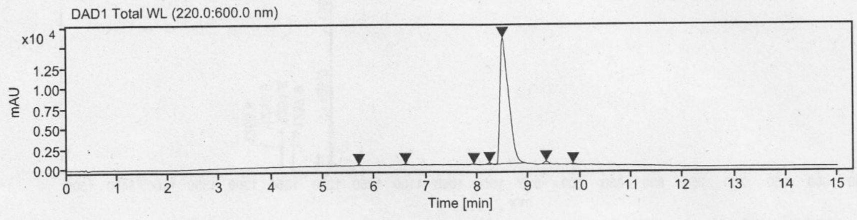

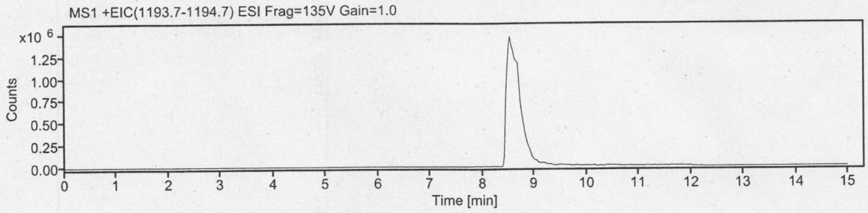

Signal: DAD1 Total WL (220.0:600.0 nm)

RT [min]	Peak MS Base Peak m/z	Area	Area%	Max Peak%	Height
5.692		277.1573	0.1467	0.150	65.290
6.598		387.7689	0.2053	0.210	88.885
7.928		319.4121	0.1691	0.173	68.928
8.234		789.6141	0.4180	0.428	179.384
8.498	1191.900	184588.3447	97.7231	100.000	15543.327
9.343		1878.6749	0.9946	1.018	313.898
9.867		648.2273	0.3432	0.351	106.484

D:\CDSProjects\Default\0-standardplot+EIC.rdl [Rev. 6480] Printed: 2022-04-19 10:24:39+02:00 Page 1 of 2

RT [min]	Peak MS Base Peak m/z	Area	Area%	Max Peak%	Height
	Sum	188889.1992			

Signal: MS1 +TIC SCAN ESI Frag=135V Gain=1,0

RT [min]	Peak MS Base Peak m/z	Area	Area%	Max Peak%	Height
8.538	1194.000	66170319.9437	100.0000	100.000	3852717.194
	Sum	66170319.9437			

Signal: MS1 -TIC SCAN ESI Frag=135V Gain=1,0

RT [min]	Peak MS Base Peak m/z	Area	Area%	Max Peak%	Height
8.512	1191.900	15138994.7493	100.0000	100.000	996385.623
	Sum	15138994.7493			

MS1 -TIC SCAN ESI Frag=135V Gain=1,0RT:8.51236623470193min (Subtracted)

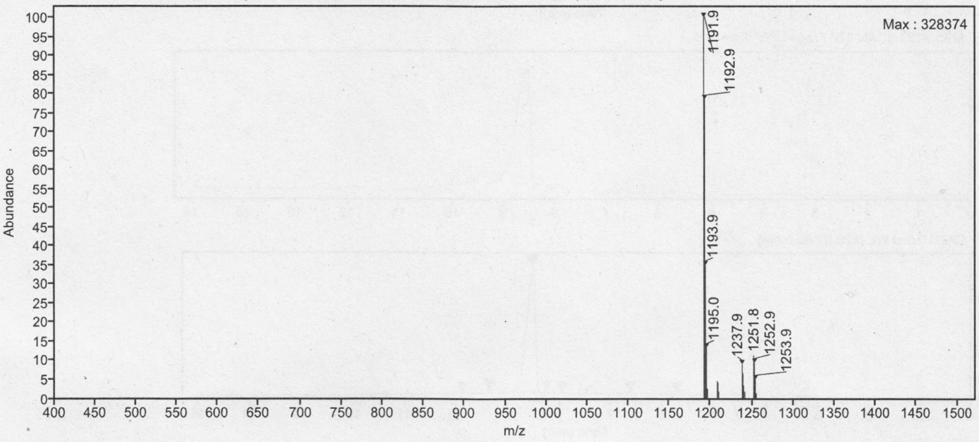

MS1 +TIC SCAN ESI Frag=135V Gain=1,0RT:8.53788004809012min (Subtracted)

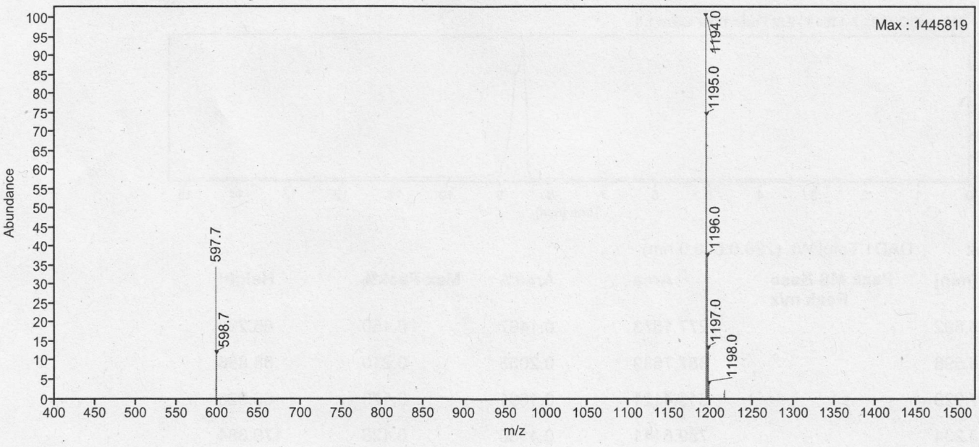

LC/MS analysis of PROTAC **13** (CST591)

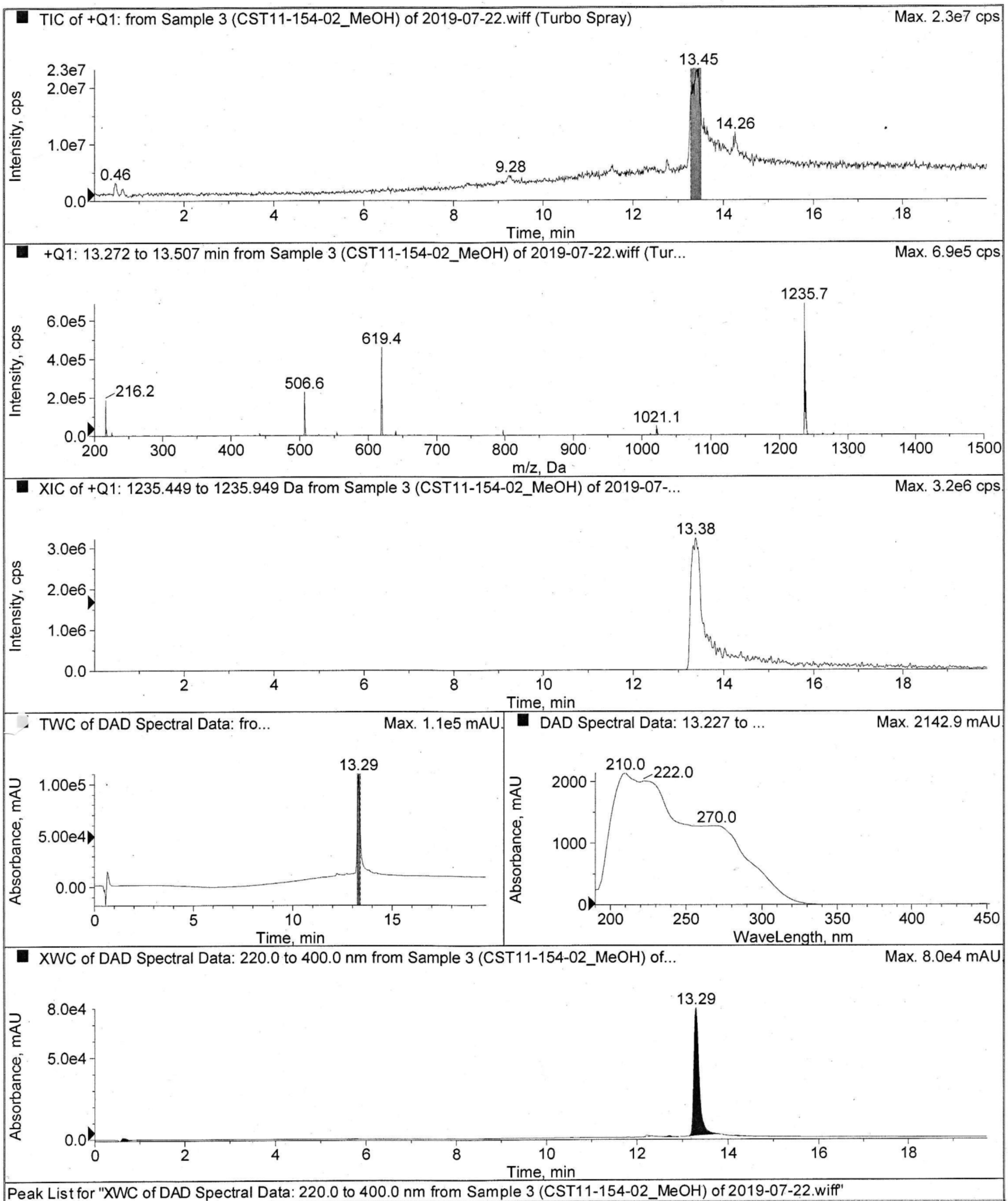

Peak List for "XWC of DAD Spectral Data: 220.0 to 400.0 nm from Sample 3 (CST11-154-02_MeOH) of 2019-07-22.wiff"

	Time (min)	Area (mAU x min)	% Area	Height (mAU)	% Height	Width (min)	Baseline Type
1	0.6339	1.4150e4	2.0286	1850.8984	2.2315	0.2933	Base to Base
2	12.2180	5382.7846	0.7717	1125.1107	1.3565	0.1800	Base to Base
3	12.7080	2981.4822	0.4274	635.3017	0.7659	0.2467	Base to Base
4	12.9660	541.4606	0.0776	102.8939	0.1241	0.1733	Base to Base
5	13.2937	6.7110e5	96.2089	7.8637e4	94.8059	0.6333	Base to Base
6	13.8194	1362.1867	0.1953	273.9060	0.3302	0.1467	Base to Base
7	14.1938	2026.5825	0.2905	320.1095	0.3859	0.3600	Base to Base

LC/MS analysis of PROTAC **14** (CST612)

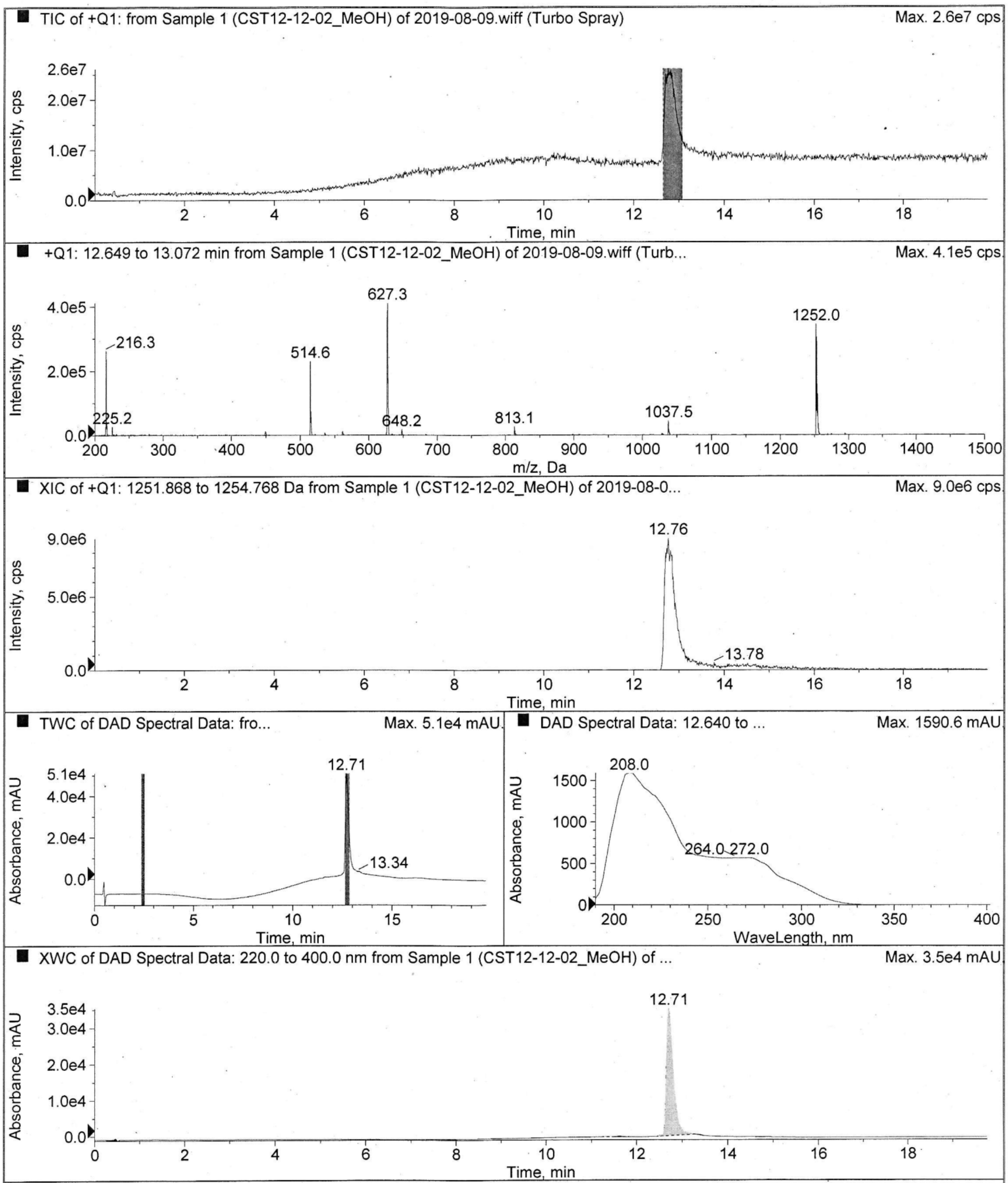

Peak List for "XWC of DAD Spectral Data: 220.0 to 400.0 nm from Sample 1 (CST12-12-02_MeOH) of 2019-08-09.wiff"

	Time (min)	Area (mAU x min)	% Area	Height (mAU)	% Height	Width (min)	Baseline Type
1	0.4620	3215.9796	0.8317	564.9591	1.5511	0.2800	Base to Base
2	11.3634	619.5585	0.1602	105.4863	0.2896	0.2533	Base to Base
3	11.6116	1571.4173	0.4064	234.3598	0.6434	0.3667	Base to Base
4	12.7058	3.8044e5	98.3827	3.5408e4	97.2105	0.8667	Base to Base
5	13.3277	846.9235	0.2190	111.2500	0.3054	0.2067	Base to Base

LC/MS analysis of PROTAC **15** (CST594)

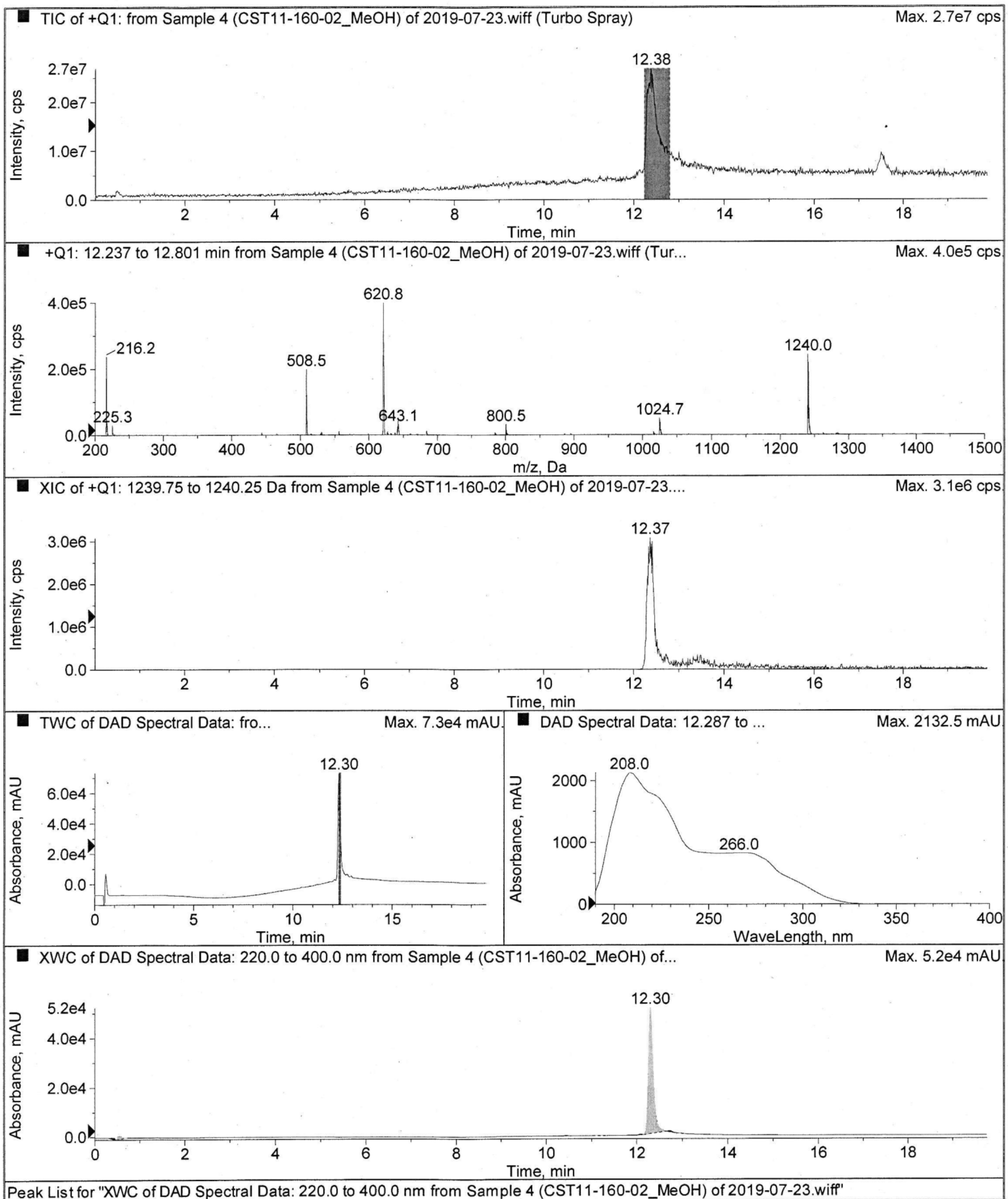

Peak List for "XWC of DAD Spectral Data: 220.0 to 400.0 nm from Sample 4 (CST11-160-02_MeOH) of 2019-07-23.wiff"

	Time (min)	Area (mAU x min)	% Area	Height (mAU)	% Height	Width (min)	Baseline Type
1	0.4238	3579.4011	0.8819	710.1985	1.2934	0.1600	Base to Base
2	0.5463	1.0043e4	2.4742	1459.1964	2.6574	0.2267	Base to Base
3	10.4348	1311.6453	0.3232	208.1422	0.3791	0.4400	Base to Base
4	11.1926	951.6867	0.2345	169.5663	0.3088	0.3000	Base to Base
5	12.0914	1259.1985	0.3102	291.2733	0.5304	0.1600	Base to Base
6	12.2990	3.8476e5	94.7942	5.1328e4	93.4748	0.5067	Base to Base
7	12.7264	2384.9772	0.5876	432.6917	0.7880	0.1800	Base to Base
8	12.9303	1599.9923	0.3942	311.9666	0.5681	0.1867	Base to Base

LC/MS analysis of PROTAC **16** (CST595)

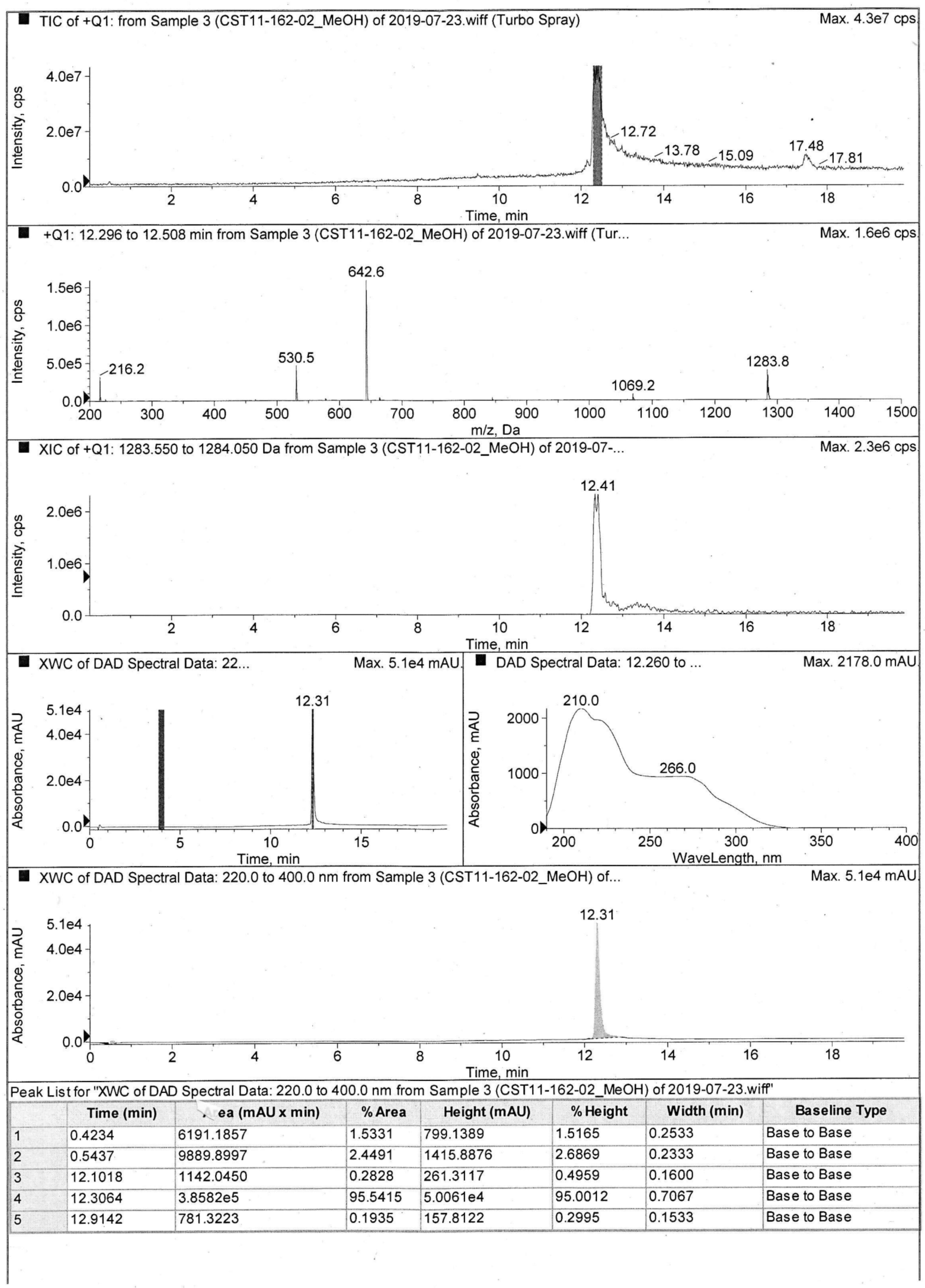

Peak List for "XWC of DAD Spectral Data: 220.0 to 400.0 nm from Sample 3 (CST11-162-02_MeOH) of 2019-07-23.wiff"

	Time (min)	[illegible]ea (mAU x min)	% Area	Height (mAU)	% Height	Width (min)	Baseline Type
1	0.4234	6191.1857	1.5331	799.1389	1.5165	0.2533	Base to Base
2	0.5437	9889.8997	2.4491	1415.8876	2.6869	0.2333	Base to Base
3	12.1018	1142.0450	0.2828	261.3117	0.4959	0.1600	Base to Base
4	12.3064	3.8582e5	95.5415	5.0061e4	95.0012	0.7067	Base to Base
5	12.9142	781.3223	0.1935	157.8122	0.2995	0.1533	Base to Base

LC/MS analysis of PROTAC **17** (CST632)

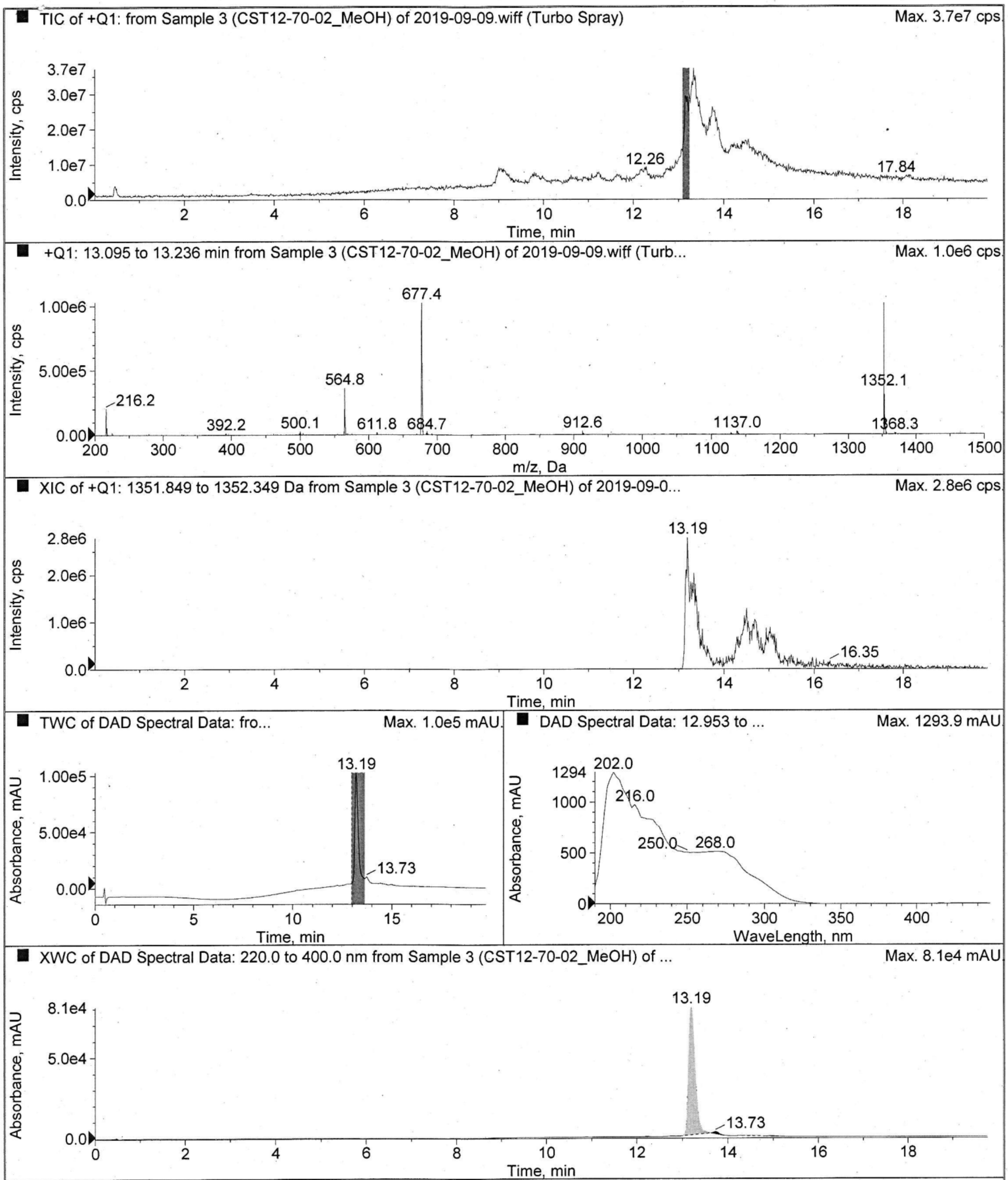

Peak List for "XWC of DAD Spectral Data: 220.0 to 400.0 nm from Sample 3 (CST12-70-02_MeOH) of 2019-09-09.wiff"

	Time (min)	Area (mAU x min)	% Area	Height (mAU)	% Height	Width (min)	Baseline Type
1	0.4690	2811.7715	0.3354	657.1332	0.7961	0.1533	Base to Base
2	0.7339	1885.5963	0.2250	25.7022	0.0311	0.2133	Base to Base
3	12.2279	2382.0481	0.2842	267.4435	0.3240	0.3533	Base to Base
4	13.1913	8.1474e5	97.2000	7.9759e4	96.6240	0.6733	Base to Base
5	13.7291	1.0600e4	1.2646	1367.7122	1.6569	0.2867	Base to Base
6	14.4407	3552.7697	0.4239	243.8383	0.2954	0.5067	Base to Base
7	14.9615	2238.1314	0.2670	224.9354	0.2725	0.4600	Base to Base

LC/MS analysis of PROTAC **18** (CST627)

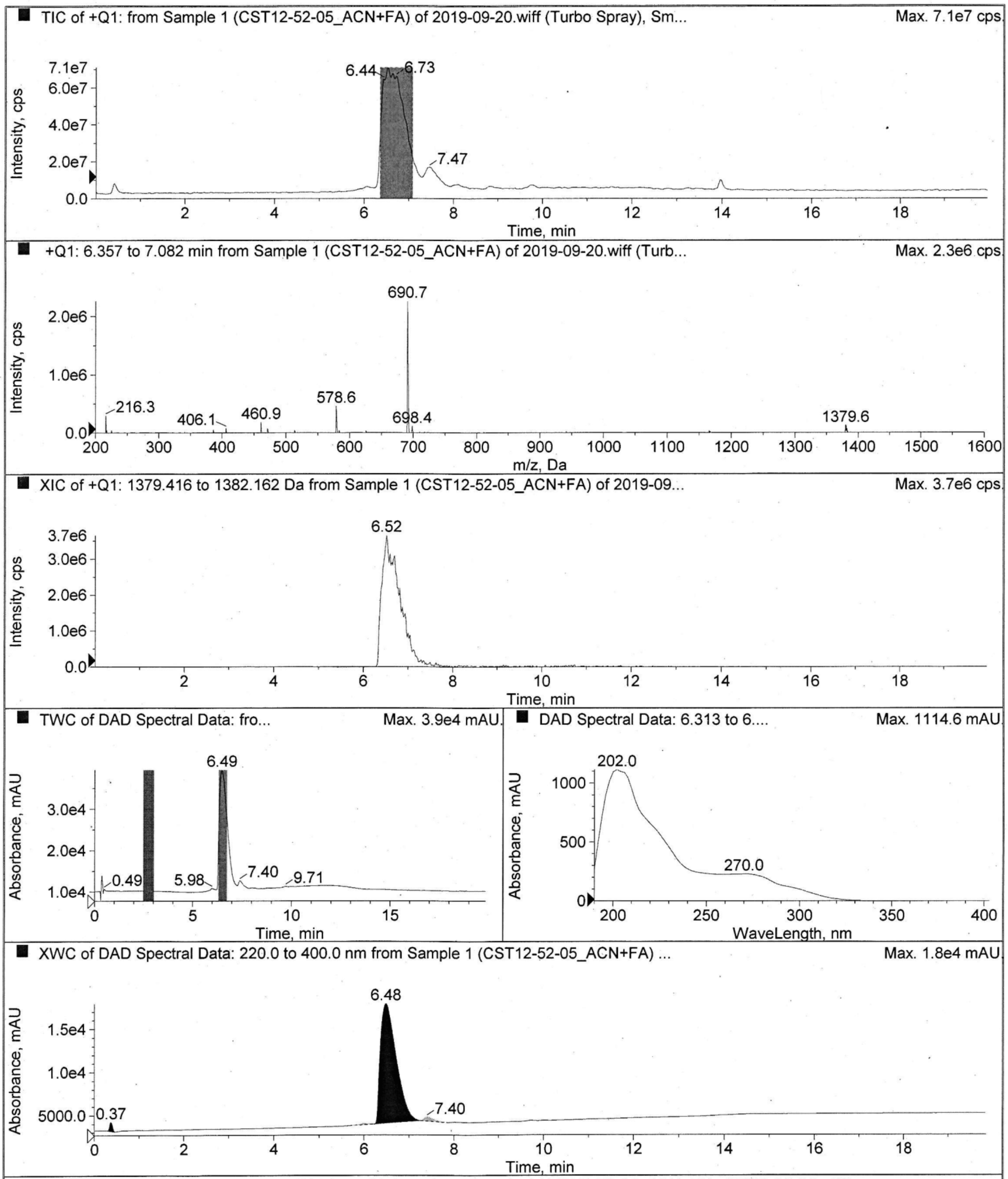

Peak List for "XWC of DAD Spectral Data: 220.0 to 400.0 nm from Sample 1 (CST12-52-05_ACN+FA) of 2019-09-20.wiff"

	Time (min)	Area (mAU x min)	% Area	Height (mAU)	% Height	Width (min)	Baseline Type
1	0.3691	4766.8231	1.3308	1055.6485	6.7559	0.1533	Base to Base
2	5.9885	2013.9931	0.5623	169.0645	1.0820	0.5267	Base to Base
3	6.4839	3.4465e5	96.2216	1.3912e4	89.0364	1.0333	Base to Base
4	7.4035	6752.6210	1.8853	488.4094	3.1257	0.5667	Base to Base

LC/MS analysis of PROTAC **19** (CST633)

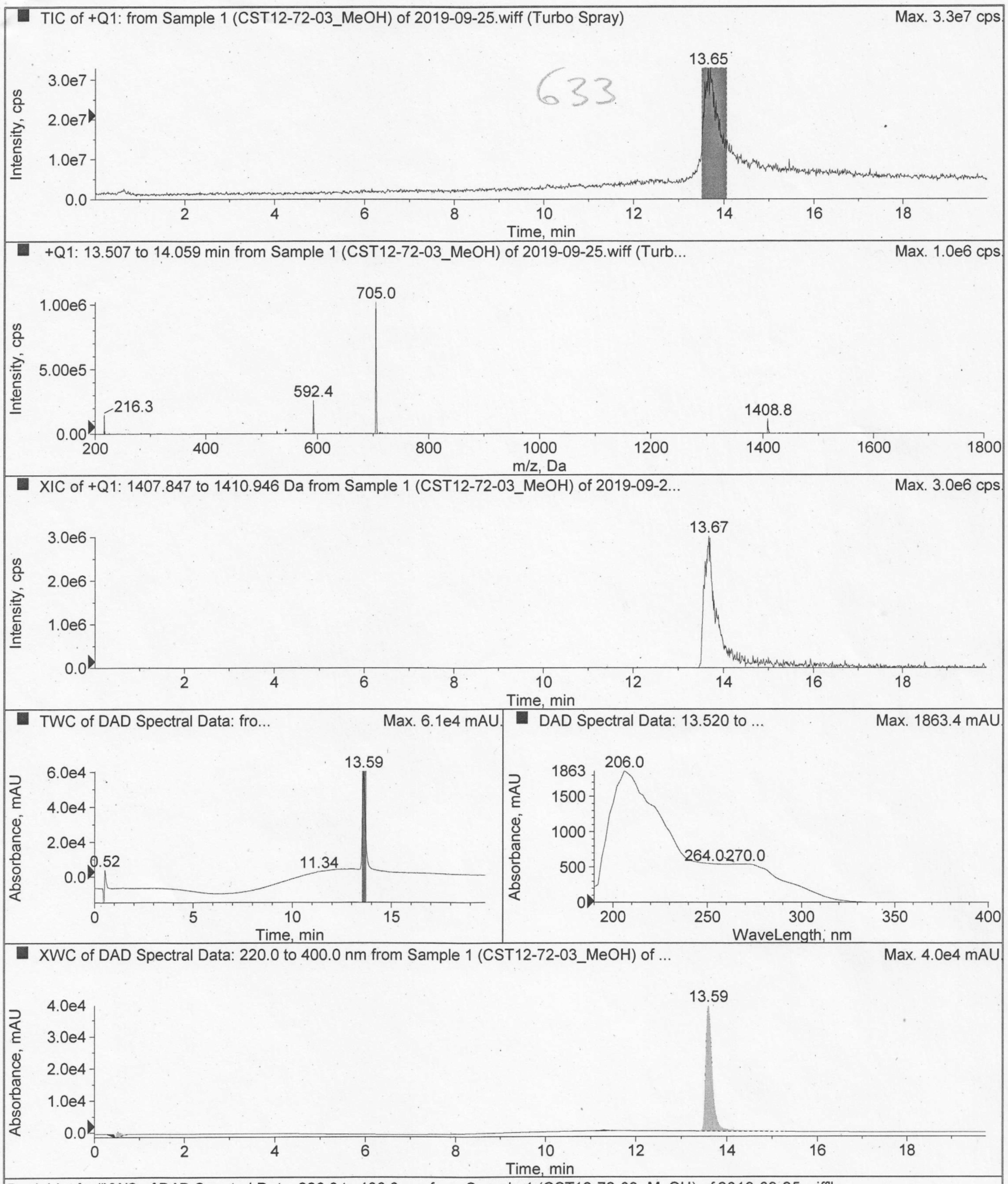

Peak List for "XWC of DAD Spectral Data: 220.0 to 400.0 nm from Sample 1 (CST12-72-03_MeOH) of 2019-09-25.wiff"

	Time (min)	Area (mAU x min)	% Area	Height (mAU)	% Height	Width (min)	Baseline Type
1	0.4096	4781.2794	1.1595	768.6576	1.8281	0.1867	Base to Base
2	0.5176	1.0711e4	2.5974	1523.7079	3.6239	0.2733	Base to Base
3	11.2905	1964.2199	0.4763	224.5519	0.5341	0.4467	Base to Base
4	13.5903	3.9491e5	95.7668	3.9529e4	94.0139	1.9400	Base to Base

HPLC analysis of PROTAC **20** (SAB116)

Chromatogram and Results

Injection Details

Injection Name:	**SAB 116**	*Run Time (min):*	**14,50**
Vial Number:	**RA6**	*Injection Volume:*	**2,00**
Injection Type:	**Unknown**	*Channel:*	**UV_VIS_3**
Calibration Level:		*Wavelength:*	**254**
Instrument Method:	**Purity_PROTAC**	*Bandwidth:*	**16**
Processing Method:	**Quantitative**	*Dilution Factor:*	**1,0000**
Injection Date/Time:	**28.okt.**	*Sample Weight:*	**1,0000**

Chromatogram

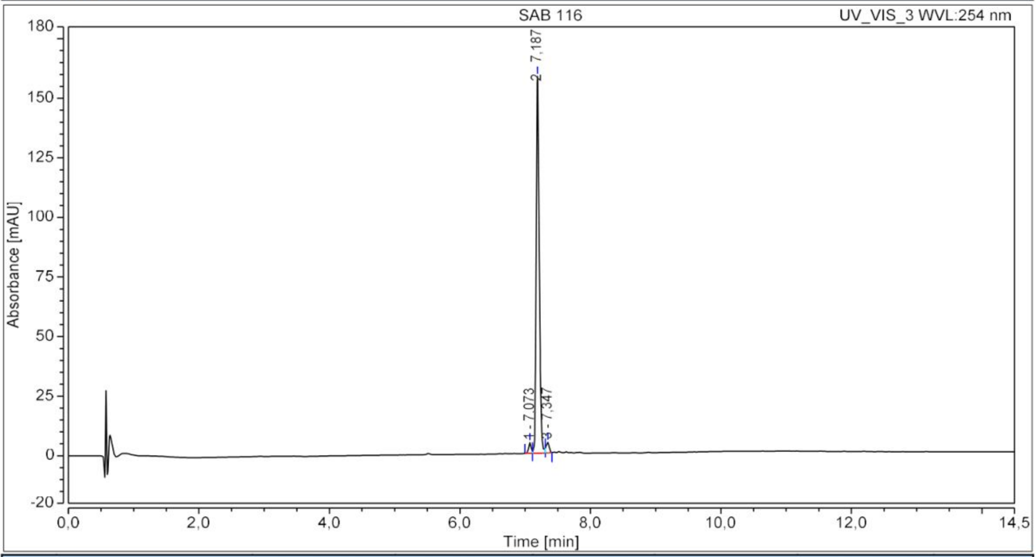

Integration Results

No.	Peak Name	Retention Time min	Area mAU*min	Height mAU	Relative Area %	Relative Height %	Amount n.a.
1		7,073	0,201	4,566	2,24	2,73	n.a.
2		7,187	8,532	158,227	95,08	94,59	n.a.
3		7,347	0,240	4,490	2,68	2,68	n.a.
**Total:**			**8,973**	**167,284**	**100,00**	**100,00**	

Chromeleon (c) Dionex
Version 7.2.9.11323

HPLC analysis of PROTAC **21** (SAB120)

Chromatogram and Results

Injection Details

Injection Name:	**SAB 120**	*Run Time (min):*	**14,50**
Vial Number:	**RA7**	*Injection Volume:*	**2,00**
Injection Type:	**Unknown**	*Channel:*	**UV_VIS_3**
Calibration Level:		*Wavelength:*	**254**
Instrument Method:	**Purity_PROTAC**	*Bandwidth:*	**16**
Processing Method:	**Quantitative**	*Dilution Factor:*	**1,0000**
Injection Date/Time:	**28.okt.**	*Sample Weight:*	**1,0000**

Chromatogram

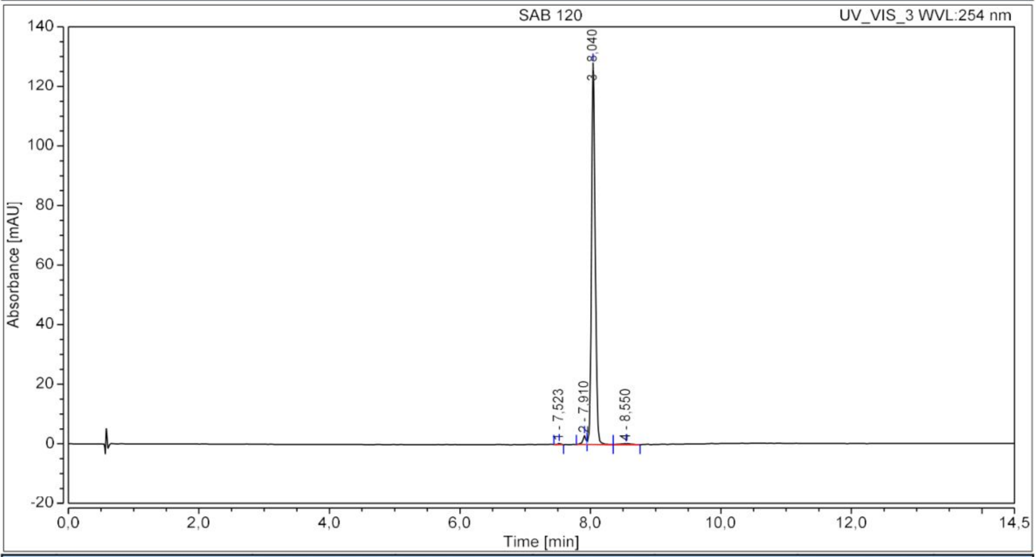

Integration Results

No.	Peak Name	Retention Time min	Area mAU*min	Height mAU	Relative Area %	Relative Height %	Amount n.a.
1		7,523	0,023	0,418	0,27	0,32	n.a.
2		7,910	0,170	3,026	1,98	2,29	n.a.
3		8,040	8,276	128,136	96,89	97,12	n.a.
4		8,550	0,073	0,357	0,86	0,27	n.a.
**Total:**			**8,542**	**131,937**	**100,00**	**100,00**	

Chromeleon (c) Dionex
Version 7.2.9.11323

HPLC analysis of PROTAC **22** (SAB131)

Chromatogram and Results

Injection Details

Injection Name:	**SAB 131**	*Run Time (min):*	**14,50**
Vial Number:	**RB2**	*Injection Volume:*	**2,00**
Injection Type:	**Unknown**	*Channel:*	**UV_VIS_3**
Calibration Level:		*Wavelength:*	**254**
Instrument Method:	**Purity_PROTAC**	*Bandwidth:*	**16**
Processing Method:	**Quantitative**	*Dilution Factor:*	**1,0000**
Injection Date/Time:	**28.okt.**	*Sample Weight:*	**1,0000**

Chromatogram

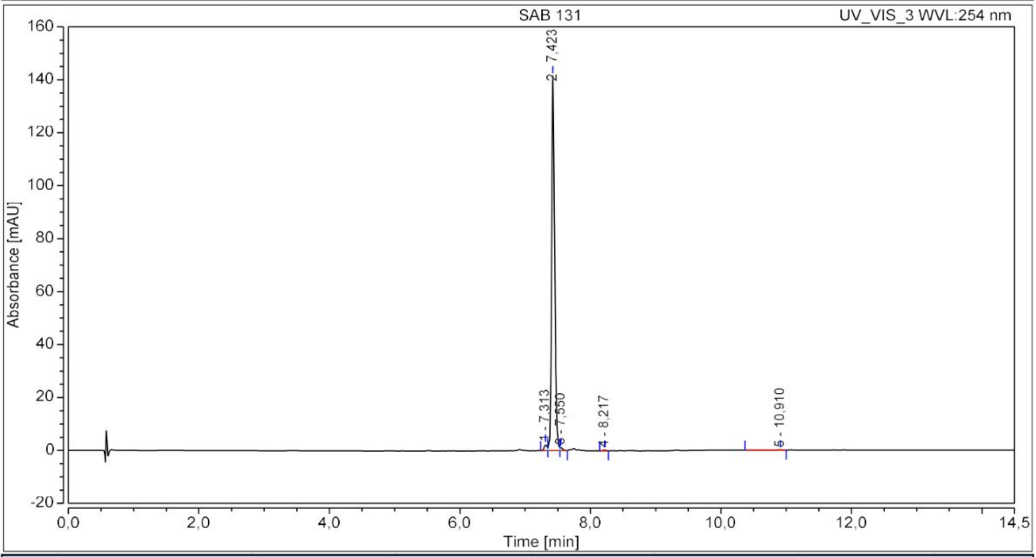

Integration Results

No.	Peak Name	Retention Time min	Area mAU*min	Height mAU	Relative Area %	Relative Height %	Amount n.a.
1		7,313	0,118	2,463	1,50	1,69	n.a.
2		7,423	7,664	141,635	97,31	97,24	n.a.
3		7,550	0,051	1,070	0,65	0,73	n.a.
4		8,217	0,020	0,421	0,25	0,29	n.a.
5		10,910	0,022	0,065	0,28	0,04	n.a.
**Total:**			**7,875**	**145,655**	**100,00**	**100,00**	

Chromeleon (c) Dionex
Version 7.2.9.11323

HPLC analysis of PROTAC **23** (SAB130)

Chromatogram and Results

Injection Details

Injection Name:	**SAB 130**	*Run Time (min):*	**14,50**
Vial Number:	**RB1**	*Injection Volume:*	**2,00**
Injection Type:	**Unknown**	*Channel:*	**UV_VIS_3**
Calibration Level:		*Wavelength:*	**254**
Instrument Method:	**Purity_PROTAC**	*Bandwidth:*	**16**
Processing Method:	**Quantitative**	*Dilution Factor:*	**1,0000**
Injection Date/Time:	**28.okt.**	*Sample Weight:*	**1,0000**

Chromatogram

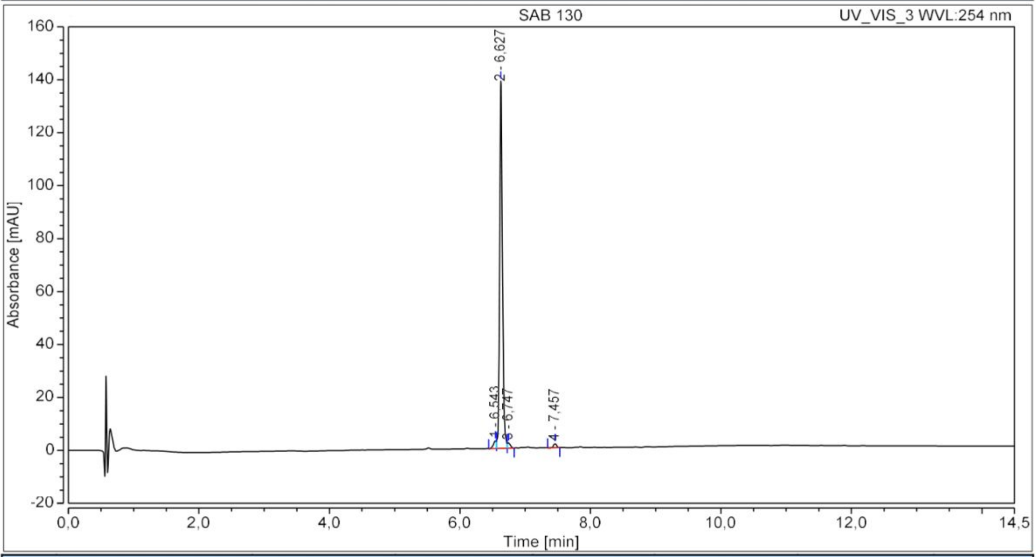

Integration Results

No.	Peak Name	Retention Time min	Area mAU*min	Height mAU	Relative Area %	Relative Height %	Amount n.a.
1		6,543	0,155	2,964	2,15	2,04	n.a.
2		6,627	6,877	138,741	95,30	95,42	n.a.
3		6,747	0,099	2,058	1,38	1,42	n.a.
4		7,457	0,085	1,633	1,17	1,12	n.a.
**Total:**			**7,216**	**145,396**	**100,00**	**100,00**	

Chromeleon (c) Dionex
Version 7.2.9.11323

HPLC analysis of PROTAC **24** (SAB122)

Chromatogram and Results

Injection Details

Injection Name:	**SAB 122**	*Run Time (min):*	**14,50**
Vial Number:	**RA8**	*Injection Volume:*	**2,00**
Injection Type:	**Unknown**	*Channel:*	**UV_VIS_3**
Calibration Level:		*Wavelength:*	**254**
Instrument Method:	**Purity_PROTAC**	*Bandwidth:*	**16**
Processing Method:	**Quantitative**	*Dilution Factor:*	**1,0000**
Injection Date/Time:	**28.okt.**	*Sample Weight:*	**1,0000**

Chromatogram

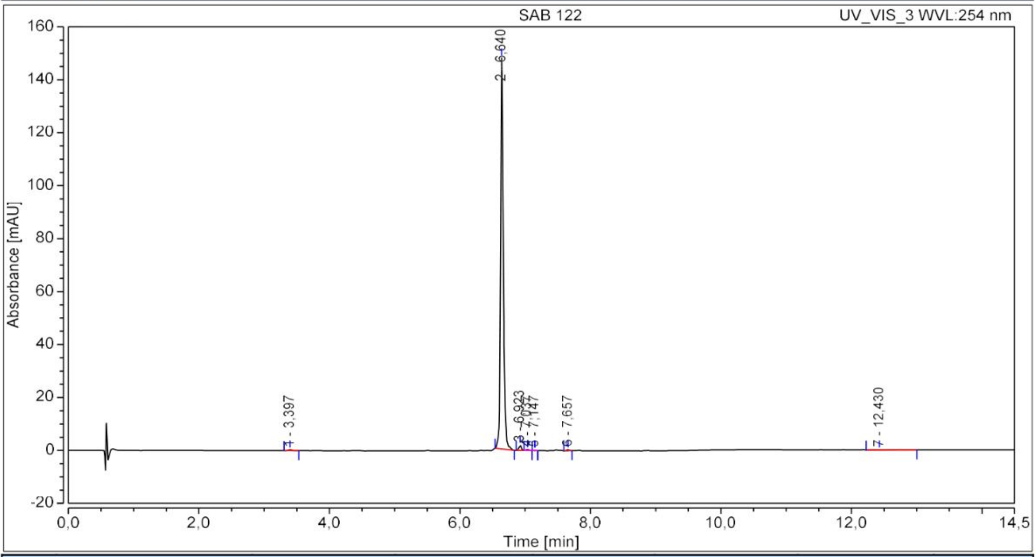

Integration Results

No.	Peak Name	Retention Time min	Area mAU*min	Height mAU	Relative Area %	Relative Height %	Amount n.a.
1		3,397	0,024	0,316	0,33	0,21	n.a.
2		6,640	7,281	147,689	97,50	97,77	n.a.
3		6,923	0,086	2,019	1,15	1,34	n.a.
4		7,037	0,020	0,331	0,27	0,22	n.a.
5		7,147	0,008	0,191	0,10	0,13	n.a.
6		7,657	0,021	0,445	0,29	0,29	n.a.
7		12,430	0,027	0,072	0,37	0,05	n.a.
**Total:**			**7,467**	**151,064**	**100,00**	**100,00**	

Chromeleon (c) Dionex
Version 7.2.9.11323

HPLC analysis of PROTAC **25** (SAB141)

Chromatogram and Results

Injection Details

Injection Name:	**SAB 141**	*Run Time (min):*	**14,50**
Vial Number:	**RA5**	*Injection Volume:*	**5,00**
Injection Type:	**Unknown**	*Channel:*	**UV_VIS_3**
Calibration Level:		*Wavelength:*	**254**
Instrument Method:	**Purity_PROTAC**	*Bandwidth:*	**16**
Processing Method:	**Quantitative**	*Dilution Factor:*	**1,0000**
Injection Date/Time:	**08.nov.**	*Sample Weight:*	**1,0000**

Chromatogram

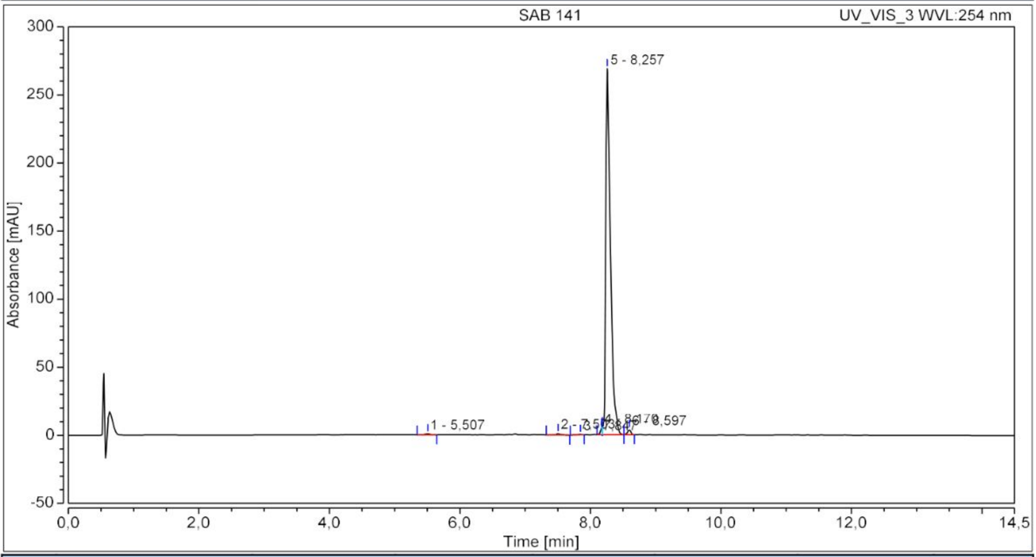

Integration Results

No.	Peak Name	Retention Time min	Area mAU*min	Height mAU	Relative Area %	Relative Height %	Amount n.a.
1		5,507	0,068	0,821	0,30	0,29	n.a.
2		7,503	0,087	1,059	0,38	0,38	n.a.
3		7,847	0,039	0,384	0,17	0,14	n.a.
4		8,170	0,160	5,044	0,69	1,80	n.a.
5		8,257	22,588	268,771	97,59	96,06	n.a.
6		8,597	0,204	3,726	0,88	1,33	n.a.
**Total:**			**23,146**	**279,806**	**100,00**	**100,00**	

Chromeleon (c) Dionex
Version 7.2.9.11323

HPLC analysis of PROTAC **26** (SAB153)

Chromatogram and Results

Injection Details

Injection Name:	**SAB 153**	*Run Time (min):*	**14,50**
Vial Number:	**RB7**	*Injection Volume:*	**5,00**
Injection Type:	**Unknown**	*Channel:*	**UV_VIS_3**
Calibration Level:		*Wavelength:*	**254**
Instrument Method:	**Purity_PROTAC**	*Bandwidth:*	**16**
Processing Method:	**Quantitative**	*Dilution Factor:*	**1,0000**
Injection Date/Time:	**11.nov.**	*Sample Weight:*	**1,0000**

Chromatogram

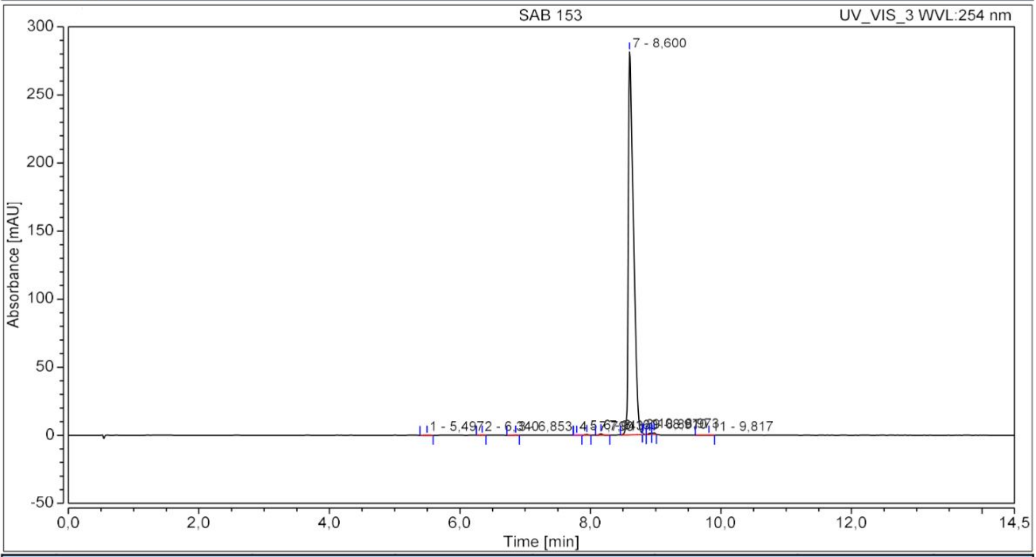

Integration Results

No.	Peak Name	Retention Time min	Area mAU*min	Height mAU	Relative Area %	Relative Height %	Amount n.a.
1		5,497	0,012	0,152	0,05	0,05	n.a.
2		6,340	0,011	0,191	0,04	0,07	n.a.
3		6,853	0,019	0,232	0,07	0,08	n.a.
4		7,790	0,004	0,064	0,02	0,02	n.a.
5		7,943	0,036	0,639	0,14	0,22	n.a.
6		8,160	0,055	1,104	0,21	0,38	n.a.
7		8,600	25,506	281,313	98,88	97,94	n.a.
8		8,807	0,027	0,968	0,10	0,34	n.a.
9		8,910	0,053	1,072	0,20	0,37	n.a.
10		8,973	0,062	1,388	0,24	0,48	n.a.
11		9,817	0,011	0,101	0,04	0,04	n.a.
**Total:**			**25,795**	**287,225**	**100,00**	**100,00**	

HPLC analysis of PROTAC **27** (SAB142)

Chromatogram and Results

Injection Details

Injection Name:	**SAB 142**	*Run Time (min):*	**14,50**
Vial Number:	**RA8**	*Injection Volume:*	**5,00**
Injection Type:	**Unknown**	*Channel:*	**UV_VIS_3**
Calibration Level:		*Wavelength:*	**254**
Instrument Method:	**Purity_PROTAC**	*Bandwidth:*	**16**
Processing Method:	**Quantitative**	*Dilution Factor:*	**1,0000**
Injection Date/Time:	**08.nov.**	*Sample Weight:*	**1,0000**

Chromatogram

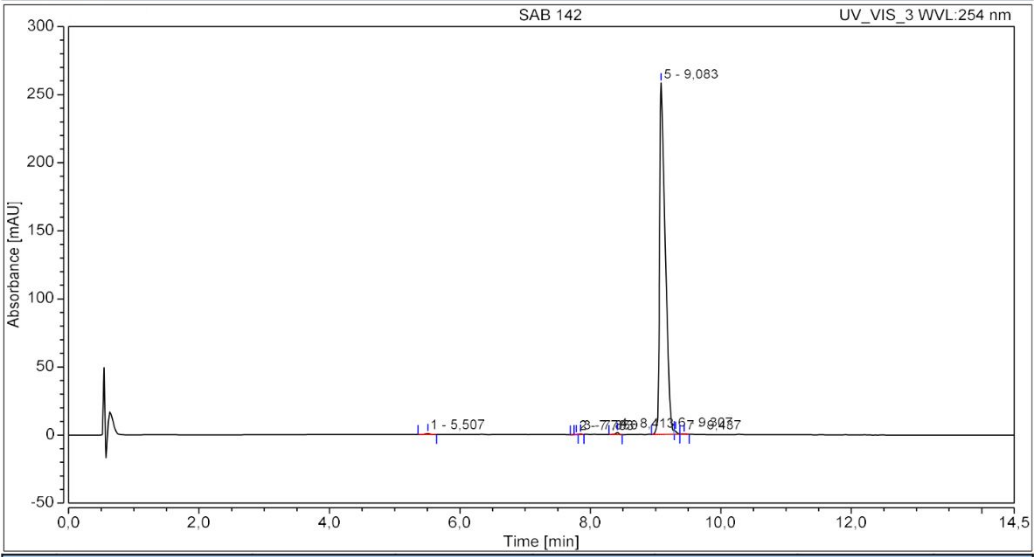

Integration Results

No.	Peak Name	Retention Time min	Area mAU*min	Height mAU	Relative Area %	Relative Height %	Amount n.a.
1		5,507	0,065	0,800	0,25	0,30	n.a.
2		7,783	0,001	0,043	0,01	0,02	n.a.
3		7,850	0,035	0,403	0,13	0,15	n.a.
4		8,413	0,097	1,680	0,37	0,64	n.a.
5		9,083	25,814	258,124	98,66	97,90	n.a.
6		9,307	0,121	2,312	0,46	0,88	n.a.
7		9,437	0,031	0,307	0,12	0,12	n.a.
**Total:**			**26,164**	**263,670**	**100,00**	**100,00**	

Chromeleon (c) Dionex
Version 7.2.9.11323

HPLC analysis of PROTAC **28** (SAB300)

Chromatogram and Results

Injection Details

Injection Name:	**SAB 300**	*Run Time (min):*	**14,50**
Vial Number:	**RA2**	*Injection Volume:*	**5,00**
Injection Type:	**Unknown**	*Channel:*	**UV_VIS_3**
Calibration Level:		*Wavelength:*	**254**
Instrument Method:	**Purity_PROTAC**	*Bandwidth:*	**16**
Processing Method:	**Quantitative**	*Dilution Factor:*	**1,0000**
Injection Date/Time:	**26.okt.**	*Sample Weight:*	**1,0000**

Chromatogram

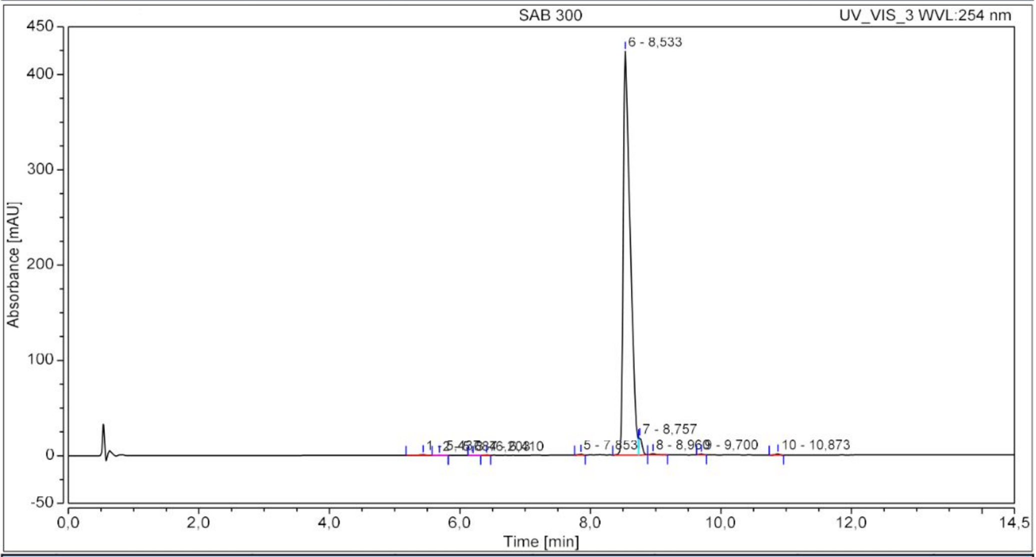

Integration Results

No.	Peak Name	Retention Time min	Area mAU*min	Height mAU	Relative Area %	Relative Height %	Amount n.a.
1		5,437	0,080	0,726	0,15	0,16	n.a.
2		5,687	0,015	0,132	0,03	0,03	n.a.
3		6,203	0,005	0,046	0,01	0,01	n.a.
4		6,410	0,023	0,349	0,04	0,08	n.a.
5		7,853	0,059	0,946	0,11	0,21	n.a.
6		8,533	52,315	423,611	97,05	94,70	n.a.
7		8,757	1,113	17,948	2,06	4,01	n.a.
8		8,960	0,153	1,354	0,28	0,30	n.a.
9		9,700	0,069	1,046	0,13	0,23	n.a.
10		10,873	0,076	1,142	0,14	0,26	n.a.
**Total:**			**53,906**	**447,297**	**100,00**	**100,00**	

Chromeleon (c) Dionex
Version 7.2.9.11323

HPLC analysis of PROTAC **29** (SAB302)

Chromatogram and Results

Injection Details

Injection Name:	**SAB 302**	*Run Time (min):*	**14,50**
Vial Number:	**RC6**	*Injection Volume:*	**5,00**
Injection Type:	**Unknown**	*Channel:*	**UV_VIS_3**
Calibration Level:		*Wavelength:*	**254**
Instrument Method:	**Purity_PROTAC**	*Bandwidth:*	**16**
Processing Method:	**Quantitative**	*Dilution Factor:*	**1,0000**
Injection Date/Time:	**16.jun.**	*Sample Weight:*	**1,0000**

Chromatogram

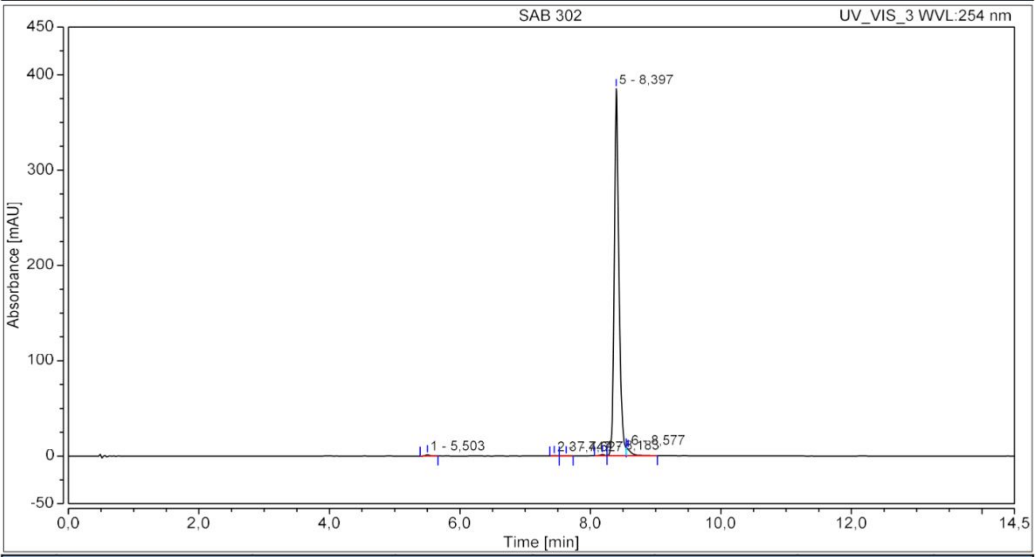

Integration Results

No.	Peak Name	Retention Time min	Area mAU*min	Height mAU	Relative Area %	Relative Height %	Amount n.a.
1		5,503	0,111	1,419	0,34	0,36	n.a.
2		7,447	0,037	0,616	0,11	0,16	n.a.
3		7,627	0,030	0,300	0,09	0,08	n.a.
4		8,183	0,103	1,318	0,31	0,33	n.a.
5		8,397	32,205	385,203	97,24	97,35	n.a.
6		8,577	0,632	6,816	1,91	1,72	n.a.
**Total:**			**33,119**	**395,672**	**100,00**	**100,00**	

Chromeleon (c) Dionex
Version 7.2.9.11323

References

(1) Gyrd-Hansen, M.; Meier, P. IAPs: From Caspase Inhibitors to Modulators of NF-KB, Inflammation and Cancer. *Nat Rev Cancer* **2010**, *10* (8), 561–574. https://doi.org/10.1038/nrc2889.

(2) Varfolomeev, E.; Goncharov, T.; Maecker, H.; Zobel, K.; Kömüves, L. G.; Deshayes, K.; Vucic, D. Cellular Inhibitors of Apoptosis Are Global Regulators of NF-KB and MAPK Activation by Members of the TNF Family of Receptors. *Sci Signal* **2012**, *5* (216), ra22. https://doi.org/10.1126/scisignal.2001878.

(3) Bertrand, M. J. M.; Milutinovic, S.; Dickson, K. M.; Ho, W. C.; Boudreault, A.; Durkin, J.; Gillard, J. W.; Jaquith, J. B.; Morris, S. J.; Barker, P. A. CIAP1 and CIAP2 Facilitate Cancer Cell Survival by Functioning as E3 Ligases That Promote RIP1 Ubiquitination. *Molecular Cell* **2008**, *30* (6), 689–700. https://doi.org/10.1016/j.molcel.2008.05.014.

(4) Fulda, S.; Vucic, D. Targeting IAP Proteins for Therapeutic Intervention in Cancer. *Nat Rev Drug Discov* **2012**, *11* (2), 109–124. https://doi.org/10.1038/nrd3627.

(5) Dueber, E. C.; Schoeffler, A. J.; Lingel, A.; Elliott, J. M.; Fedorova, A. V.; Giannetti, A. M.; Zobel, K.; Maurer, B.; Varfolomeev, E.; Wu, P.; Wallweber, H. J. A.; Hymowitz, S. G.; Deshayes, K.; Vucic, D.; Fairbrother, W. J. Antagonists Induce a Conformational Change in CIAP1 That Promotes Autoubiquitination. *Science* **2011**, *334* (6054), 376–380. https://doi.org/10.1126/science.1207862.

(6) Feltham, R.; Bettjeman, B.; Budhidarmo, R.; Mace, P. D.; Shirley, S.; Condon, S. M.; Chunduru, S. K.; McKinlay, M. A.; Vaux, D. L.; Silke, J.; Day, C. L. Smac Mimetics Activate the E3 Ligase Activity of CIAP1 Protein by Promoting RING Domain Dimerization. *J Biol Chem* **2011**, *286* (19), 17015–17028. https://doi.org/10.1074/jbc.M111.222919.

(7) Mitsuuchi, Y.; Benetatos, C. A.; Deng, Y.; Haimowitz, T.; Beck, S. C.; Arnone, M. R.; Kapoor, G. S.; Seipel, M. E.; Chunduru, S. K.; McKinlay, M. A.; Begley, C. G.; Condon, S. M. Bivalent IAP Antagonists, but Not Monovalent IAP Antagonists, Inhibit TNF-Mediated NF-KB Signaling by Degrading TRAF2-Associated CIAP1 in Cancer Cells. *Cell Death Discov.* **2017**, *3* (1), 1–10. https://doi.org/10.1038/cddiscovery.2016.46.

(8) Sun, H.; Nikolovska-Coleska, Z.; Lu, J.; Meagher, J. L.; Yang, C.-Y.; Qiu, S.; Tomita, Y.; Ueda, Y.; Jiang, S.; Krajewski, K.; Roller, P. P.; Stuckey, J. A.; Wang, S. Design, Synthesis, and Characterization of a Potent, Nonpeptide, Cell-Permeable, Bivalent Smac Mimetic That Concurrently Targets Both the BIR2 and BIR3 Domains in XIAP. *J Am Chem Soc* **2007**, *129* (49), 15279–15294. https://doi.org/10.1021/ja074725f.

(9) Lu, J.; Bai, L.; Sun, H.; Nikolovska-Coleska, Z.; McEachern, D.; Qiu, S.; Miller, R. S.; Yi, H.; Shangary, S.; Sun, Y.; Meagher, J. L.; Stuckey, J. A.; Wang, S. SM-164: A Novel, Bivalent Smac Mimetic That Induces Apoptosis and Tumor Regression by Concurrent Removal of the Blockade of CIAP-1/2 and XIAP. *Cancer Res* **2008**, *68* (22), 9384–9393. https://doi.org/10.1158/0008-5472.CAN-08-2655.

(10) Steinebach, C.; Ng, Y. L. D.; Sosič, I.; Lee, C.-S.; Chen, S.; Lindner, S.; Vu, L. P.; Bricelj, A.; Haschemi, R.; Monschke, M.; Steinwarz, E.; Wagner, K. G.; Bendas, G.; Luo, J.; Gütschow, M.; Krönke, J. Systematic Exploration of Different E3 Ubiquitin Ligases: An Approach towards Potent and Selective CDK6 Degraders. *Chem. Sci.* **2020**, *11* (13), 3474–3486. https://doi.org/10.1039/D0SC00167H.

Table of Intermediates

Cmpd	Structure
**30**	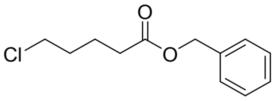
**31**	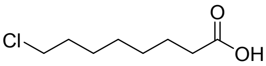
**32**	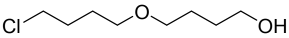
**33**	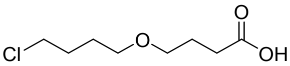
**34**	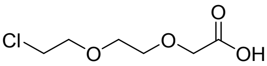
**35**	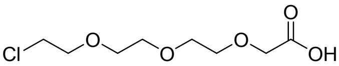
**36**	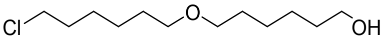
**37**	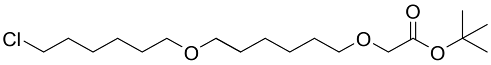
**38**	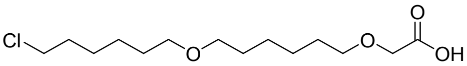
**39**	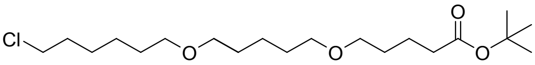
**40**	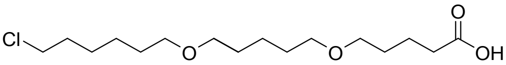
**41**	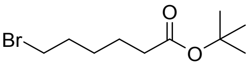
**42**	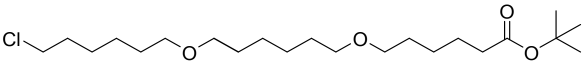
**43**	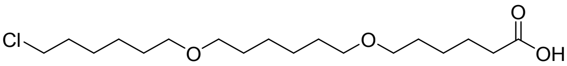
**44**	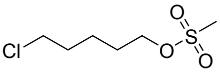

**45**	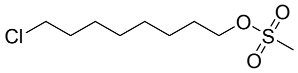
**46**	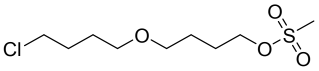
**47**	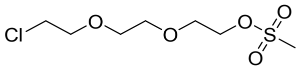
**48**	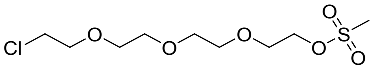
**49**	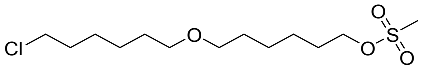
**50**	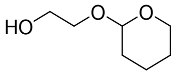
**51**	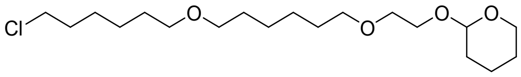
**52**	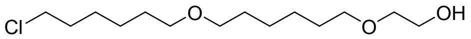
**53**	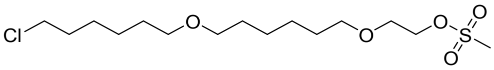
**54**	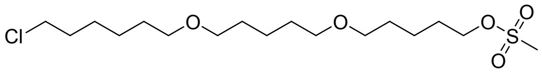
**55**	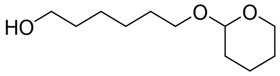
**56**	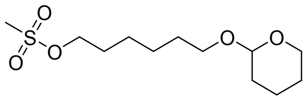
**57**	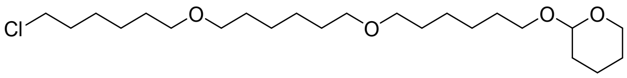
**58**	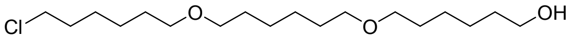
**59**	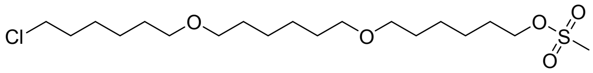

**60**	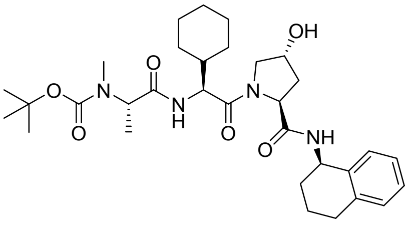
**61**	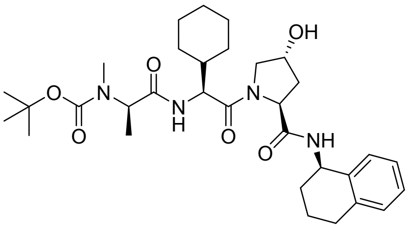
**62**	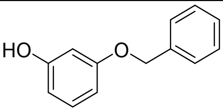
**63**	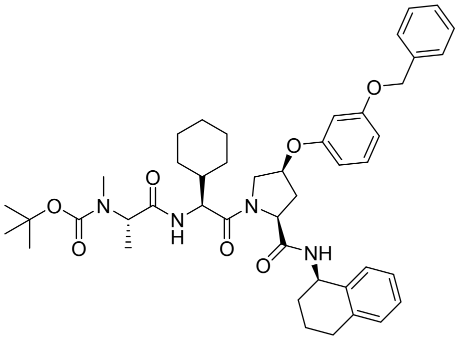
**64**	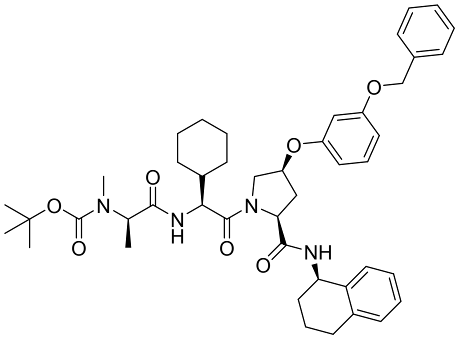
**65**	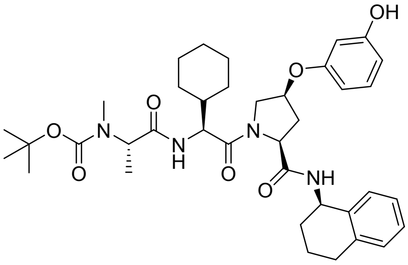

**66**	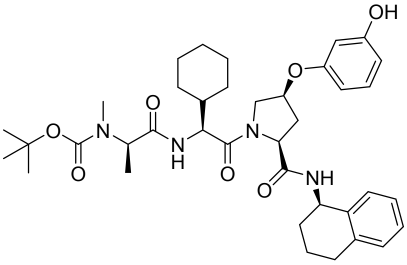
**67**	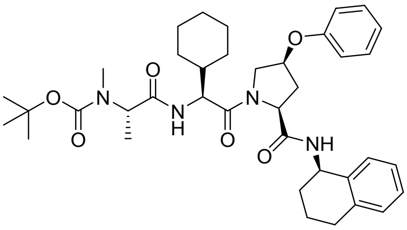
**68**	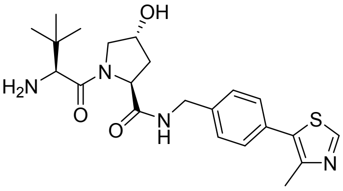
**69**	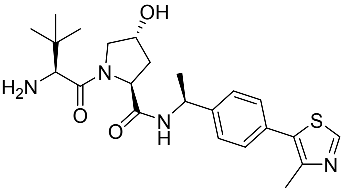
**70**	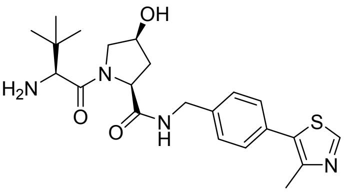
**71**	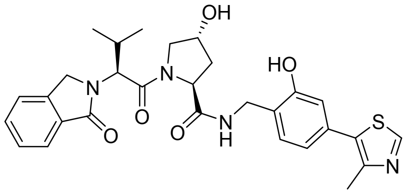
**72**	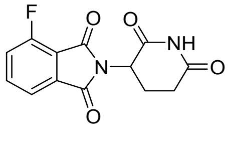

**73**	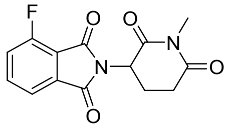
**74**	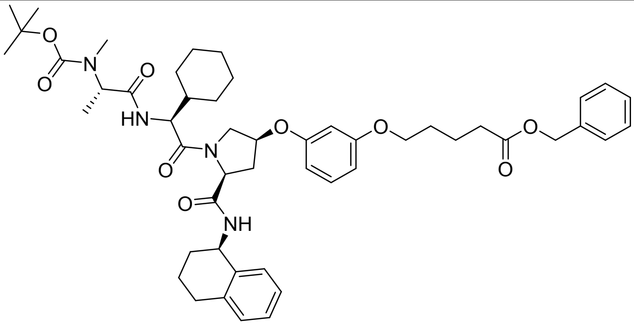
**75**	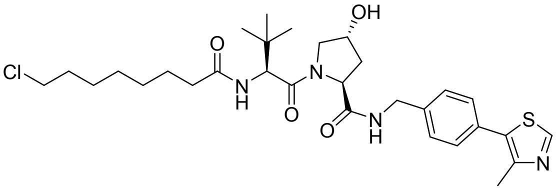
**76**	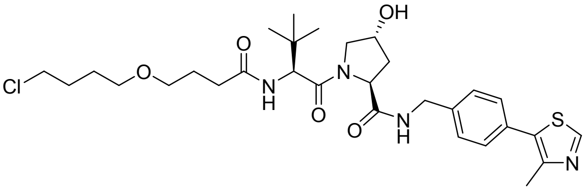
**77**	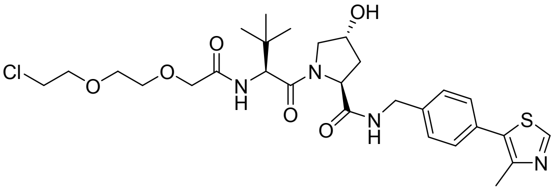
**78**	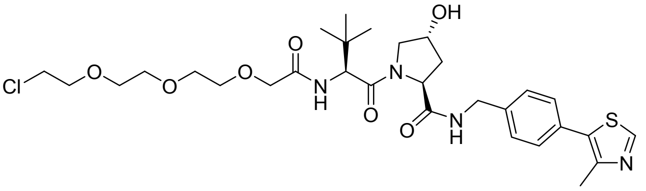
**79**	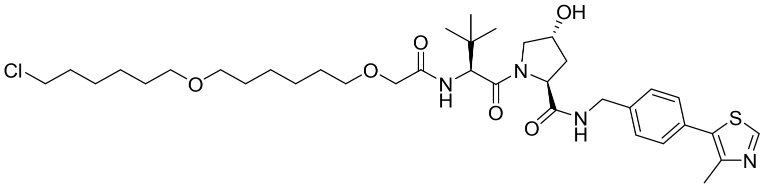

**80**	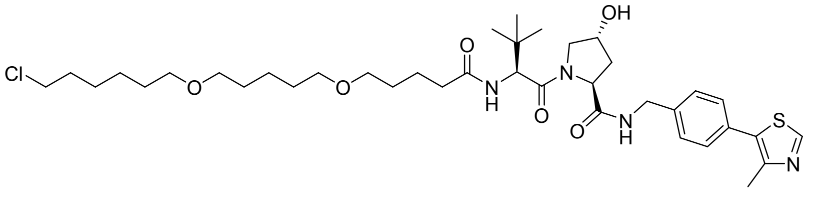
**81**	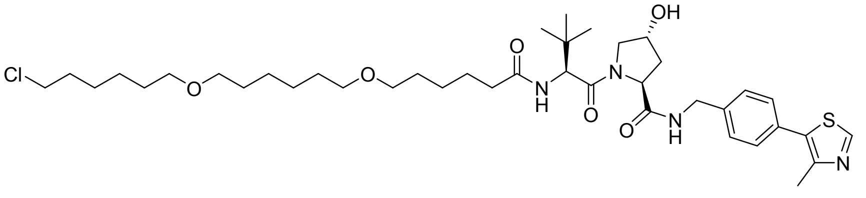
**82**	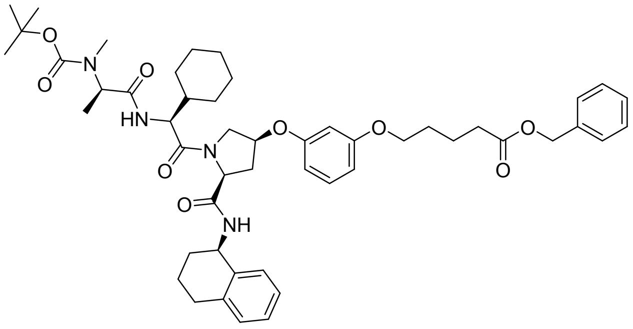
**83**	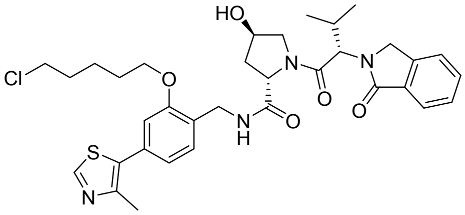
**84**	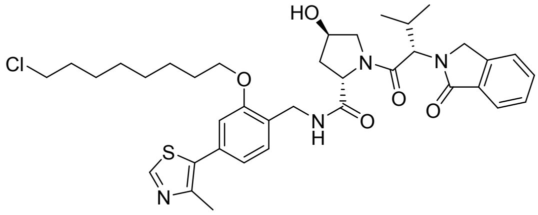
**85**	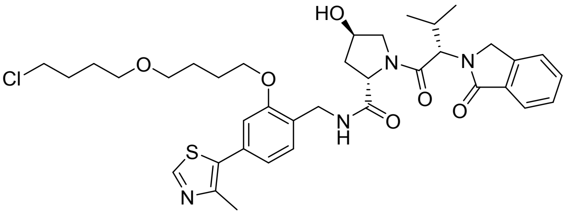

**86**	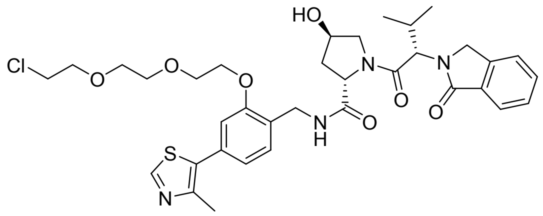
**87**	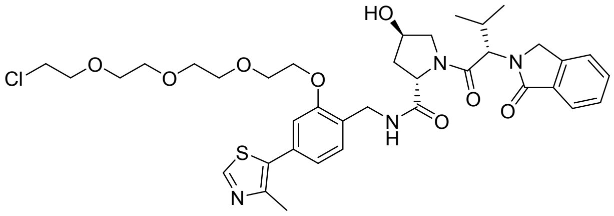
**88**	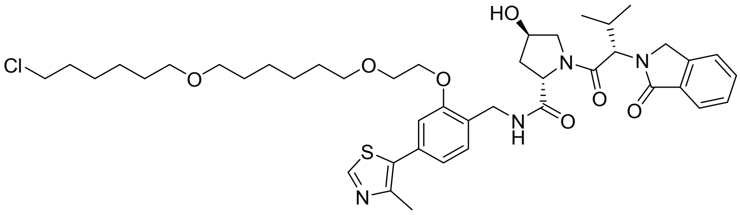
**89**	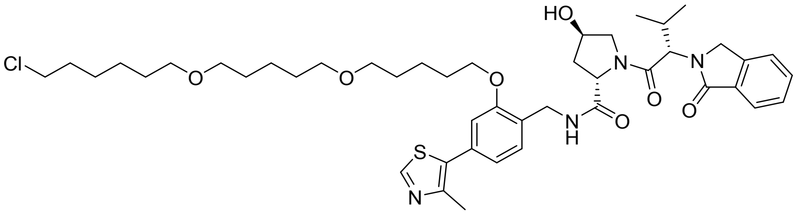
**90**	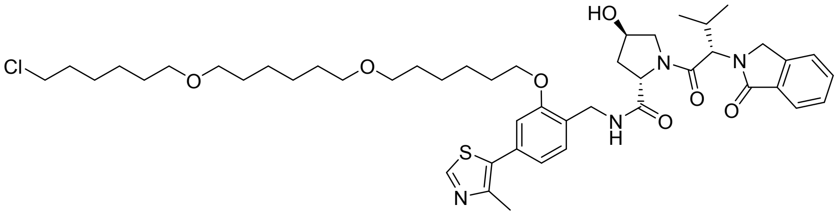
**91**	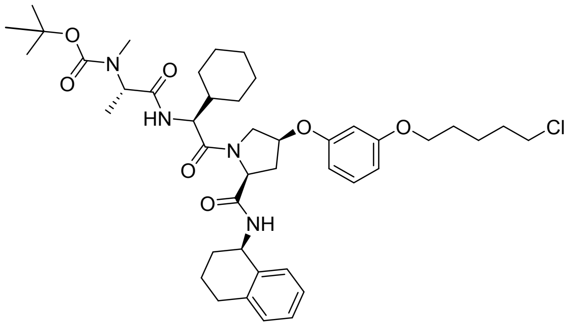

**92**	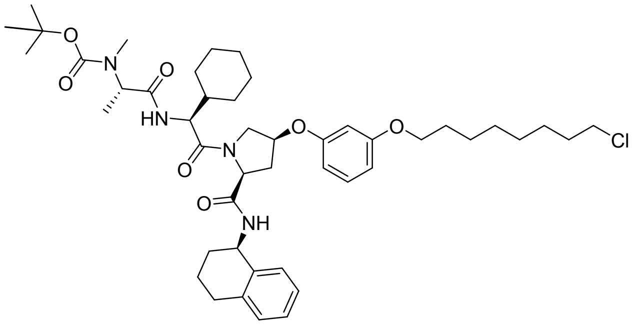
**93**	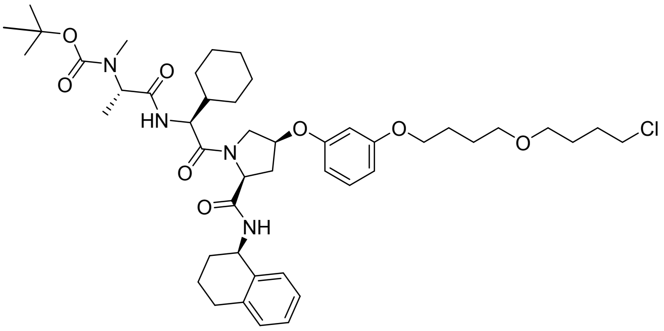
**94**	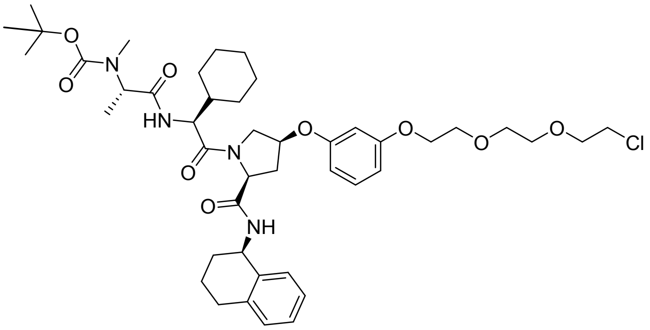
**95**	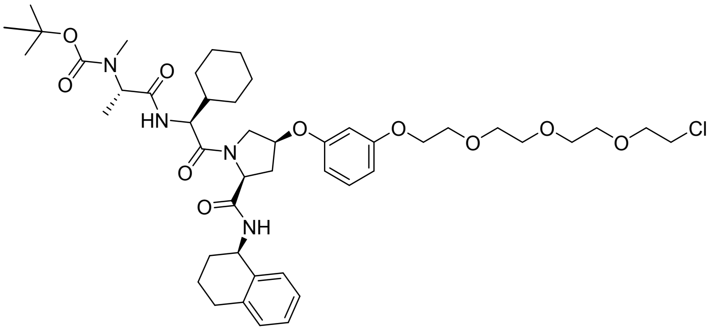

**96**	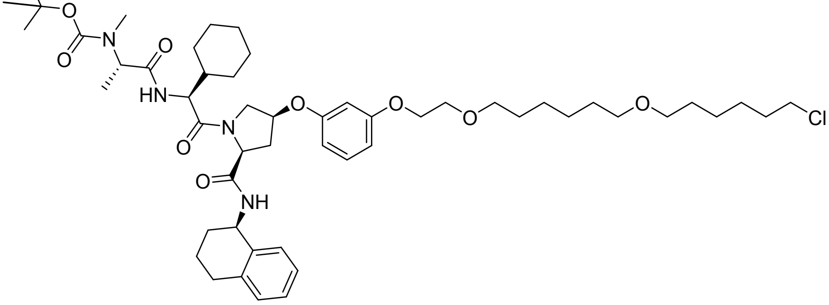O<br>N<br>O<br>O<br>HN<br>O<br>N<br>O<br>O<br>O<br>O<br>O<br>Cl<br>O<br>NH
**97**	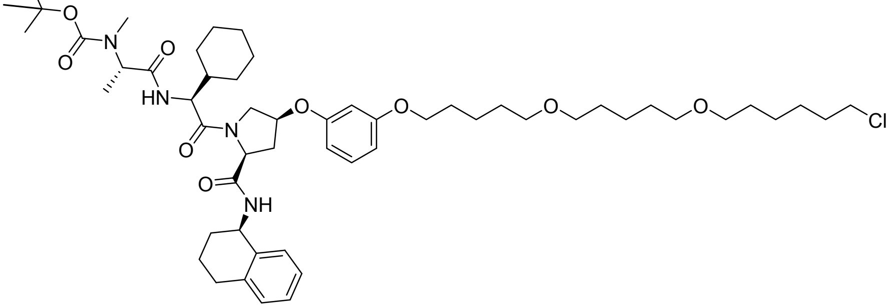O<br>N<br>O<br>O<br>HN<br>O<br>N<br>O<br>O<br>O<br>O<br>Cl<br>O<br>NH
**98**	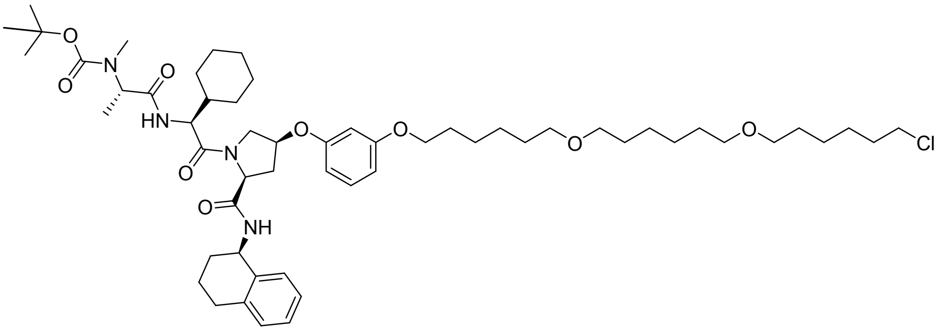O<br>N<br>O<br>O<br>HN<br>O<br>N<br>O<br>O<br>O<br>O<br>Cl<br>O<br>NH
**99**	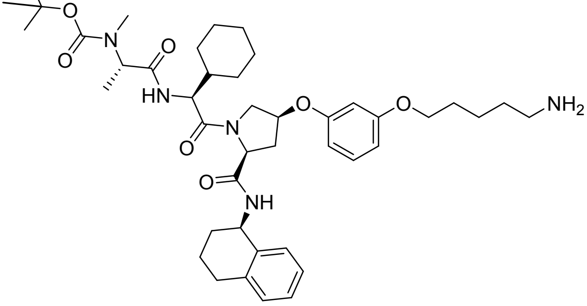O<br>N<br>O<br>O<br>HN<br>O<br>N<br>O<br>O<br>NH2<br>O<br>NH

**100**	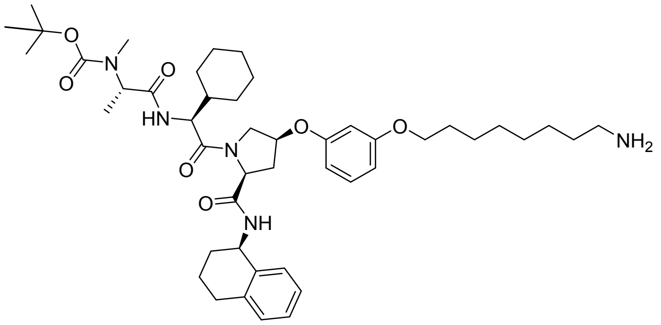
**101**	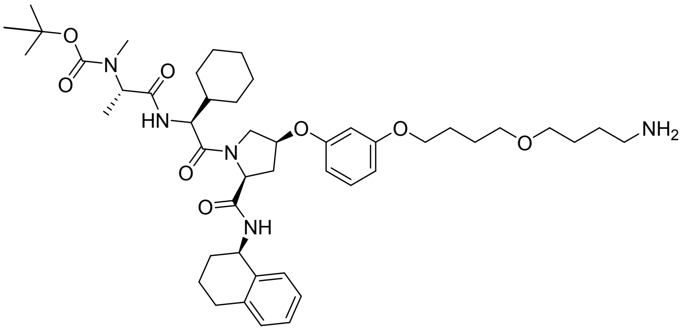
**102**	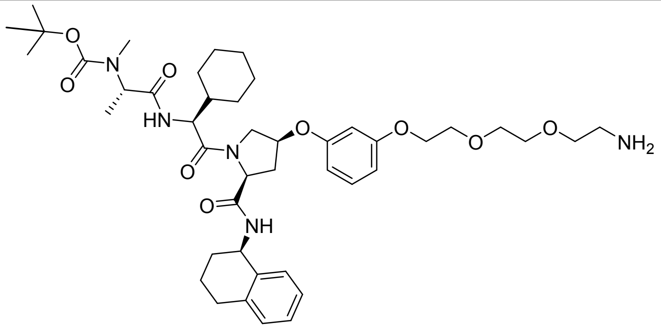
**103**	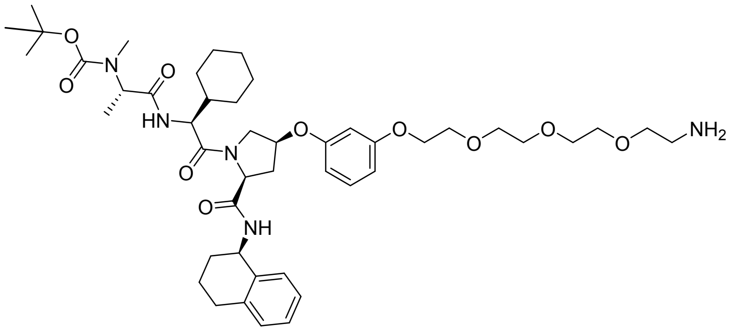

**104**	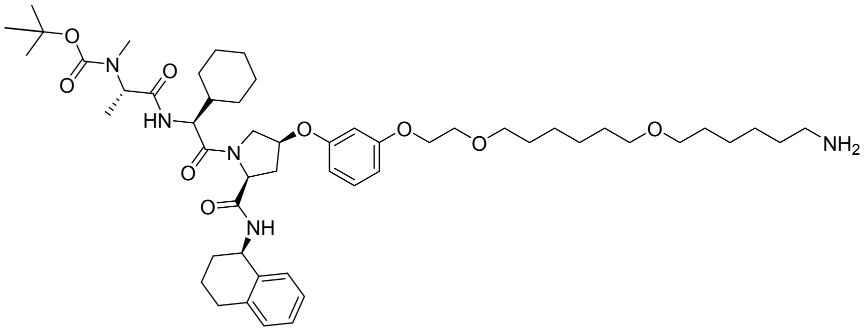
**105**	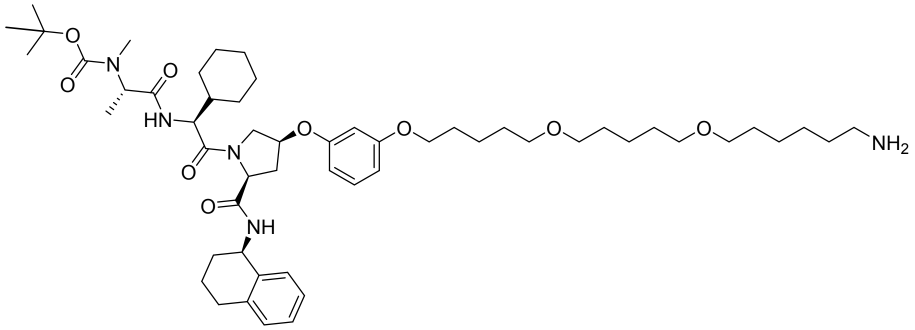
**106**	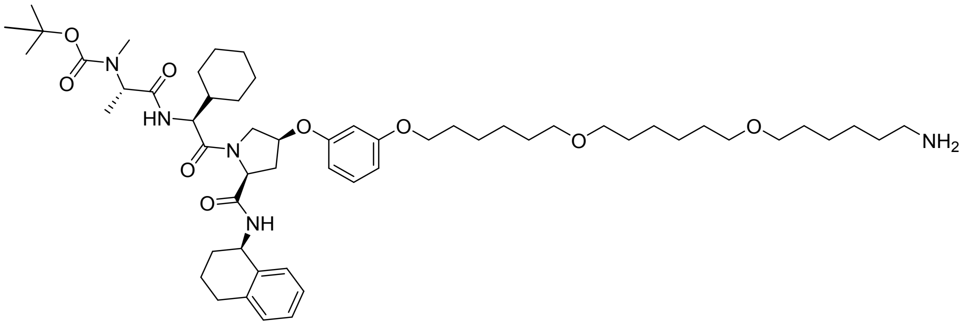
**107**	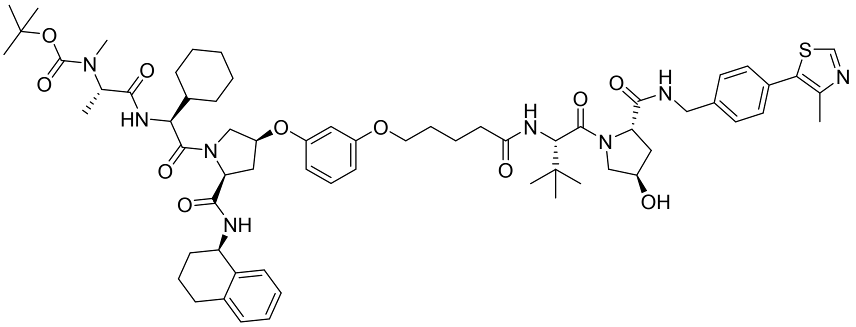

**108**	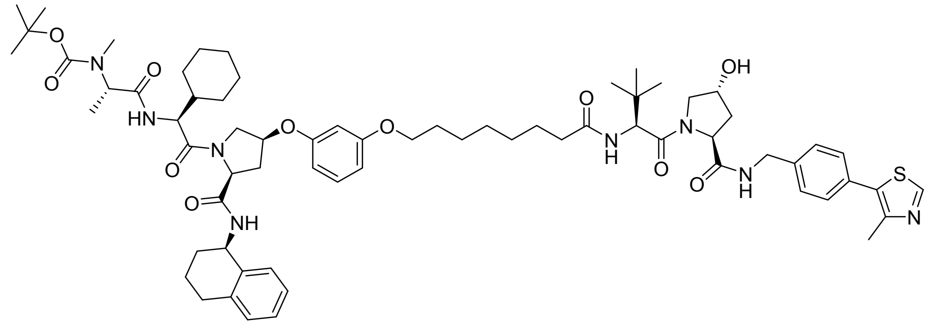
**109**	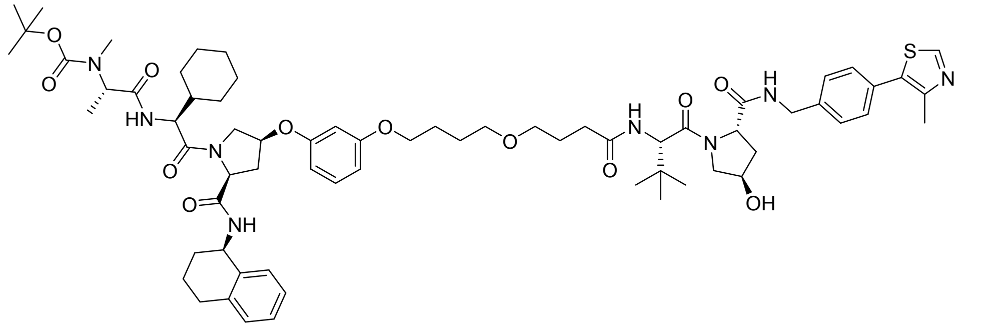
**110**	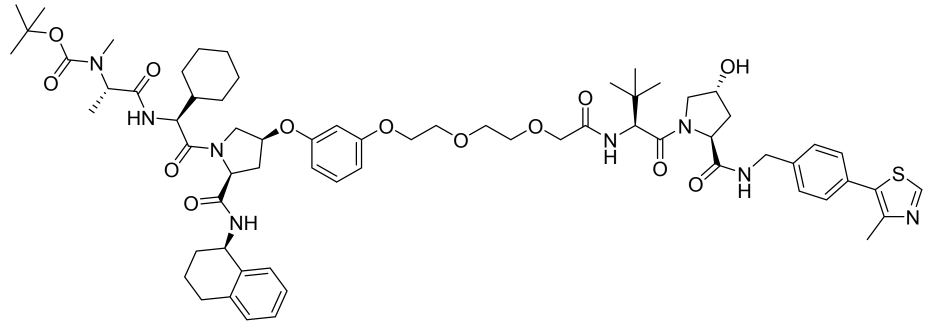
**111**	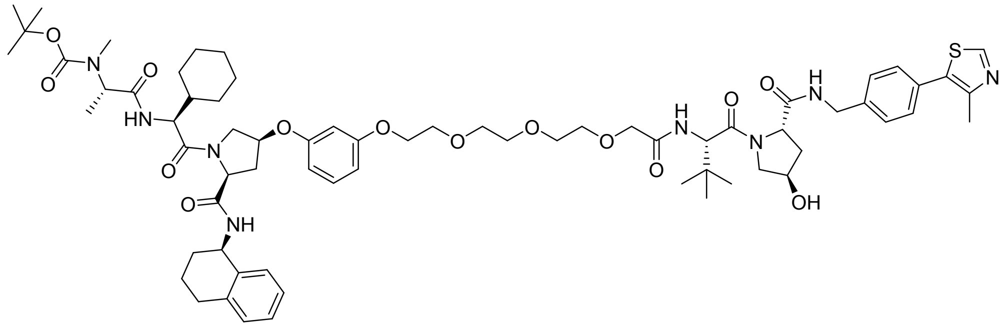
**112**	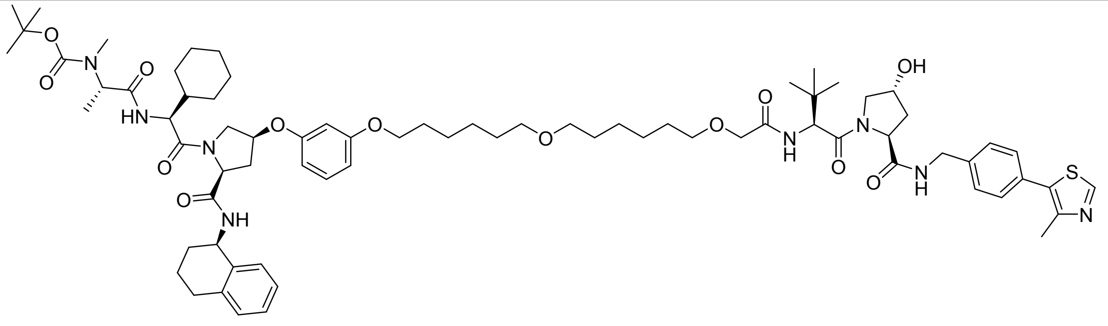

**113**	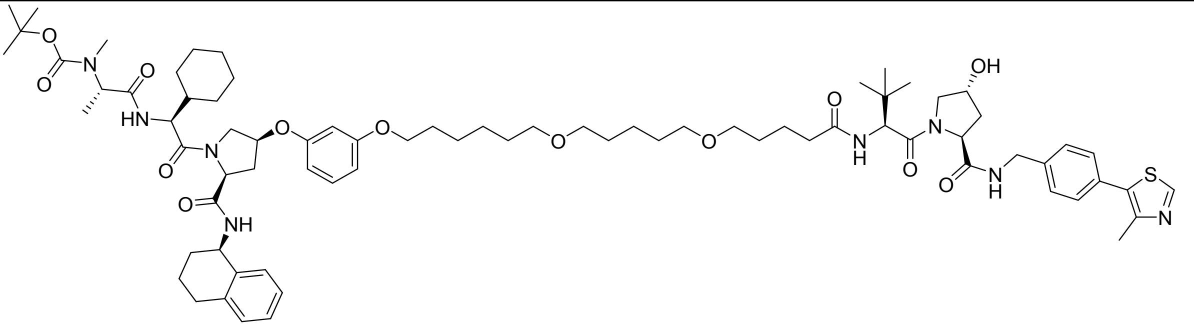
**114**	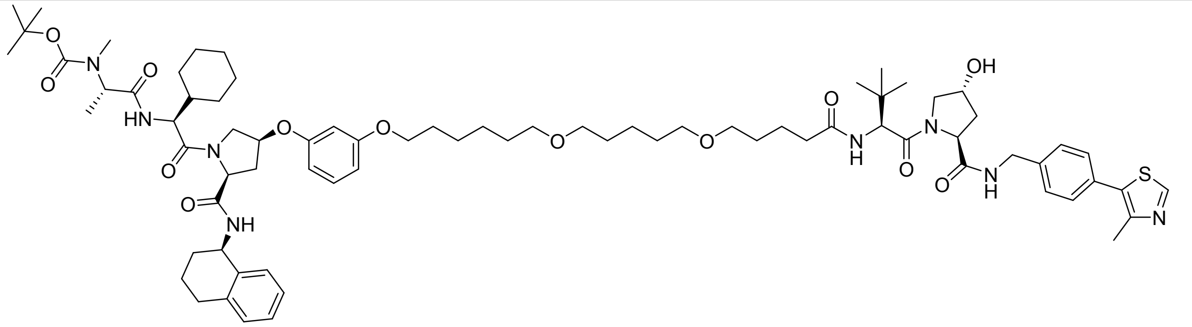
**115**	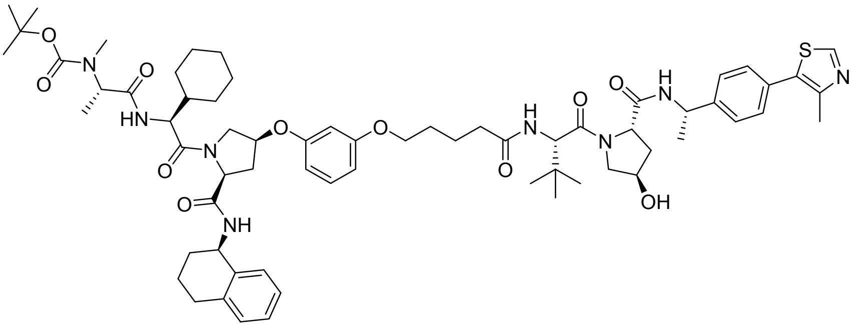
**116**	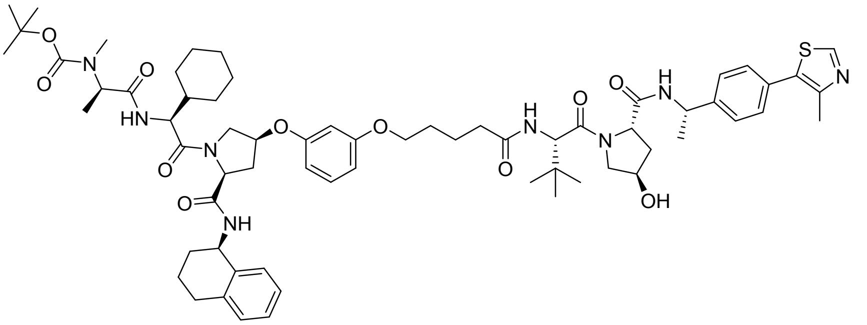

**117**	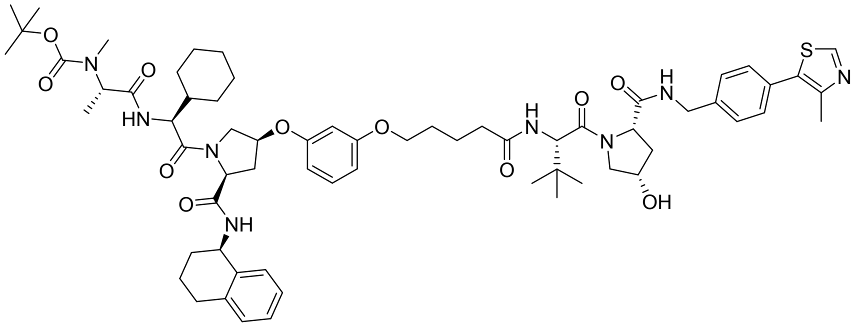
**118**	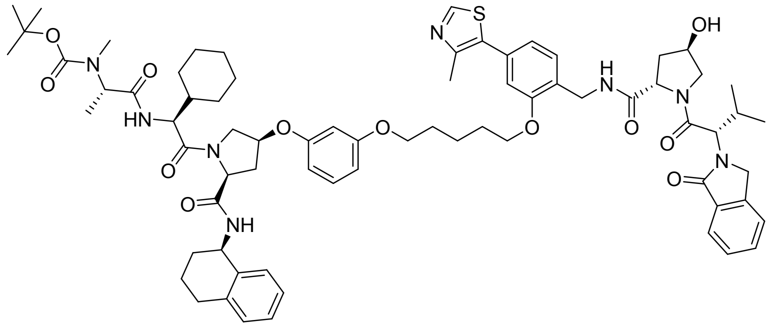
**119**	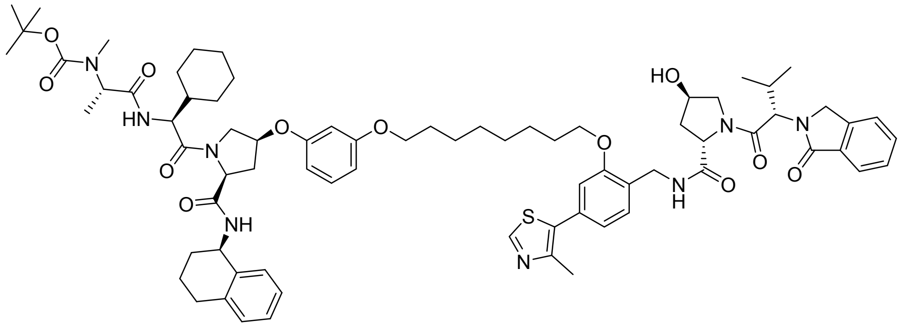
**120**	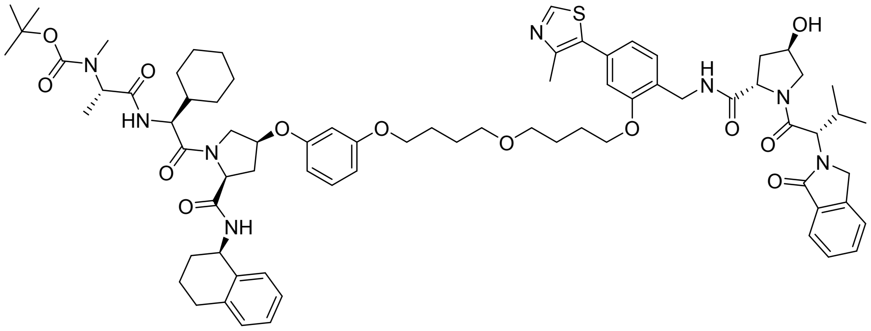

**121**	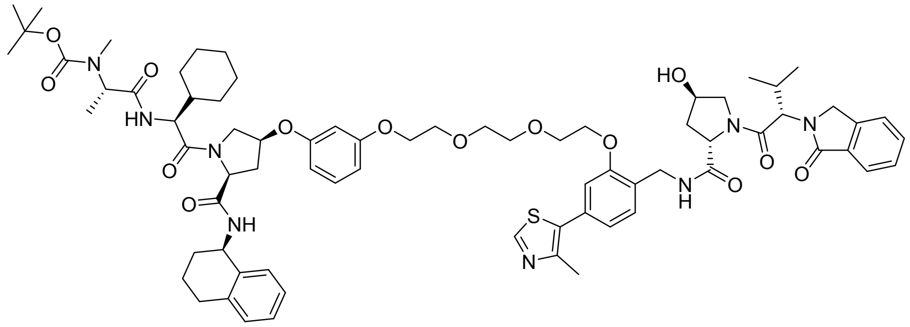
**122**	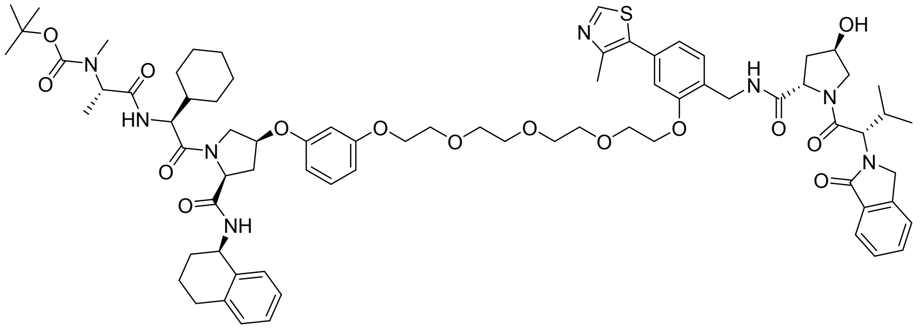
**123**	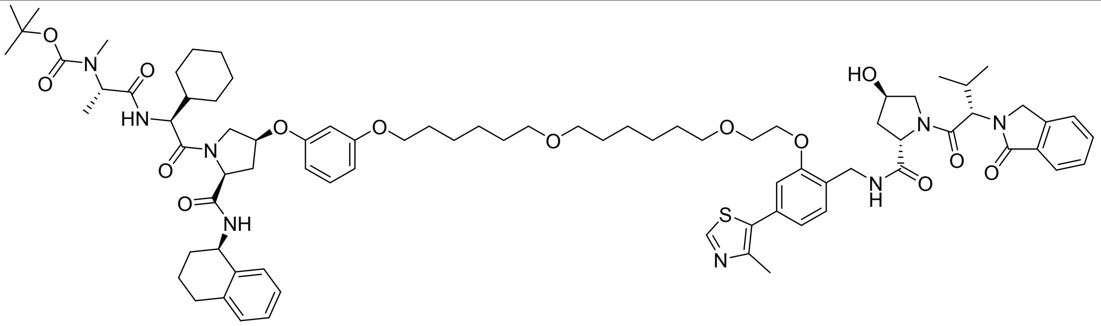
**124**	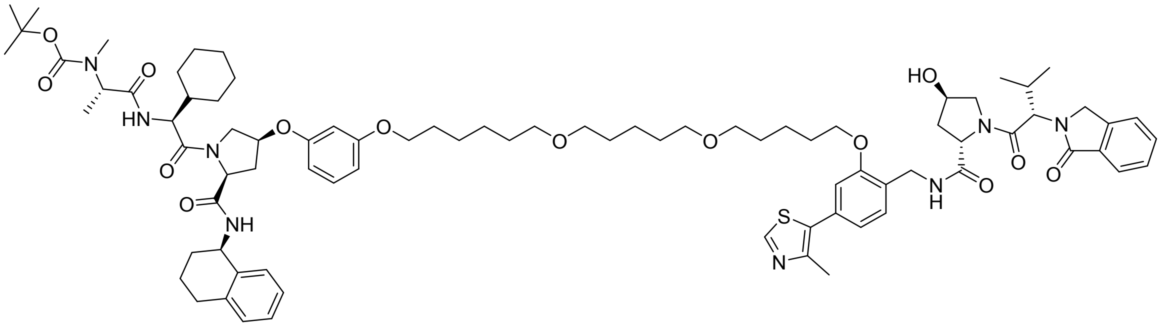

**125**	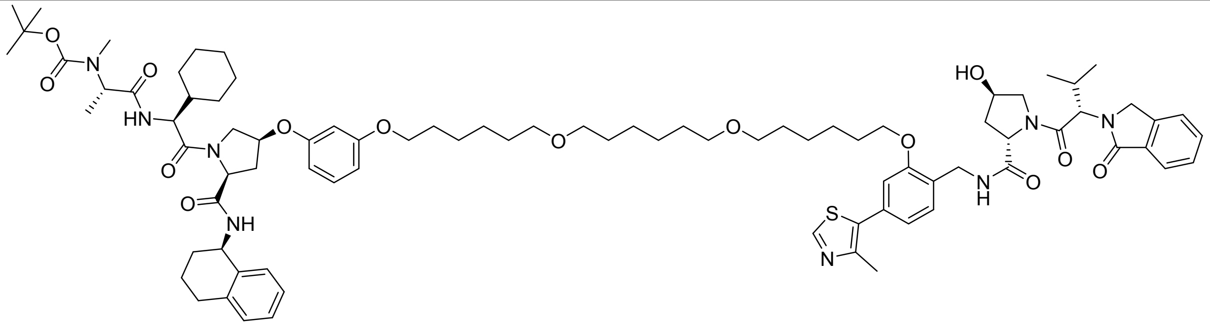
**126**	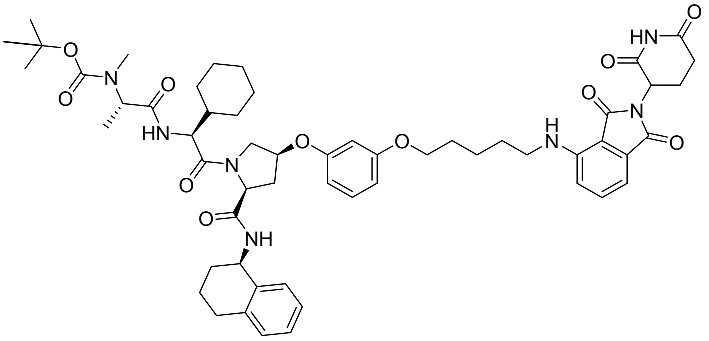
**127**	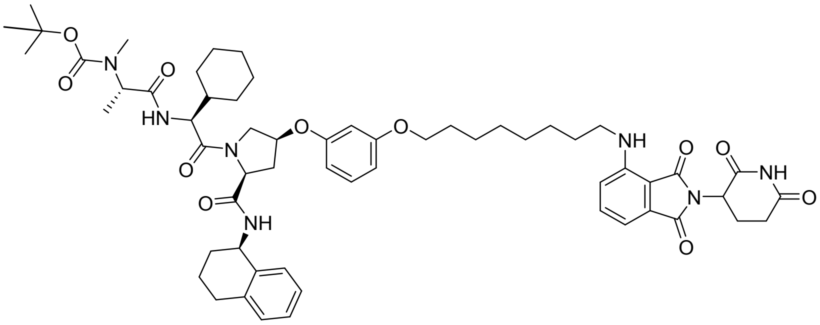
**128**	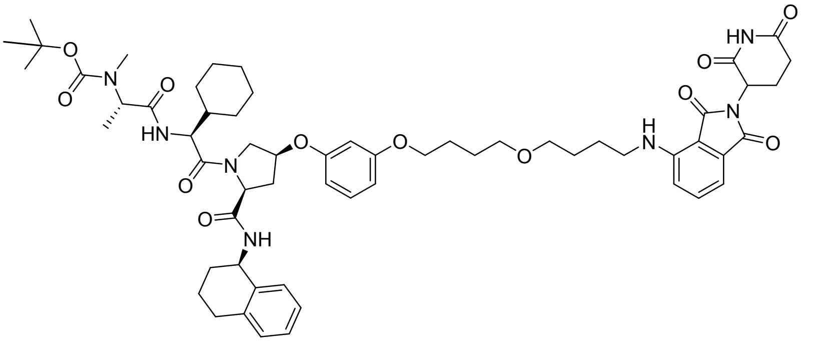

**129**	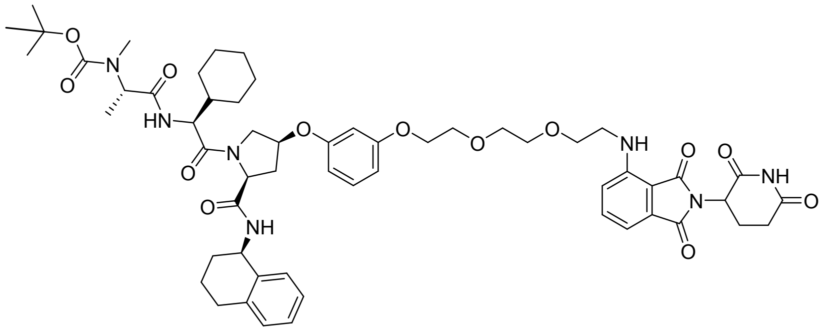
**130**	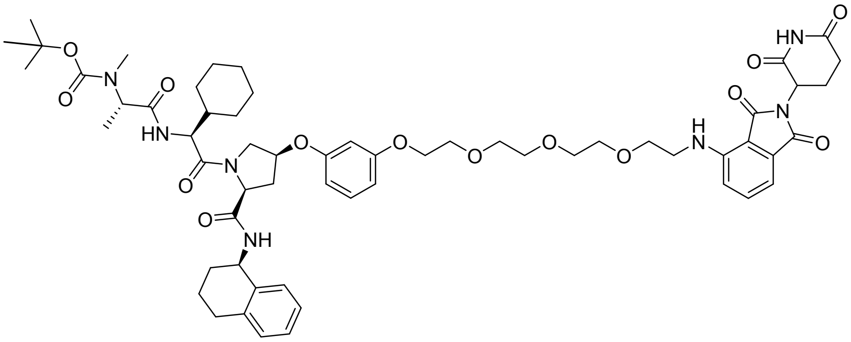
**131**	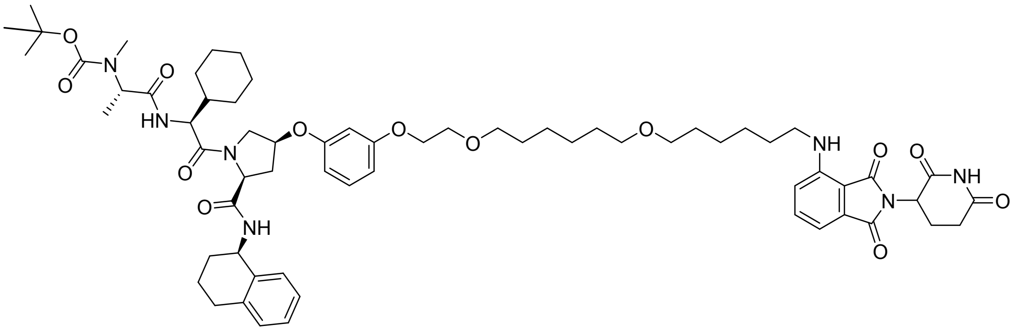
**132**	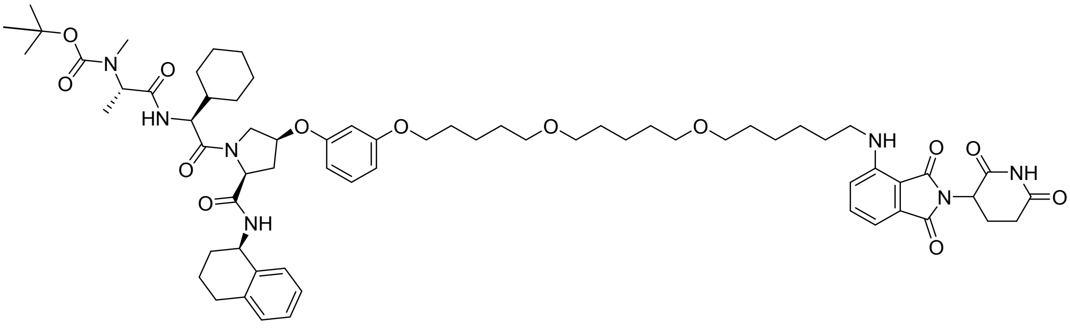

**133**	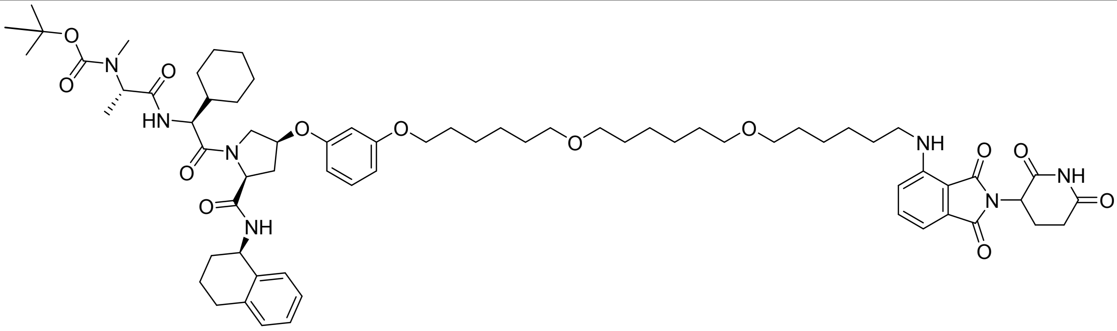O
N
O
O
HN
O
N
O
O
NH
O
O
O
NH
O
N
O
O
NH
O
**134**	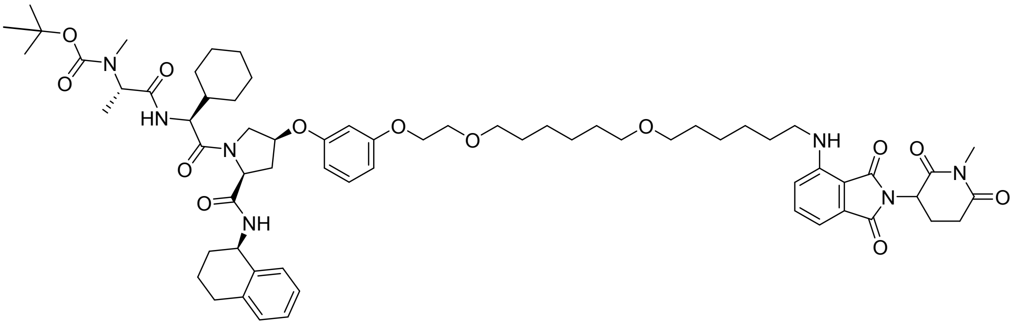O
N
O
O
HN
O
N
O
O
NH
O
O
O
NH
O
N
O
O
N
O
**135**	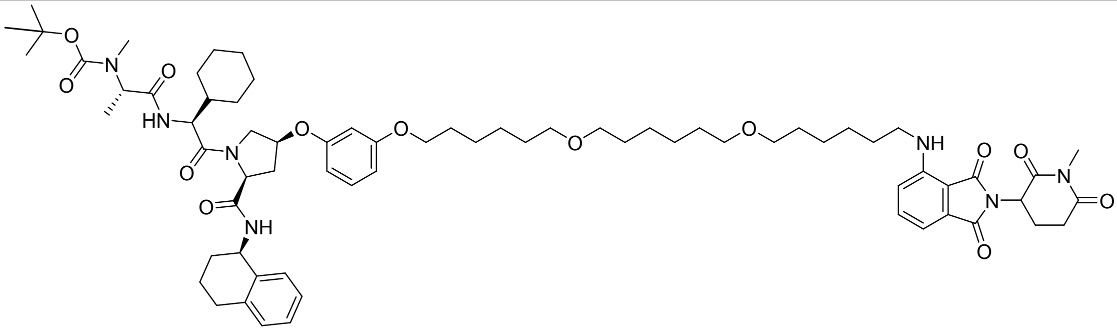O
N
O
O
HN
O
N
O
O
NH
O
O
O
NH
O
N
O
O
N
O
